# Allopatry as a Gordian Knot for Taxonomists: Patterns of DNA Barcode Divergence in Arctic-Alpine Lepidoptera

**DOI:** 10.1371/journal.pone.0047214

**Published:** 2012-10-11

**Authors:** Marko Mutanen, Axel Hausmann, Paul D. N. Hebert, Jean-François Landry, Jeremy R. de Waard, Peter Huemer

**Affiliations:** 1 Zoological Museum, Department of Biology, University of Oulu, Oulu, Finland; 2 Zoologische Staatssammlung München, Sektion Lepidoptera, München, Germany; 3 Biodiversity Institute of Ontario, University of Guelph, Guelph, Ontario, Canada; 4 Agriculture and Agri-Food Canada, Eastern Cereal and Oilseed Research Centre, Ottawa, Ontario, Canada; 5 Biodiversity Institute of Ontario, University of Guelph, Guelph, Ontario, Canada; 6 Tiroler Landesmuseen Betriebsges.m.b.H., Innsbruck, Austria; University of Rome, Italy

## Abstract

Many cold adapted species occur in both montane settings and in the subarctic. Their disjunct distributions create taxonomic complexity because there is no standardized method to establish whether their allopatric populations represent single or different species. This study employs DNA barcoding to gain new perspectives on the levels and patterns of sequence divergence among populations of 122 arctic-alpine species of Lepidoptera from the Alps, Fennoscandia and North America. It reveals intraspecific variability in the barcode region ranging from 0.00–10.08%. Eleven supposedly different species pairs or groups show close genetic similarity, suggesting possible synonymy in many cases. However, a total of 33 species show evidence of cryptic diversity as evidenced by the presence of lineages with over 2% maximum barcode divergence in Europe, in North America or between the two continents. Our study also reveals cases where taxonomic names have been used inconsistently between regions and exposes misidentifications. Overall, DNA barcodes have great potential to both increase taxonomic resolution and to make decisions concerning the taxonomic status of allopatric populations more objective.

## Introduction

Species delimitation is not straightforward. Young species pairs often show limited morphological and genetic divergence and decisions on their status are complicated because the acquisition of diagnostic characters does not always happen in the same order or at the same rate. As well, different species concepts emphasize different properties [1]–[2]. For example, the biological species concept highlights the importance of reproductive incompatibility, while the phylogenetic species concept only requires diagnosability. In practice, most species owe their description to the study of morphological variation, but some are based on the analysis of both molecular and morphological characters. The coupling of differing species concepts with variation in the characters examined has created an undesirable level of subjectivity in species delineation, particularly for taxa with allopatric ranges. Many species found in both alpine and arctic habitats fall into the latter category because their ranges are fragmented, reflecting the discontinuous distribution of the habitats that they occupy. Their disjunct distributions were gained through range shifts following deglaciation as rising temperatures provoked both the northward movement of populations and the shift of southern populations to higher elevations on mountains. Separated by broad zones of deciduous and boreal forest, gene flow between populations from the Alps and Scandinavia has now been halted for at least 10,000 years. Gene flow between North American and Eurasian conspecifics across the Beringian land bridge [3] was interrupted at about the same time, reflecting the postglacial flooding of the Bering Strait.

Few prior studies have examined the levels of sequence divergence across broad geographical areas in a large number of taxa. Past phylogeographic studies on arctic-alpine species have targeted single taxa, such as the moth *Zygaena exulans* [4] and the butterfly *Erebia epiphron* [5]. Prior barcoding studies have examined many species, but they have usually focused on relatively small geographic regions. Work on Central Asian butterflies [6] is exceptional as it examined patterns of sequence diversity on a larger geographic scale. It established that the performance of DNA barcodes in differentiating species was not significantly reduced as geographic coverage expanded. It also showed a high correspondence in levels of morphological and genetic differentiation between allopatric populations, suggesting that decisions concerning the status of allopatric species can be made in a more standardized way than in the past.

In this study, we examine levels and patterns of barcode divergence among 122 species of Alpine, Fennoscandian and Nearctic populations of Lepidoptera that have almost certainly experienced very limited or no gene flow for more than 10K years. We have also tested the potential value of DNA barcode data to aid the delineation of species.

## Materials and Methods

### Taxon Sampling and Nomenclature

We initially targeted about 170 artic-alpine species of Lepidoptera shared by Fennoscandia and the Alps, but we could only obtain representatives from 116 of these species, belonging to 26 families, from both areas (845 specimens in total, see Table S1 and Table S2 for details). We subsequently examined potential conspecifics from North America, analyzing 29 species with representatives from all three areas and 2 with arctic-alpine representatives, but absent from Fennoscandia (Table S1). We did not examine specimens from the subarctic or montane regions of Asia. Species assignments followed current taxonomy, which is mainly based on external morphology and genitalia. In total, we examined 1424 specimens with DNA barcode sequence data belonging to 122 species according to the nomenclature of Fauna Europaea [7] and Hodges *et al.* [8]. 1331 of these records are previously unpublished. Table S1 provides taxonomic authorities for all taxa.

### DNA Sequencing and Analysis

Sequences for the barcode region were obtained at the Canadian Centre for DNA Barcoding (CCDB) using standard protocols [9]. A full barcode sequence (658 bp) was recovered from 1180 specimens (118 species), sequences greater than 500 bp from 1399 specimens (121 species) and shorter sequences from 25 specimens (one species). Full length barcodes were recovered from at least one specimen for all species except *Stenoptilia alpinalis*, *S. buvati*, *Xestia rhaetica*, and *X. fennica*. Barcode records for the European specimens are available in the BOLD [10] dataset “DATASET-AALE1”, accessed at http://dx.doi.org/10.5883/DATASET-AALE1. Data for the North American specimens are available in the BOLD dataset “DATASET-ALNA1”, accessed at http://dx.doi.org/10.5883/DATASET-ALNA1. All sequences are also available on GenBank under the accession numbers provided in Table S1.

Sequence divergences were quantified using the Kimura 2-parameter model of nucleotide substitution calculated with the analytical tools on BOLD. We determined the maximum, mean and minimum intraspecific variation for each species and then separately for the three regions (Alps, Fennoscandia, North America) and between the regions. BOLD provides tools for calculating mean and maximum intraspecific variation values for each species, but analysis is based on species names so minimum and maximum distances within and between regions were obtained by assembling all pairwise values for each comparison type. For example, the mean divergence between Alpine and Fennoscandian populations involved the assembly of all pairwise distances for individuals from these two regions and then calculating their mean. Neighbor-joining (NJ) similarity trees were constructed with MEGA 5.05 [11] using the Kimura 2-parameter model of base substitution (with pairwise deletion of missing data).

## Results

### Molecular Divergence - Overview

**Figure 1:**
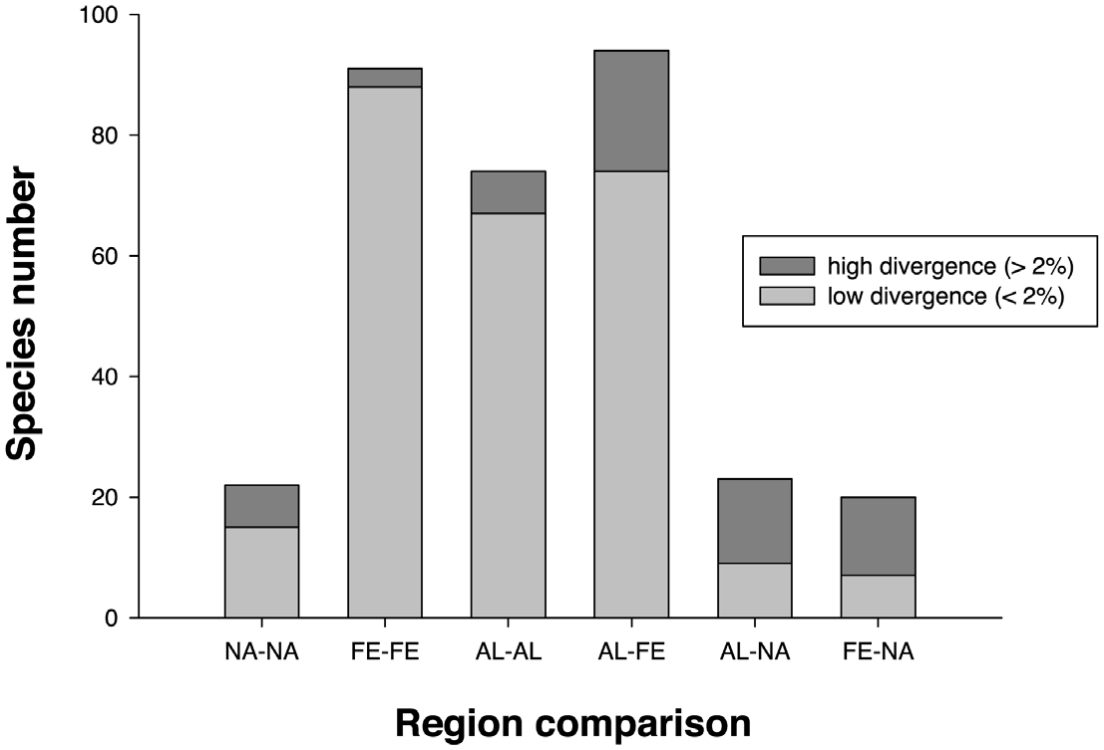
Proportion of high (>2%) intraspecific maximum divergences in DNA barcodes within and between regions. NA (North America), AL (Alps), FE (Fennoscandia).

**Figure 2:**
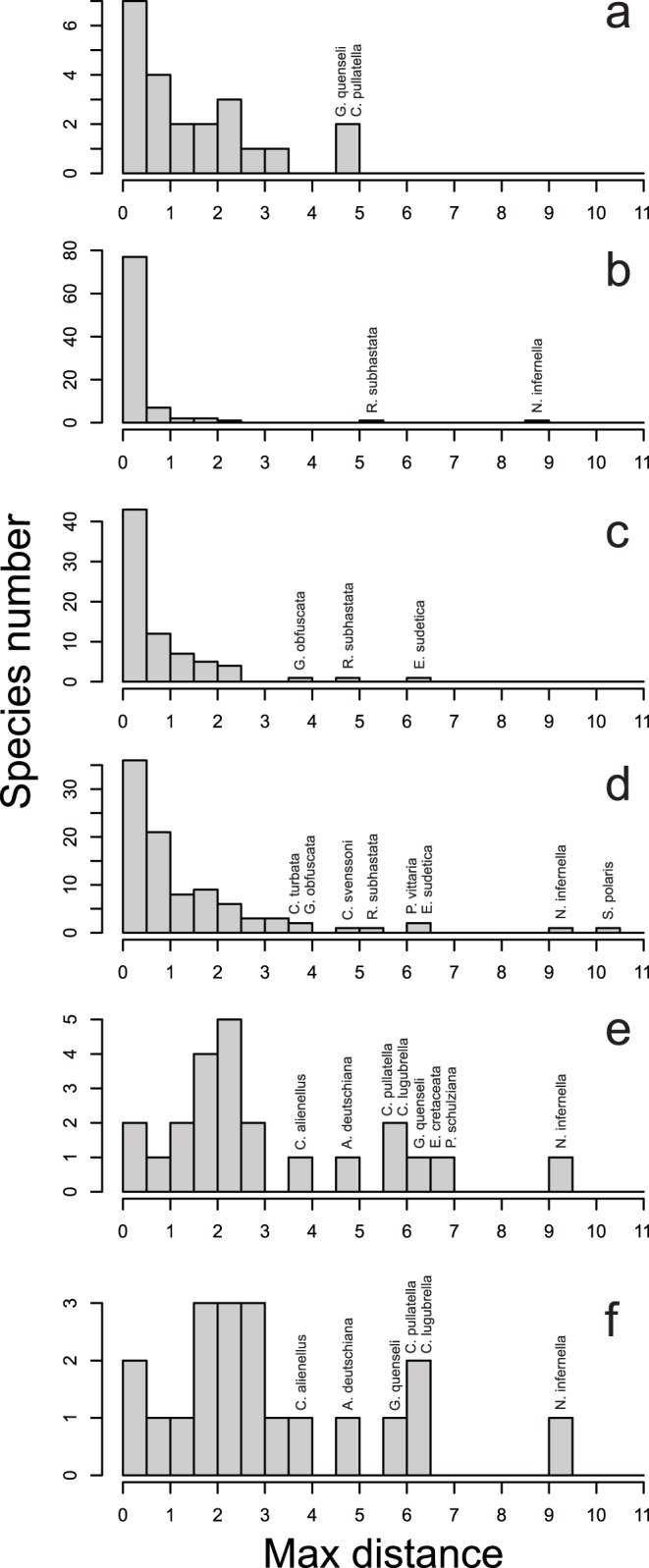
Distributions of maximum intraspecific barcode divergences (MAX) within and between the study regions. The x-axis shows MAX values in percentage. 2A. within North America, 2B. within Fennoscandia, 2C. within Alps, 2D. between Alps and Fennoscandia, 2E. between Alps and North America, 2F. between Fennoscandia and North America. Species showing over 4% intraspecific divergence are indicated above the bars.

Intraspecific variation averaged 1.08%, but ranged from 0.00%–10.08%. The mean value was increased by a few cases (discussed later) of probable cryptic species, most involving allopatric lineages. Intraspecific variation was considerably lower within single geographic regions, averaging 0.65% for North America, 0.39% for the Alps and 0.22% for Fennoscandia with levels of variation in the same rank order as the size of the regions (Figures 1 and 2). Mean intraspecific divergence was higher for comparisons between regions, averaging 1.03% between Fennoscandia and the Alps, 2.59% between the Alps and North America and 2.43% between Fennoscandia and North America (Figures 1 and 2). These values were roughly proportional to the distance between the areas, but precision of the comparison is impeded by taxonomic uncertainty (see below). Table S3 provides exact values of intraspecific variation within and between regions.

### Low Intraspecific Barcode Divergence

Sequence divergences higher than 2% in the barcode region often correspond to interspecific differences, while lower values are typical of intraspecific variation [12]. Of course, young sister-species may fall below the 2% threshold, while unusually variable species may exceed it. In our study areas, most species represented by multiple individuals followed the rule of less than 2% maximum divergence. In fact, 82 of 89 (92%) Fennoscandian species met this criterion and 78 of these species possessed less than 1% divergence. The same pattern was evident in Alpine specimens with 59 of 67 (88%) species falling below 2% divergence and 48 species falling below 1%. More intraspecific variation was apparent in North America as just 12 of 19 (63%) species fell below the 2% threshold. A comparison between the two regions in Europe showed that 66 of 86 (77%) species from both Fennoscandia and Alps had a maximum divergence below 2%, but the fraction of species with low divergence was much less for intercontinental comparisons. Only 6 of 18 (33%) species from Fennoscandia and North America and 6 of 19 (32%) species from the Alps and North America had a barcode divergence of less than 2%.

### Low Barcode Divergence in Different Taxa – Conspecificity *versus* Barcode Similarity

**Figure 3:**
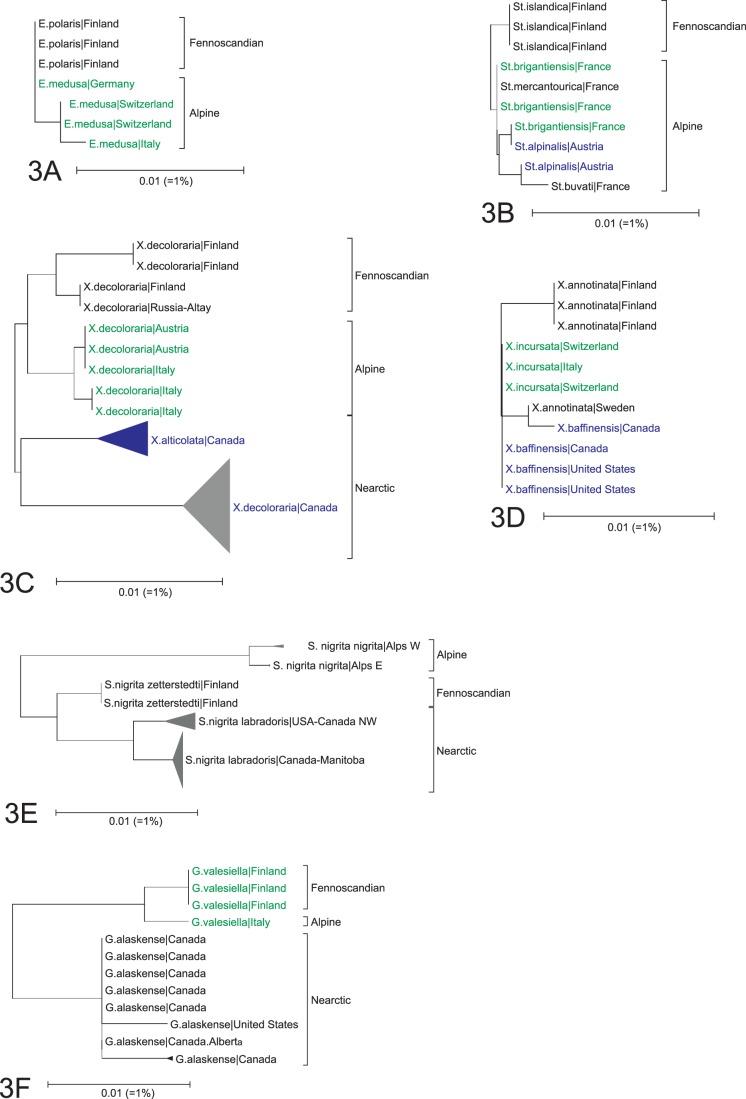
Examples of taxonomic findings. Taxa showing low interspecific divergence (3A. *Erebia polaris - E. medusa*, 3B. *Stenoptilia islandica - S. brigantiensis - S. mercantourica - S. alpinalis*, 3C. *Xanthorhoe decoloraria - X. alticolata*); taxonomic incongruence (3D. *Xanthorhoe annotinata - X. incursata - X. baffinensis*, 3E. *Sympistis nigrita* subspecies); support for the recent taxonomic revisions (3F. *Gnorimoschema valesiella - G. alaskense*).

Our analyses reveal 11 possible cases of overlooked synonymy (as indicated by close barcode similarity) including five species pairs and one quintet in Europe (Figure 3, Table S4). Five species pairs (*Erebia medusa - E. polaris, Oeneis glacialis - O. norna*, *Holoarctia cervini - H. puengeleri*, *Apamea maillardi - A. schildei*, *Xestia fennica - X. rhaetica*) showed less than 1% maximum sequence divergence between the Alps and northern Europe (Figure 3A). As well, a group of five species, including the Fennoscandian *Stenoptilia islandica* and four Alpine endemics (*S. alpinalis, S. buvati, S. brigantiensis, S. mercantourica*) showed less than 1% divergence (Figure 3B). As all these taxa are all extremely similar in external morphology and genitalia, their species rank needs reconsideration. In fact, the status of most of these taxa is controversial. For example, *Xestia rhaetica* was treated as a subspecies of *X. fennica* by Kullberg *et al.* [13], but as a separate species by Fibiger *et al.* [14]. The latter authors viewed *Apamea schildei* as distinct from *A. maillardi,* reversing a decision on its subspecific status made just a few years earlier [15]. Similarly, *Holoarctia cervini* and *H. puengeleri* have traditionally been viewed as subspecies, but more recently as separate species [16]–[17]. The taxonomic status of *Stenoptilia* is also uncertain as the four alpine taxa in our study have been treated both as junior synonyms of *S. pelidnodactyla* [18] and as distinct species [19]. Our data provide another option – their possible synonymy with *S. islandica* (Figure 3B). The species pairs of *Erebia* and *Oeneis* provide a final example of low genetic divergence and strong morphological similarity between allopatric lineages.

We encountered one triad and four species pairs where specimens from our three study areas showed low divergence. The group of three species included *Xanthorhoe incursata* from the Alps, *X. annotinata* from Finland and *X. baffinensis* from North America (Figure 3D). They showed very low divergence (MIN = 0.08%, MAX = 0.66%), despite the fact that the two European taxa have very different genital morphology. The four species pairs included:

1. European lineages of *Xanthorhoe decoloraria* and North American *X. alticolata* had little divergence (MIN = 0.77%) (Figure 3C). Interestingly, European and North American lineages of *X. decoloraria* showed more divergence (MIN = 1.55%).
2. *S. nigrita* from the Alps had substantial divergence (MIN = 2.34%) from Fennoscandian lineages of this species, while North American *Sympistis zetterstedtii* were very close (MIN = 1.08%) to Finnish *S. nigrita* (Figure 3E). The taxonomy of these species has been controversial. Skou [20] treated *S. zetterstedtii* and *S. nigrita* as distinct species, but Ronkay & Ronkay [21] noted the genitalic similarity of *S. nigrita* and *S. zetterstedtii* versus the marked morphological difference between alpine and Fennoscandian *S. nigrita*. On this basis they recognized four subspecies: *S. nigrita nigrita* (Alps), *S. nigrita zetterstedtii* (northern Eurasia), *S. nigrita sibirica* (Central Asia) and *S. nigrita labradoris* (Nearctic). Lafontaine & Schmidt [22] supported this decision, but suggested that *S. nigrita zetterstedti* was also present in the far north of Canada.
3. *Coenophila subrosea* in Europe and its North American sister taxon, *C. opacifrons,* show little barcode divergence (MIN = 1.29%) and prior taxonomic publications have treated them both as subspecies and as distinct species [23].
4. The taxonomy of *Apamea zeta* is complex. Based on deep barcode divergences (MIN = 3.0%) between populations from the Alps and North America, Zilli *et al.* [15] partitioned the *zeta* complex into four species with *A. zeta* restricted to the mountains of southern and central Europe, while *A. exulis* was found only in Canada. By contrast, Mikkola *et al.* [24] recognized five subspecies of *A. zeta* in North America, treating *A. exulis* as one of these. They did, however, note that *A. zeta* was the most polymorphic species of *Apamea*, and that the status of its various forms and populations was uncertain. Fibiger *et al.* [14] came to a different conclusion, recognizing several Eurasian species with *A. exulis* as a circumboreal species with more than 2% barcode divergence from *A. zeta*.

### High Barcode Divergences in Single Taxa – Potential Cryptic Species

Prior studies have shown that barcode divergences of more than 2% are often an indication of overlooked species or misidentifications. For example, *Gnorimoschema valesiella* and *G. alaskense* were formerly treated as synonyms, but were recently separated because of their differing genitalic morphology [25]. Barcode data support this decision as these taxa show a bit over 2% mean divergence (MIN = 1.62%) (Figure 3F). For brevity, in the balance of this section, we only discuss taxa with more than 4% maximum divergence, where the likelihood of cryptic taxa is high. However, additional overlooked species are likely among other species, especially those with maximum divergences in the 2–4% range.

### Divergence Within Regions

#### Fennoscandia

**Figure 4:**
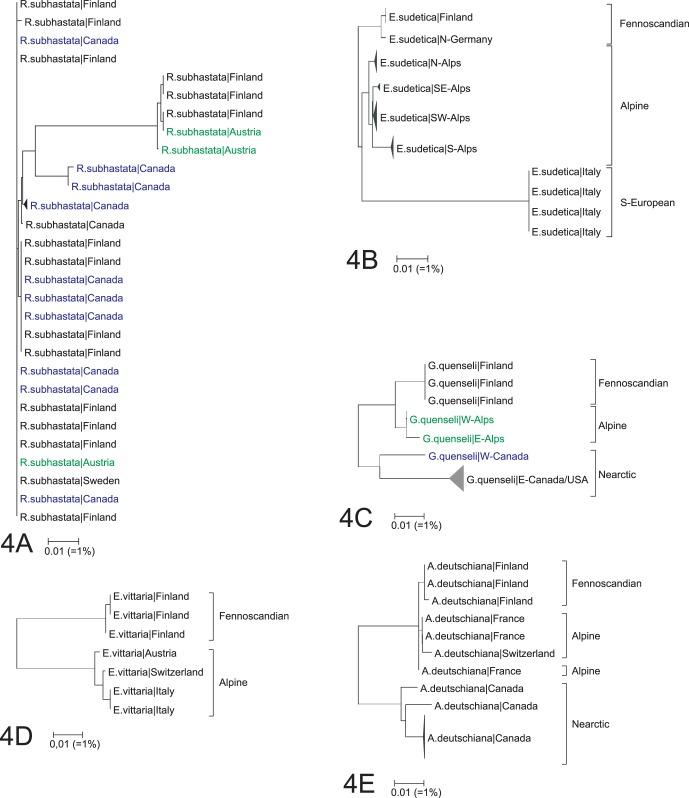
Species showing deep intraspecific splits within or between regions. 4A. *Rheumaptera subhastata*, 4B. *Eudonia sudetica*, 4C. *Grammia quenseli,* 4D. *Elophos vittaria*, 4E. *Aethes deutschiana*.

One of the 91 species from Fennoscandia possessed a maximum divergence greater than 4% - *Rheumaptera subhastata* (MAX = 5.07%, Figure 4A). It split into two barcode clusters, both shared with alpine populations in Europe, but only one of these lineages was detected in North America.

#### Alps

Two of the 74 species from the Alps had a barcode divergence greater than 4%. *Rheumaptera subhastata* (MAX = 4.75%) was divided into the same two clusters found in Fennoscandia, while *Eudonia sudetica* (MAX = 6.32%, Figure 4B) included two barcode clusters with allopatric distributions. Most alpine specimens belonged to the same barcode cluster as those in Fennoscandia, but specimens from Central Italy belonged to a second cluster that is likely an undescribed cryptic species.

#### North America

Two of 22 species from North America showed more than 4% barcode divergence: *Caryocolum pullatella* (MAX = 4.56%) and *Grammia quenseli* (MAX = 4.59%, Figure 4C). *C. pullatella* has been recognized as a probable cryptic species complex (Huemer [26] and our barcode studies revealed two barcode lineages in North America and another two in Europe. North American specimens of *G. quenseli* also split into two divergent barcode clusters distinct from those in Europe, but their status is uncertain as Schmidt [27] found that mitochondrial markers were of limited taxonomic value in *Grammia* due to frequent hybridization and introgression.

### Divergence Between Regions

#### Fennoscandia – Alps

Four of 94 species analyzed from Fennoscandia and the Alps showed greater than 4% divergence: *Coleophora svenssoni* (MAX = 4.67%), *Synanthedon polaris* (MAX = 10.08%), *Eudonia sudetica* (MAX = 6.32%), and *Elophos vittaria* (MAX = 6.47%, Figure 4D). We expect that most, if not all, of these cases involve overlooked species. For example, intraspecific divergence is reduced below 2% in *E. sudetica* once the divergent populations from Central Italy are excluded. Specimens of *S. polaris* from the Alps show close barcode similarity to those from Sweden and Norway, but not with specimens from Finland. This case involves two previously recognized species which are currently accepted as synonyms.

#### Alps/Fennoscandia – North America

Five of 23 species found in both the Alps and North America showed barcode divergences greater than 4%: *Caryocolum pullatella* (MAX = 5.63%), *Chionodes lugubrella* (MAX = 5.95%), *Aethes deutschiana* (MAX = 4.93%, Figure 4E), *Grammia quenseli* (MAX = 6.12%, Figure 4C) and *Eupithecia cretaceata* (MAX = 6.83%). The same pattern of deep divergences was also seen in comparisons involving these species from Fennoscandia and North America, excepting *E. cretaceata* which was not collected in Fennoscandia.

### Corrected Misidentifications

Our barcode studies revealed three cases of deep intraspecific barcode divergence and one case of barcode sharing that were later found to arise from misidentifications.

1. Specimens of *Olethreutes schulziana* from North America fell into two barcode clusters with a maximum divergence of 7.68%. Subsequent dissection and literature comparison [28], [29] revealed that one of the groups was actually *O. inquietana*. After this adjustment, specimens of *O. schulziana* from North America show low divergence (MAX = 0.16%), but considerable divergence from their European counterparts (MIN = 1.55%).
2. Specimens of *Olethreutes septentrionana* from Finland showed marked barcode divergence from those in the Alps (MAX = 8.57%). The northern European specimens formed a novel barcode cluster, but the sole specimen from the Alps shared its barcode with *O. palustrana*. Detailed analysis, including the dissection of additional specimens and literature survey [29], [30], revealed that alpine specimens identified as *O. septentrionana* are actually *O. palustrana*. *O. septentrionana* is known from montane sites in Poland [29], but it seems absent from the Alps.
3. *Neofaculta infernella* included two barcode clusters (MAX = 8.88%) in Fennoscandia, while just one occurred in the Alps. Barcoding of Siberian *N. taigana* material, including type material (unpublished records) and examination of the original description of *N. taigana* [31] suggested that specimens in the cluster from Fennoscandia shared with North America actually belong to another species - *N. taigana*.
4. Specimens from Finland thought to represent *Ancylis rhenana* shared identical barcodes with a specimen of *A. habeleri* from the Alps. These two species are best identified by external morphology, and based on forewing coloration and patterns [29]–[32] we conclude that supposed *A. rhenana* from Finland are actually *A. habeleri.*

## Discussion

DNA barcoding has proven an efficient tool for both differentiating animal species and revealing cryptic diversity [12], [33]–[37]. In groups with well-established taxonomy, such as European Lepidoptera and North American birds, identification success has been strong [12], [34], [38]. Most reports of low success have involved groups which were known to be taxonomically problematic [39]. Other apparent cases of failure have arisen from flawed taxonomy or from misidentification [40]–[42]. Our study revealed four cases of misidentification which would have led, if not corrected, to apparent barcoding failures. As our work involved one of the best known groups of insects and the resolution of each case required substantial effort, it is probable that many other cases of misidentification have been overlooked.

Few studies have examined patterns of barcode variability over large geographic areas, and fewer still have examined divergences between allopatric populations or sister species. Past studies have shown that wider geographic sampling usually increases the amount of intraspecific variation, but that it often has little effect on identification success because the incremental variation typically erodes the barcode gap rather than producing overlap between species [6]. However, there are exceptions. Broader geographic sampling reduced both identification success and the number of monophyletic species in a group of closely related beetles [43]. Further studies of this sort are needed because taxonomic complexity rises as the scale of geographic coverage expands. Species with discontinuous ranges present a particular challenge because some species concepts cannot be employed. For example, the biological species concept can only be applied to sympatric taxa because range overlap is required to assess the presence or absence of gene flow. Other species concepts, such as the differential fitness concept [2], are theoretically applicable, but would necessitate breeding experiments with allopatric populations. The phylogenetic species concept has the advantage of being easily applied to allopatric lineages as it only requires that species be diagnosable clusters of individuals with shared ancestry [44]. However, few taxonomists favour the recognition of species that can only be discriminated by genetic markers [45]. Moreover, strict application of the phylogenetic species concept would lead to a tremendous increase in species numbers [46]. For example, many of the allopatric populations that we examined possessed diagnostic barcodes, qualifying them for recognition as separate species under the phylogenetic species concept.

In practice, diversity in morphological traits underlies most taxonomic systems with genitalic characters playing a decisive role in much insect taxonomy [47], reflecting the view that such divergence acts as a prezygotic isolating mechanism [48]. Sauer & Hausdorf [49] support this priority, arguing that copulatory organs should be assigned special value in taxonomy because they are directly involved in speciation. It has also been suggested that genitalia show rapid divergence as a result of selection against hybrids when populations with partial reproductive isolation come into secondary contact [50]. These views have often led genitalic variation to be rated as decisive in considerations of taxonomic status. For example, alpine and Fennoscandian populations of *Xestia lorezi* are considered as subspecies because they lack diagnostic genital characteristics [23], [51]–[52] although they possess differing wing patterns and colouration, differing larval characters [53] and substantial COI divergence (MIN = 2.82%). Conversely, *Xestia rhaetica* and *X. fennica* are recognized as distinct species [13]–[14] because of small genital differences [14], [51], despite their morphological similarity and lack of barcode divergence (MIN = 0%). The arctic-alpine butterflies, *Erebia polaris* and *E. medusa,* provide another example of the subjectivity of taxonomic decisions based solely on morphology. While noting differences in size, wing colour and markings, Warren [54] treated *Erebia polaris* as a subspecies of *Erebia medusa* because of their similar genitalia, but other authors [55]–[56] have accepted them as valid species because of their differing external morphology. These examples not only demonstrate that morphological and genetic data do not always provide coincident signals in relation to species status, but also that there is no invariant rule for prioritizing external versus genitalic morphology in determinations of species status. There is certainly no basis for considering morphological features more “valuable” than genetic data and no reason to assume that species should always be morphologically distinct. *Neofaculta infernella* may represent a case of the latter situation as its Finnish populations include two barcode clusters with deep divergence (MIN = 8.4%), but no obvious morphological differences. If this barcode split is correlated to divergence in a nuclear marker or in ecological traits, recognition of two species should follow [45].

The idea that genitalic differentiation (or the lack of it) can indicate the presence or absence of reproductive isolation is unconvincing. For example, sperm transfer was frequently successful among species in the moth genus *Euxoa* despite their genitalic differences [57]. However, because genitalic morphology evolves rapidly [48], [58] it remains taxonomically informative. Our results further demonstrate a close correspondence between genitalic and barcode divergences. For example, five of six cases of barcode sharing that we detected between Fennoscandian and alpine taxa involved species with little or no divergence in their genital morphology. Conversely, species with clearly different genitalia nearly always possessed substantial barcode divergence.

Based on their review of many case studies, Funk & Omland [59] concluded that the transfer of mitochondrial genomes was relatively common among closely related species. It now seems that their conclusion reflected the fact that species selected for genetic analysis were not chosen randomly - they often represent taxonomically complex situations. Although Funk & Omland’s study thus probably overestimated the incidence of mitochondrial introgression, this complication does occur in a few arctic-alpine taxa. For example, Schmidt [27] found that Nearctic specimens of *Grammia quenseli* possessed five COI clusters with a maximum divergence of 11%. Because there was no evidence for their linkage to morphology, Schmidt & Sperling [60] concluded that hybridization and introgression were widespread in this genus. Our study revealed an apparent case of paraphyly in *Rheumaptera subhastata*. This species included two barcode clusters, one closer to *R. hastata* than to its conspecifics. However, such cases of paraphyly are rare; this is the sole example that we detected among more than 3000 barcoded species of European Lepidoptera. Before speculating further on the origin of this case, studies are needed on nuclear markers to rule out the possibility that *R. subhastata* is actually two species.

The current subjectivity in decisions concerning the taxonomic status of allopatric populations is unsatisfactory. We believe that objectivity can be enhanced by assigning priority to molecular rather than morphological traits. Under this approach, lineages would be flagged for consideration as distinct species if their allopatric populations exceeded a threshold value (e.g. 2%) of sequence divergence in the DNA barcode region. Although no threshold can act as a species diagnostic in all situations, the same criticism applies to any other criterion. On a positive side, the use of a molecular yardstick as an initial screening tool has a primary advantage; it can be applied in a standardized way across all species. Furthermore, analytical models (ABGD [61], GMYC [62]) can provide an objective way to operationalize the flagging process. Many morphological taxonomists would impose a supplemental requirement; allopatric lineages meeting a sequence threshold should also possess diagnostic morphological difference(s) before gaining recognition as distinct species. This view places molecular characters as subservient to morphological characters since the converse situation of recognizing morphologically distinct, but genetically similar populations as separate species is established practice. A compromise solution to delineate allopatric populations might reserve species status for cases where divergence is apparent for two or more independent characters. Under this approach, species status would be granted to lineages which not only exceed the barcode threshold, but that also show correlated differentiation in any ecological or morphological trait, or in an unlinked molecular marker. The latter criterion will play an increasingly important role in aiding the recognition of species in structurally simple groups of eukaryotes [63], just as it is has in bacteria, archaea and fungi.

## Supporting Information

Table S1 List of specimens with sequence and collection data information.
(PDF)

Table S2 Provisional check-list of arctic-alpine and boreo-montane species shared between the Alps and Fennoscandia but not barcoded from both major distribution areas in this study; taxonomic status of several taxa needs revision.
(PDF)

Table S3 Minimum, mean and maximum intraspecific variation in DNA barcodes in study species within and between the study regions. NA refers to North America, AL to Alps and FE to Fennoscandia. Species showing over 2% intraspecific divergence within or between the regions in question are shown highlighted.
(PDF)

Table S4 Minimum, mean and maximum variation in DNA barcodes between allopatric species, which morphologically are considered distinct taxa, but which genetically are closely similar. NA refers to North America, AL to Alps and FE to Fennoscandia. Superscript numbers after the species refer to the areas of occurrence as follows: 1 = Alps, 2 = Fennoscandia, 3 = North America.
(PDF)
