# Supplementary material for: Allopatry as a Gordian Knot for Taxonomists: Patterns of DNA Barcode Divergence in Arctic-Alpine Lepidoptera
Source: PLoS One. 2012 Oct 11;7(10):e47214. doi: 10.1371/journal.pone.0047214 (PMC3469483; doi:10.1371/journal.pone.0047214)
Supplement: Table S1 — List of specimens with sequence and collection data information. (PDF) [file pone.0047214.s001.pdf]

# Table S1. List of specimens with sequence and collection data information.

| Sample ID | Seq. Length | Gen Bank accession # | Family | Subfamily | Species | Author | Collectors | Collection Date | Country | Province | Region | Sector | Exact Site | Lat | Lon | Dissection |
|---|---|---|---|---|---|---|---|---|---|---|---|---|---|---|---|---|
| CNCLEP00025465 | 655 | JX008152 | Tortricidae | Tortricinae | Acleris maccana | Treitschke | JF Landry & L Humble | 02-Jun-2006 | Canada | British Columbia | | Surrey | Green Timbers Nursery & Arboretum | 49,17900085 | -122,8300018 | |
| CNCLEP0002774 | 658 | JX008418 | Tortricidae | Tortricinae | Acleris maccana | Treitschke | JF Landry | 07-Oct-200 | Canad | Quebe | Terrebonne | Ste Agathe | lac Brule | 46,0880012 | -74,2789993 | |
| CNCLEP0002941 | 658 | JX008124 | Tortricidae | Tortricinae | Acleris maccana | Treitschke | D Holder | 24-Aug-200 | Canad | British Columbi | | Bute Inle | Camp Homathc | 50,9150009 | -124,860000 | |
| DNA-ATBI-215 | 658 | JX008260 | Tortricidae | Tortricinae | Acleris maccana | Treitschke | Michael Pogu | 20-May-200 | United State | Tennesse | Haywoo | Poll`s Gap, Rough Fork Tra | BLBT7 | 35,5640983 | -83,1613998 | |
| DNA-ATBI-2154 | 658 | JX008191 | Tortricidae | Tortricinae | Acleris maccana | Treitschke | Adams | 20-May-2005 | United States | Tennessee | Sevier | 4700`, Just below upper tunnel, east side of 441 | BLBT10 | 35,61780167 | -83,41919708 | |
| DNA-ATBI-2155 | 658 | JX008172 | Tortricidae | Tortricinae | Acleris maccana | Treitschke | Adams | 20-May-2005 | United States | Tennessee | Sevier | 4700`, Just below upper tunnel, east side of 441 | BLBT10 | 35,61780167 | -83,41919708 | |
| jflandry1751 | 614 | JX008155 | Tortricidae | Tortricinae | Acleris maccana | Treitschke | L. Scott | 15-Apr-2005 | Canad | Ontari | Ottawa-Carleto | Dunrobin | 122 Dunhaven Driv | 45,4099998 | -76,0579986 | |
| MDH00089 | 658 | JX008276 | Tortricidae | Tortricinae | Acleris maccana | Treitschke | Daniel Handfiel | 24-Apr-2005 | Canad | Quebe | La Presentatio | | Rang 5 Andre Racico | 45,6889991 | -73,0879974 | |
| MDH00194 | 658 | JX008488 | Tortricidae | Tortricinae | Acleris maccana | Treitschke | Daniel Handfiel | 02-Oct-200 | Canad | Quebe | Ste-Christine | | Boise Julien Picard | 45,6510009 | -72,4479980 | |
| MDH00195 | 658 | JX008104 | Tortricidae | Tortricinae | Acleris maccana | Treitschke | Daniel Handfiel | 02-Oct-200 | Canad | Quebe | Ste-Christine | | Boise Julien Picard | 45,6510009 | -72,4479980 | |
| MDH00206 | 658 | JX008205 | Tortricidae | Tortricinae | Acleris maccana | Treitschke | Daniel Handfiel | 22-Apr-2005 | Canad | Quebe | Ste-Christine | | Boise Julien Picard | 45,6510009 | -72,4479980 | |
| MDH00212 | 658 | JX008313 | Tortricidae | Tortricinae | Acleris maccana | Treitschke | Daniel Handfiel | 17-Apr-2005 | Canad | Quebe | Mont-St-Hilaire | | Rue Noiseu | 45,5439987 | -74,25 | |
| MDH00214 | 658 | JX008331 | Tortricidae | Tortricinae | Acleris maccana | Treitschke | Daniel Handfiel | 13-Apr-2005 | Canad | Quebe | La Presentatio | | Rang 5 Andre Racico | 45,6889991 | -73,0879974 | |
| MDH00282 | 575 | JX008200 | Tortricidae | Tortricinae | Acleris maccana | Treitschke | Daniel Handfiel | 19-Apr-2005 | Canad | Quebe | CAN:QC -Mont-St-Hilair | | Chez L.Handfiel | 45,5289993 | -73,1790008 | |
| MDH00282 | 657 | JX008224 | Tortricidae | Tortricinae | Acleris maccana | Treitschke | Daniel Handfiel | 07-May-200 | Canad | Quebe | CAN:QC -Mont-St-Hilair | | 545 boul Laurie | 45,5680007 | -73,1750030 | |
| MM08197 | 658 | HM873813 | Tortricidae | Tortricinae | Acleris maccana | Treitschke | Marko Mutanen, Panu Vaelimaeki | | Finland | | Lapponia kemensis pars occidentalis | | Kolari | 67,27600098 | 23,75300026 | |
| MM0829 | 658 | HM87385 | Tortricidae | Tortricinae | Acleris maccana | Treitschke | Marko Mutane | | Finlan | | Lapponia inarensi | | Inari | 68,78800201 | 27,0990009 | |
| MM08762 | 658 | HM874067 | Tortricidae | Tortricinae | Acleris maccana | Treitschke | Marko Mutanen, Nestori Mutanen, Anttoni Mutane | 02-May-2008 | Finland | | Ostrobottnia borealis pars australis | | Kiiminki | 65,07099915 | 25,72500038 | |
| MM13882 | 658 | HM875780 | Tortricidae | Tortricinae | Acleris maccana | Treitschke | Marko Mutanen | | Finland | | Ostrobottnia borealis pars borealis | | Tornio | 65,89800262 | 24,45499992 | |
| MM17981 | 658 | JX034630 | Tortricidae | Tortricinae | Acleris maccana | Treitschke | Marko Mutanen | | Finland | | Lapponia kemensis pars occidentalis | | Kolari | 67,27610016 | 23,75250053 | |
| NoA-08-097 | 658 | JX008467 | Tortricidae | Tortricinae | Acleris maccana | Treitschke | D.A. Macaulay | 19-Apr-2003 | Canada | Alberta | | | Holmes Crossing Eco Res, 7.3 km SE Ft Assiniboine | 54,3030014 | -114,8119965 | |
| NoA-08-134 | 658 | JX008479 | Tortricidae | Tortricinae | Acleris maccana | Treitschke | | 05-Apr-1996 | Canad | Albert | | | Edmonto | 53,5390014 | -113,460998 | |
| NoA-08-135 | 658 | JX008116 | Tortricidae | Tortricinae | Acleris maccana | Treitschke | | 12-Apr-1999 | Canad | Albert | | | Wagner Natural Are | 53,5680007 | -113,824996 | |
| NoA-08-136 | 658 | JX008370 | Tortricidae | Tortricinae | Acleris maccana | Treitschke | | 27-Apr-2001 | Canad | Albert | | | Gainford | 53,5849990 | -114,787002 | |
| NoA-08-137 | 658 | JX008289 | Tortricidae | Tortricinae | Acleris maccana | Treitschke | D.A. Macaulay | 06-Jun-2002 | Canada | Alberta | | | Colin-Cornwall Lakes Wildland Prov Pk | 59,55699921 | -110,2750015 | |
| NoA-08-138 | 587 | JX008105 | Tortricidae | Tortricinae | Acleris maccana | Treitschke | M. Michaelian et al | 14-Sep-199 | Canad | Saskatchewa | | | nr. Big Rive | 54,0029983 | -107,261001 | |
| TLMF Lep 0284 | 658 | JF860323 | Tortricidae | Tortricinae | Acleris maccana | Treitschke | Huemer P | 02-May-200 | Austria | Vorarlberg | Umg.Dornbir | | Boedele, Fohramoo | 47,4169998 | 9,800000191 | |
| TLMF Lep 0284 | 658 | JF860324 | Tortricidae | Tortricinae | Acleris maccana | Treitschke | Huemer P | 02-May-200 | Austria | Vorarlberg | Umg.Dornbir | | Boedele, Fohramoo | 47,4169998 | 9,800000191 | |
| TLMF Lep 02881 | 658 | JF860341 | Tortricidae | Tortricinae | Acleris maccana | Treitschke | Jaros J. & Spitzer K. | 01-Oct-2002 | Czech Republic | Bohemia | | | Sumava Mt., Mrtvy luh near Volary | 48,86700058 | 13,86699963 | |
| TLMF Lep 02882 | 658 | JF860342 | Tortricidae | Tortricinae | Acleris maccana | Treitschke | Jaros J. & Spitzer K. | 24-Oct-2004 | Czech Republic | Bohemia | | | Novohradske hory Mts., Pohorske raseliniste | 48,59999847 | 14,68299961 | |
| TLMF Lep 0297 | 658 | JF860404 | Tortricidae | Tortricinae | Acleris maccana | Treitschke | Huemer P | 02-May-200 | Austria | Vorarlberg | | | Fohramoos EN | 47,4210014 | 9,80599975 | |
| 07PROBE-00581 | 658 | JX008426 | Tortricidae | Tortricinae | Aethes deutschiana | Zetterstedt | J.deWaard | 16-Jul-2007 | Canada | Manitoba | Churchill | 14 km E Churchill, Golf Balls Intertida | Golfball Beach | 58,76499939 | -93,93199921 | |
| 07PROBE-1006 | 658 | JX008338 | Tortricidae | Tortricinae | Aethes deutschian | Zetterstedt | P.D.N. Heber | 16-Jul-2007 | Canad | Manitob | Churchill | 13 km E Churchill, Eastern Cree | | 58,7550010 | -93,9440002 | |
| 08BBLEP-02400 | 658 | JX008401 | Tortricidae | Tortricinae | Aethes deutschiana | Zetterstedt | J.Straka,J.Cossey | 24-Jul-2008 | Canada | Alberta | Waterton | Waterton Lakes Nat. Park - Rowe Trailhea | Roadside forest/meadow | 49,05699921 | -114,0110016 | |
| 09PROBE-09357 | 658 | HM375613 | Tortricidae | Tortricinae | Aethes deutschiana | Zetterstedt | P.D.N. Hebert | 26-Jul-2009 | Canada | Manitoba | Churchill | 26 km SE Churchill, Twin Lakes burn site | | 58,61999893 | -93,83000183 | |
| 09PROBE-0940 | 658 | HM37565 | Tortricidae | Tortricinae | Aethes deutschian | Zetterstedt | P.D.N. Heber | 27-Jul-200 | Canad | Manitob | Churchill | 23 km E Churchill, Ramsay Cree | | 58,7299995 | -93,7799987 | |
| 09PROBE-0940 | 632 | HM37566 | Tortricidae | Tortricinae | Aethes deutschian | Zetterstedt | P.D.N. Heber | 30-Jul-200 | Canad | Manitob | Churchill | 11 km S Churchill, Goose Cree | Goose Creek Rd. at Rail Lin | 58,6599998 | -94,1699981 | |
| 09PROBE-0944 | 658 | HM37569 | Tortricidae | Tortricinae | Aethes deutschian | Zetterstedt | P.D.N. Heber | 30-Jul-200 | Canad | Manitob | Churchill | 11 km S Churchill, Goose Cree | Goose Creek Rd. at Rail Lin | 58,6599998 | -94,1699981 | |
| 09PROBE-0944 | 658 | HM37569 | Tortricidae | Tortricinae | Aethes deutschian | Zetterstedt | P.D.N. Heber | 30-Jul-200 | Canad | Manitob | Churchill | 11 km S Churchill, Goose Cree | Goose Creek Rd. at Rail Lin | 58,6599998 | -94,1699981 | |
| 09PROBE-0944 | 658 | HM37570 | Tortricidae | Tortricinae | Aethes deutschian | Zetterstedt | P.D.N. Heber | 28-Jul-200 | Canad | Manitob | Churchill | 26 km SE Churchill, Twin Lake | | 58,6300010 | -93,8190002 | |
| 09PROBE-0947 | 658 | HM37573 | Tortricidae | Tortricinae | Aethes deutschian | Zetterstedt | P.D.N. Heber | 28-Jul-200 | Canad | Manitob | Churchill | 23 km E Churchill, Ramsay Cree | | 58,7299995 | -93,7799987 | |
| 09PROBE-0948 | 658 | HM37573 | Tortricidae | Tortricinae | Aethes deutschian | Zetterstedt | P.D.N. Heber | 28-Jul-200 | Canad | Manitob | Churchill | 23 km E Churchill, Ramsay Cree | | 58,7299995 | -93,7799987 | |
| 09PROBE-0948 | 658 | HM37573 | Tortricidae | Tortricinae | Aethes deutschian | Zetterstedt | P.D.N. Heber | 28-Jul-200 | Canad | Manitob | Churchill | 23 km E Churchill, Ramsay Cree | | 58,7299995 | -93,7799987 | |
| 09PROBE-0949 | 658 | HM37574 | Tortricidae | Tortricinae | Aethes deutschian | Zetterstedt | P.D.N. Heber | 28-Jul-200 | Canad | Manitob | Churchill | 23 km E Churchill, Ramsay Cree | | 58,7299995 | -93,7799987 | |
| 09PROBE-0949 | 658 | HM37574 | Tortricidae | Tortricinae | Aethes deutschian | Zetterstedt | P.D.N. Heber | 28-Jul-200 | Canad | Manitob | Churchill | 23 km E Churchill, Ramsay Cree | | 58,7299995 | -93,7799987 | |
| 09PROBE-0957 | 658 | HM37582 | Tortricidae | Tortricinae | Aethes deutschian | Zetterstedt | P.D.N. Heber | 26-Jul-200 | Canad | Manitob | Churchill | 26 km SE Churchill, Twin Lake | | 58,6199989 | -93,7799987 | |
| 09PROBE-09592 | 658 | HM430239 | Tortricidae | Tortricinae | Aethes deutschiana | Zetterstedt | P.D.N. Hebert | 26-Jul-2009 | Canada | Manitoba | Churchill | 26 km SE Churchill, Twin Lakes burn site | | 58,61999893 | -93,83000183 | |
| MM0008 | 658 | HM39636 | Tortricidae | Tortricinae | Aethes deutschian | Zetterstedt | Marko Mutane | | Finlan | | | | Enontekio | 68,9970016 | 20,7439994 | |
| MM0412 | 658 | HM38691 | Tortricidae | Tortricinae | Aethes deutschian | Zetterstedt | Marko Mutane | | Finlan | | | | Enontekio | 68,9970016 | 20,7439994 | |
| MM06320 | 658 | HM873198 | Tortricidae | Tortricinae | Aethes deutschiana | Zetterstedt | Marko Mutanen, Anttoni Mutanen, Nestori Mutane | 28-Jun-2006 | Finland | | Lapponia inarensis | | Utsjoki | 69,85500336 | 27 | |
| NoA-08-144 | 658 | JX008274 | Tortricidae | Tortricinae | Aethes deutschian | Zetterstedt | | 29-Jul-200 | Canad | Albert | | | Kakwa Wildland Prov P | 54,1150016 | -119,938003 | |
| TLMF Lep 02701 | 658 | JF860231 | Tortricidae | Tortricinae | Aethes deutschiana | Zetterstedt | Schmid J. | 16-Jul-2009 | Switzerland | Graubunden | | | Avers-Cresta, Cucalner/Guggermue | 46,5019989 | 9,484999657 | |
| TLMF Lep 02858 | 639 | JX034623 | Tortricidae | Tortricinae | Aethes deutschiana | Zetterstedt | Nel J. | 22-Jul-2001 | France | Provence-Alpes-Cote d'Azu | | | Col Agnel | 44,68899918 | 6,984000206 | 13254JN |
| TLMF Lep 02859 | 608 | JX034651 | Tortricidae | Tortricinae | Aethes deutschiana | Zetterstedt | Nel J. | 04-Jul-2004 | France | Provence-Alpes-Cote d'Azu | | | Col de l`Izoard | 44,80899811 | 6,761000156 | 17256JN |
| TLMF Lep 02860 | 658 | JX034633 | Tortricidae | Tortricinae | Aethes deutschiana | Zetterstedt | Nel J. | 04-Jul-2004 | France | Provence-Alpes-Cote d'Azu | | | Col de l`Izoard | 44,80899811 | 6,761000156 | 17234JN |
| MM10662 | 658 | HM874840 | Incurvariidae | | Alloclemensia mesospilella | Herrich-Schäffer | Marko Mutanen, Panu Vaelimaeki | | Finland | | Lapponia kemensis pars orientalis | | Pelkosenniemi | 67,15399933 | 27,85400009 | |
| MM10668 | 658 | HM874844 | Incurvariidae | | Alloclemensia mesospilella | Herrich-Schäffer | Marko Mutanen, Panu Vaelimaeki | | Finland | | Lapponia kemensis pars orientalis | | Pelkosenniemi | 67,15399933 | 27,85400009 | |
| MM1793 | 658 | JF853993 | Incurvariidae | | Alloclemensia mesospilel | Herrich-Schäffer | Sami Haapal | 02-Jun-200 | Finlan | | Savonia australi | | Lappeenrant | 61,0638999 | 28,7222995 | |
| TLMF Lep 00553 | 658 | HM381395 | Incurvariidae | | Alloclemensia mesospilella | Herrich-Schäffer | Huemer P. | 13-Jul-2009 | Austria | Karnten | | | Petzen N, Umg. Gh. Siebenhuetten | 46,51819992 | 14,77350044 | |
| MM0410 | 658 | HM38689 | Noctuidae | Noctuinae | Anarta melanop | Thunberg | Marko Mutane | | Finlan | | | | Enontekio | 68,9970016 | 20,7439994 | |
| MM0410 | 658 | HM38689 | Noctuidae | Noctuinae | Anarta melanop | Thunberg | Marko Mutane | | Finlan | | | | Enontekio | 68,9970016 | 20,7439994 | |
| MM1458 | 658 | HM87617 | Noctuidae | Noctuinae | Anarta melanop | Thunberg | Marko Mutane | | Finlan | | Lapponia enontekiens | | Enontekio | 69,0630035 | 21,1019992 | |

| | | | | | | | | | | | | | | | |
|---|---|---|---|---|---|---|---|---|---|---|---|---|---|---|---|
| MM17997 | 658 | JF854051 | Noctuidae | Noctuinae | Anarta melanopa | Thunberg | Marko Mutanen, Nestori Mutanen, Anttoni Mutaner | 10-Jul-2010 | Finland | | Lapponia inarensis | | Utsjoki | 69,82800293 | 27 |
| TLMF Lep 0193 | 658 | HQ96835 | Noctuidae | Noctuinae | Anarta melanopa | Thunberg | Aistleitner E | 17-Jul-1994 | Austria | Tyrol | Osttiro | Zentralalpen | Panargenkamm: Weisser Klap | 46,9860000 | 12,2742004 |
| TLMF Lep 0270 | 658 | JF860233 | Noctuidae | Noctuinae | Anarta melanopa | Thunberg | Schmid J. | 10-Apr-2007 | Switzerland | Graubunder | | | Vals, Dachberg | 46,5919990 | 9,10299968 |
| MM0214 | 575 | HM87142 | Tortricidae | Olethreutinae | Ancylis habeler | Huemer & Tarmann | Marko Mutanen, Panu Vaelimae | | Finland | | Savonia australis | | Imatra | 61,10800171 | 28,7989997 |
| MM05357 | 636 | HM872781 | Tortricidae | Olethreutinae | Ancylis habeleri | Huemer & Tarmann | Marko Mutanen | | Finland | Lansi-Suomen Laani | Regio Aboensis | | Turku | 60,44100189 | 22,20199966 |
| MM13831 | 658 | HM875777 | Tortricidae | Olethreutinae | Ancylis habeler | Huemer & Tarmann | Marko Mutanen, Panu Vaelimae | | Finland | | Regio Aboensis | | Dragsfjaerd | 60,0110015 | 22,4979991 |
| MM1448 | 658 | HM87612 | Tortricidae | Olethreutinae | Ancylis habeler | Huemer & Tarmann | Tomi Mutanen | | Finland | | Regio Aboensis | | Salo | 60,2620010 | 23,02499962 |
| MM1448 | 658 | HM87612 | Tortricidae | Olethreutinae | Ancylis habeler | Huemer & Tarmann | Tomi Mutanen | | Finland | | Regio Aboensis | | Salo | 60,2620010 | 23,02499962 |
| MM1448 | 658 | HM87612 | Tortricidae | Olethreutinae | Ancylis habeler | Huemer & Tarmann | Tomi Mutanen | | Finland | | Regio Aboensis | | Salo | 60,2620010 | 23,02499962 |
| MM1448 | 658 | HM87612 | Tortricidae | Olethreutinae | Ancylis habeler | Huemer & Tarmann | Tomi Mutanen | | Finland | | Regio Aboensis | | Salo | 60,2620010 | 23,02499962 |
| MM1729 | 642 | JX034663 | Tortricidae | Olethreutinae | Ancylis habeler | Huemer & Tarmann | Tomi Mutanen | | Finland | | Regio Aboensis | | Salo | 60,340999 | 23,1110000 |
| MM1729 | 658 | JX034607 | Tortricidae | Olethreutinae | Ancylis habeler | Huemer & Tarmann | Tomi Mutanen | | Finland | | Regio Aboensis | | Salo | 60,340999 | 23,1110000 |
| MM1729 | 658 | JX034683 | Tortricidae | Olethreutinae | Ancylis habeler | Huemer & Tarmann | Tomi Mutanen | | Finland | | Regio Aboensis | | Salo | 60,340999 | 23,1110000 |
| TLMF Lep 0278 | 658 | JF860289 | Tortricidae | Olethreutinae | Ancylis habeler | Huemer & Tarmann | Deutsch H. | 18-Jun-2002 | Austria | Tyrol | Osttiro | | Dorfermaehder/ Praegraten NW | 47,0250015 | 12,3710002 |
| AOC Lep 0036 | 658 | JX034640 | Noctuidae | Amphipyrinae | Apamea maillard | Geyer | J.J. Guerrero | 03-Aug-2010 | Spain | Lleida | Lerida, Cataluna | | Valle de Aran | 42,6912002 | 0,90175998 |
| BC ZSM Lep 48525 | 658 | JX034692 | Noctuidae | Amphipyrinae | Apamea maillardi | Geyer | J. Halwax | 05-Aug-2008 | France | Provence-Alpes-Cote d'Azur | | | nr Embrun, Les Orres, Torrent d. Vachers | 44,51670074 | 6,550000191 |
| TLMF Lep 00318 | 658 | HM425813 | Noctuidae | Amphipyrinae | Apamea maillardi | Geyer | Huemer P. | 20-Jul-2009 | Italy | | Belluno | | Passo di Valparola E - Passo Falzarego | 46,5223999 | 12,00699997 |
| TLMF Lep 00410 | 658 | HM425893 | Noctuidae | Amphipyrinae | Apamea maillardi | Geyer | Huemer P. | 23-Jul-2009 | Italy | | Cuneo | | Colle Valcavera NE/ Demonte NW | 44,38439941 | 7,106389999 |
| TLMF Lep 0246 | 658 | JF860009 | Noctuidae | Amphipyrinae | Apamea maillard | Geyer | Huemer P | 19-Jul-2010 | Italy | South Tyro | | | Ritten/ Obergruenwal | 46,5970001 | 11,4390001 |
| TLMF Lep 0270 | 658 | JF860234 | Noctuidae | Amphipyrinae | Apamea maillard | Geyer | Schmid J. | 22-Jun-2000 | Switzerland | Graubunder | | | Zervreila/ Vals | 46,5779991 | 9,12300014 |
| TLMF Lep 05538 | 658 | JX034596 | Noctuidae | Amphipyrinae | Apamea maillardi | Geyer | Huemer P. & Tarmann G. M. | 28-Jul-2011 | Macedonia | | | | Mavrovo NP, Korab, Korabska jezero, Kobilino pole | 41,77799988 | 20,58200073 |
| MM09205 | 658 | HM874166 | Noctuidae | Amphipyrinae | Apamea schildei | Staudinger | Petri Hirvonen | 30-Jul-2008 | Finland | | Lapponia kemensis pars occidentalis | | Muonio | 67,91200256 | 23,66399956 |
| MM1107 | 658 | HM87498 | Noctuidae | Amphipyrinae | Apamea schilde | Staudinger | Marko Mutanen | 19-Jul-2005 | Finland | | Regio kuusamoensis | | Salla | 66,6819992 | 28,5839996 |
| MM1107 | 658 | HM87498 | Noctuidae | Amphipyrinae | Apamea schilde | Staudinger | Marko Mutanen | 17-Jul-2005 | Finland | | Regio kuusamoensis | | Salla | 66,6819992 | 28,5839996 |
| MM1747 | 658 | JF853860 | Noctuidae | Amphipyrinae | Apamea schilde | Staudinger | Juhani Itaemies | 23-Aug-2008 | Finland | | Kuusamo | | Salla | 67,6589965 | 29,3570003 |
| MM1875 | 658 | JF854601 | Noctuidae | Amphipyrinae | Apamea schilde | Staudinger | Reima Leinonen | 16-Jul-1999 | Finland | | | | | | |
| MM1875 | 658 | JF854602 | Noctuidae | Amphipyrinae | Apamea schilde | Staudinger | Reima Leinonen | 17-Jul-2001 | Finland | | Karelia borealis | | Lieksa | 63,7400016 | 24,2460002 |
| MM1875 | 658 | JF854603 | Noctuidae | Amphipyrinae | Apamea schilde | Staudinger | Reima Leinonen | 18-Jul-2001 | Finland | | | | | | |
| MM1875 | 658 | JF854604 | Noctuidae | Amphipyrinae | Apamea schilde | Staudinger | Reima Leinonen | 18-Jul-2001 | Finland | | Ostrobottnia kajanensi | | Suomussalm | 65,0110015 | 24,0520000 |
| MM1875 | 658 | JX034632 | Noctuidae | Amphipyrinae | Apamea schilde | Staudinger | Reima Leinonen | 19-Jul-2001 | Finland | | Regio kuusamoensis | | Kuusamo | 65,6299972 | 24,0030002 |
| MM1875 | 658 | JF854605 | Noctuidae | Amphipyrinae | Apamea schilde | Staudinger | Reima Leinonen | 16-Jul-1999 | Finland | | | | | | |
| MM1875 | 658 | JF854606 | Noctuidae | Amphipyrinae | Apamea schilde | Staudinger | Reima Leinonen | 18-Jul-2001 | Finland | | Ostrobottnia kajanensi | | Kuhmo | 63,9080009 | 30,301000 |
| AOC Lep 0037 | 658 | JX034670 | Noctuidae | Amphipyrinae | Apamea zeta | Treitschke | J.J. Guerrero | 03-Aug-2010 | Spain | Lleida | Lerida, Cataluna | | Valle de Aran | 42,7400016 | 0,75713002 |
| CNCNoctuoidea1038 | 658 | JX008436 | Noctuidae | Amphipyrinae | Apamea zeta | Treitschke | | 27-Jul-1990 | Canada | Quebec | | | Post de la Baleine | | |
| CNCNoctuoidea1038 | 658 | JX008483 | Noctuidae | Amphipyrinae | Apamea zeta | Treitschke | | 03-Aug-1990 | Canada | Quebec | | | Post de la Baleine | | |
| CNCNoctuoidea13892 | 638 | JX008210 | Noctuidae | Amphipyrinae | Apamea zeta | Treitschke | S.A. Johnson | 17-Jul-2007 | United States | Colorado | | | Pikes Peak East face, El Paso County | | |
| CNCNoctuoidea13893 | 658 | JX008265 | Noctuidae | Amphipyrinae | Apamea zeta | Treitschke | S.A. Johnson | 17-Jul-2007 | United States | Colorado | | | Pikes Peak East face, El Paso County | | |
| CNCNoctuoideaE01 | 527 | JX034668 | Noctuidae | Amphipyrinae | Apamea zeta | Treitschke | M. Fibiger | 08-Aug-1998 | France | | | | Restiford | | |
| CNCNoctuoideaE01 | 517 | JX034614 | Noctuidae | Amphipyrinae | Apamea zeta | Treitschke | S. Beschkov | 10-Aug-1991 | Bulgaria | | | | Mousala | | |
| CNCNoctuoideaE01 | 597 | JX034591 | Noctuidae | Amphipyrinae | Apamea zeta | Treitschke | G Stengelmai | 25-Jul-1994 | Romania | | | | Bileasate | | |
| CNCNoctuoideaE01 | 658 | JX034626 | Noctuidae | Amphipyrinae | Apamea zeta | Treitschke | F. Scheper | 04-Aug-1985 | Austria | | | | Grossglockner Hochte | | |
| CNCNoctuoideaE02 | 658 | JX034688 | Noctuidae | Amphipyrinae | Apamea zeta | Treitschke | A. Zilli | 10-Jul-2003 | Italy | Abruzzi | | | Gran Sasso | | |
| CNCNoctuoideaE02 | 557 | JX034679 | Noctuidae | Amphipyrinae | Apamea zeta | Treitschke | P.Provera | 27-Aug-1997 | Switzerland | | | | Graubundenpreda | | |
| TLMF Lep 0024 | 632 | HM42575 | Noctuidae | Amphipyrinae | Apamea zeta | Treitschke | Huemer P | 13-Jul-2009 | Austria | Karnten | | | Petzen N, Obere Krischa | 46,5060997 | 14,75699997 |
| TLMF Lep 00329 | 658 | HM425822 | Noctuidae | Amphipyrinae | Apamea zeta | Treitschke | Huemer P. | 20-Jul-2009 | Italy | | Belluno | | Passo di Valparola E - Passo Falzarego | 46,5223999 | 12,00699997 |
| TLMF Lep 00411 | 658 | HM425894 | Noctuidae | Amphipyrinae | Apamea zeta | Treitschke | Huemer P. | 23-Jul-2009 | Italy | | Cuneo | | Colle Valcavera NE/ Demonte NW | 44,38439941 | 7,106389999 |
| TLMF Lep 00463 | 658 | HM425939 | Noctuidae | Amphipyrinae | Apamea zeta | Treitschke | Huemer P. | 25-Jul-2009 | France | Provence-Alpes-Cote d'Azur | | | Col de la Moutiere SE/ W St. Dalmas le Selvage | 44,30670166 | 6,809999943 |
| TLMF Lep 01953 | 658 | HQ968365 | Noctuidae | Amphipyrinae | Apamea zeta | Treitschke | Huemer P. | 14-Jul-2010 | Italy | Abruzzi | L`Aquila | | NP Gran Sasso, Campo Imperatore, ex Miniera di Lignite | 42,42670059 | 13,71329975 |
| TLMF Lep 0270 | 483 | JX034691 | Noctuidae | Amphipyrinae | Apamea zeta | Treitschke | Schmid J. | 22-Aug-2001 | Switzerland | Graubunder | | | Nagens/ Laax | 46,8650016 | 9,232999802 |
| TLMF Lep 05565 | 658 | JX034693 | Noctuidae | Amphipyrinae | Apamea zeta | Treitschke | Huemer P. & Tarmann G. M. | 28-Jul-2011 | Macedonia | | | | Mavrovo NP, Korab, eastern ridge | 41,7859993 | 20,57900047 |
| TLMF Lep 05566 | 658 | JX034646 | Noctuidae | Amphipyrinae | Apamea zeta | Treitschke | Huemer P. & Tarmann G. M. | 28-Jul-2011 | Macedonia | | | | Mavrovo NP, Korab, eastern ridge | 41,7859993 | 20,57900047 |
| MM1764 | 658 | JX034675 | Oecophoridae | Oecophorinae | Aplota nigricans | Zeller | Kaitila, Salin, Palen, Ratinen, Vinn | | Finland | | Nylandia | | Inkoo | 59,9269981 | 23,9594993 |
| MM1764 | 658 | JX034641 | Oecophoridae | Oecophorinae | Aplota nigricans | Zeller | Kaitila, Salin, Palen, Ratinen, Vinn | | Finland | | Nylandia | | Inkoo | 59,9269981 | 23,9594993 |
| MM1765 | 658 | JX034660 | Oecophoridae | Oecophorinae | Aplota nigricans | Zeller | Kaitila, Salin, Palen, Ratinen, Vinn | | Finland | | Nylandia | | Inkoo | 59,9269981 | 23,9594993 |
| TLMF Lep 0174 | 658 | HQ96895 | Oecophoridae | Oecophorinae | Aplota nigricans | Zeller | Huemer P | 30-Jun-2010 | Italy | South Tyro | Etschta | | Montiggl/ Kleiner Pric | 46,4282989 | 11,3000001 |
| TLMF Lep 0211 | 658 | JF859701 | Oecophoridae | Oecophorinae | Aplota nigricans | Zeller | Huemer P | 04-Jun-2010 | Italy | South Tyro | Etschta | | Montiggl/ Kleiner Pric | 46,4280014 | 11,3000001 |
| MM03442 | 658 | HQ57030 | Tortricidae | Olethreutinae | Argyroploce arbutella | Linnaeus | Marko Mutanen | | Finland | Lappi | Lapponia enontekiensi | | Enontekioe | 68,9970016 | 20,7439994 |
| MM12391 | 658 | HM87526 | Tortricidae | Olethreutinae | Argyroploce arbutella | Linnaeus | Marko Mutanen, Panu Vaelimae | | Finland | | Savonia australis | | Imatra | 61,10800171 | 28,7989997 |
| MM14517 | 658 | HM876149 | Tortricidae | Olethreutinae | Argyroploce arbutella | Linnaeus | Marko Mutanen | 10-Jul-2009 | Finland | | Ostrobottnia borealis pars australis | | Hailuoto | 65 | 25,00699997 |
| TLMF Lep 0168 | 658 | HQ96888 | Tortricidae | Olethreutinae | Argyroploce arbutella | Linnaeus | Huemer P | 16-Jul-2010 | Italy | Lazio | Rieti | | Monte Terminillo | 42,4832992 | 13 |
| TLMF Lep 0168 | 658 | HQ96888 | Tortricidae | Olethreutinae | Argyroploce arbutella | Linnaeus | Huemer P | 16-Jul-2010 | Italy | Lazio | Rieti | | Monte Terminillo | 42,4832992 | 13 |
| MM0008 | 658 | HM39636 | Tortricidae | Olethreutinae | Argyroploce noricana | Herrich-Schäffer | Marko Mutanen | | Finland | | | | Enontekioe | 68,9970016 | 20,7439994 |
| MM0412 | 407 | JX034576 | Tortricidae | Olethreutinae | Argyroploce noricana | Herrich-Schäffer | Marko Mutanen | | Finland | | | | Enontekioe | 68,9970016 | 20,7439994 |
| MM0704 | 658 | JX034609 | Tortricidae | Olethreutinae | Argyroploce noricana | Herrich-Schäffer | | | Finland | Lappi | | | | | |
| TLMF Lep 0060 | 658 | HM38144 | Tortricidae | Olethreutinae | Argyroploce noricana | Herrich-Schäffer | Huemer P | 20-Jul-2009 | Italy | | Belluno | | Passo di Valparola E | 46,5255012 | 11,9997997 |
| TLMF Lep 0060 | 658 | HM381447 | Tortricidae | Olethreutinae | Argyroploce noricana | Herrich-Schäffer | Huemer P | 20-Jul-2009 | Italy | | Belluno | | Passo di Valparola E | 46,5255012 | 11,9997997 |
| TLMF Lep 00756 | 658 | HM426110 | Tortricidae | Olethreutinae | Argyroploce noricana | Herrich-Schäffer | Huemer P. | 29-Jul-2008 | Austria | Vorarlberg | | | Umg. Goeppinger Huette, Gamsboden | 47,21749878 | 10,03470039 |
| TLMF Lep 0105 | 658 | HM38161 | Tortricidae | Olethreutinae | Argyroploce noricana | Herrich-Schäffer | Huemer P | 08-Jul-2007 | Italy | South Tyro | | | Schlern, Petz, Kalkschut | 46,5120010 | 11,57499981 |
| TLMF Lep 0270 | 620 | JX034672 | Tortricidae | Olethreutinae | Argyroploce noricana | Herrich-Schäffer | Schmid J. | 26-Jul-2008 | Switzerland | Graubunder | | | Albula Hospiz | 46,5820007 | 9,83699989 |
| MM00696 | 658 | HM871137 | Tortricidae | Olethreutinae | Argyroploce roseomaculana | Herrich-Schäffer | Tomi Mutanen | 09-Jul-2006 | Finland | | Ostrobottnia borealis pars borealis | | Ranua | 65,88200378 | 26,34199905 |

| | | | | | | | | | | | | | | | |
|---|---|---|---|---|---|---|---|---|---|---|---|---|---|---|---|
| MM15688 | 658 | HM876474 | Tortricidae | Olethreutinae | Argyroploce roseomaculana | Herrich-Schäffer | Meelika Mutanen, Atsalea Mutanen, Sylvia Mutanen | | Finland | | Ostrobottnia borealis pars australis | | Kiiminki | 65,07099915 | 25,72500038 |
| MM15689 | 658 | HM876475 | Tortricidae | Olethreutinae | Argyroploce roseomaculana | Herrich-Schäffer | Meelika Mutanen, Atsalea Mutanen, Sylvia Mutanen | | Finland | | Ostrobottnia borealis pars australis | | Kiiminki | 65,07099915 | 25,72500038 |
| MM15690 | 658 | HM876476 | Tortricidae | Olethreutinae | Argyroploce roseomaculana | Herrich-Schäffer | Tomi Mutanen | 04-Jul-2003 | Finland | | Lapponia enontekiensis | | Enontekioe | 68,98699951 | 20,49500084 |
| TLMF Lep 02838 | 639 | JF860320 | Tortricidae | Olethreutinae | Argyroploce roseomaculana | Herrich-Schäffer | Huemer P | 10-Jun-2006 | Italy | South Tyrol | | | Bad Ratzes S/ Seis am Schlern | 46,52998878 | 11,58600044 |
| TLMF Lep 02883 | 658 | JF860343 | Tortricidae | Olethreutinae | Argyroploce roseomaculana | Herrich-Schäffer | Jaros J. & Spitzer K. | 26-Apr-2007 | Czech Republic | Bohemia | | | Chroboly env., Miletinky | 48,91699982 | 14,08300018 |
| TLMF Lep 02884 | 658 | JF860344 | Tortricidae | Olethreutinae | Argyroploce roseomaculana | Herrich-Schäffer | Jaros J. & Spitzer K. | 26-Apr-2007 | Czech Republic | Bohemia | | | Chroboly env., Miletinky | 48,91699982 | 14,08300018 |
| TLMF Lep 02885 | 658 | JF860345 | Tortricidae | Olethreutinae | Argyroploce roseomaculana | Herrich-Schäffer | Jaros J. & Spitzer K. | 26-Apr-2007 | Czech Republic | Bohemia | | | Chroboly env., Miletinky | 48,91699982 | 14,08300018 |
| BC ZSM Lep 21060 | 658 | HQ601063 | Geometridae | Ennominae | Arichanna melanaria | Linnaeus | Karl Ambi | 03-Jul-2008 | Germany | Bavaria | Upper Bavaria | | Schlagenhofen a. Woerthsee | 48,04309845 | 11,1736002 |
| BC ZSM Lep 24045 | 632 | GU654869 | Geometridae | Ennominae | Arichanna melanaria | Linnaeus | A. Haslberger | 12-Jun-2000 | Germany | Bavaria | Oberbayern | Traunstein | Schoenramer Filz | 47,89530182 | 12,84729958 |
| MM01351 | 658 | HM386648 | Geometridae | Ennominae | Arichanna melanaria | Linnaeus | Marko Mutanen, Panu Vaelimaek | | Finland | | | | Imatra | 61,10800171 | 28,79899979 |
| MM01352 | 658 | HM386649 | Geometridae | Ennominae | Arichanna melanaria | Linnaeus | Marko Mutanen, Panu Vaelimaek | | Finland | | | | Imatra | 61,10800171 | 28,79899979 |
| MM08115 | 658 | HM873784 | Geometridae | Ennominae | Arichanna melanaria | Linnaeus | Marko Mutanen, Panu Vaelimaeki | | Finland | | Lapponia kemensis pars occidentalis | | Kolari | 67,27600098 | 23,75300026 |
| TLMF Lep 02877 | 658 | JF860339 | Geometridae | Ennominae | Arichanna melanaria | Linnaeus | Huemer P | 21-Jun-1998 | Austria | Vorarlberg | Umg.Langenegg | | Langenegg-Leiten, Fohren | 47,46699905 | 9,883000374 |
| MM03341 | 658 | HM871928 | Lycaenidae | Polyommatinae | Aricia nicias | Meigen | Marko Mutanen, Nestori Mutanen, Anttoni Mutanen | 14-Jul-2006 | Finland | Oulun laani | Ostrobottnia borealis pars australis | | Kiiminki | 65,07099915 | 25,72500038 |
| MM06112 | 658 | HM873047 | Lycaenidae | Polyommatinae | Aricia nicias | Meigen | Panu Vaelimaeki | 19-Jul-2009 | Finland | | Ostrobottnia borealis pars borealis | | Tornio | 65,84899902 | 24,1970005 |
| MM14661 | 658 | HM876207 | Lycaenidae | Polyommatinae | Aricia nicias | Meigen | Marko Mutanen | 30-Jul-2009 | Finland | | Ostrobottnia kajanensis | | Ristijaervi | 64,4260025 | 28,18300056 |
| TLMF Lep 01819 | 658 | HQ968283 | Lycaenidae | Polyommatinae | Aricia nicias | Meigen | Mayr T. | 04-Jul-2009 | Switzerland | Graubunden | | | Val Bever, Alp Suvretta | 46,54999924 | 9,783329964 |
| TLMF Lep 01820 | 658 | HQ968284 | Lycaenidae | Polyommatinae | Aricia nicias | Meigen | Mayr T. | 01-Aug-2009 | Switzerland | Graubunden | | | Val Bever, Alp Suvretta | 46,54999924 | 9,783329964 |
| TLMF Lep 01821 | 658 | HQ968285 | Lycaenidae | Polyommatinae | Aricia nicias | Meigen | Mayr T. | 23-Aug-2009 | Switzerland | Graubunden | | | Val Bever, Alp Suvretta | 46,54999924 | 9,783329964 |
| TLMF Lep 02751 | 658 | JF860269 | Lycaenidae | Polyommatinae | Aricia nicias | Meigen | Schmid J. | 18-Aug-2009 | Switzerland | Graubunden | | | V. Laschadura/ Zernez | 46,70500183 | 10,15499973 |
| MM15620 | 658 | HQ570408 | Gelechiidae | Gelechiinae | Aristotelia heliacella | Herrich-Schäffer | Marko Mutanen | | Finland | | Lapponia enontekiensis | | Enontekioe | 68,99700165 | 20,74399948 |
| MM15621 | 658 | HQ570409 | Gelechiidae | Gelechiinae | Aristotelia heliacella | Herrich-Schäffer | Marko Mutanen | | Finland | | Lapponia enontekiensis | | Enontekioe | 68,99700165 | 20,74399948 |
| MM18210 | 658 | JX034665 | Gelechiidae | Gelechiinae | Aristotelia heliacella | Herrich-Schäffer | Marko Mutanen | | Finland | | Lapponia enontekiensis | | Enontekioe | 68,99659729 | 20,74370003 |
| TLMF Lep 00613 | 658 | HM425989 | Gelechiidae | Gelechiinae | Aristotelia heliacella | Herrich-Schäffer | Tarmann G. M. | 22-Jul-2009 | Italy | | Belluno | | Passo di Valparola SW, Mt. Castello | 46,52389908 | 11,97669983 |
| TLMF Lep 00614 | 614 | HM425990 | Gelechiidae | Gelechiinae | Aristotelia heliacella | Herrich-Schäffer | Tarmann G. M. | 22-Jul-2009 | Italy | | Belluno | | Passo di Valparola SW, Mt. Castello | 46,52389908 | 11,97669983 |
| TLMF Lep 02707 | 658 | JF860235 | Gelechiidae | Gelechiinae | Aristotelia heliacella | Herrich-Schäffer | Schmid J. | 26-Jul-2008 | Switzerland | Graubunden | | | Albula Hospiz | 46,58200073 | 9,836999893 |
| 04HBL003062 | 658 | GU096671 | Nymphalidae | Heliconiinae | Boloria eunomia | Esper | P.D.N. Hebert | 04-Aug-2004 | Canada | Manitoba | Churchill | 22 km E Churchill, CNSC | Churchill Northern Studies Centre | 58,72999954 | -93,81999969 |
| 04HBL003063 | 658 | GU096676 | Nymphalidae | Heliconiinae | Boloria eunomia | Esper | P.D.N. Hebert | 04-Aug-2004 | Canada | Manitoba | Churchill | 22 km E Churchill, CNSC | Churchill Northern Studies Centre | 58,72999954 | -93,81999969 |
| 04HBL003064 | 658 | GU096675 | Nymphalidae | Heliconiinae | Boloria eunomia | Esper | P.D.N. Hebert | 04-Aug-2004 | Canada | Manitoba | Churchill | 22 km E Churchill, CNSC | Churchill Northern Studies Centre | 58,72999954 | -93,81999969 |
| 04HBL003065 | 658 | GU096674 | Nymphalidae | Heliconiinae | Boloria eunomia | Esper | P.D.N. Hebert | 04-Aug-2004 | Canada | Manitoba | Churchill | 22 km E Churchill, CNSC | Churchill Northern Studies Centre | 58,72999954 | -93,81999969 |
| 04HBL003067 | 658 | GU096679 | Nymphalidae | Heliconiinae | Boloria eunomia | Esper | P.D.N. Hebert | 04-Aug-2004 | Canada | Manitoba | Churchill | 22 km E Churchill, CNSC | Churchill Northern Studies Centre | 58,72999954 | -93,81999969 |
| 04HBL003068 | 658 | GU096678 | Nymphalidae | Heliconiinae | Boloria eunomia | Esper | P.D.N. Hebert | 04-Aug-2004 | Canada | Manitoba | Churchill | 22 km E Churchill, CNSC | Churchill Northern Studies Centre | 58,72999954 | -93,81999969 |
| 04HBL003069 | 658 | GU096677 | Nymphalidae | Heliconiinae | Boloria eunomia | Esper | P.D.N. Hebert | 04-Aug-2004 | Canada | Manitoba | Churchill | 22 km E Churchill, CNSC | Churchill Northern Studies Centre | 58,72999954 | -93,81999969 |
| 04HBL003070 | 658 | GU096673 | Nymphalidae | Heliconiinae | Boloria eunomia | Esper | P.D.N. Hebert | 04-Aug-2004 | Canada | Manitoba | Churchill | 22 km E Churchill, CNSC | Churchill Northern Studies Centre | 58,72999954 | -93,81999969 |
| 04HBL003071 | 658 | GU096672 | Nymphalidae | Heliconiinae | Boloria eunomia | Esper | P.D.N. Hebert | 04-Aug-2004 | Canada | Manitoba | Churchill | 22 km E Churchill, CNSC | Churchill Northern Studies Centre | 58,72999954 | -93,81999969 |
| 04HBL003072 | 658 | GU096681 | Nymphalidae | Heliconiinae | Boloria eunomia | Esper | P.D.N. Hebert | 04-Aug-2004 | Canada | Manitoba | Churchill | 22 km E Churchill, CNSC | Churchill Northern Studies Centre | 58,72999954 | -93,81999969 |
| 04HBL003073 | 658 | GU096680 | Nymphalidae | Heliconiinae | Boloria eunomia | Esper | P.D.N. Hebert | 04-Aug-2004 | Canada | Manitoba | Churchill | 22 km E Churchill, CNSC | Churchill Northern Studies Centre | 58,72999954 | -93,81999969 |
| 07PROBE-00097 | 658 | JX008469 | Nymphalidae | Heliconiinae | Boloria eunomia | Esper | P.D.N. Hebert | 14-Jul-2007 | Canada | Manitoba | Churchill | 4 km SE Churchill, Dene Village | | 58,73500061 | -94,1190033 |
| 07PROBE-00099 | 658 | JX008332 | Nymphalidae | Heliconiinae | Boloria eunomia | Esper | P.D.N. Hebert | 14-Jul-2007 | Canada | Manitoba | Churchill | 4 km SE Churchill, Dene Village | | 58,73500061 | -94,1190033 |
| 07PROBE-00155 | 658 | JX008319 | Nymphalidae | Heliconiinae | Boloria eunomia | Esper | P.D.N. Hebert | 15-Jul-2007 | Canada | Manitoba | Churchill | 26 km SE Churchill, Twin Lakes burn site | Burn site | 58,61700058 | -93,81199646 |
| 07PROBE-00416 | 658 | JX008157 | Nymphalidae | Heliconiinae | Boloria eunomia | Esper | J.deWaard | 16-Jul-2007 | Canada | Manitoba | Churchill | 16 km E Churchill, Bird Cove, Rock Bluff C | Tundra flat nr. rock bluff C | 58,76499939 | -93,86499786 |
| 07PROBE-00502 | 658 | JX008222 | Nymphalidae | Heliconiinae | Boloria eunomia | Esper | J.deWaard | 13-Jul-2007 | Canada | Manitoba | Churchill | Town of Churchill | field S of Churchill train station | 58,76699829 | -94,1760025 |
| 07PROBE-00599 | 658 | JX008308 | Nymphalidae | Heliconiinae | Boloria eunomia | Esper | P.D.N. Hebert | 15-Jul-2007 | Canada | Manitoba | Churchill | 26 km SE Churchill, Twin Lake | | 58,63199997 | -93,81900024 |
| 07PROBE-03878 | 621 | JX008314 | Nymphalidae | Heliconiinae | Boloria eunomia | Esper | P.D.N. Hebert | 14-Jul-2007 | Canada | Manitoba | Churchill | 4 km SE Churchill, Dene Village | | 58,73500061 | -94,1190033 |
| 07PROBE-03883 | 658 | JX008408 | Nymphalidae | Heliconiinae | Boloria eunomia | Esper | P.D.N. Hebert | 14-Jul-2007 | Canada | Manitoba | Churchill | 4 km SE Churchill, Dene Village | | 58,73500061 | -94,1190033 |
| 07PROBE-03887 | 658 | JX008133 | Nymphalidae | Heliconiinae | Boloria eunomia | Esper | P.D.N. Hebert | 17-Jul-2007 | Canada | Manitoba | Churchill | 26 km SE Churchill, Twin Lakes fen | | 58,63199997 | -93,78800201 |
| 07PROBE-03892 | 658 | JX008491 | Nymphalidae | Heliconiinae | Boloria eunomia | Esper | P.D.N. Hebert | 17-Jul-2007 | Canada | Manitoba | Churchill | 26 km SE Churchill, Twin Lakes fen | | 58,63199997 | -93,78800201 |
| 07PROBE-03922 | 658 | JX008357 | Nymphalidae | Heliconiinae | Boloria eunomia | Esper | P.D.N. Hebert | 18-Jul-2007 | Canada | Manitoba | Churchill | 16 km E Churchill, Bird Cove | Tundra Pond Area | 58,76800156 | -93,85800171 |
| 07PROBE-04437 | 658 | JX008345 | Nymphalidae | Heliconiinae | Boloria eunomia | Esper | C.Hastings | 25-Jul-2007 | Canada | Manitoba | Churchill | 4 km SE Churchill, Dene Village | | 58,73300171 | -94,11499786 |
| 07PROBE-10046 | 658 | JX008402 | Nymphalidae | Heliconiinae | Boloria eunomia | Esper | P.D.N. Hebert | 19-Jul-2007 | Canada | Manitoba | Churchill | 22 km E Churchill, CNSC | | 58,73799896 | -93,81900024 |
| 07PROBE-10054 | 658 | JX008110 | Nymphalidae | Heliconiinae | Boloria eunomia | Esper | P.D.N. Hebert | 16-Jul-2007 | Canada | Manitoba | Churchill | 13 km E Churchill, Eastern Creek | | 58,75500107 | -93,94400024 |
| 07PROBE-10055 | 658 | JX008452 | Nymphalidae | Heliconiinae | Boloria eunomia | Esper | P.D.N. Hebert | 16-Jul-2007 | Canada | Manitoba | Churchill | 13 km E Churchill, Eastern Creek | | 58,75500107 | -93,94400024 |
| 07PROBE-10056 | 658 | JX008227 | Nymphalidae | Heliconiinae | Boloria eunomia | Esper | P.D.N. Hebert | 16-Jul-2007 | Canada | Manitoba | Churchill | 13 km E Churchill, Eastern Creek | | 58,75500107 | -93,94400024 |
| 07PROBE-10057 | 658 | JX008231 | Nymphalidae | Heliconiinae | Boloria eunomia | Esper | P.D.N. Hebert | 16-Jul-2007 | Canada | Manitoba | Churchill | 13 km E Churchill, Eastern Creek | | 58,75500107 | -93,94400024 |
| 07PROBE-10113 | 658 | JX008343 | Nymphalidae | Heliconiinae | Boloria eunomia | Esper | P.D.N. Hebert | 18-Jul-2007 | Canada | Manitoba | Churchill | 16 km E Churchill, tundra pond | | 58,75500107 | -93,91500092 |
| 07PROBE-10877 | 658 | JX008444 | Nymphalidae | Heliconiinae | Boloria eunomia | Esper | P.D.N. Hebert | 24-Jul-2007 | Canada | Manitoba | Churchill | 26 km SE Churchill, Twin Lake | | 58,63000107 | -93,81900024 |
| 08BBLEP-02677 | 658 | JX008102 | Nymphalidae | Heliconiinae | Boloria eunomia | Esper | J.Straka,J.Cossey | 02-Aug-2008 | Canada | Alberta | Banff | Banff Nat. Park - Sunshine Meadows | Alpine meadow | 51,06100082 | -115,7839966 |
| 09PROBE-09569 | 658 | HM375820 | Nymphalidae | Heliconiinae | Boloria eunomia | Esper | P.D.N. Hebert | 24-Jul-2009 | Canada | Manitoba | Churchill | 26 km SE Churchill, Twin Lakes burn site | | 58,61999893 | -93,83000183 |
| 09PROBE-09590 | 658 | HM430237 | Nymphalidae | Heliconiinae | Boloria eunomia | Esper | P.D.N. Hebert | 26-Jul-2009 | Canada | Manitoba | Churchill | 26 km SE Churchill, Twin Lakes burn site | | 58,61999893 | -93,83000183 |
| BC ZSM Lep 21543 | 658 | JF415682 | Nymphalidae | Heliconiinae | Boloria eunomia | Esper | P.Schwarzbauer | 03-Jun-2005 | Germany | Bavaria | Oberbayern | | Koenigsdorf, Weitfilz | 47,81669998 | 11,44999981 |
| BC ZSM Lep 25470 | 658 | GU655024 | Nymphalidae | Heliconiinae | Boloria eunomia | Esper | Wolfgang Schacht | 14-Jun-2009 | Germany | Bavaria | Oberbayern | Landsberg a.Lech | Diessen am Ammersee, Vilgertshofener Fors | 47,94649887 | 10,91539955 |
| BC ZSM Lep 32171 | 658 | JF415681 | Nymphalidae | Heliconiinae | Boloria eunomia | Esper | M. Seizmair | 21-May-2009 | Germany | Bavaria | Oberbayern | Bad Toelz-Wolfratshausen | Zellwies | 47,80450058 | 11,46360016 |
| BC ZSM Lep 53120 | 658 | JX034677 | Nymphalidae | Heliconiinae | Boloria eunomia | Esper | Ernst Jung | 10-Jun-2004 | Germany | Bavaria | Schwaben | Ostallgaeu | Lamerdingen, Kleinkitzighofe | 48,07720184 | 10,76959991 |

| | | | | | | | | | | | | | | | | |
|---|---|---|---|---|---|---|---|---|---|---|---|---|---|---|---|---|
| BC ZSM Lep 5312 | 658 | JX034619 | Nymphalidae | Heliconiinae | Boloria eunomia | Esper | Ernst Jung | 10-Jun-2004 | Germany | Bavaria | Schwaben | Ostallgaeu | Lamerdingen, Kleinkitzighofe | 48,0772018 | 10,76959991 | |
| MM13982 | 658 | HM875823 | Nymphalidae | Heliconiinae | Boloria eunomia | Esper | Marko Mutanen | 11-Jun-2009 | Finland | | Ostrobottnia borealis pars australis | | Kempele | 64,8809967 | 25,58600044 | |
| MM13983 | 658 | HM875824 | Nymphalidae | Heliconiinae | Boloria eunomia | Esper | Marko Mutanen | 11-Jun-2009 | Finland | | Ostrobottnia borealis pars australis | | Kempele | 64,8809967 | 25,58600044 | |
| MM17169 | 658 | JF853660 | Nymphalidae | Heliconiinae | Boloria eunomia | Esper | Marko Mutanen | 01-Jan-2005 | Finland | | Ostrobottnia borealis pars australis | | Kiiminki | 64,98100281 | 25,72900009 | |
| MM0340 | 658 | HQ57028 | Nymphalidae | Heliconiinae | Boloria napaea | Hoffmannsegg | Marko Mutanen | | Finland | Lappi | Lapponia enontekiensi | | Enontekioe | 68,9970016 | 20,7439994 | |
| MM0410 | 658 | HM38689 | Nymphalidae | Heliconiinae | Boloria napaea | Hoffmannsegg | Marko Mutanen | | Finland | | | | Enontekioe | 68,9970016 | 20,7439994 | |
| MM1716 | 658 | JN272537 | Nymphalidae | Heliconiinae | Boloria napaea | Hoffmannsegg | Tomi Mutanen | 02-Jul-2003 | Finland | | Lapponia enontekiensi | | Enontekioe | 69,0456008 | 20,8554000 | |
| TLMF Lep 0183 | 658 | HQ96830 | Nymphalidae | Heliconiinae | Boloria napaea | Hoffmannsegg | Mayr T. | 11-Jul-2009 | Switzerland | Graubunden | | | Val Bever, Alp Suvrett | 46,5499992 | 9,78332996 | |
| TLMF Lep 0183 | 658 | HQ968301 | Nymphalidae | Heliconiinae | Boloria napaea | Hoffmannsegg | Mayr T. | 01-Aug-2009 | Switzerland | Graubunden | | | Val Bever, Alp Suvrett | 46,5499992 | 9,78332996 | |
| TLMF Lep 0183 | 658 | HQ96830 | Nymphalidae | Heliconiinae | Boloria napaea | Hoffmannsegg | Mayr T. | 11-Jul-2009 | Switzerland | Graubunden | | | Val Bever, Alp Suvrett | 46,5499992 | 9,78332996 | |
| BC ZSM Lep 21896 | 658 | JF415688 | Nymphalidae | Heliconiinae | Boloria thore | Hübner | Buchsbaum | 17-Jul-1999 | Germany | Bavaria | Oberbayern | | Wettersteingebirge, Kreuzeck-Stuibensee | 47,45280075 | 11,0685997 | |
| BC ZSM Lep 2712 | 658 | HM39182 | Nymphalidae | Heliconiinae | Boloria thore | Hübner | Dr. Andreas H. Segerer | 18-Jun-1996 | Germany | Bavaria | Oberbayern | Traunstein | Ruhpolding, Eschelmoo | 47,7288017 | 12,559399 | |
| BC ZSM Lep 3054 | 658 | GU68847 | Nymphalidae | Heliconiinae | Boloria thore | Hübner | E. Albert | 13-Jun-2009 | Germany | Bavaria | | | Laubau | 47,71120071 | 12,65620041 | |
| BC ZSM Lep 5041 | 658 | JX034684 | Nymphalidae | Heliconiinae | Boloria thore | Hübner | Alfred Haslberge | 01-Jul-2008 | Germany | Bavaria | Schwaben | Oberallgaeu | Oberstdorf, Oyta | 47,3894996 | 10,3199996 | |
| MM0340 | 658 | HQ57028 | Nymphalidae | Heliconiinae | Boloria thore | Hübner | Marko Mutanen | | Finland | Lappi | Lapponia enontekiensi | | Enontekioe | 68,9970016 | 20,7439994 | |
| MM0409 | 627 | HM38688 | Nymphalidae | Heliconiinae | Boloria thore | Hübner | Marko Mutanen | | Finland | | | | Enontekioe | 68,9970016 | 20,7439994 | |
| MM0409 | 658 | HM38688 | Nymphalidae | Heliconiinae | Boloria thore | Hübner | Marko Mutanen | | Finland | | | | Enontekioe | 68,9970016 | 20,7439994 | |
| TLMF Lep 01092 | 603 | JX034685 | Nymphalidae | Heliconiinae | Boloria thore | Hübner | Aistleitner U. | 02-Jul-2009 | Switzerland | Graubunden | | | Tiefencastel S/ Salouf, Got Grond | 46,65000153 | 9,597000122 | |
| TLMF Lep 0183 | 658 | HQ96829 | Nymphalidae | Heliconiinae | Boloria thore | Hübner | Mayr T. | 14-Jun-2009 | Switzerland | Graubunden | | | Chur, Pagig | 46,8333015 | 9,63333034 | |
| TLMF Lep 0183 | 658 | HQ96829 | Nymphalidae | Heliconiinae | Boloria thore | Hübner | Mayr T. | 05-Jul-2009 | Switzerland | Sankt Gallen | | | Alt St. Johann, Gmeinenwie | 47,21670151 | 9,25 | |
| TLMF Lep 0183 | 658 | HQ96829 | Nymphalidae | Heliconiinae | Boloria thore | Hübner | Mayr T. | 05-Jul-2009 | Switzerland | Sankt Gallen | | | Alt St. Johann, Gmeinenwie | 47,21670151 | 9,25 | |
| TLMF Lep 0272 | 658 | JF860244 | Nymphalidae | Heliconiinae | Boloria thore | Hübner | Schmid J. | 05-Jul-2006 | Switzerland | Graubunden | | | Riefawald/ Vals | 46,6180000 | 9,194000244 | |
| MM08052 | 658 | HM873750 | Gelechiidae | Gelechiinae | Bryotropha boreella | Douglas | Marko Mutanen, Panu Vaelimaeki | | Finland | | Lapponia kemensis pars occidentalis | | Kolari | 67,27600098 | 23,75300026 | |
| MM15622 | 658 | HM876411 | Gelechiidae | Gelechiinae | Bryotropha boreella | Douglas | Marko Mutanen | 14-Jul-2004 | Finland | | Ostrobottnia borealis pars australis | | Kiiminki | 65,07099915 | 25,72500038 | |
| MM1562 | 658 | HM87641 | Gelechiidae | Gelechiinae | Bryotropha boreella | Douglas | Marko Mutanen | | Finland | | Regio kuusamoensi | | Kuusamo | 66,3160018 | 29,2299995 | |
| TLMF Lep 0174 | 658 | HQ96894 | Gelechiidae | Gelechiinae | Bryotropha boreella | Douglas | Huemer P | 11-Jun-2010 | Austria | Steiermark | | | Woerschacher Moos N | 47,5581016 | 14,178299 | |
| TLMF Lep 0174 | 658 | HQ96894 | Gelechiidae | Gelechiinae | Bryotropha boreella | Douglas | Huemer P | 11-Jun-2010 | Austria | Steiermark | | | Woerschacher Moos N | 47,5581016 | 14,178299 | |
| TLMF Lep 0174 | 658 | HQ96894 | Gelechiidae | Gelechiinae | Bryotropha boreella | Douglas | Huemer P | 11-Jun-2010 | Austria | Steiermark | | | Woerschacher Moos N | 47,5581016 | 14,178299 | |
| TLMF Lep 0174 | 658 | HQ96894 | Gelechiidae | Gelechiinae | Bryotropha boreella | Douglas | Huemer P | 11-Jun-2010 | Austria | Steiermark | | | Woerschacher Moos N | 47,5581016 | 14,178299 | |
| CLV15101 | 658 | JF855560 | Gracillariidae | Gracillariinae | Callisto coffeella | Zetterstedt | Helmut Deutsch | 17-Jun-2005 | Austria | | Osttirol | Lienzer Dolomiten | Lavanter Almta | 46,7747001 | 12,8538999 | |
| CLV1511 | 658 | JF855561 | Gracillariidae | Gracillariinae | Callisto coffeella | Zetterstedt | Helmut Deutsch | 17-Jun-2005 | Austria | | Osttirol | Lienzer Dolomiten | Lavanter Almta | 46,7747001 | 12,8538999 | |
| CLV1965 | 658 | JF848501 | Gracillariidae | Gracillariinae | Callisto coffeella | Zetterstedt | Stanislav Gombo | 17-Jun-2005 | Austria | Tyrol | Osttirol | Lienzer Dolomiten | Lavanter Almta | 46,79199982 | 12,8059997 | |
| MM0852 | 658 | HQ57036 | Gracillariidae | Gracillariinae | Callisto coffeella | Zetterstedt | Tomi Mutanen | 29-Jun-2003 | Finland | | Lapponia enontekiensi | | Enontekioe | 68,9970016 | 20,7439994 | |
| MM0852 | 658 | HQ57036 | Gracillariidae | Gracillariinae | Callisto coffeella | Zetterstedt | Tomi Mutanen | 29-Jun-2003 | Finland | | Lapponia enontekiensi | | Enontekioe | 68,9970016 | 20,7439994 | |
| MM08573 | 658 | HM873980 | Gracillariidae | Gracillariinae | Callisto coffeella | Zetterstedt | Marko Mutanen, Anttoni Mutanen | 14-Jun-2008 | Finland | | Ostrobottnia borealis pars borealis | | Rovaniemi | 66,50499725 | 25,65200043 | |
| MM1809 | 658 | JN27196 | Gracillariidae | Gracillariinae | Callisto coffeella | Zetterstedt | Tomi Mutanen | 06-Jul-2003 | Finland | | Lapponia enontekiensi | | Enontekioe | 69,0456008 | 20,8554000 | |
| TLMF Lep 0061 | 647 | HM42598 | Gracillariidae | Gracillariinae | Callisto coffeella | Zetterstedt | Huemer P | 20-Jul-2009 | Italy | | Belluno | | Passo di Valparola E | 46,5255012 | 11,9997997 | |
| TLMF Lep 0061 | 658 | HM38145 | Gracillariidae | Gracillariinae | Callisto coffeella | Zetterstedt | Huemer P | 20-Jul-2009 | Italy | | Belluno | | Passo di Valparola E | 46,5255012 | 11,9997997 | |
| TLMF Lep 0093 | 658 | HQ96844 | Gracillariidae | Gracillariinae | Callisto coffeella | Zetterstedt | Huemer P | 26-Jun-1989 | Austria | Vorarlberg | Umg.Brand | | S-Schafgafal | 47,0670013 | 9,76700019 | |
| TLMF Lep 0180 | 658 | HQ968271 | Gracillariidae | Gracillariinae | Callisto coffeella | Zetterstedt | Huemer P | 20-Jul-2009 | Italy | | Belluno | | Passo di Valparola E | 46,5255012 | 11,9997997 | |
| TLMF Lep 0180 | 658 | HQ96827 | Gracillariidae | Gracillariinae | Callisto coffeella | Zetterstedt | Huemer P | 20-Jul-2009 | Italy | | Belluno | | Passo di Valparola E | 46,5255012 | 11,9997997 | |
| TLMF Lep 0193 | 658 | HQ96834 | Gracillariidae | Gracillariinae | Callisto coffeella | Zetterstedt | Huemer P | 08-Jul-2010 | Austria | | Vorarlberg | | E Laguzalpe/ Maru | 47,1932983 | 9,9533300 | |
| TLMF Lep 0193 | 658 | HQ96834 | Gracillariidae | Gracillariinae | Callisto coffeella | Zetterstedt | Huemer P | 08-Jul-2010 | Austria | | Vorarlberg | | E Laguzalpe/ Maru | 47,1932983 | 9,9533300 | |
| TLMF Lep 0193 | 658 | HQ96834 | Gracillariidae | Gracillariinae | Callisto coffeella | Zetterstedt | Huemer P | 08-Jul-2010 | Austria | | Vorarlberg | | E Laguzalpe/ Maru | 47,1932983 | 9,9533300 | |
| TLMF Lep 0193 | 658 | HQ96834 | Gracillariidae | Gracillariinae | Callisto coffeella | Zetterstedt | Huemer P | 08-Jul-2010 | Austria | | Vorarlberg | | E Laguzalpe/ Maru | 47,1932983 | 9,9533300 | |
| TLMF Lep 0271 | 658 | JF860237 | Gracillariidae | Gracillariinae | Callisto coffeella | Zetterstedt | Schmid J. | 24-May-2008 | Switzerland | Graubunden | | | Riefawald/ Vals | 46,6180000 | 9,194000244 | |
| 06-PROBE-017 | 650 | JX008262 | Geometridae | Larentiinae | Carsia sororiata | Hübner | P.D.N. Hebert | | Canada | Manitoba | Churchill | 23 km E Churchill, Ramsay Cree | locality unconfirmed | 58,7299995 | -93,7799987 | |
| 06-PROBE-266 | 651 | JX008256 | Geometridae | Larentiinae | Carsia sororiata | Hübner | P.D.N. Hebert | 16-Aug-2006 | Canada | Manitoba | Churchill | 22 km E Churchill, CNSC | | 58,7379989 | -93,8190002 | |
| MM0143 | 658 | HM38672 | Geometridae | Larentiinae | Carsia sororiata | Hübner | Marko Mutanen, Panu Vaelimae | | Finland | | | | Imatra | 61,10800171 | 28,7989997 | |
| MM02919 | 658 | HM871743 | Geometridae | Larentiinae | Carsia sororiata | Hübner | Marko Mutanen | | Finland | | Ostrobottnia borealis pars australis | | Kiiminki | 65,07099915 | 25,72500038 | |
| MM08117 | 658 | HM873785 | Geometridae | Larentiinae | Carsia sororiata | Hübner | Marko Mutanen, Panu Vaelimaeki | | Finland | | Lapponia kemensis pars occidentalis | | Kolari | 67,27600098 | 23,75300026 | |
| TLMF Lep 0271 | 658 | JF860238 | Geometridae | Larentiinae | Carsia sororiata | Hübner | Schmid J. | 02-Sep-2006 | Switzerland | Graubunden | | | Pl. Tschanueff/ Ramoscl | 46,8349990 | 10,375 | |
| TLMF Lep 0279 | 658 | JF860293 | Geometridae | Larentiinae | Carsia sororiata | Hübner | Naessig W. & Weyh R. | 17-Aug-2010 | Austria | Tyrol | Osttiro | | Karn.A., Untertilliach, Winklerta | 46,68199921 | 12,6540002 | |
| MM1757 | 658 | JX034639 | Gelechiidae | Gelechiinae | Caryocolum petrophila | Preissecker | Juhani Itaemies | | Finland | | Nylandia | | Tammisaar | 59,8190002 | 23,2558994 | |
| MM17972 | 658 | JF854029 | Gelechiidae | Gelechiinae | Caryocolum petrophila | Preissecker | Marko Mutanen | 29-Jul-2010 | Finland | | Ostrobottnia borealis pars australis | | Oulunsalo | 64,99369812 | 25,21969986 | |
| MM17973 | 658 | JF854030 | Gelechiidae | Gelechiinae | Caryocolum petrophila | Preissecker | Marko Mutanen | 29-Jul-2010 | Finland | | Ostrobottnia borealis pars australis | | Oulunsalo | 64,99369812 | 25,21969986 | |
| TLMF Lep 0169 | 658 | HQ96889 | Gelechiidae | Gelechiinae | Caryocolum petrophila | Preissecker | Huemer P | 09-Aug-2010 | Slovenia | | | | Veliki Mangar | 46,4384994 | 13,6352996 | |
| TLMF Lep 0169 | 658 | HQ96890 | Gelechiidae | Gelechiinae | Caryocolum petrophila | Preissecker | Huemer P | 09-Aug-2010 | Slovenia | | | | Veliki Mangar | 46,4384994 | 13,6352996 | |
| TLMF Lep 0271 | 658 | JF860239 | Gelechiidae | Gelechiinae | Caryocolum petrophila | Preissecker | Schmid J. | 21-Jul-2009 | Switzerland | Graubunden | | | Crot/ Avers | 46,4770011 | 9,48400020 | |
| TLMF Lep 05269 | 658 | JX034690 | Gelechiidae | Gelechiinae | Caryocolum petrophila | Preissecker | Huemer P. & Tarmann G. M. | 28-Jul-2011 | Macedonia | | | | Mavrovo NP, Korab, Korabska jezero, Kobilino pole | 41,77799988 | 20,58200073 | genitalia in glycerin |
| MM1564 | 658 | HM87643 | Gelechiidae | Gelechiinae | Caryocolum petry | O. Hofmann | Timo Nupponen, Kari Nuppone | | Finland | | Satakunda | | Saekylae | 60,9599990 | 22,3829994 | |
| MM1739 | 658 | JF853815 | Gelechiidae | Gelechiinae | Caryocolum petry | O. Hofmann | Juhani Itaemies, Arja Itaemies | 24-Jun-2004 | Finland | | Satakunda | | Saekylae | 61,0419998 | 22,4270000 | |
| MM1740 | 658 | JF853816 | Gelechiidae | Gelechiinae | Caryocolum petry | O. Hofmann | Juhani Itaemies, Arja Itaemies | 24-Jun-2004 | Finland | | Satakunda | | Saekylae | 61,0419998 | 22,4270000 | |
| TLMF Lep 0169 | 658 | HQ968901 | Gelechiidae | Gelechiinae | Caryocolum petry | O. Hofmann | Huemer P | 09-Aug-2010 | Slovenia | | | | Veliki Mangar | 46,4384994 | 13,6352996 | |
| TLMF Lep 0190 | 658 | HQ96832 | Gelechiidae | Gelechiinae | Caryocolum petry | O. Hofmann | Erlebach S. | 14-Jul-1997 | Austria | Karnten | | | W Goessnitzbrette | | | |
| TLMF Lep 0191 | 658 | JF859623 | Gelechiidae | Gelechiinae | Caryocolum petry | O. Hofmann | Erlebach S. | 14-Jul-1997 | Austria | Karnten | | | W Goessnitzbrette | | | |
| TLMF Lep 0191 | 658 | HQ96832 | Gelechiidae | Gelechiinae | Caryocolum petry | O. Hofmann | Erlebach S. | 14-Jul-1997 | Austria | Karnten | | | W Goessnitzbrette | | | |
| TLMF Lep 0271 | 658 | JF860240 | Gelechiidae | Gelechiinae | Caryocolum petry | O. Hofmann | Schmid J. | 30-Jun-2009 | Switzerland | Graubunden | | | Riefawald/ Vals | 46,6180000 | 9,194000244 | |
| 08-JDWBC-0080 | 658 | JX008271 | Gelechiidae | Gelechiinae | Caryocolum pullatella | Tengström | deWaard, J.R. | 05-Jun-2008 | Canada | British Columbia | Winfield | Beaver Lake Rd | | 50,0219993 | -119,324996 | |
| CNCLEP0002529 | 658 | JX008225 | Gelechiidae | Gelechiinae | Caryocolum pullatella | Tengström | JF Landry & L Humble | 01-Jun-2006 | Canada | British Columbia | | 4.5 km NE Kelowna | Beaver Lake Ro | 50,0359993 | -119,348999 | MIC5727 M |
| CNCLEP0002529 | 657 | JX008182 | Gelechiidae | Gelechiinae | Caryocolum pullatella | Tengström | JF Landry & L Humble | 01-Jun-2006 | Canada | British Columbia | | 4.5 km NE Kelowna | Beaver Lake Ro | 50,0359993 | -119,348999 | |
| CNCLEP0002529 | 658 | JX008461 | Gelechiidae | Gelechiinae | Caryocolum pullatella | Tengström | JF Landry & L Humble | 01-Jun-2006 | Canada | British Columbia | | 4.5 km NE Kelowna | Beaver Lake Ro | 50,0359993 | -119,348999 | MIC5728 F |
| CNCLEP0002529 | 658 | JX008376 | Gelechiidae | Gelechiinae | Caryocolum pullatella | Tengström | JF Landry & L Humble | 01-Jun-2006 | Canada | British Columbia | | 4.5 km NE Kelowna | Beaver Lake Ro | 50,0359993 | -119,348999 | |
| CNCLEP0002529 | 658 | JX008368 | Gelechiidae | Gelechiinae | Caryocolum pullatella | Tengström | JF Landry & L Humble | 01-Jun-2006 | Canada | British Columbia | | 4.5 km NE Kelowna | Beaver Lake Ro | 50,0359993 | -119,348999 | |
| CNCLEP0002529 | 636 | JX008180 | Gelechiidae | Gelechiinae | Caryocolum pullatella | Tengström | JF Landry & L Humble | 01-Jun-2006 | Canada | British Columbia | | 4.5 km NE Kelowna | Beaver Lake Ro | 50,0359993 | -119,348999 | |
| CNCLEP0002529 | 658 | JX008242 | Gelechiidae | Gelechiinae | Caryocolum pullatella | Tengström | JF Landry & L Humble | 01-Jun-2006 | Canada | British Columbia | | 4.5 km NE Kelowna | Beaver Lake Ro | 50,0359993 | -119,348999 | |

| | | | | | | | | | | | | | | | | |
|---|---|---|---|---|---|---|---|---|---|---|---|---|---|---|---|---|
| CNCLEP00025300 | 658 | JX008251 | Gelechiidae | Gelechiinae | Caryocolum pullatella | Tengström | JF Landry & L Humble | 01-Jun-2006 | Canada | British Columbia | | 4.5 km NE Kelowna | Beaver Lake Rd | 50,0359993 | -119,348999 | |
| CNCLEP00025301 | 655 | JX008333 | Gelechiidae | Gelechiinae | Caryocolum pullatella | Tengström | JF Landry & L Humble | 01-Jun-2006 | Canada | British Columbia | | 4.5 km NE Kelowna | Beaver Lake Rd | 50,0359993 | -119,348999 | |
| CNCLEP00025302 | 658 | JX008299 | Gelechiidae | Gelechiinae | Caryocolum pullatella | Tengström | JF Landry & L Humble | 01-Jun-2006 | Canada | British Columbia | | 4.5 km NE Kelowna | Beaver Lake Rd | 50,0359993 | -119,348999 | |
| MDOK-1684 | 658 | GU800793 | Gelechiidae | Gelechiinae | Caryocolum pullatella | Tengström | Mark Dreiling | 22-Oct-2008 | United States | Oklahoma | | Bartlesville | 1301 Cherokee Hills Drive | 36,74000168 | -95,94999695 | |
| MDOK-2240 | 602 | GU800945 | Gelechiidae | Gelechiinae | Caryocolum pullatella | Tengström | Mark Dreiling | 08-May-2009 | United States | Oklahoma | | Bartlesville | 1301 Cherokee Hills Drive | 36,74000168 | -95,94999695 | |
| MDOK-2537 | 658 | GU801318 | Gelechiidae | Gelechiinae | Caryocolum pullatella | Tengström | Mark Dreiling | 04-Jun-2009 | United States | Oklahoma | | Bartlesville | 1301 Cherokee Hills Drive | 36,74000168 | -95,94999695 | |
| MDOK-4041 | 658 | JF857861 | Gelechiidae | Gelechiinae | Caryocolum pullatella | Tengström | Mark Dreiling | 06-Jun-2010 | United States | Oklahoma | | Bartlesville | 1301 Cherokee Hills Drive | 36,74000168 | -95,94999695 | |
| MM00778 | 658 | HM871187 | Gelechiidae | Gelechiinae | Caryocolum pullatella | Tengström | Marko Mutanen | | Finland | | Ostrobottnia borealis pars australis | | Kiiminki | 65,07099915 | 25,72500038 | |
| MM03085 | 658 | HM871813 | Gelechiidae | Gelechiinae | Caryocolum pullatella | Tengström | Marko Mutanen | | Finland | | Ostrobottnia borealis pars australis | | Kiiminki | 65,07099915 | 25,72500038 | |
| MM09053 | 658 | HM874153 | Gelechiidae | Gelechiinae | Caryocolum pullatella | Tengström | Marko Mutanen, Panu Vaelimaeki | | Finland | | Karelia ladogensis | | Simpele | 61,42300034 | 29,32299995 | |
| MM14646 | 658 | HM876200 | Gelechiidae | Gelechiinae | Caryocolum pullatella | Tengström | Marko Mutanen, Nestori Mutanen, Anttoni Mutanen | 27-Jul-2009 | Finland | | Ostrobottnia borealis pars australis | | Kiiminki | 65,07099915 | 25,72500038 | |
| SL0359 | 658 | HM902807 | Gelechiidae | Gelechiinae | Caryocolum pullatella | Tengström | S. Lee | 04-Aug-2008 | United States | Arkansas | Conway Co. | | Petit Jean St. Pk | 35,1189003 | -92,93440247 | |
| TLMF Lep 02876 | 658 | JF860338 | Gelechiidae | Gelechiinae | Caryocolum pullatella | Tengström | Baldizzone G. | 20-Jul-2006 | Italy | Aosta | | | Champorcher, Dondena sent. Mt. Rascias | 45,60300064 | 7,506000042 | 21013JN |
| TLMF Lep 05272 | 658 | JX034621 | Gelechiidae | Gelechiinae | Caryocolum pullatella | Tengström | Huemer P. & Tarmann G. M. | 28-Jul-2011 | Macedonia | | | | Mavrovo NP, Korab, Korabska jezero, Kobilino pole | 41,77799988 | 20,58200073 | |
| TLMF Lep 05273 | 658 | JX034655 | Gelechiidae | Gelechiinae | Caryocolum pullatella | Tengström | Huemer P. & Tarmann G. M. | 28-Jul-2011 | Macedonia | | | | Mavrovo NP, Korab, Korabska jezero, Kobilino pole | 41,77799988 | 20,58200073 | |
| TLMF Lep 05276 | 658 | JX034697 | Gelechiidae | Gelechiinae | Caryocolum pullatella | Tengström | Huemer P. & Tarmann G. M. | 28-Jul-2011 | Macedonia | | | | Mavrovo NP, Korab, Korabska jezero, Kobilino pole | 41,77799988 | 20,58200073 | |
| MM00077 | 658 | HM396356 | Crambidae | Crambinae | Catoptria furcatellus | Zetterstedt | Marko Mutanen | | Finland | | | | Enontekioe | 68,99700165 | 20,74399948 | |
| MM14585 | 658 | HM876176 | Crambidae | Crambinae | Catoptria furcatellus | Zetterstedt | Marko Mutanen | | Finland | | Lapponia enontekiensis | | Enontekioe | 69,06300354 | 21,10199928 | |
| MM18392 | 658 | JN266125 | Crambidae | Crambinae | Catoptria furcatellus | Zetterstedt | Marko Mutanen, Nestori Mutanen, Anttoni Mutanen | 10-Jul-2010 | Finland | | Lapponia inarensis | | Utsjoki | 69,82800293 | 27 | |
| TLMF Lep 02715 | 658 | JN266133 | Crambidae | Crambinae | Catoptria furcatellus | Zetterstedt | Schmid J. | 25-Jul-2009 | Switzerland | Graubunden | | | Umg. Piz Minschuns, V. Costainas | 46,57920074 | 10,48330021 | |
| TLMF Lep 02854 | 658 | JF860332 | Crambidae | Crambinae | Catoptria furcatellus | Zetterstedt | Tarmann G | 10-Aug-1990 | Austria | | Osttiro | | Westlicher Sattelkopf/ Lienz NW | 46,90000153 | 12,76299953 | |
| TLMF Lep 02855 | 658 | JF860333 | Crambidae | Crambinae | Catoptria furcatellus | Zetterstedt | Tarmann G | 10-Aug-1990 | Austria | | Osttiro | | Westlicher Sattelkopf/ Lienz NW | 46,90000153 | 12,76299953 | |
| MM02573 | 658 | HM871617 | Gelechiidae | Gelechiinae | Chionodes holosericella | Herrich-Schäffer | Marko Mutanen, Panu Vaelimaeki | | Finland | | Savonia australis | | Imatra | 61,10800171 | 28,79899979 | |
| MM08090 | 658 | HM873774 | Gelechiidae | Gelechiinae | Chionodes holosericella | Herrich-Schäffer | Marko Mutanen, Panu Vaelimaeki | | Finland | | Lapponia kemensis pars occidentalis | | Kolari | 67,27600098 | 23,75300026 | |
| MM10386 | 658 | HM874686 | Gelechiidae | Gelechiinae | Chionodes holosericella | Herrich-Schäffer | family Marko Mutanen | 22-Jul-2008 | Finland | Aland | | | Eckeroe | 60,24000168 | 19,5890007 | |
| TLMF Lep 01912 | 658 | JF859624 | Gelechiidae | Gelechiinae | Chionodes holosericella | Herrich-Schäffer | Huemer P | 30-Jul-1999 | Austria | | Vorarlberg | Brandnerta | Schattenlagan | 47,0727005 | 9,75 | |
| TLMF Lep 01913 | 658 | JF859625 | Gelechiidae | Gelechiinae | Chionodes holosericella | Herrich-Schäffer | Huemer P | 29-Jun-1999 | Austria | | Vorarlberg | Brandnerta | Daleuwald SW | 47,11970139 | 9,756110191 | |
| TLMF Lep 01914 | 633 | JF859626 | Gelechiidae | Gelechiinae | Chionodes holosericella | Herrich-Schäffer | Huemer P. & Erlebach S | 04-Aug-1999 | Austria | Karnten | Umg.St.Jakob im Les | | Mussen Ost, (9 | 46,71670151 | 12,93330002 | |
| TLMF Lep 01915 | 658 | HQ968327 | Gelechiidae | Gelechiinae | Chionodes holosericella | Herrich-Schäffer | Huemer P. | 16-Jul-2009 | Austria | | Vorarlberg | | Marul, Lagutzbach, Beim alten Stafel | 47,20220184 | 9,928059578 | |
| TLMF Lep 01916 | 658 | HQ968328 | Gelechiidae | Gelechiinae | Chionodes holosericella | Herrich-Schäffer | Huemer P. | 07-Aug-2009 | Austria | | Vorarlberg | | Sonntag, Buchboden, NNE Untere Alpschellaalpe | 47,2378006 | 10,03030014 | |
| MM00693 | 658 | HM871135 | Gelechiidae | Gelechiinae | Chionodes luctuella | Hübner | Tomi Mutanen | 10-Jul-2006 | Finland | | Ostrobottnia borealis pars borealis | | Ranua | 65,88200378 | 26,34199905 | |
| MM09778 | 658 | HM387036 | Gelechiidae | Gelechiinae | Chionodes luctuella | Hübner | Marko Mutanen, Meelika Mutanen, Atsalea Mutanen, Sylvia Mutanen | 18-Jul-2008 | Finland | | | | Eckeroe | 60,27500153 | 19,57999992 | |
| MM10155 | 658 | HM874598 | Gelechiidae | Gelechiinae | Chionodes luctuella | Hübner | Marko Mutanen, Nestori Mutanen, Anttoni Mutanen, Tomi Mutanen | 28-Jul-2008 | Finland | | Ostrobottnia borealis pars australis | | Oulu | 64,97699738 | 25,30599976 | |
| TLMF Lep 01907 | 658 | HQ968323 | Gelechiidae | Gelechiinae | Chionodes luctuella | Hübner | Huemer P | 29-Jun-1999 | Austria | | Vorarlberg | Brandnerta | Daleuwald SW | 47,11970139 | 9,756110191 | |
| TLMF Lep 01908 | 631 | HQ968324 | Gelechiidae | Gelechiinae | Chionodes luctuella | Hübner | Huemer P | 29-Jun-1999 | Austria | | Vorarlberg | Brandnerta | Daleuwald SW | 47,11970139 | 9,756110191 | |
| 08BBLEP-02431 | 658 | JX008122 | Gelechiidae | Gelechiinae | Chionodes lugubrella | Fabricius | J.Straka,J.Cossey | 26-Jul-2008 | Canada | Alberta | Waterton | Waterton Lakes Nat. Park - Maskinonge Lake | Marsh/meadow | 49,11000061 | -113,8399963 | |
| 08BBLEP-02504 | 658 | JX008466 | Gelechiidae | Gelechiinae | Chionodes lugubrella | Fabricius | J.Straka,J.Cossey | 28-Jul-2008 | Canada | Alberta | Banff | Banff Nat. Park - Tunnel Mountain Campground | Meadow/pine forest border | 51,19300079 | -115,5329971 | |
| 08BBLEP-02853 | 658 | JX008193 | Gelechiidae | Gelechiinae | Chionodes lugubrella | Fabricius | J.Straka,J.Cossey | 25-Jul-2008 | Canada | Alberta | Waterton | Waterton Lakes Nat. Park - Carthew Creek | Meadow/creekside | 49,04899979 | -113,913002 | |
| 08BBLEP-03171 | 597 | JX008390 | Gelechiidae | Gelechiinae | Chionodes lugubrella | Fabricius | | 06-Aug-2008 | Canada | Alberta | Waterton | Waterton Lakes Nat. Park - Blakiston Creek | Rocky creekside | 49,07799911 | -113,8820038 | |
| 08BBLEP-03249 | 658 | JX008456 | Gelechiidae | Gelechiinae | Chionodes lugubrella | Fabricius | J.Straka,J.Cossey | 08-Aug-2008 | Canada | Alberta | Waterton | Waterton Lakes Nat. Park - Coppermine Creek | Roadside meadow | 49,10499954 | -113,9589996 | |
| CNCLEP00061416 | 658 | GU693340 | Gelechiidae | Gelechiinae | Chionodes lugubrella | Fabricius | B.C. Schmidt | 23-Jun-2004 | Canada | Yukon Territory | 2 km N Carcross | Sand dunes | | 60,18799973 | -134,6959991 | MIC5790 M |
| CNCLEP00061417 | 658 | GU693341 | Gelechiidae | Gelechiinae | Chionodes lugubrella | Fabricius | B.C. Schmidt | 23-Jun-2004 | Canada | Yukon Territory | 2 km N Carcross | Sand dunes | | 60,18799973 | -134,6959991 | |
| MM03079 | 615 | HM871810 | Gelechiidae | Gelechiinae | Chionodes lugubrella | Fabricius | Marko Mutanen | | Finland | | Ostrobottnia borealis pars australis | | Kiiminki | 65,07099915 | 25,72500038 | |
| MM03257 | 658 | HM871902 | Gelechiidae | Gelechiinae | Chionodes lugubrella | Fabricius | Marko Mutanen | 29-Jun-2006 | Finland | Oulun laani | Ostrobottnia borealis pars australis | | Kiiminki | 65,07099915 | 25,72500038 | |
| MM13505 | 658 | HM875628 | Gelechiidae | Gelechiinae | Chionodes lugubrella | Fabricius | Marko Mutanen, Panu Vaelimaeki | | Finland | | Regio Aboensis | | Dragsfjaerd | 60,01100159 | 22,49799919 | |
| NoA-08-250 | 587 | JX008421 | Gelechiidae | Gelechiinae | Chionodes lugubrella | Fabricius | D.A. Macaulay, G.R. Pohl, A.D. Roe | 06-Jul-2001 | Canada | Alberta | | | La Butte Ck Wildlands Prov Pk | 59,42699814 | -111,4440002 | |
| NoA-08-251 | 658 | JX008150 | Gelechiidae | Gelechiinae | Chionodes lugubrella | Fabricius | D.A. Macaulay | 22-Jun-2002 | Canada | Alberta | | | Ft Assiniboine | 55,30099869 | -114,8280029 | |
| NoA-08-252 | 658 | JX008278 | Gelechiidae | Gelechiinae | Chionodes lugubrella | Fabricius | | 15-Jul-2004 | Canada | Alberta | | | Birch Mtns Wildland Pk | 57,57099915 | -112,4499969 | |
| NoA-08-253 | 658 | JX008334 | Gelechiidae | Gelechiinae | Chionodes lugubrella | Fabricius | | 08-Jul-2004 | Canada | Alberta | | | Birch Mtns Wildland Pk | 57,58000183 | -112,4670029 | |
| NoA-08-254 | 658 | JX008457 | Gelechiidae | Gelechiinae | Chionodes lugubrella | Fabricius | | 25-Jul-2003 | Canada | Alberta | | | Red Lodge Prov Pk | 51,95000076 | -114,25 | |
| TLMF Lep 01887 | 658 | HQ968315 | Gelechiidae | Gelechiinae | Chionodes lugubrella | Fabricius | Nel J. | 07-Jul-2004 | France | Provence-Alpes-Cote d'Azur | | | Valle du Fournel | 44,7820015 | 6,513000011 | |
| TLMF Lep 01888 | 658 | HQ968316 | Gelechiidae | Gelechiinae | Chionodes lugubrella | Fabricius | | 25-Jul-1992 | France | Auvergne | | | Saint-Christophe | 45 | 3,816669941 | |
| TLMF Lep 02805 | 658 | JF860300 | Gelechiidae | Gelechiinae | Chionodes lugubrella | Fabricius | Huemer P | 11-Jun-2010 | Austria | Steiermark | | | Woerschacher Moos N | 47,55799866 | 14,17800045 | |
| MM00086 | 658 | HM396365 | Gelechiidae | Gelechiinae | Chionodes viduella | Fabricius | Marko Mutanen | | Finland | | | | Enontekioe | 68,99700165 | 20,74399948 | |
| MM03439 | 658 | JX034603 | Gelechiidae | Gelechiinae | Chionodes viduella | Fabricius | Marko Mutanen | | Finland | | Lapponia enontekiensis | | Enontekioe | 68,99700165 | 20,74399948 | |
| MM03893 | 658 | HM872209 | Gelechiidae | Gelechiinae | Chionodes viduella | Fabricius | Marko Mutanen | | Finland | Ita-Suomen Laan | Karelia ladogensis | | Kesaelaht | 61,79299927 | 29,71100044 | |
| MM04153 | 658 | HM386940 | Gelechiidae | Gelechiinae | Chionodes viduella | Fabricius | Marko Mutanen | | Finland | | | | Enontekioe | 68,99700165 | 20,74399948 | |
| MM06284 | 658 | HM873171 | Gelechiidae | Gelechiinae | Chionodes viduella | Fabricius | Marko Mutanen, Nestori Mutanen, Anttoni Mutanen | 27-Jun-2007 | Finland | | Lapponia kemensis pars orientalis | | Sodankylae | 68,22299957 | 27,21699905 | |
| TLMF Lep 01894 | 658 | JX034574 | Gelechiidae | Gelechiinae | Chionodes viduella | Fabricius | Nel J. | 21-Jul-1995 | France | Provence-Alpes-Cote d'Azur | | | Col du Galibier | 45,06420135 | 6,391940117 | 3544JN |
| TLMF Lep 01895 | 658 | JF859620 | Gelechiidae | Gelechiinae | Chionodes viduella | Fabricius | Deutsch H. | 19-Jun-2000 | Austria | Tyrol | Osttirol, Lienzer Dolomiten | | Lavanter Almtal/ Nikolsdorf SW | 46,76890182 | 12,84889984 | |

| | | | | | | | | | | | | | | | |
|---|---|---|---|---|---|---|---|---|---|---|---|---|---|---|---|
| TLMF Lep 01896 | 658 | JF859621 | Gelechiidae | Gelechiinae | Chionodes viduella | Fabricius | Deutsch H. | 02-Jul-1992 | Austria | Tyrol | Osttirol, Teischnitztal | | Teischnitztal/ Talausgang S (linker Talhang | 47,02999878 | 12,63809967 |
| TLMF Lep 0189 | 658 | HQ96831 | Gelechiida | Gelechiina | Chionodes viduell | Fabricius | Deutsch H. | 22-Jul-1997 | Austria | Tyrol | Osttiro | | Lienzer Huette W/ Lienz | 46,9350013 | 12,7458000 |
| TLMF Lep 0271 | 658 | JF860241 | Gelechiida | Gelechiina | Chionodes viduell | Fabricius | Schmid J. | 09-Jun-2007 | Switzerlan | Graubunde | | | Miraniga, Kartitscha/ Obersaxe | 46,7299995 | 9,100000381 |
| MM1773 | 647 | JF853935 | Tortricida | Tortricina | Clepsis rogan | Guenée | Endel Mae | 10-Jun-199 | Estonia | | | | Kohala | | |
| MM1774 | 658 | JX034687 | Tortricida | Tortricina | Clepsis rogan | Guenée | D. Kolligs | | Czech Republi | | | | Simplo | | |
| MM1774 | 658 | JF853936 | Tortricida | Tortricina | Clepsis rogan | Guenée | Meiringe | 09-Jul-2004 | Czech Republi | | Grindel Al | | | | |
| TLMF Lep 00523 | 656 | HM381368 | Tortricidae | Tortricinae | Clepsis rogana | Guenée | Huemer P. | 23-Jul-2009 | Italy | | Cuneo | | Colle Fauniera Umgebung/ Demonte NW | 44,38560104 | 7,121940136 |
| TLMF Lep 00770 | 658 | HM426123 | Tortricidae | Tortricinae | Clepsis rogana | Guenée | Huemer P. | 15-Jul-2009 | Austria | Steiermark | | | Tauplitzalm, Umg. Naturfreundehau | 47,59579849 | 13,99499989 |
| TLMF Lep 00771 | 658 | HM426124 | Tortricidae | Tortricinae | Clepsis rogana | Guenée | Huemer P. | 15-Jul-2009 | Austria | Steiermark | | | Tauplitzalm, Umg. Naturfreundehau | 47,59579849 | 13,99499989 |
| TLMF Lep 01785 | 658 | HQ968984 | Tortricidae | Tortricinae | Clepsis rogana | Guenée | Huemer P. | 15-Jul-2009 | Austria | Steiermark | | | Tauplitzalm, Umg. Naturfreundehau | 47,59579849 | 13,99499989 |
| TLMF Lep 01786 | 658 | HQ968985 | Tortricidae | Tortricinae | Clepsis rogana | Guenée | Huemer P. | 15-Jul-2009 | Austria | Steiermark | | | Tauplitzalm, Umg. Naturfreundehau | 47,59579849 | 13,99499989 |
| TLMF Lep 0271 | 658 | JF860242 | Tortricida | Tortricina | Clepsis rogan | Guenée | Schmid J. | 26-Jun-2004 | Switzerlan | Graubunde | | | Fontaunas novas/ Vignog | 46,7260017 | 9,14500045 |
| 10-JDWBC-4036 | 658 | HM86595 | Noctuida | Noctuina | Coenophila opacifron | Grote | deWaard, J.R. | 30-Jul-200 | Canad | British Columbi | nr. Hazelto | Date Cr. Silvicultural Syste | A2-T | 55,465999 | -127,815002 |
| 10-JDWBC-4475 | 658 | HM86643 | Noctuida | Noctuina | Coenophila opacifron | Grote | deWaard, J.R. | 30-Jul-200 | Canad | British Columbi | nr. Hazelto | Date Creek Silvicultural System | B1-T | 55,4370002 | -127,801002 |
| BC ZSM Lep 3575 | 658 | HQ56547 | Noctuida | Noctuina | Coenophila subrose | Stephens | Weigert | 16-Aug-200 | German | Bavari | Oberbayer | | Bay. Wald, Schnellenzi | 48,8807983 | 13,7305002 |
| MM0173 | 651 | HM87086 | Noctuida | Noctuina | Coenophila subrose | Stephens | Marko Mutanen, Panu Vaelimae | | Finlan | | | | Imatra | 61,10800171 | 28,7989997 |
| MM0173 | 658 | HM87086 | Noctuida | Noctuina | Coenophila subrose | Stephens | Marko Mutanen, Panu Vaelimae | | Finlan | | | | Imatra | 61,10800171 | 28,7989997 |
| MM04650 | 632 | HM872501 | Noctuidae | Noctuinae | Coenophila subrosea | Stephens | Marko Mutanen | | Finland | Lansi-Suomen Laani | Regio Aboensis | | Turku | 60,44100189 | 22,20199966 |
| TLMF Lep 0074 | 658 | HM42609 | Noctuida | Noctuina | Coenophila subrose | Stephens | Huemer P | 26-Aug-200 | Austria | Steiermar | | | Woerschacher Moos NW | 47,5578002 | 14,1705999 |
| MM0392 | 658 | HM87223 | Coleophorida | Coleophorina | Coleophora pappiferell | Hofmann | Marko Mutane | 13-Jun-200 | Finlan | Ita-Suomen Laan | Karelia boreali | | Liperi | 62,61399841 | 29,4740009 |
| MM06384 | 658 | HQ570345 | Coleophoridae | Coleophorinae | Coleophora pappiferella | Hofmann | Marko Mutanen, Anttoni Mutanen, Nestori Mutane | 29-Jun-2007 | Finland | | Lapponia inarensis | | Utsjoki | 69,37599945 | 25,80500031 |
| MM1818 | 658 | JX034577 | Coleophorida | Coleophorina | Coleophora pappiferell | Hofmann | Marko Mutane | | Finlan | | Lapponia enontekiens | | Enontekioe | 68,9965972 | 20,7437000 |
| MM1818 | 658 | JF854186 | Coleophorida | Coleophorina | Coleophora pappiferell | Hofmann | Tomi Mutane | 05-Jun-2004 | Finlan | | Karelia boreali | | Liperi | 62,6376991 | 29,1443996 |
| TLMF Lep 02722 | 658 | JF860245 | Coleophoridae | Coleophorinae | Coleophora pappiferella | Hofmann | Schmid J. | 16-Jul-2009 | Switzerland | Graubunden | | | Avers-Cresta, Cucalner/Guggermue | 46,5019989 | 9,484999657 |
| MM0009 | 658 | HM39637 | Coleophorida | Coleophorina | Coleophora svensso | Baldizzone | Marko Mutane | | Finlan | | | | Enontekioe | 68,9970016 | 20,7439994 |
| MM0590 | 653 | HQ57032 | Coleophorida | Coleophorina | Coleophora svensso | Baldizzone | Panu Vaelimae | 03-Jul-2007 | Finlan | | Lapponia inarensi | | Utsjok | 69,375 | 25,7290000 |
| MM1385 | 658 | HM38706 | Coleophorida | Coleophorina | Coleophora svensso | Baldizzone | M. Mutane | | Finlan | | | | Enontekioe | 69,0350036 | 20,8080005 |
| TLMF Lep 0272 | 658 | JF860246 | Coleophorida | Coleophorina | Coleophora svensso | Baldizzone | Schmid J. | 04-Jul-200 | Switzerlan | Graubunde | | | NW Abhang Horn/ Val | 46,61000061 | 9,19499969 |
| MM15981 | 658 | JF853564 | Coleophoridae | Coleophorinae | Coleophora uliginosella | Glitz | Marko Mutanen, Panu Vaelimaeki, Tomi Mutanen, Petri Hirvonen | 03-Sep-2010 | Finland | | Nylandia | | Hanko | 59,83200073 | 23,07600021 |
| TLMF Lep 0284 | 658 | JF860328 | Coleophorida | Coleophorina | Coleophora uliginosell | Glitz | Huemer P | 27-Jun-200 | Austria | Vorarlberg | Umg.Dornbir | | Boedele, Fohramoo | 47,4169998 | 9,800000191 |
| MM0009 | 658 | HM39637 | Coleophorida | Coleophorina | Coleophora unigenell | Svensson | Marko Mutane | | Finlan | | | | Enontekioe | 68,9970016 | 20,7439994 |
| MM0416 | 646 | HM38695 | Coleophorida | Coleophorina | Coleophora unigenell | Svensson | Marko Mutane | | Finlan | | | | Enontekioe | 68,9970016 | 20,7439994 |
| MM1816 | 658 | JX034669 | Coleophorida | Coleophorina | Coleophora unigenell | Svensson | Marko Mutane | | Finlan | | Lapponia enontekiens | | Enontekioe | 68,9965972 | 20,7437000 |
| TLMF Lep 01879 | 407 | JX034627 | Coleophoridae | Coleophorinae | Coleophora unigenella | Svensson | Huemer P. | 15-May-1989 | Austria | Tyrol | Nordtirol, Lechtal | | Johannesbruecke E/ Forchach NE | 47,43220139 | 10,60919952 |
| BC ZSM Lep 0121 | 658 | HQ60113 | Geometrida | Larentiina | Colostygia turbat | Hübner | W. Schacht | 30-Jun-199 | German | Bavari | south | Garmisch | Kreuzeck | 47,4529991 | 11,0810003 |
| BC ZSM Lep 1651 | 624 | JX034578 | Geometrida | Larentiina | Colostygia turbat | Hübner | A. Hausman | 27-Jun-200 | Italy | | South Tyrol, Alto Adig | | Rojental 6km SE Reschen (B | 46,7832984 | 10,4499998 |
| BC ZSM Lep 1655 | 658 | JX034605 | Geometrida | Larentiina | Colostygia turbat | Hübner | A. Hausman | 27-Jun-200 | Italy | | South Tyrol, Alto Adig | Rojenta | 6km SE Reschen (BZ | 45,7832984 | 10,4499998 |
| BC ZSM Lep 2417 | 658 | GU68723 | Geometrida | Larentiina | Colostygia turbat | Hübner | A. Haslberge | 03-Jun-200 | German | Bavari | Oberbayer | Berchtesgadener Lan | Schneizlreuth, Schrecksatte | 47,6431999 | 12,8025999 |
| MM1046 | 658 | HM87474 | Geometrida | Larentiina | Colostygia turbat | Hübner | Marko Mutane | 10-Jul-1994 | Finlan | | Regio kuusamoensi | | Salla | 67,7450027 | 29,6019992 |
| MM1581 | 658 | HM87659 | Geometrida | Larentiina | Colostygia turbat | Hübner | Marko Mutane | 10-Jul-1994 | Finlan | | Regio kuusamoensi | | Salla | 67,7450027 | 29,6019992 |
| MM1844 | 647 | JF854327 | Geometrida | Larentiina | Colostygia turbat | Hübner | Marko Mutane | 10-Jul-1994 | Finlan | | Kuusam | | Salla | 67,7454986 | 29,6021995 |
| MM1844 | 658 | JF854328 | Geometrida | Larentiina | Colostygia turbat | Hübner | Marko Mutane | 10-Jul-1994 | Finlan | | Kuusam | | Salla | 67,7454986 | 29,6021995 |
| SE MNC Lep 0057 | 658 | JN274054 | Geometrida | Larentiina | Colostygia turbat | Hübner | S. Erlacher | 03-Jun-1994 | Austria | Tyro | Innsbruck | | Hallta | 47,3264999 | 11,5022001 |
| SE MNC Lep 0057 | 658 | JN27405 | Geometrida | Larentiina | Colostygia turbat | Hübner | S. Erlacher | 30-Jun-1994 | Austria | Tyro | Hinterhornbac | | Hochvoge | 50,75 | 14,3999996 |
| TLMF Lep 0026 | 658 | HM42576 | Geometrida | Larentiina | Colostygia turbat | Hübner | Huemer P | 13-Jul-200 | Austria | Karnte | | | Petzen N, Obere Krisch | 46,5060997 | 14,7569999 |
| TLMF Lep 00325 | 658 | HM425818 | Geometridae | Larentiinae | Colostygia turbata | Hübner | Huemer P. | 20-Jul-2009 | Italy | | Belluno | | Passo di Valparola E - Passo Falzareg | 46,5223999 | 12,00699997 |
| BC ZSM Lep 3448 | 658 | HQ56537 | Noctuida | Hadenina | Coranarta cordiger | Thunber | A. Haslberge | 12-May-200 | German | Bavari | Oberbayer | Traunstei | Petting, Schoenramer Fil | 47,897399 | 12,8514003 |
| BC ZSM Lep 37577 | 658 | JN272101 | Noctuidae | Hadeninae | Coranarta cordigera | Thunberg | Dr. Andreas H. Segerer | 13-Apr-2002 | Germany | Bavaria | Mittelfranken | Nuernberger Land | westl. Ernhofen bei Leinburg, Wolfsgrube HSTrasse noerdl. A6 | 49,41239929 | 11,33920002 |
| MM0387 | 658 | HM87219 | Noctuida | Hadenina | Coranarta cordiger | Thunber | Marko Mutane | | Finlan | Ita-Suomen Laan | Karelia ladogensi | | Kesaelaht | 61,7929992 | 29,7110004 |
| MM13976 | 658 | HM875821 | Noctuidae | Hadeninae | Coranarta cordigera | Thunberg | Marko Mutanen | 11-Jun-2009 | Finland | | Ostrobottnia borealis pars australi | | Kempele | 64,8809967 | 25,58600044 |
| MM1403 | 658 | HM87585 | Noctuida | Hadenina | Coranarta cordiger | Thunber | Marko Mutane | | Finlan | | Karelia boreali | | Tuupovaar | 62,375 | 30,9050006 |
| TLMF Lep 0182 | 658 | HQ96829 | Noctuida | Hadenina | Coranarta cordiger | Thunber | Mayr T. | 04-Jul-200 | Switzerlan | Graubunde | | | Val Bever, Alp Suvrett | 46,5499992 | 9,78332996 |
| 07PROBE-0007 | 658 | JX008459 | Crambida | Crambina | Crambus alienellu | Germar & Kaulfuss | P.D.N. Heber | 14-Jul-2007 | Canad | Manitob | Churchil | 23 km E Churchill, Ramsay Cree | | 58,7229995 | -93,80699921 |
| 07PROBE-0007 | 656 | JX008318 | Crambida | Crambina | Crambus alienellu | Germar & Kaulfuss | P.D.N. Heber | 14-Jul-2007 | Canad | Manitob | Churchil | 23 km E Churchill, Ramsay Cree | | 58,7229995 | -93,80699921 |
| 07PROBE-0007 | 658 | JX008134 | Crambida | Crambina | Crambus alienellu | Germar & Kaulfuss | P.D.N. Heber | 14-Jul-2007 | Canad | Manitob | Churchil | 23 km E Churchill, Ramsay Cree | | 58,7229995 | -93,80699921 |
| 07PROBE-00159 | 658 | JX008473 | Crambidae | Crambinae | Crambus alienellus | Germar & Kaulfuss | P.D.N. Hebert | 15-Jul-2007 | Canada | Manitoba | Churchill | 26 km SE Churchill, Twin Lakes burn site | | 58,61700058 | -93,81199646 |
| 07PROBE-03898 | 658 | JX008423 | Crambidae | Crambinae | Crambus alienellus | Germar & Kaulfuss | P.D.N. Hebert | 17-Jul-2007 | Canada | Manitoba | Churchill | 26 km SE Churchill, Twin Lakes fen | | 58,63199997 | -93,78800201 |
| 07PROBE-10167 | 658 | JX008486 | Crambidae | Crambinae | Crambus alienellus | Germar & Kaulfuss | P.D.N. Hebert | 19-Jul-2007 | Canada | Manitoba | Churchill | 26 km SE Churchill, Twin Lakes fen | | 58,63199997 | -93,78800201 |
| 07PROBE-1024 | 658 | JX008342 | Crambida | Crambina | Crambus alienellu | Germar & Kaulfuss | P.D.N. Heber | 21-Jul-2007 | Canad | Manitob | Churchil | Town of Churchil | | 58,76900101 | -94,1719970 |
| 07PROBE-1041 | 658 | JX008261 | Crambida | Crambina | Crambus alienellu | Germar & Kaulfuss | P.D.N. Heber | 22-Jul-2007 | Canad | Manitob | Churchil | 10 km E Churchill, Launch Roa | | 58,7599983 | -94 |
| 07PROBE-1075 | 658 | JX008223 | Crambida | Crambina | Crambus alienellu | Germar & Kaulfuss | P.D.N. Heber | 23-Jul-2007 | Canad | Manitob | Churchil | 26 km SE Churchill, Twin Lake | | 58,6300010 | -93,8190002 |
| 07PROBE-1084 | 658 | JX008176 | Crambida | Crambina | Crambus alienellu | Germar & Kaulfuss | P.D.N. Heber | 23-Jul-2007 | Canad | Manitob | Churchil | 26 km SE Churchill, Twin Lake | | 58,6300010 | -93,8190002 |
| 08BBLEP-0155 | 616 | JX008163 | Crambida | Crambina | Crambus alienellu | Germar & Kaulfuss | J.Cossey, N.Jeffery, J.Straka | 10-Jul-200 | Canad | Manitob | Riding Mountai | Riding Mountain National Pa | Clear Spring spruce bo | 50,6790008 | -99,8040008 |
| MDH00203 | 657 | JX008252 | Crambida | Crambina | Crambus alienellu | Germar & Kaulfuss | Daniel Handfiel | 23-Jun-200 | Canad | Quebe | CAN:QC -Saint-Narcic | | Tourbier | 46,5849990 | -73,1979980 |
| MDH002874 | 657 | JX008255 | Crambidae | Crambinae | Crambus alienellus | Germar & Kaulfuss | Daniel Handfield | 02-Jun-2005 | Canada | Quebec | CAN:QC -St-Valerien de Milto | | Tourbiere St-Valerien (petit 6) | 45,53900146 | -72,67099762 |
| MDH002875 | 658 | JX008189 | Crambidae | Crambinae | Crambus alienellus | Germar & Kaulfuss | Daniel Handfield | 02-Jun-2005 | Canada | Quebec | CAN:QC -St-Valerien de Milto | | Tourbiere St-Valerien (petit 6) | 45,53900146 | -72,67099762 |
| MDH002876 | 657 | JX008458 | Crambidae | Crambinae | Crambus alienellus | Germar & Kaulfuss | Daniel Handfield | 02-Jun-2005 | Canada | Quebec | CAN:QC -St-Valerien de Milto | | Tourbiere St-Valerien (petit 6) | 45,53900146 | -72,67099762 |

| | | | | | | | | | | | | | | | |
|---|---|---|---|---|---|---|---|---|---|---|---|---|---|---|---|
| MDH005752 | 658 | JX008462 | Crambidae | Crambinae | Crambus alienellus | Germar & Kaulfuss | D.Handfield | 09-Jul-2008 | Canada | Quebec | Saint-Henri-de-Taillon | Parc de la Pointe-Taillon | | 49,24169922 | -72,42299652 |
| MM03227 | 658 | HM871887 | Crambidae | Crambinae | Crambus alienellus | Germar & Kaulfuss | Marko Mutanen | 29-Jun-2006 | Finland | Oulun laani | Ostrobottnia borealis pars australis | | Kiiminki | 65,07099915 | 25,72500038 |
| MM04226 | 658 | HM872340 | Crambidae | Crambinae | Crambus alienellus | Germar & Kaulfuss | Marko Mutanen | | Finland | Ita-Suomen Laan | Karelia borealis | | Kesaelaht | 61,79299927 | 29,71100044 |
| MM14020 | 658 | HM875841 | Crambidae | Crambinae | Crambus alienellus | Germar & Kaulfuss | Marko Mutanen | | Finland | | Karelia borealis | | Tuupovaara | 62,375 | 30,90500069 |
| TLMF Lep 02839 | 658 | JF860321 | Crambidae | Crambinae | Crambus alienellus | Germar & Kaulfuss | Huemer P. | 09-Jun-2006 | Austria | | Nordtirol, Kaisergebirge | | Walchsee NW - Schwemm N-Teil | 47,65999985 | 12,30200005 |
| TLMF Lep 02840 | 658 | JF860322 | Crambidae | Crambinae | Crambus alienellus | Germar & Kaulfuss | Huemer P. | 09-Jun-2006 | Austria | | Nordtirol, Kaisergebirge | | Walchsee NW - Schwemm N-Teil | 47,65999985 | 12,30200005 |
| MM02212 | 633 | HM871446 | Elachistidae | Depressariinae | Depressaria leucocephala | Snellen | Marko Mutanen, Panu Vaelimaeki | | Finland | | Savonia australis | | Imatra | 61,10800171 | 28,79899979 |
| MM06123 | 658 | HM873056 | Elachistidae | Depressariinae | Depressaria leucocephala | Snellen | Tomi Mutanen | | Finland | | Regio Aboensis | | Salo | 60,26200104 | 23,02499962 |
| MM18133 | 658 | JF854150 | Elachistidae | Depressariinae | Depressaria leucocephala | Snellen | Marko Mutanen | 29-Jul-2010 | Finland | | Ostrobottnia borealis pars australis | | Oulunsalo | 64,99369812 | 25,21969986 |
| TLMF Lep 02724 | 658 | JF860247 | Elachistidae | Depressariinae | Depressaria leucocephala | Snellen | Schmid J. | 28-Jun-2010 | Switzerland | Graubunder | Unterengadin | | Lavin Umgebung | 46,77000046 | 10,11100006 |
| TLMF Lep 02870 | 658 | JF860336 | Elachistidae | Depressariinae | Depressaria leucocephala | Snellen | Burmann K. & Huemer P | 13-Aug-1988 | Austria | Tyrol | Nordtirol | | Steinseehuettenweg/ Zams NW | 47,17599869 | 10,60400009 |
| MM18563 | 658 | JF853361 | Elachistidae | Elachistinae | Elachista elsaella | Traugott-Olsen | Ingvar Svensson | 01-May-1990 | Sweden | | Sm | | Hoegsby | | |
| MM18590 | 658 | JF853374 | Elachistidae | Elachistinae | Elachista elsaella | Traugott-Olsen | Bengt A. Bengtsson | 25-Jun-2010 | Sweden | | Sm | | Lenhovda, Varaskruv | | |
| TLMF Lep 03013 | 658 | JN267144 | Elachistidae | Elachistinae | Elachista elsaella | Traugott-Olsen | Huemer P | 14-Sep-2010 | Italy | South Tyrol | Etschtal | | Montiggl/ Kleiner Prio | 46,42828995 | 11,30000019 |
| MM02650 | 658 | HM871652 | Elachistidae | Elachistinae | Elachista subalbidella | Schläger | Marko Mutanen, Panu Vaelimaeki | | Finland | | Savonia australis | | Imatra | 61,10800171 | 28,79899979 |
| MM02651 | 658 | HM871653 | Elachistidae | Elachistinae | Elachista subalbidella | Schläger | Marko Mutanen, Panu Vaelimaeki | | Finland | | Savonia australis | | Imatra | 61,10800171 | 28,79899979 |
| MM03154 | 658 | HM871842 | Elachistidae | Elachistinae | Elachista subalbidella | Schläger | Marko Mutanen | | Finland | | Ostrobottnia borealis pars australis | | Kiiminki | 65,07099915 | 25,72500038 |
| MM05332 | 572 | HM872775 | Elachistidae | Elachistinae | Elachista subalbidella | Schläger | Marko Mutanen | | Finland | Lansi-Suomen Laani | Regio Aboensis | | Turku | 60,44100189 | 22,20199966 |
| MM05490 | 658 | HM872850 | Elachistidae | Elachistinae | Elachista subalbidella | Schläger | Marko Mutanen | 06-Jun-2007 | Finland | | Ostrobottnia borealis pars australis | | Kiiminki | 65,07099915 | 25,72500038 |
| MM06221 | 658 | HM873125 | Elachistidae | Elachistinae | Elachista subalbidella | Schläger | Marko Mutanen | 19-Jun-2007 | Finland | | Karelia australis | | Joutseno | 61,05500031 | 28,72200012 |
| MM08055 | 658 | HM873753 | Elachistidae | Elachistinae | Elachista subalbidella | Schläger | Marko Mutanen, Panu Vaelimaeki | | Finland | | Lapponia kemensis pars occidentalis | | Kolari | 67,27600098 | 23,75300026 |
| MM09037 | 658 | HM874150 | Elachistidae | Elachistinae | Elachista subalbidella | Schläger | Marko Mutanen, Panu Vaelimaeki | | Finland | | Karelia ladogensis | | Simpele | 61,42300034 | 29,32299995 |
| MM13727 | 658 | HM875756 | Elachistidae | Elachistinae | Elachista subalbidella | Schläger | Marko Mutanen, Panu Vaelimaeki | | Finland | | Regio Aboensis | | Dragsfjaerd | 60,01100159 | 22,49799919 |
| MM14064 | 658 | HM875871 | Elachistidae | Elachistinae | Elachista subalbidella | Schläger | Marko Mutanen | | Finland | | Karelia borealis | | Tuupovaara | 62,375 | 30,90500069 |
| MM18154 | 658 | JF854167 | Elachistidae | Elachistinae | Elachista subalbidella | Schläger | Tomi Mutanen | 19-Jun-2004 | Finland | | Ostrobottnia kajanensis | | Kajaani | 64,26750183 | 27,61899948 |
| TLMF Lep 02843 | 658 | JF860325 | Elachistidae | Elachistinae | Elachista subalbidella | Schläger | Huemer P. | 15-Jun-2010 | Austria | Vorarlberg | | | Sonntag, Buchboden, E Hintergurga | 47,24900055 | 9,991000175 |
| TLMF Lep 02844 | 658 | JF860326 | Elachistidae | Elachistinae | Elachista subalbidella | Schläger | Huemer P. | 15-Jun-2010 | Austria | Vorarlberg | | | Sonntag, Buchboden, E Hintergurga | 47,24900055 | 9,991000175 |
| MM02824 | 658 | HM871721 | Geometridae | Ennominae | Elophos vittaria | Thunberg | Marko Mutanen | | Finland | | Ostrobottnia borealis pars australis | | Kiiminki | 65,07099915 | 25,72500038 |
| MM04136 | 658 | HM386923 | Geometridae | Ennominae | Elophos vittaria | Thunberg | Marko Mutanen | | Finland | | | | Enontekioe | 68,99700165 | 20,74399948 |
| MM14085 | 658 | HM875882 | Geometridae | Ennominae | Elophos vittaria | Thunberg | Marko Mutanen | | Finland | | Karelia borealis | | Tuupovaara | 62,375 | 30,90500069 |
| TLMF Lep 00248 | 648 | HM425757 | Geometridae | Ennominae | Elophos vittaria | Thunberg | Huemer P | 13-Jul-2009 | Austria | Karnten | | | Petzen N, Obere Krischa | 46,5060997 | 14,75699997 |
| TLMF Lep 02417 | 658 | JF859959 | Geometridae | Ennominae | Elophos vittaria | Thunberg | Huemer P | 01-Jul-2010 | Italy | South Tyrol | | | Ritten/ Obergruenwal | 46,59700012 | 11,43900013 |
| TLMF Lep 02482 | 658 | JF860022 | Geometridae | Ennominae | Elophos vittaria | Thunberg | Huemer P | 19-Jul-2010 | Italy | South Tyrol | | | Ritten/ Obergruenwal | 46,59700012 | 11,43900013 |
| TLMF Lep 02725 | 658 | JF860248 | Geometridae | Ennominae | Elophos vittaria | Thunberg | Schmid J. | 22-Jun-2005 | Switzerland | Graubunder | | | Riefawald/ Vals | 46,61800003 | 9,194000244 |
| BC NP 0165 | 658 | JX034674 | Geometridae | Larentiinae | Entephria nobiliaria | Herrich-Schäffer | N. Poel | 20-Jul-2007 | Austria | Salzburg | Hohe Tauern | | Dorfertal, Spinewitro | 47,0890007 | 12,61699963 |
| BC NP 0166 | 575 | JX034671 | Geometridae | Larentiinae | Entephria nobiliaria | Herrich-Schäffer | N. Poel | 20-Jul-2007 | Austria | Salzburg | Hohe Tauern | | Dorfertal, Spinewitro | 47,0890007 | 12,61699963 |
| BC NP 0167 | 609 | JX034638 | Geometridae | Larentiinae | Entephria nobiliaria | Herrich-Schäffer | N. Poel | 20-Jul-2007 | Austria | Salzburg | Hohe Tauern | | Dorfertal, Spinewitro | 47,0890007 | 12,61699963 |
| BC NP 0199 | 658 | JX034698 | Geometridae | Larentiinae | Entephria nobiliaria | Herrich-Schäffer | S. Ortner | 30-Jul-2008 | France | Provence-Alpes-Cote d'Azur | | | Col du Galibier | 45,06000137 | 6,409999847 |
| BC NP 0286 | 658 | JX034604 | Geometridae | Larentiinae | Entephria nobiliaria | Herrich-Schäffer | S. Ortner | 10-Aug-2008 | Austria | Tyrol | | | Soelden, Rettenbachferne | 46,91999817 | 10,94999981 |
| BC ZSM Lep 11366 | 621 | JX034657 | Geometridae | Larentiinae | Entephria nobiliaria | Herrich-Schäffer | Stanislav Gombo | 04-Aug-1997 | Slovenia | Carniola | | | Julijske Alpe, Mangart (ID=688 | | |
| BC ZSM Lep 11367 | 598 | JX034618 | Geometridae | Larentiinae | Entephria nobiliaria | Herrich-Schäffer | Stanislav Gombo | 04-Aug-1997 | Slovenia | Carniola | | | Julijske Alpe, Mangart (ID=688 | | |
| BC ZSM Lep 11368 | 589 | JX034594 | Geometridae | Larentiinae | Entephria nobiliaria | Herrich-Schäffer | Stanislav Gomboc | 02-Aug-2002 | Slovenia | Carniola | | | Julijske Alpe, Kanin, okolica smuia (ID=715) | | |
| BC ZSM Lep 11369 | 659 | JX034601 | Geometridae | Larentiinae | Entephria nobiliaria | Herrich-Schäffer | Stanislav Gomboc | 27-Jul-2006 | Austria | Karnten | | | Kaernten, Grossglockner Gebiet, Wallackhaus (ID=9s589 | | |
| BC ZSM Lep 11370 | 659 | JX034572 | Geometridae | Larentiinae | Entephria nobiliaria | Herrich-Schäffer | Stanislav Gomboc | 27-Jul-2006 | Austria | Karnten | | | Kaernten, Grossglockner Gebiet, Wallackhaus (ID=9s589 | | |
| BC ZSM Lep 21967 | 656 | JX034599 | Geometridae | Larentiinae | Entephria nobiliaria | Herrich-Schäffer | N.Zahm | 28-Jul-2008 | Italy | Abruzzi | | Maiella | Valle Cannella | 42,09019852 | 14,09020042 |
| BC ZSM Lep 26465 | 658 | GU655468 | Geometridae | Larentiinae | Entephria nobiliaria | Herrich-Schäffer | K. Cerny | 23-Aug-2004 | Austria | Tyrol | | Serfaus | Kamperdel | 47,03850174 | 10,5692997 |
| BC ZSM Lep 31853 | 658 | HQ957245 | Geometridae | Larentiinae | Entephria nobiliaria | Herrich-Schäffer | Dr. T. Gruenewald | 16-Aug-2002 | France | Provence-Alpes-Cote d'Azur | Hautes Alpes | | Col de Lautaret | 45,03499985 | 6,40500021 |
| BC ZSM Lep 33964 | 658 | HM903305 | Geometridae | Larentiinae | Entephria nobiliaria | Herrich-Schäffer | H. Deutsch | 02-Sep-2006 | Austria | | Osttirol | Lienzer Dolomiten | Lavanter Forchach | 46,79550171 | 12,83990002 |
| BC ZSM Lep 33965 | 658 | HM903306 | Geometridae | Larentiinae | Entephria nobiliaria | Herrich-Schäffer | H. Deutsch | 20-Aug-2009 | Austria | | Osttirol | Lienzer Dolomiten | Lavanter Alp | 46,78720093 | 12,80700016 |
| BC ZSM Lep 33966 | 658 | HM903307 | Geometridae | Larentiinae | Entephria nobiliaria | Herrich-Schäffer | H. Deutsch | 11-Aug-2004 | Austria | Karnten | | NP Hohe Tauern | Hochtor | | |
| BC ZSM Lep 34401 | 658 | HQ957855 | Geometridae | Larentiinae | Entephria nobiliaria | Herrich-Schäffer | P. Skou | 25-Jul-2006 | Italy | Piedmont | | | Colle di Lombarda | 44,19919968 | 7,149000168 |
| MM18700 | 658 | JX034689 | Geometridae | Larentiinae | Entephria nobiliaria | Herrich-Schäffer | Panu Vaelimaeki | 11-Jul-2003 | Finland | | Lapponia enontekiensis | | Enontekioe | 68,99659729 | 20,74370003 |
| MM18701 | 658 | JN279420 | Geometridae | Larentiinae | Entephria nobiliaria | Herrich-Schäffer | Panu Vaelimaeki | 15-Jul-2001 | Finland | | Lapponia enontekiensis | | Enontekioe | 69,04560089 | 20,85540009 |
| TLMF Lep 00368 | 658 | HM425858 | Geometridae | Larentiinae | Entephria nobiliaria | Herrich-Schäffer | Huemer P. | 01-Aug-2009 | Austria | Karnten | | | Mallnitz, Doesental, Arthur-v.-Schmid-Haus W | 46,98939896 | 13,26690006 |
| TLMF Lep 02028 | 658 | HQ968437 | Geometridae | Larentiinae | Entephria nobiliaria | Herrich-Schäffer | Huemer P | 12-Jul-2010 | Italy | Lazio | Rieti | | Monte Terminillo N | 42,48329926 | 13 |
| TLMF Lep 02726 | 658 | JF860249 | Geometridae | Larentiinae | Entephria nobiliaria | Herrich-Schäffer | Schmid J. | 10-Jun-2007 | Switzerland | Graubunder | Unterengadin | | Ardez | 46,77399826 | 10,20100021 |
| MM06048 | 658 | HM873018 | Tortricidae | Olethreutinae | Epinotia mercuriana | Frölich | Tomi Mutanen | 09-Aug-2007 | Finland | | Lapponia enontekiensis | | Enontekioe | | |
| MM14635 | 658 | HM876195 | Tortricidae | Olethreutinae | Epinotia mercuriana | Frölich | Marko Mutanen | | Finland | | Lapponia enontekiensis | | Enontekioe | 69,06300354 | 21,10199928 |
| MM18327 | 658 | JX034694 | Tortricidae | Olethreutinae | Epinotia mercuriana | Frölich | Tomi Mutanen, Salla Mutanen | 09-Aug-2007 | Finland | | Lapponia enontekiensis | | Enontekioe | 68,90740204 | 20,7689991 |
| TLMF Lep 00121 | 407 | JX034700 | Tortricidae | Olethreutinae | Epinotia mercuriana | Frölich | Huemer P. | 03-Aug-2008 | Italy | Piedmont | Cuneo | Colle Fauniera Umgebung- Demonte NW | | 44,38600159 | 7,122000217 |
| TLMF Lep 00597 | 658 | HM381437 | Tortricidae | Olethreutinae | Epinotia mercuriana | Frölich | Huemer P. | 20-Jul-2009 | Italy | | Belluno | | Passo di Valparola E - Passo Falzarego | 46,5223999 | 12,00699997 |
| TLMF Lep 00789 | 658 | HM426135 | Tortricidae | Olethreutinae | Epinotia mercuriana | Frölich | Huemer P. | 01-Aug-2009 | Austria | Karnten | | | Mallnitz, Doesental, Arthur-v.-Schmid-Haus W | 46,98939896 | 13,26690006 |
| TLMF Lep 01733 | 658 | HQ968937 | Tortricidae | Olethreutinae | Epinotia mercuriana | Frölich | Huemer P | 09-Aug-2010 | Slovenia | | | | Veliki Mangar | 46,43849945 | 13,63529968 |
| TLMF Lep 01734 | 658 | HQ968938 | Tortricidae | Olethreutinae | Epinotia mercuriana | Frölich | Huemer P | 09-Aug-2010 | Slovenia | | | | Veliki Mangar | 46,43849945 | 13,63529968 |
| TLMF Lep 01738 | 658 | HQ968942 | Tortricidae | Olethreutinae | Epinotia mercuriana | Frölich | Huemer P | 09-Aug-2010 | Slovenia | | | | Veliki Mangar | 46,43849945 | 13,63529968 |
| MM03905 | 658 | HM872218 | Tortricidae | Olethreutinae | Epinotia nemorivaga | Tengström | Marko Mutanen | 27-Jun-2006 | Finland | Oulun laani | Ostrobottnia borealis pars australis | | Pudasjaervi | 65,38999939 | 26,91399956 |
| MM04124 | 625 | HM386913 | Tortricidae | Olethreutinae | Epinotia nemorivaga | Tengström | Marko Mutanen | | Finland | | | | Enontekioe | 68,99700165 | 20,74399948 |
| MM04173 | 658 | HM386960 | Tortricidae | Olethreutinae | Epinotia nemorivaga | Tengström | Marko Mutanen | | Finland | | | | Enontekioe | 68,99700165 | 20,74399948 |

| | | | | | | | | | | | | | | | | |
|---|---|---|---|---|---|---|---|---|---|---|---|---|---|---|---|---|
| MM09973 | 658 | HM874510 | Tortricidae | Olethreutinae | Epinotia nemorivaga | Tengström | Marko Mutanen | 16-Jun-2008 | Finland | | Karelia ladogensis | | Parikkala | 61,61700058 | 29,54500008 | |
| TLMF Lep 02728 | 658 | JF860250 | Tortricidae | Olethreutinae | Epinotia nemorivaga | Tengström | Schmid J. | 13-Jul-2009 | Switzerland | Graubunden | | | Bernina/ Hospiz | 46,4109993 | 10,02299976 | genitalia on card boa |
| BC ZSM Lep 25438 | 658 | GU655018 | Nymphalidae | Satyrinae | Erebia medusa | Denis & Schiffermüller | Wolfgang Schacht | 20-May-2009 | Germany | Bavaria | Oberbayern | Fuerstenfeldbruck | Jexhof, Hackenwiesen | 48,1129989 | 11,21399975 | |
| BC ZSM Lep 30695 | 658 | HM393204 | Nymphalidae | Satyrinae | Erebia medusa | Denis & Schiffermüller | R. Sturm | 10-Jun-2004 | Germany | Bavaria | Niederbayern | Straubing-Boger | Bayer. Wald, Gandsberg | 48,9459991 | 12,85499954 | |
| BC ZSM Lep 30696 | 658 | HM393205 | Nymphalidae | Satyrinae | Erebia medusa | Denis & Schiffermüller | R. Sturm | 25-May-2001 | Germany | Bavaria | Schwaben | | Rott a. Lech | 47,90800095 | 10,93599987 | |
| BC ZSM Lep 32177 | 658 | JF415701 | Nymphalidae | Satyrinae | Erebia medusa | Denis & Schiffermüller | M. Seizmair | 20-May-2009 | Germany | Bavaria | Oberbayern | Bad Toelz-Wolfratshausen | Lindenrain, Geretsried | 47,8362999 | 11,48419952 | |
| BC ZSM Lep 32181 | 658 | JF415700 | Nymphalidae | Satyrinae | Erebia medusa | Denis & Schiffermüller | M. Seizmair | 19-May-2009 | Germany | Bavaria | Oberbayern | Munich | Isarauen, Fischerhaeuse | 48,25130081 | 11,69019985 | |
| BC ZSM Lep 50482 | 658 | JX034611 | Nymphalidae | Satyrinae | Erebia medusa | Denis & Schiffermüller | Dr. Andreas H. Segerer | 22-May-2010 | Germany | Bavaria | Oberpfalz | Regensburg | Nittendorf | 49,03139877 | 11,96259975 | |
| TLMF Lep 01115 | 658 | HQ968478 | Nymphalidae | Satyrinae | Erebia medusa | Denis & Schiffermüller | Aistleitner U | 14-Jun-2009 | Switzerland | Graubunden | | | Waltensburg/Vuorz, Spinatsch | 46,84000015 | 9,361000061 | |
| TLMF Lep 01151 | 658 | HQ968511 | Nymphalidae | Satyrinae | Erebia medusa | Denis & Schiffermüller | Mayr T. | 23-May-2009 | Switzerland | Graubunden | | | Chur, Pagig | 46,83300018 | 9,633000374 | |
| TLMF Lep 02398 | 658 | JF859942 | Nymphalidae | Satyrinae | Erebia medusa | Denis & Schiffermüller | Huemer P | 01-Jul-2010 | Italy | South Tyrol | | | Ritten/ Obergruenwal | 46,59700012 | 11,43900013 | |
| BC ZSM Lep 21999 | 658 | GU686974 | Nymphalidae | Satyrinae | Erebia pandrose | Borkhausen | U. Buchsbaum | 17-Jul-1999 | Germany | Bavaria | Oberbayern | | Wettersteingebirge, Kreuzeck-Stuibensee | 47,45280075 | 11,0685997 | |
| BC ZSM Lep 50329 | 658 | JX034616 | Nymphalidae | Satyrinae | Erebia pandrose | Borkhausen | Alfred Haslberge | 04-Aug-2004 | Germany | Bavaria | Schwaben | Oberallgaeu | Oberstdorf, Nebelhorn/Kobla | 47,4239006 | 10,36320019 | |
| MM00091 | 658 | HM396370 | Nymphalidae | Satyrinae | Erebia pandrose | Borkhausen | Marko Mutanen | | Finland | | | | Enontekioe | 68,99700165 | 20,74399948 | |
| MM06305 | 658 | HM873189 | Nymphalidae | Satyrinae | Erebia pandrose | Borkhausen | Marko Mutanen, Anttoni Mutanen, Nestori Mutanen | 28-Jun-2006 | Finland | | Lapponia inarensis | | Inari | 68,4469986 | 27,41399956 | |
| MM14572 | 658 | HM876171 | Nymphalidae | Satyrinae | Erebia pandrose | Borkhausen | Marko Mutanen | | Finland | | Lapponia enontekiensis | | Enontekioe | 69,06300354 | 21,10199928 | |
| TLMF Lep 00344 | 658 | HM425836 | Nymphalidae | Satyrinae | Erebia pandrose | Borkhausen | Huemer P | 20-Jul-2009 | Italy | | Belluno | | Passo di Valparola E | 46,52550125 | 11,99979973 | |
| TLMF Lep 00407 | 658 | HM425891 | Nymphalidae | Satyrinae | Erebia pandrose | Borkhausen | Huemer P. | 23-Jul-2009 | Italy | | Cuneo | | Colle Fauniera Umgebung/ Demonte NW | 44,38560104 | 7,121940136 | |
| TLMF Lep 01172 | 658 | HQ968528 | Nymphalidae | Satyrinae | Erebia pandrose | Borkhausen | Mayr T. | 20-Jun-2009 | Switzerland | Graubunden | | | Val Bever, Alp Suvretta | 46,54999924 | 9,782999992 | |
| TLMF Lep 01836 | 658 | HQ968299 | Nymphalidae | Satyrinae | Erebia pandrose | Borkhausen | Mayr T. | 20-Jun-2009 | Switzerland | Graubunden | | | Val Bever, Alp Suvretta | 46,54999924 | 9,783329964 | |
| TLMF Lep 05519 | 658 | JX034634 | Nymphalidae | Satyrinae | Erebia pandrose | Borkhausen | Huemer P. & Tarmann G. M. | 28-Jul-2011 | Macedonia | | | | Mavrovo NP, Korab, summit ridge | 41,78900146 | 20,54700089 | |
| TLMF Lep 05520 | 658 | JX034650 | Nymphalidae | Satyrinae | Erebia pandrose | Borkhausen | Huemer P. & Tarmann G. M. | 28-Jul-2011 | Macedonia | | | | Mavrovo NP, Korab, summit ridge | 41,78900146 | 20,54700089 | |
| MM10583 | 658 | HQ570388 | Nymphalidae | Satyrinae | Erebia polaris | Staudinger | Marko Mutanen | | Finland | | Lapponia inarensis | | Utsjok | 69,28600311 | 25,73399925 | |
| MM10584 | 658 | HQ570389 | Nymphalidae | Satyrinae | Erebia polaris | Staudinger | Marko Mutanen | | Finland | | Lapponia inarensis | | Utsjok | 69,28600311 | 25,73399925 | |
| MM17178 | 658 | JF853668 | Nymphalidae | Satyrinae | Erebia polaris | Staudinger | Marko Mutanen | 27-Jun-2002 | Finland | | Lapponia inarensis | | Utsjok | 69,28600311 | 25,73399925 | |
| MM00717 | 658 | HM871151 | Crambidae | Scopariinae | Eudonia sudetica | Zeller | Tomi Mutanen | 22-Jul-2006 | Finland | | Ostrobottnia borealis pars borealis | | Ranua | 65,88200378 | 26,34199905 | |
| MM04135 | 658 | HM386922 | Crambidae | Scopariinae | Eudonia sudetica | Zeller | Marko Mutanen | | Finland | | | | Enontekioe | 68,99700165 | 20,74399948 | |
| MM08041 | 658 | HM873745 | Crambidae | Scopariinae | Eudonia sudetica | Zeller | Marko Mutanen, Panu Vaelimaeki | | Finland | | Lapponia kemensis pars occidentalis | | Kolari | 67,27600098 | 23,75300026 | |
| MM14647 | 658 | HM876201 | Crambidae | Scopariinae | Eudonia sudetica | Zeller | Marko Mutanen, Nestori Mutanen, Anttoni Mutanen | 28-Jul-2009 | Finland | | Ostrobottnia borealis pars australis | | Kiiminki | 65,07099915 | 25,72500038 | |
| TLMF Lep 00128 | 658 | GU689176 | Crambidae | Scopariinae | Eudonia sudetica | Zeller | Huemer P. | 03-Aug-2008 | Italy | Piedmont | Cuneo | Colle Fauniera Umgebung- Demonte NW | | 44,38600159 | 7,122000217 | |
| TLMF Lep 00129 | 658 | GU689177 | Crambidae | Scopariinae | Eudonia sudetica | Zeller | Huemer P. | 03-Aug-2008 | Italy | Piedmont | Cuneo | Colle Fauniera Umgebung- Demonte NW | | 44,38600159 | 7,122000217 | |
| TLMF Lep 00543 | 658 | HM381386 | Crambidae | Scopariinae | Eudonia sudetica | Zeller | Huemer P. | 13-Jul-2009 | Austria | Karnten | | | Petzen N, Umg. Gh. Siebenhuetten | 46,51819992 | 14,77350044 | |
| TLMF Lep 00619 | 658 | HM381452 | Crambidae | Scopariinae | Eudonia sudetica | Zeller | Tarmann G. M. | 22-Jul-2009 | Italy | | Belluno | | Passo di Valparola SW, Mt. Castello | 46,52389908 | 11,97669983 | |
| TLMF Lep 00639 | 658 | HM426007 | Crambidae | Scopariinae | Eudonia sudetica | Zeller | Huemer P. | 27-Jul-2009 | France | Provence-Alpes-Cote d'Azur | | | N Col de la Boucharde/ Col de la Cayolle N | 44,28329849 | 6,743330002 | |
| TLMF Lep 01321 | 307 | JX034575 | Crambidae | Scopariinae | Eudonia sudetica | Zeller | Nel J. | 04-Aug-2002 | France | Rhone-Alpes | | | Port de la Neige, Iseran | | | 14394JN |
| TLMF Lep 01524 | 658 | HQ968736 | Crambidae | Scopariinae | Eudonia sudetica | Zeller | Huemer P. | 14-Jul-2010 | Italy | Abruzzi | L`Aquila | | NP Gran Sasso, Campo Imperatore, ex Miniera di Lignite | 42,42670059 | 13,71329975 | |
| TLMF Lep 01525 | 658 | HQ968737 | Crambidae | Scopariinae | Eudonia sudetica | Zeller | Huemer P. | 14-Jul-2010 | Italy | Abruzzi | L`Aquila | | NP Gran Sasso, Campo Imperatore, ex Miniera di Lignite | 42,42670059 | 13,71329975 | |
| TLMF Lep 01730 | 658 | HQ968934 | Crambidae | Scopariinae | Eudonia sudetica | Zeller | Huemer P | 09-Aug-2010 | Slovenia | | | | Veliki Mangar | 46,43849945 | 13,63529968 | |
| MM03145 | 658 | HM871840 | Yponomeutidae | Yponomeutinae | Euhyponomeutoides albithoracellus | Gaj | Marko Mutanen | | Finland | | Ostrobottnia borealis pars australis | | Kiiminki | 65,07099915 | 25,72500038 | |
| MM08051 | 641 | HM872911 | Yponomeutidae | Yponomeutinae | Euhyponomeutoides albithoracellus | Gaj | Marko Mutanen, Panu Vaelimaeki | | Finland | | Lapponia kemensis pars occidentalis | | Kolari | 67,27600098 | 23,75300026 | |
| MM13587 | 658 | HM875668 | Yponomeutidae | Yponomeutinae | Euhyponomeutoides albithoracellu | Gaj | Marko Mutanen, Panu Vaelimael | | Finland | | Regio Aboensis | | Dragsfjaerd | 60,01100159 | 22,49799919 | |
| TLMF Lep 02732 | 627 | JF860253 | Yponomeutidae | Yponomeutinae | Euhyponomeutoides albithoracellus | Gaj | Schmid J. | 30-Jun-2008 | Switzerland | Graubunden | Sesvenna | | Santa Maria in Muestair, Costeras | 46,60699844 | 10,42399979 | |
| TLMF Lep 02868 | 658 | JF860334 | Yponomeutidae | Yponomeutinae | Euhyponomeutoides albithoracellu | Gaj | Huemer P | 23-Jun-1993 | Austria | Tyrol | Osttirol, Virgenta | | Bichl/ Praegraten NW | 47,02000046 | 12,36299992 | |
| TLMF Lep 02869 | 658 | JF860335 | Yponomeutidae | Yponomeutinae | Euhyponomeutoides albithoracellu | Gaj | Huemer P | 23-Jun-1993 | Austria | Tyrol | Osttirol, Virgenta | | Bichl/ Praegraten NW | 47,02000046 | 12,36299992 | |
| 08BBLEP-02354 | 658 | JX008100 | Geometridae | Larentiinae | Eupithecia cretaceata | Packard | J.Straka | 21-Jul-2008 | Canada | Alberta | Waterton | Waterton Lakes Nat. Park - Townsite Campground | Riverside/lakeside meadow | 49,04499817 | -113,913002 | |
| 08BBLEP-02423 | 658 | JX008109 | Geometridae | Larentiinae | Eupithecia cretaceata | Packard | J.Straka,J.Cossey | 26-Jul-2008 | Canada | Alberta | Waterton | Waterton Lakes Nat. Park - Maskinonge Lake | Marsh/meadow | 49,11000061 | -113,8399963 | |
| 08BBLEP-02565 | 656 | JX008388 | Geometridae | Larentiinae | Eupithecia cretaceata | Packard | J.Straka,J.Cossey | 26-Jul-2008 | Canada | Alberta | Waterton | Waterton Lakes Nat. Park - Blakiston Creek | Fast-flowing, rocky creekside | 49,07799911 | -113,8820038 | |
| 08BBLEP-03494 | 658 | JX008145 | Geometridae | Larentiinae | Eupithecia cretaceata | Packard | J.Straka,J.Cossey | 26-Jul-2008 | Canada | Alberta | Waterton | Waterton Lakes Nat. Park - Blakiston Creek | Fast-flowing, rocky creekside | 49,07799911 | -113,8820038 | |
| 08BBLEP-04512 | 658 | JX008167 | Geometridae | Larentiinae | Eupithecia cretaceata | Packard | J.Straka,J.Cossey | 31-Jul-2008 | Canada | Alberta | Banff | Banff Nat. Park - Cave and Basin Area | Spring-fed marsh/meadow | 51,17100143 | -115,5879974 | |
| 08BBLEP-04906 | 658 | JX008275 | Geometridae | Larentiinae | Eupithecia cretaceata | Packard | | 06-Aug-2008 | Canada | Alberta | Waterton | Waterton Lakes Nat. Park - Blakiston Creek | Rocky creekside | 49,07799911 | -113,8820038 | |
| 08BBLEP-04913 | 658 | JX008365 | Geometridae | Larentiinae | Eupithecia cretaceata | Packard | | 06-Aug-2008 | Canada | Alberta | Waterton | Waterton Lakes Nat. Park - Blakiston Creek | Rocky creekside | 49,07799911 | -113,8820038 | |
| 08BBLEP-05001 | 658 | JX008181 | Geometridae | Larentiinae | Eupithecia cretaceata | Packard | J.Straka,J.Cossey | 07-Aug-2008 | Canada | Alberta | Waterton | Waterton Lakes Nat. Park - Crandell campground | Grassland/creekside forest | 49,09899902 | -113,9509964 | |
| 08BBLEP-05053 | 658 | JX008117 | Geometridae | Larentiinae | Eupithecia cretaceata | Packard | J.Straka,J.Cossey | 07-Aug-2008 | Canada | Alberta | Waterton | Waterton Lakes Nat. Park - Crandell campground | Grassland/creekside forest | 49,09899902 | -113,9509964 | |
| 10BBCLP-1070 | 658 | JF842083 | Geometridae | Larentiinae | Eupithecia cretaceata | Packard | BIObus 2010 | 20-Jul-2010 | Canada | British Columbia | Yoho NP | Kicking Horse Cmpgrd | Forested cmpgrd | 51,42399979 | -116,4290009 | |
| 10BBCLP-1076 | 658 | JF842089 | Geometridae | Larentiinae | Eupithecia cretaceata | Packard | BIObus 2010 | 15-Jul-2010 | Canada | British Columbia | Glacier NP | Hemlock Grove Trl. | Grassy area near hemlock forest | 51,24200058 | -117,6490021 | |
| TLMF Lep 00209 | 658 | GU689138 | Geometridae | Larentiinae | Eupithecia cretaceata | Packard | Huemer P. | 12-Jun-2007 | Italy | Friuli-Venezia Giulia | Udine, Val Venzonassa | Jof Ungarina SW-Seite- Malga Confin W | | 46,34400177 | 13,20899963 | |
| TLMF Lep 00210 | 574 | GU689132 | Geometridae | Larentiinae | Eupithecia cretaceata | Packard | Huemer P. | 12-Jun-2007 | Italy | Friuli-Venezia Giulia | Udine, Val Venzonassa | Jof Ungarina SW-Seite- Malga Confin W | | 46,34400177 | 13,20899963 | |
| TLMF Lep 00234 | 658 | HM425743 | Geometridae | Larentiinae | Eupithecia cretaceata | Packard | Huemer P. | 13-Jul-2009 | Austria | Karnten | | | Petzen N, Umg. Gh. Siebenhuetten | 46,51819992 | 14,77350044 | |

| | | | | | | | | | | | | | | | | |
|---|---|---|---|---|---|---|---|---|---|---|---|---|---|---|---|---|
| TLMF Lep 00370 | 658 | HM425860 | Geometridae | Larentiinae | Eupithecia cretaceata | Packard | Huemer P. | 01-Aug-2009 | Austria | Karnten | | | Mallnitz, Doesental, Arthur-v.-Schmid-Haus W | 46,98939896 | 13,26690006 | |
| TLMF Lep 00396 | 658 | HM425882 | Geometridae | Larentiinae | Eupithecia cretaceata | Packard | Huemer P. | 26-Jul-2009 | France | Provence-Alpes-Cote d'Azu | | | N Col de la Boucharde/ Col de la Cayolle N | 44,28329849 | 6,743330002 | |
| TLMF Lep 05579 | 658 | JX034656 | Geometridae | Larentiinae | Eupithecia cretaceata | Packard | Huemer P. & Tarmann G. M. | 28-Jul-2011 | Macedonia | | | | Mavrovo NP, Korab, Korabska jezero, Kobilino pole | 41,77799988 | 20,58200073 | |
| TLMF Lep 05580 | 658 | JX034701 | Geometridae | Larentiinae | Eupithecia cretaceata | Packard | Huemer P. & Tarmann G. M. | 28-Jul-2011 | Macedonia | | | | Mavrovo NP, Korab, Korabska jezero, Kobilino pole | 41,77799988 | 20,58200073 | |
| MM00399 | 609 | JX034654 | Elachistidae | Depressariinae | Exaeretia ciniflonella | Lienig & Zeller | Marko Mutanen | 03-May-2006 | Finland | | Ostrobottnia borealis pars australis | | Kiiminki | 65,07099915 | 25,72500038 | |
| MM00400 | 670 | JX034637 | Elachistidae | Depressariinae | Exaeretia ciniflonella | Lienig & Zeller | Marko Mutanen | 30-Apr-2006 | Finland | | Ostrobottnia borealis pars australis | | Kiiminki | 65,07099915 | 25,72500038 | |
| MM00427 | 658 | HM871014 | Elachistidae | Depressariinae | Exaeretia ciniflonella | Lienig & Zeller | Marko Mutanen | 03-May-2006 | Finland | | Ostrobottnia borealis pars australis | | Kiiminki | 65,07099915 | 25,72500038 | |
| MM02221 | 571 | HM87145( | Elachistidae | Depressariinae | Exaeretia ciniflonella | Lienig & Zeller | Marko Mutanen, Panu Vaelimael | | Finland | | Savonia australis | | Imatra | 61,10800171 | 28,79899979 | |
| MM05297 | 658 | HM872753 | Elachistidae | Depressariinae | Exaeretia ciniflonella | Lienig & Zeller | Marko Mutanen | | Finland | Lansi-Suomen Laani | Regio Aboensis | | Turku | 60,44100189 | 22,20199966 | |
| TLMF Lep 0187 | 407 | JX034699 | Elachistidae | Depressariinae | Exaeretia ciniflonella | Lienig & Zeller | Burmann K. | 21-Mar-1981 | Austria | Tyrol | Nordtirol, Wippta | | Ahrnberg - Innsbruck S | 47,22060013 | 11,3980999 | |
| TLMF Lep 0479 | 658 | JX034588 | Elachistidae | Depressariinae | Exaeretia ciniflonella | Lienig & Zeller | Sonderegger P. | 06-Sep-2006 | Switzerland | Valais | | | Saviese | 46,29719925 | 8,619689941 | |
| MM1250 | 658 | HM875301 | Hepialidae | | Gazoryctra ganna | Hübner | Tomi Mutanen | | Finland | | Tavastia australi | | Kuru | 61,96900177 | 23,35700035 | |
| MM1250 | 658 | HM875302 | Hepialidae | | Gazoryctra ganna | Hübner | Tomi Mutanen | | Finland | | Tavastia australi | | Kuru | 61,96900177 | 23,35700035 | |
| MM17517 | 658 | JX034573 | Hepialidae | | Gazoryctra ganna | Hübner | Juhani Itaemies | | Finland | | Ostrobottnia borealis pars australis | | Muhos | 64,81300354 | 25,98999977 | |
| TLMF Lep 0181 | 658 | JF859593 | Hepialidae | | Gazoryctra ganna | Hübner | Mayr T. | 07-Aug-1998 | Austria | | | | Hahntennjoch (Lechtaler A. | 47,2799987 | 10,64999962 | |
| TLMF Lep 0181 | 658 | HQ96828( | Hepialidae | | Gazoryctra ganna | Hübner | Mayr T. | 07-Aug-1998 | Austria | | | | Hahntennjoch (Lechtaler A. | 47,2799987 | 10,64999962 | |
| TLMF Lep 03061 | 658 | JN307292 | Hepialidae | | Gazoryctra ganna | Hübner | Huemer P. | 28-Aug-2008 | Austria | Vorarlberg | | | Umg. Goeppinger Huette, Gamsboden | 47,21749878 | 10,03470039 | |
| 06-PROBE-2811 | 650 | JX008147 | Crambidae | Scopariinae | Gesneria centuriella | Denis & Schiffermüller | E.Stur, T.Ekrem | 19-Aug-2006 | Canada | Manitoba | Churchill | 26 km SE Churchill, Twin Lakes burn site | | 58,61700058 | -93,81199646 | PYR566 M |
| 06-PROBE-2812 | 650 | JX008239 | Crambidae | Scopariinae | Gesneria centuriella | Denis & Schiffermüller | E.Stur, T.Ekrem | 19-Aug-2006 | Canada | Manitoba | Churchill | 26 km SE Churchill, Twin Lakes burn site | | 58,61700058 | -93,81199646 | |
| 06-PROBE-2813 | 650 | JX008214 | Crambidae | Scopariinae | Gesneria centuriella | Denis & Schiffermüller | E.Stur, T.Ekrem | 19-Aug-2006 | Canada | Manitoba | Churchill | 26 km SE Churchill, Twin Lakes burn site | | 58,61700058 | -93,81199646 | |
| 07PROBE-1027 | 658 | JX008244 | Crambidae | Scopariinae | Gesneria centuriella | Denis & Schiffermüller | P.D.N. Hebert | 21-Jul-2007 | Canada | Manitoba | Churchill | 22 km E Churchill, CNSC | | 58,7379989 | -93,81900024 | |
| 07WNP-10794 | 658 | JX008143 | Crambidae | Scopariinae | Gesneria centuriella | Denis & Schiffermüller | J.McGowan | 27-Jul-2007 | Canada | Manitoba | Churchill | 22 km E Churchill, CNSC | | 58,72999954 | -93,8199996 | |
| 08BBLEP-00252 | 658 | JX008471 | Crambidae | Scopariinae | Gesneria centuriella | Denis & Schiffermüller | J.Cossey,J.Straka,S.McCubbin,J.Sones,N.Jeffery | 30-Jun-2008 | Canada | Ontario | Pukaskwa | Pukaskwa National Park | Park road entrance | 48,59199905 | -86,28900146 | |
| 08BBLEP-0036 | 622 | JX008120 | Crambidae | Scopariinae | Gesneria centuriella | Denis & Schiffermüller | N.Jeffery,C.Carr,M.Brummel | 29-Jun-2008 | Canada | Ontario | Pukaskwa | Pukaskwa National Par | camp site evergreen fores | 48,5900001 | -86,2900009 | |
| 08BBLEP-0067 | 658 | JX008238 | Crambidae | Scopariinae | Gesneria centuriella | Denis & Schiffermüller | J.Sones,S.McCubbin,L.Wallace | 02-Jul-2008 | Canada | Ontario | Pukaskwa | Pukaskwa National Par | Visitor center parking lo | 48,5900001 | -86,2900009 | |
| 08BBLEP-02289 | 658 | JX008485 | Crambidae | Scopariinae | Gesneria centuriella | Denis & Schiffermüller | J.Straka,J.Cossey | 26-Jul-2008 | Canada | Alberta | Waterton | Waterton Lakes NP - Blakiston Creek | Fast-flowing, rocky creekside | 49,07799911 | -113,8820038 | |
| 08BBLEP-02527 | 653 | JX008341 | Crambidae | Scopariinae | Gesneria centuriella | Denis & Schiffermüller | J.Straka,J.Cossey | 28-Jul-2008 | Canada | Alberta | Banff | Banff Nat. Park - Tunnel Mountain Campgrounc | Meadow/pine forest border | 51,19300079 | -115,5329971 | |
| 08BBLEP-02539 | 658 | JX008240 | Crambidae | Scopariinae | Gesneria centuriella | Denis & Schiffermüller | J.Straka,J.Cossey | 28-Jul-2008 | Canada | Alberta | Banff | Banff Nat. Park - Tunnel Mountain Campgrounc | Meadow/pine forest border | 51,19300079 | -115,5329971 | |
| 08BBLEP-02545 | 658 | JX008360 | Crambidae | Scopariinae | Gesneria centuriella | Denis & Schiffermüller | J.Straka,J.Cossey | 28-Jul-2008 | Canada | Alberta | Banff | Banff Nat. Park - Tunnel Mountain Campgrounc | Meadow/pine forest border | 51,19300079 | -115,5329971 | |
| 08BBLEP-02546 | 658 | JX008384 | Crambidae | Scopariinae | Gesneria centuriella | Denis & Schiffermüller | J.Straka,J.Cossey | 28-Jul-2008 | Canada | Alberta | Banff | Banff Nat. Park - Tunnel Mountain Campgrounc | Meadow/pine forest border | 51,19300079 | -115,5329971 | |
| 08BBLEP-02553 | 658 | JX008106 | Crambidae | Scopariinae | Gesneria centuriella | Denis & Schiffermüller | J.Straka,J.Cossey | 28-Jul-2008 | Canada | Alberta | Banff | Banff Nat. Park - Tunnel Mountain Campgrounc | Meadow/pine forest border | 51,19300079 | -115,5329971 | |
| 08BBLEP-02554 | 651 | JX008153 | Crambidae | Scopariinae | Gesneria centuriella | Denis & Schiffermüller | J.Straka,J.Cossey | 28-Jul-2008 | Canada | Alberta | Banff | Banff Nat. Park - Tunnel Mountain Campgrounc | Meadow/pine forest border | 51,19300079 | -115,5329971 | |
| 08BBLEP-02559 | 653 | JX008164 | Crambidae | Scopariinae | Gesneria centuriella | Denis & Schiffermüller | J.Straka,J.Cossey | 28-Jul-2008 | Canada | Alberta | Banff | Banff Nat. Park - Tunnel Mountain Campgrounc | Meadow/pine forest border | 51,19300079 | -115,5329971 | |
| 08BBLEP-02568 | 658 | JX008209 | Crambidae | Scopariinae | Gesneria centuriella | Denis & Schiffermüller | J.Straka,J.Cossey | 26-Jul-2008 | Canada | Alberta | Waterton | Waterton Lakes Nat. Park - Blakiston Creek | Fast-flowing, rocky creekside | 49,07799911 | -113,8820038 | |
| 08BBLEP-03412 | 658 | JX008130 | Crambidae | Scopariinae | Gesneria centuriella | Denis & Schiffermüller | J.Straka,J.Cossey | 28-Jul-2008 | Canada | Alberta | Banff | Banff Nat. Park - Tunnel Mountain Campgrounc | Meadow/pine forest border | 51,19300079 | -115,5329971 | |
| 08BBLEP-03413 | 638 | JX008283 | Crambidae | Scopariinae | Gesneria centuriella | Denis & Schiffermüller | J.Straka,J.Cossey | 28-Jul-2008 | Canada | Alberta | Banff | Banff Nat. Park - Tunnel Mountain Campgrounc | Meadow/pine forest border | 51,19300079 | -115,5329971 | |
| 08BBLEP-03418 | 640 | JX008329 | Crambidae | Scopariinae | Gesneria centuriella | Denis & Schiffermüller | J.Straka,J.Cossey | 28-Jul-2008 | Canada | Alberta | Banff | Banff Nat. Park - Tunnel Mountain Campgrounc | Meadow/pine forest border | 51,19300079 | -115,5329971 | |
| 08BBLEP-03646 | 658 | JX008432 | Crambidae | Scopariinae | Gesneria centuriella | Denis & Schiffermüller | J.Straka | 21-Jul-2008 | Canada | Alberta | Waterton | Waterton Lakes Nat. Park - Townsite Campgrounc | Riverside/lakeside meadow | 49,04499817 | -113,913002 | |
| 08BBLEP-03702 | 658 | JX008099 | Crambidae | Scopariinae | Gesneria centuriella | Denis & Schiffermüller | J.Straka | 21-Jul-2008 | Canada | Alberta | Waterton | Waterton Lakes Nat. Park - Townsite Campgrounc | Riverside/lakeside meadow | 49,04499817 | -113,913002 | |
| 08BBLEP-03712 | 658 | JX008190 | Crambidae | Scopariinae | Gesneria centuriella | Denis & Schiffermüller | J.Straka | 21-Jul-2008 | Canada | Alberta | Waterton | Waterton Lakes Nat. Park - Townsite Campgrounc | Riverside/lakeside meadow | 49,04499817 | -113,913002 | |
| 08BBLEP-03714 | 658 | JX008233 | Crambidae | Scopariinae | Gesneria centuriella | Denis & Schiffermüller | J.Straka | 21-Jul-2008 | Canada | Alberta | Waterton | Waterton Lakes Nat. Park - Townsite Campgrounc | Riverside/lakeside meadow | 49,04499817 | -113,913002 | |
| 08BBLEP-03835 | 658 | JX008284 | Crambidae | Scopariinae | Gesneria centuriella | Denis & Schiffermüller | J.Straka,J.Cossey | 25-Jul-2008 | Canada | Alberta | Waterton | Waterton Lakes Nat. Park - Carthew Creek | Meadow/creekside | 49,04899979 | -113,913002 | |
| 08BBLEP-03849 | 658 | JX008169 | Crambidae | Scopariinae | Gesneria centuriella | Denis & Schiffermüller | J.Straka,J.Cossey | 25-Jul-2008 | Canada | Alberta | Waterton | Waterton Lakes Nat. Park - Carthew Creek | Meadow/creekside | 49,04899979 | -113,913002 | |
| 08BBLEP-03855 | 658 | JX008387 | Crambidae | Scopariinae | Gesneria centuriella | Denis & Schiffermüller | J.Straka,J.Cossey | 25-Jul-2008 | Canada | Alberta | Waterton | Waterton Lakes Nat. Park - Carthew Creek | Meadow/creekside | 49,04899979 | -113,913002 | |
| 08BBLEP-03857 | 658 | JX008288 | Crambidae | Scopariinae | Gesneria centuriella | Denis & Schiffermüller | J.Straka,J.Cossey | 25-Jul-2008 | Canada | Alberta | Waterton | Waterton Lakes Nat. Park - Carthew Creek | Meadow/creekside | 49,04899979 | -113,913002 | |
| 08BBLEP-03859 | 658 | JX008409 | Crambidae | Scopariinae | Gesneria centuriella | Denis & Schiffermüller | J.Straka,J.Cossey | 25-Jul-2008 | Canada | Alberta | Waterton | Waterton Lakes Nat. Park - Carthew Creek | Meadow/creekside | 49,04899979 | -113,913002 | |
| 08BBLEP-04266 | 658 | JX008416 | Crambidae | Scopariinae | Gesneria centuriella | Denis & Schiffermüller | J.Straka,J.Cossey | 04-Aug-2008 | Canada | Alberta | Banff | Banff Nat. Park - Johnson Lake Area | Meadow, beside creek and lake | 51,19800186 | -115,4789963 | |
| 08BBLEP-04269 | 658 | JX008441 | Crambidae | Scopariinae | Gesneria centuriella | Denis & Schiffermüller | J.Straka,J.Cossey | 04-Aug-2008 | Canada | Alberta | Banff | Banff Nat. Park - Johnson Lake Area | Meadow, beside creek and lake | 51,19800186 | -115,4789963 | |
| 08BBLEP-04272 | 658 | JX008161 | Crambidae | Scopariinae | Gesneria centuriella | Denis & Schiffermüller | J.Straka,J.Cossey | 04-Aug-2008 | Canada | Alberta | Banff | Banff Nat. Park - Johnson Lake Area | Meadow, beside creek and lake | 51,19800186 | -115,4789963 | |
| 08BBLEP-04273 | 658 | JX008474 | Crambidae | Scopariinae | Gesneria centuriella | Denis & Schiffermüller | J.Straka,J.Cossey | 04-Aug-2008 | Canada | Alberta | Banff | Banff Nat. Park - Johnson Lake Area | Meadow, beside creek and lake | 51,19800186 | -115,4789963 | |

| | | | | | | | | | | | | | | | | |
|---|---|---|---|---|---|---|---|---|---|---|---|---|---|---|---|---|
| 08BBLEP-04274 | 658 | JX008184 | Crambidae | Scopariinae | Gesneria centuriella | Denis & Schiffermüller | J.Straka,J.Cossey | 04-Aug-2008 | Canada | Alberta | Banff | Banff Nat. Park - Johnson Lake Area | Meadow, beside creek and lake | 51,19800186 | -115,4789963 | |
| 08BBLEP-04282 | 658 | JX008170 | Crambidae | Scopariinae | Gesneria centuriella | Denis & Schiffermüller | J.Straka,J.Cossey | 04-Aug-2008 | Canada | Alberta | Banff | Banff Nat. Park - Johnson Lake Area | Meadow, beside creek and lake | 51,19800186 | -115,4789963 | |
| 08BBLEP-04297 | 658 | JX008405 | Crambidae | Scopariinae | Gesneria centuriella | Denis & Schiffermüller | J.Straka,J.Cossey | 04-Aug-2008 | Canada | Alberta | Banff | Banff Nat. Park - Johnson Lake Area | Meadow, beside creek and lake | 51,19800186 | -115,4789963 | |
| 08BBLEP-04309 | 658 | JX008309 | Crambidae | Scopariinae | Gesneria centuriella | Denis & Schiffermüller | J.Straka,J.Cossey | 04-Aug-2008 | Canada | Alberta | Banff | Banff Nat. Park - Johnson Lake Area | Meadow, beside creek and lake | 51,19800186 | -115,4789963 | |
| 08BBLEP-04447 | 633 | JX008395 | Crambidae | Scopariinae | Gesneria centuriella | Denis & Schiffermüller | J.Straka,J.Cossey | 31-Jul-2008 | Canada | Alberta | Banff | Banff Nat. Park - Cave and Basin Area | Spring-fed marsh/meadow | 51,17100143 | -115,5879974 | |
| 08BBLEP-04450 | 656 | JX008348 | Crambidae | Scopariinae | Gesneria centuriella | Denis & Schiffermüller | J.Straka,J.Cossey | 31-Jul-2008 | Canada | Alberta | Banff | Banff Nat. Park - Cave and Basin Area | Spring-fed marsh/meadow | 51,17100143 | -115,5879974 | |
| 08BBLEP-04451 | 658 | JX008362 | Crambidae | Scopariinae | Gesneria centuriella | Denis & Schiffermüller | J.Straka,J.Cossey | 31-Jul-2008 | Canada | Alberta | Banff | Banff Nat. Park - Cave and Basin Area | Spring-fed marsh/meadow | 51,17100143 | -115,5879974 | |
| 08BBLEP-04459 | 655 | JX008249 | Crambidae | Scopariinae | Gesneria centuriella | Denis & Schiffermüller | J.Straka,J.Cossey | 31-Jul-2008 | Canada | Alberta | Banff | Banff Nat. Park - Cave and Basin Area | Spring-fed marsh/meadow | 51,17100143 | -115,5879974 | |
| 08BBLEP-04460 | 599 | JX008232 | Crambidae | Scopariinae | Gesneria centuriella | Denis & Schiffermüller | J.Straka,J.Cossey | 31-Jul-2008 | Canada | Alberta | Banff | Banff Nat. Park - Cave and Basin Area | Spring-fed marsh/meadow | 51,17100143 | -115,5879974 | |
| 08BBLEP-04462 | 621 | JX008429 | Crambidae | Scopariinae | Gesneria centuriella | Denis & Schiffermüller | J.Straka,J.Cossey | 31-Jul-2008 | Canada | Alberta | Banff | Banff Nat. Park - Cave and Basin Area | Spring-fed marsh/meadow | 51,17100143 | -115,5879974 | |
| 08BBLEP-04479 | 658 | JX008354 | Crambidae | Scopariinae | Gesneria centuriella | Denis & Schiffermüller | J.Straka,J.Cossey | 31-Jul-2008 | Canada | Alberta | Banff | Banff Nat. Park - Cave and Basin Area | Spring-fed marsh/meadow | 51,17100143 | -115,5879974 | |
| 08BBLEP-04481 | 622 | JX008266 | Crambidae | Scopariinae | Gesneria centuriella | Denis & Schiffermüller | J.Straka,J.Cossey | 31-Jul-2008 | Canada | Alberta | Banff | Banff Nat. Park - Cave and Basin Area | Spring-fed marsh/meadow | 51,17100143 | -115,5879974 | |
| 08BBLEP-04508 | 658 | JX008280 | Crambidae | Scopariinae | Gesneria centuriella | Denis & Schiffermüller | J.Straka,J.Cossey | 31-Jul-2008 | Canada | Alberta | Banff | Banff Nat. Park - Cave and Basin Area | Spring-fed marsh/meadow | 51,17100143 | -115,5879974 | |
| 08BBLEP-04514 | 634 | JX008337 | Crambidae | Scopariinae | Gesneria centuriella | Denis & Schiffermüller | J.Straka,J.Cossey | 31-Jul-2008 | Canada | Alberta | Banff | Banff Nat. Park - Cave and Basin Area | Spring-fed marsh/meadow | 51,17100143 | -115,5879974 | |
| 08BBLEP-04516 | 658 | JX008378 | Crambidae | Scopariinae | Gesneria centuriella | Denis & Schiffermüller | J.Straka,J.Cossey | 31-Jul-2008 | Canada | Alberta | Banff | Banff Nat. Park - Cave and Basin Area | Spring-fed marsh/meadow | 51,17100143 | -115,5879974 | |
| 08BBLEP-04517 | 658 | JX008186 | Crambidae | Scopariinae | Gesneria centuriella | Denis & Schiffermüller | J.Straka,J.Cossey | 31-Jul-2008 | Canada | Alberta | Banff | Banff Nat. Park - Cave and Basin Area | Spring-fed marsh/meadow | 51,17100143 | -115,5879974 | |
| 08BBLEP-04522 | 658 | JX008171 | Crambidae | Scopariinae | Gesneria centuriella | Denis & Schiffermüller | J.Straka,J.Cossey | 31-Jul-2008 | Canada | Alberta | Banff | Banff Nat. Park - Cave and Basin Area | Spring-fed marsh/meadow | 51,17100143 | -115,5879974 | |
| 08BBLEP-04524 | 632 | JX008138 | Crambidae | Scopariinae | Gesneria centuriella | Denis & Schiffermüller | J.Straka,J.Cossey | 31-Jul-2008 | Canada | Alberta | Banff | Banff Nat. Park - Cave and Basin Area | Spring-fed marsh/meadow | 51,17100143 | -115,5879974 | |
| 08BBLEP-04527 | 658 | JX008234 | Crambidae | Scopariinae | Gesneria centuriella | Denis & Schiffermüller | J.Straka,J.Cossey | 31-Jul-2008 | Canada | Alberta | Banff | Banff Nat. Park - Cave and Basin Area | Spring-fed marsh/meadow | 51,17100143 | -115,5879974 | |
| 08BBLEP-04530 | 658 | JX008394 | Crambidae | Scopariinae | Gesneria centuriella | Denis & Schiffermüller | J.Straka,J.Cossey | 31-Jul-2008 | Canada | Alberta | Banff | Banff Nat. Park - Cave and Basin Area | Spring-fed marsh/meadow | 51,17100143 | -115,5879974 | |
| 08BBLEP-04544 | 613 | JX008445 | Crambidae | Scopariinae | Gesneria centuriella | Denis & Schiffermüller | J.Straka,J.Cossey | 31-Jul-2008 | Canada | Alberta | Banff | Banff Nat. Park - Cave and Basin Area | Spring-fed marsh/meadow | 51,17100143 | -115,5879974 | |
| 08BBLEP-04545 | 612 | JX008305 | Crambidae | Scopariinae | Gesneria centuriella | Denis & Schiffermüller | J.Straka,J.Cossey | 31-Jul-2008 | Canada | Alberta | Banff | Banff Nat. Park - Cave and Basin Area | Spring-fed marsh/meadow | 51,17100143 | -115,5879974 | |
| 08BBLEP-04546 | 646 | JX008340 | Crambidae | Scopariinae | Gesneria centuriella | Denis & Schiffermüller | J.Straka,J.Cossey | 31-Jul-2008 | Canada | Alberta | Banff | Banff Nat. Park - Cave and Basin Area | Spring-fed marsh/meadow | 51,17100143 | -115,5879974 | |
| 08BBLEP-04548 | 613 | JX008419 | Crambidae | Scopariinae | Gesneria centuriella | Denis & Schiffermüller | J.Straka,J.Cossey | 31-Jul-2008 | Canada | Alberta | Banff | Banff Nat. Park - Cave and Basin Area | Spring-fed marsh/meadow | 51,17100143 | -115,5879974 | |
| 08BBLEP-04552 | 658 | JX008269 | Crambidae | Scopariinae | Gesneria centuriella | Denis & Schiffermüller | J.Straka,J.Cossey | 31-Jul-2008 | Canada | Alberta | Banff | Banff Nat. Park - Cave and Basin Area | Spring-fed marsh/meadow | 51,17100143 | -115,5879974 | |
| 08BBLEP-04742 | 658 | JX008241 | Crambidae | Scopariinae | Gesneria centuriella | Denis & Schiffermüller | J.Straka,J.Cossey | 04-Aug-2008 | Canada | Alberta | Banff | Banff Nat. Park - Johnson Lake Area | Mixed forest/grassland | 51,19900131 | -115,4869995 | |
| 08-JDWBC-1392 | 658 | JX008428 | Crambidae | Scopariinae | Gesneria centuriella | Denis & Schiffermüller | deWaard, J.R. | 09-Jul-2008 | Canada | British Columbia | Kamloops | Sugarloaf Ranch | TRSB1 | 50,63800049 | -120,4509964 | |
| 09PROBE-09305 | 658 | HM375562 | Crambidae | Scopariinae | Gesneria centuriella | Denis & Schiffermüller | P.D.N. Hebert | 26-Jul-2009 | Canada | Manitoba | Churchill | 26 km SE Churchill, Twin Lakes burn site | | 58,61999893 | -93,83000183 | |
| 10BBCLP-1185 | 658 | JF842198 | Crambidae | Scopariinae | Gesneria centuriella | Denis & Schiffermüller | BIObus 2010 | 23-Jun-2010 | Canada | Alberta | Jasper NP | Whistlers Cmpgrd | Pine forest | 52,8429985 | -118,0749969 | |
| 10BBCLP-1191 | 658 | JF842204 | Crambidae | Scopariinae | Gesneria centuriella | Denis & Schiffermüller | BIObus 2010 | 22-Jul-2010 | Canada | British Columbia | Yoho NP | Kicking Horse Cmpgrd | Forested cmpgrd | 51,42399979 | -116,4290009 | |
| 10-JDWBC-3083 | 658 | HM864912 | Crambidae | Scopariinae | Gesneria centuriella | Denis & Schiffermüller | deWaard, J.R. | 30-Jul-2009 | Canada | British Columbia | nr. Hazelton | Date Cr. Silvicultural System | B5-T | 55,42699814 | -127,8030014 | |
| 10-JDWBC-3811 | 658 | HM865709 | Crambidae | Scopariinae | Gesneria centuriella | Denis & Schiffermüller | deWaard, J.R. | 30-Jul-2009 | Canada | British Columbia | nr. Hazelton | Date Creek Silvicultural System | B3-T | 55,43899918 | -127,814003 | |
| 10-JDWBC-4047 | 633 | HQ682305 | Crambidae | Scopariinae | Gesneria centuriella | Denis & Schiffermüller | deWaard, J.R. | 30-Jul-2009 | Canada | British Columbia | nr. Hazelton | Date Cr. Silvicultural System | A2-T | 55,4659996 | -127,8150024 | |
| 10-JDWBC-5949 | 658 | HM868036 | Crambidae | Scopariinae | Gesneria centuriella | Denis & Schiffermüller | deWaard, J.R. | 10-Jul-2009 | Canada | British Columbia | Okanagan Falls | Irrigation Rd. East | OFIR13 | 49,31200027 | -119,5019989 | |
| 10-JDWBC-6012 | 658 | HM868104 | Crambidae | Scopariinae | Gesneria centuriella | Denis & Schiffermüller | deWaard, J.R. | 11-Jul-2009 | Canada | British Columbia | nr. Kamloops | Coquihalla Highway | TRCQ1 | 50,64300156 | -120,4850006 | |
| 10-JDWBC-6054 | 658 | HM868149 | Crambidae | Scopariinae | Gesneria centuriella | Denis & Schiffermüller | deWaard, J.R. | 11-Jul-2009 | Canada | British Columbia | nr. Kamloops | Dewdrop Ecoreserve East | TRDE2 | 50,74700165 | -120,5550003 | |
| 10-JDWBC-6101 | 658 | HM868201 | Crambidae | Scopariinae | Gesneria centuriella | Denis & Schiffermüller | deWaard, J.R. | 11-Jul-2009 | Canada | British Columbia | nr. Kamloops | Six Mile East transect | TR6M1 | 50,73899841 | -120,7269974 | |
| 10-JDWBC-6393 | 658 | HM868505 | Crambidae | Scopariinae | Gesneria centuriella | Denis & Schiffermüller | deWaard, J.R. | 04-Aug-2009 | Canada | British Columbia | nr. Kamloops | Coquihalla Highway | TRCQ1 | 50,64300156 | -120,4850006 | |
| CNCLEP00025413 | 623 | JX008114 | Crambidae | Scopariinae | Gesneria centuriella | Denis & Schiffermüller | JF Landry & L Humble | 01-Jun-2006 | Canada | British Columbia | | 4.5 km NE Kelowna | Beaver Lake R | 50,0359993 | -119,348999 | PYR766 M |
| CNCLEP00059307 | 658 | JX008126 | Crambidae | Scopariinae | Gesneria centuriella | Denis & Schiffermüller | E.E. Sterns | 26-Jul-1955 | Canada | Newfoundland and Labrador | | Cartwright | | 53,70600128 | -57,02700043 | PYR567 F |
| CNCLEP00067183 | 658 | HQ970952 | Crambidae | Scopariinae | Gesneria centuriella | Denis & Schiffermüller | K. Mikkola | 07-Jun-1992 | United States | Wyoming | | | Bubois Togwotee Pas | | | |
| CNCLEP00067842 | 658 | JX008236 | Crambidae | Scopariinae | Gesneria centuriella | Denis & Schiffermüller | B. Landry | 02-Jul-1988 | Canada | Quebec | Abitibi-Ouest | Lasarre | | 48,80699921 | -79,20500183 | |
| CNCLEP00067843 | 658 | HQ575153 | Crambidae | Scopariinae | Gesneria centuriella | Denis & Schiffermüller | Henry Hensel | 21-Jul-1991 | Canada | Manitoba | | Churchill | 24 km E Northern Studies Centre | 58,73400116 | -93,80999756 | |
| CNCLEP00067844 | 658 | HQ575154 | Crambidae | Scopariinae | Gesneria centuriella | Denis & Schiffermüller | A. Mutuura | 15-Jul-1968 | Canada | Newfoundland and Labrador | | near Doyles | | 47,83100128 | -59,19100189 | |
| CNCLEP00067845 | 658 | JX008246 | Crambidae | Scopariinae | Gesneria centuriella | Denis & Schiffermüller | Henry Hense | 04-Aug-1990 | Canada | Quebec | | vicinity of Schefferville | | 54,82099915 | -66,82499695 | |
| CNCLEP00067846 | 658 | JX008101 | Crambidae | Scopariinae | Gesneria centuriella | Denis & Schiffermüller | Henry Hense | 01-Aug-1990 | Canada | Quebec | | vicinity of Schefferville | | 54,82099915 | -66,82499695 | |
| CNCLEP00067847 | 658 | JX008442 | Crambidae | Scopariinae | Gesneria centuriella | Denis & Schiffermüller | Henry Hense | 03-Aug-1990 | Canada | Quebec | | vicinity of Schefferville | | 54,82099915 | -66,82499695 | |
| CNCLEP00067848 | 658 | JX008127 | Crambidae | Scopariinae | Gesneria centuriella | Denis & Schiffermüller | Henry Hense | 01-Aug-1990 | Canada | Quebec | | vicinity of Schefferville | | 54,82099915 | -66,82499695 | PYR568 M |
| CNCLEP00068810 | 658 | JX008131 | Crambidae | Scopariinae | Gesneria centuriella | Denis & Schiffermüller | Normand Juneau | 06-Jul-2002 | Canada | Quebec | | Oriskany | | 47,48899841 | -73,65499878 | |
| CNCLEP00068811 | 658 | JX008136 | Crambidae | Scopariinae | Gesneria centuriella | Denis & Schiffermüller | Normand Juneau | 06-Jul-2002 | Canada | Quebec | | Oriskany | | 47,48899841 | -73,65499878 | |
| CNCLEP00068812 | 658 | JX008103 | Crambidae | Scopariinae | Gesneria centuriella | Denis & Schiffermüller | Normand Juneau | 06-Jul-2002 | Canada | Quebec | | Oriskany | | 47,48899841 | -73,65499878 | |
| CNCLEP00068813 | 658 | JX008158 | Crambidae | Scopariinae | Gesneria centuriella | Denis & Schiffermüller | Normand Juneau | 25-Jun-2000 | Canada | Quebec | | Oriskany | | 47,48899841 | -73,65499878 | |
| CNCLEP00068814 | 658 | HQ575157 | Crambidae | Scopariinae | Gesneria centuriella | Denis & Schiffermüller | Normand Juneau | 09-Jul-2002 | Canada | Quebec | | Oriskany | | 47,48899841 | -73,65499878 | |
| CNCLEP00078036 | 658 | JX008179 | Crambidae | Scopariinae | Gesneria centuriella | Denis & Schiffermüller | H. Hense | 14-Jul-2002 | Canada | Nunavut | vic. Arviat | | | 61,09600067 | -94,0719986 | |
| KENWR 6636 | 658 | JX008410 | Crambidae | Scopariinae | Gesneria centuriella | Denis & Schiffermüller | D.Collet | | United States | Alaska | Kenai National Wildlife Refuge | | | | | |
| KENWR 6645 | 658 | JX008148 | Crambidae | Scopariinae | Gesneria centuriella | Denis & Schiffermüller | D.Collet | 08-Jul-2004 | United States | Alaska | Kenai National Wildlife Refuge | Sterling, Corey St. | glauca forest | 60,5 | | |

| | | | | | | | | | | | | | | | | |
|---|---|---|---|---|---|---|---|---|---|---|---|---|---|---|---|---|
| KENWR 6645b | 658 | JX008185 | Crambidae | Scopariinae | Gesneria centuriella | Denis & Schiffermüller | D.Collet | 08-Jul-2004 | United States | Alaska | Kenai National Wildlife Refuge | Sterling, Corey St. | glauca forest | 60,5 | | |
| KENWR 6646 | 655 | JX008162 | Crambidae | Scopariinae | Gesneria centuriella | Denis & Schiffermüller | D.Collet | 10-Jul-2004 | United States | Alaska | Kenai National Wildlife Refuge | Sterling, Corey St | glauca forest | 60,5 | -150,8329926 | |
| KENWR LEP-18 | 658 | JX008317 | Crambidae | Scopariinae | Gesneria centuriella | Denis & Schiffermüller | D.Collet | 19-Jun-2004 | United States | Alaska | Kenai National Wildlife Refuge | Finger Lake Road | closed birch forest | 60,65000153 | -150,8329926 | |
| MDH00290 | 658 | JX008427 | Crambidae | Scopariinae | Gesneria centuriella | Denis & Schiffermüller | Daniel Handfield | 18-Jun-2003 | Canada | Quebec | CAN:QC -Carleton | | Chemin St-Onge | | | |
| MM10542 | 603 | HQ570384 | Crambidae | Scopariinae | Gesneria centuriella | Denis & Schiffermüller | Marko Mutanen | | Finland | | Lapponia enontekiensi | | Enontekioe | 68,99700165 | 20,74399948 | |
| MM15775 | 658 | HM876555 | Crambidae | Scopariinae | Gesneria centuriella | Denis & Schiffermüller | Tomi Mutanen | 29-Jun-1996 | Finland | | Ostrobottnia borealis pars borealis | | Tornio | 65,77100372 | 24,37999916 | |
| TLMF Lep 0047 | 658 | HM425950 | Crambidae | Scopariinae | Gesneria centuriella | Denis & Schiffermüller | Huemer P | 05-Jun-2009 | Italy | South Tyro | | | Bad Ratzes S/ Seis am Schlern | 46,52999878 | 11,58640003 | |
| UBC-2006-0392 | 656 | JX008268 | Crambidae | Scopariinae | Gesneria centuriella | Denis & Schiffermüller | A Li & J. Derhousoff | 27-Jun-2006 | Canada | British Columbia | | Pitt Meadows | | 49,26900101 | -122,6389999 | |
| BC NP 0306 | 658 | JX034647 | Geometridae | Ennominae | Glacies coracina | Esper | K. Huber | 29-Jun-2000 | Sweden | Norrbotter | | | Abisko | 68,36000061 | 18,69000053 | |
| BC NP 0307 | 658 | JX034652 | Geometridae | Ennominae | Glacies coracina | Esper | N. Poel | 17-Jul-2005 | Slovenia | | Julian Alps | | Prestreljenik | 46,34999847 | 13,55000019 | |
| BC NP 0308 | 658 | JX034592 | Geometridae | Ennominae | Glacies coracina | Esper | N. Poel | 20-Jul-2007 | Austria | | Salzburg | | Dorfertal, Im Grund | 47,11000061 | 12,61999989 | |
| MM0008 | 658 | HM396368 | Geometridae | Ennominae | Glacies coracina | Esper | Marko Mutanen | | Finland | | | | Enontekioe | 68,99700165 | 20,74399948 | |
| MM0411 | 658 | HM386901 | Geometridae | Ennominae | Glacies coracina | Esper | Marko Mutanen | | Finland | | | | Enontekioe | 68,99700165 | 20,74399948 | |
| MM06309 | 658 | HM873192 | Geometridae | Ennominae | Glacies coracina | Esper | Marko Mutanen, Anttoni Mutanen, Nestori Mutanen | 28-Jun-2006 | Finland | | Lapponia inarensis | | Inari | 68,4469986 | 27,41399956 | |
| TLMF Lep 0034 | 658 | HM425833 | Geometridae | Ennominae | Glacies coracina | Esper | Huemer P | 20-Jul-2009 | Italy | | Belluno | | Passo di Valparola E | 46,52550125 | 11,99979973 | |
| TLMF Lep 0038 | 658 | HM425871 | Geometridae | Ennominae | Glacies coracina | Esper | Huemer P | 01-Aug-2009 | Austria | Karnten | | | Mallnitz, Doesental, Seealı | 46,99309921 | 13,27110004 | |
| TLMF Lep 01463 | 620 | HQ968679 | Geometridae | Ennominae | Glacies coracina | Esper | Habeler H. | 05-Jul-2009 | Austria | Steiermark | | | Kornock NE-Flanke/ Turracher Hoehe NW | 46,91699982 | 13,85700035 | |
| TLMF Lep 0146 | 658 | HQ968680 | Geometridae | Ennominae | Glacies coracina | Esper | Habeler H | 11-Jul-2003 | Austria | Steiermark | | | Klosterneuburger Huett | 47,26499939 | 14,37800026 | |
| BC ZSM Lep 15089 | 609 | JX034631 | Geometridae | Ennominae | Gnophos obfuscata | Denis & Schiffermüller | G Petranyi | 28-Jun-2008 | Italy | Trentino-Alto Adige | Sesvenna | | 2km W Schlienig | 46,71670151 | 10,44999981 | |
| BC ZSM Lep 2202 | 658 | GU686952 | Geometridae | Ennominae | Gnophos obfuscata | Denis & Schiffermüller | U. Buchsbaum | 20-Aug-1998 | Germany | Bavaria | Oberbayern | | Wettersteingebirge, Kreuzeck | 47,45000076 | 11,06669998 | |
| BC ZSM Lep 24055 | 658 | GU687337 | Geometridae | Ennominae | Gnophos obfuscata | Denis & Schiffermüller | A. Haslberger | 15-Jul-2007 | Germany | Bavaria | Schwaben | Oberallgaeu | Oberstdorf, Edmund-Probst-Haus | 47,41289902 | 10,34850025 | |
| BC ZSM Lep 2405 | 658 | GU687330 | Geometridae | Ennominae | Gnophos obfuscata | Denis & Schiffermüller | A. Haslberger | 25-Jul-2001 | Germany | Bavaria | Oberbayern | Berchtesgadener Land | Reiter Alm, Hirschwiese | | | |
| MM12883 | 658 | HM875424 | Geometridae | Ennominae | Gnophos obfuscata | Denis & Schiffermüller | Marko Mutanen, Panu Vaelimaen | | Finland | | Regio Aboensis | | Dragsfjaerd | 60,01100159 | 22,47999919 | |
| MM15842 | 658 | HM876617 | Geometridae | Ennominae | Gnophos obfuscata | Denis & Schiffermüller | Marko Mutanen | 27-Jul-2005 | Finland | | Regio Aboensis | | Dragsfjaerd | 59,97000122 | 22,34199905 | |
| MM15843 | 658 | HM876618 | Geometridae | Ennominae | Gnophos obfuscata | Denis & Schiffermüller | Marko Mutanen | 21-Jul-2004 | Finland | | Regio Aboensis | | Lohja | 60,19200134 | 23,75499916 | |
| TLMF Lep 00310 | 658 | HM425807 | Geometridae | Ennominae | Gnophos obfuscata | Denis & Schiffermüller | Huemer P. | 16-Jul-2009 | Austria | Vorarlberg | | | Marul, Lagutzbach, Beim alten Stafel | 47,20220184 | 9,928059578 | |
| TLMF Lep 00417 | 658 | HM425900 | Geometridae | Ennominae | Gnophos obfuscata | Denis & Schiffermüller | Huemer P. | 23-Jul-2009 | Italy | | Cuneo | | Colle Valcavera NE/ Demonte NW | 44,38439941 | 7,106389999 | |
| TLMF Lep 01102 | 658 | HQ968469 | Geometridae | Ennominae | Gnophos obfuscata | Denis & Schiffermüller | Aistleitner U. | 20-Jul-2009 | Switzerland | Graubunden | | | Tiefencastel S/ Salouf, Got Grond | 46,65000153 | 9,597000122 | |
| TLMF Lep 01973 | 658 | HQ968384 | Geometridae | Ennominae | Gnophos obfuscata | Denis & Schiffermüller | Huemer P. | 14-Jul-2010 | Italy | Abruzzi | L`Aquila | | NP Gran Sasso, Campo Imperatore, ex Miniera di Lignite | 42,42670059 | 13,71329975 | |
| TLMF Lep 0199 | 658 | HQ968409 | Geometridae | Ennominae | Gnophos obfuscata | Denis & Schiffermüller | Huemer P | 16-Jul-2010 | Italy | Lazio | Rieti | | Monte Terminillo I | 42,48329926 | 13 | |
| TLMF Lep 0246 | 658 | JF860004 | Geometridae | Ennominae | Gnophos obfuscata | Denis & Schiffermüller | Huemer P | 19-Jul-2010 | Italy | South Tyro | | | Ritten/ Obergruenwal | 46,59700012 | 11,43900013 | |
| TLMF Lep 05547 | 658 | JX034678 | Geometridae | Ennominae | Gnophos obfuscata | Denis & Schiffermüller | Huemer P. & Tarmann G. M. | 28-Jul-2011 | Macedonia | | | | Mavrovo NP, Korab, Korabska jezero, Kobilino pole | 41,77799988 | 20,58200073 | |
| TLMF Lep 05548 | 658 | JX034581 | Geometridae | Ennominae | Gnophos obfuscata | Denis & Schiffermüller | Huemer P. & Tarmann G. M. | 28-Jul-2011 | Macedonia | | | | Mavrovo NP, Korab, Korabska jezero, Kobilino pole | 41,77799988 | 20,58200073 | |
| 07PROBE-1081 | 658 | JX008174 | Gelechiidae | Gelechiinae | Gnorimoschema alaskens | Povolny | P.D.N. Hebert | 23-Jul-2007 | Canada | Manitoba | Churchill | 26 km SE Churchill, Twin Lake | | 58,63000107 | -93,81900024 | MIC5443 M |
| CNCLEP0006134 | 658 | GU693677 | Gelechiidae | Gelechiinae | Gnorimoschema alaskens | Povolny | J.F. Landry | 23-Jun-1983 | Canada | Saskatchewan | Maidstone | 55 km SE Lloydministe | | 53,08599854 | -109,2959976 | |
| CNCLEP0006138 | 658 | GU693373 | Gelechiidae | Gelechiinae | Gnorimoschema alaskens | Povolny | B.C. Schmidt | 03-Jul-2004 | Canada | Yukon Territory | N Whitehorse | Yukon Rd. at Takhini Rive | S facing grassy slope | 60,84000015 | -135,1900024 | MIC5967 M |
| CNCLEP0006138 | 658 | GU693370 | Gelechiidae | Gelechiinae | Gnorimoschema alaskens | Povolny | B.C. Schmidt | 03-Jul-2004 | Canada | Yukon Territory | N Whitehorse | Yukon Rd. at Takhini Rive | S facing grassy slope | 60,84000015 | -135,1900024 | |
| CNCLEP00067547 | 658 | GU693492 | Gelechiidae | Gelechiinae | Gnorimoschema alaskense | Povolny | B. Landry | 12-Jul-1993 | United States | Colorado | Gilpin Co. | Roosevelt national Forest Campground | 3 km S Nederland | 39,93999863 | -105,5100021 | MIC5942 F |
| MZH-LEP000000 | 658 | HM379459 | Gelechiidae | Gelechiinae | Gnorimoschema alaskens | Povolny | M. Ahola & L. Kaila | 12-Jul-1994 | Canada | Yukon Territory | | Carcross | | 60,18600082 | -134,6970062 | |
| POHL-10-0015 | 658 | HQ575101 | Gelechiidae | Gelechiinae | Gnorimoschema alaskens | Povolny | Pohl, G. R.: Kutash, T | 13-Jun-1995 | Canada | Saskatchewan | | | vic. Big River | 53,81999969 | -107,0299988 | |
| POHL-10-00159 | 658 | HM887802 | Gelechiidae | Gelechiinae | Gnorimoschema alaskense | Povolny | Pohl, G. R. | 01-Jun-2006 | Canada | Alberta | Strathcona County | | 8km southest of Sherwood Park | 53,47800064 | -113,2289963 | |
| POHL-10-00160 | 658 | HM887803 | Gelechiidae | Gelechiinae | Gnorimoschema alaskense | Povolny | Pohl, G. R. | 13-Jul-2002 | Canada | Alberta | Strathcona County | | 8km southest of Sherwood Park | 53,47800064 | -113,2289963 | |
| POHL-10-00168 | 658 | HM887811 | Gelechiidae | Gelechiinae | Gnorimoschema alaskense | Povolny | Pohl, G. R. | 30-Jun-2008 | Canada | Alberta | Strathcona County | | 8km southest of Sherwood Park | 53,47800064 | -113,2289963 | |
| MM06377 | 658 | HQ570340 | Gelechiidae | Gelechiinae | Gnorimoschema valesiella | Staudinger | Marko Mutanen, Anttoni Mutanen, Nestori Mutanen | 29-Jun-2007 | Finland | | Lapponia inarensis | | Utsjoki | 69,37599945 | 25,80500031 | |
| MM14150 | 658 | HM875916 | Gelechiidae | Gelechiinae | Gnorimoschema valesiella | Staudinger | Marko Mutanen, Nestori Mutanen, Anttoni Mutanen | 21-Jun-2009 | Finland | | Ostrobottnia kajanensis | | Ristijaervi | 64,33200073 | 28,59300041 | |
| MM1563 | 658 | HM876419 | Gelechiidae | Gelechiinae | Gnorimoschema valesiell | Staudinger | Tomi Mutanen | 31-May-2002 | Finland | | Satakunda | | Saekylae | 60,95999908 | 22,38299942 | |
| TLMF Lep 01919 | 658 | HQ968331 | Gelechiidae | Gelechiinae | Gnorimoschema valesiella | Staudinger | Huemer P. | 02-Aug-2010 | Italy | | Cuneo | | Colle Valcavera Umgebung/ Demonte NW | 44,38140106 | 7,101669788 | |
| 07PROBE-1068 | 658 | JX008380 | Arctiidae | Arctiinae | Grammia quense | Paykull | P.D.N. Hebert | 22-Jul-2007 | Canada | Manitoba | Churchill | 23 km E Churchill, Ramsay Cree | | 58,73099899 | -93,77999878 | |
| BC AB Lep 0004 | 658 | HM910288 | Arctiidae | Arctiinae | Grammia quense | Paykull | Rothacher | 15-Jul-2009 | Switzerland | Graubunder | Tschierv | | National park, Ofen-Pas | 46,63940048 | 10,29220009 | |
| BC AB Lep 00041 | 658 | HM910289 | Arctiidae | Arctiinae | Grammia quense | Paykull | Rothacher | 15-Jul-2009 | Switzerland | Graubunder | Tschierv | | National park, Ofen-Pas | 46,63940048 | 10,29220009 | |
| BC AB Lep 00042 | 658 | HM910290 | Arctiidae | Arctiinae | Grammia quense | Paykull | Rothacher | 15-Jul-2009 | Switzerland | Graubunder | Tschierv | | National park, Ofen-Pas | 46,63940048 | 10,29220009 | |
| CHU06-LEP-033 | 658 | JX008366 | Arctiidae | Arctiinae | Grammia quenseli | Paykull | Arctic & Boreal Entomology | 18-Aug-2006 | Canada | Manitoba | Churchill | 16 km E Churchill, krummholz formation | Tundra | 58,75400162 | -93,91300201 | |
| LEP03195 | 591 | JX008320 | Arctiidae | Arctiinae | Grammia quense | Paykull | | | United States | New Hampshire | Mt Washington | | | | | |
| LEP037809 | 658 | JX008399 | Arctiidae | Arctiinae | Grammia quenseli | Paykull | Jennifer Line | | Canada | Yukon Territory | Mt. Klotz ( SW. of ), dry Dryas slope | | | 65,36499786 | -140,1829987 | |
| LEP037926 | 648 | JX008198 | Arctiidae | Arctiinae | Grammia quenseli | Paykull | Henry Hensel | | Canada | Quebec | Sunny Mtn., tundra 40 Km. NW of Schefferville | | | | | |
| LEP038076 | 648 | JX008166 | Arctiidae | Arctiinae | Grammia quenseli | Paykull | J. Troubridge | | Canada | Newfoundland and Labrador | Forteau | | | | | |
| LEP038077 | 658 | JX008356 | Arctiidae | Arctiinae | Grammia quenseli | Paykull | J. Troubridge | | Canada | Newfoundland and Labrador | Forteau | | | | | |
| LEP041304 | 587 | JX008237 | Arctiidae | Arctiinae | Grammia quenseli | Paykull | | | Canada | Quebec | Schefferville, Bean Lake Tundra | | | | | |
| LEP04130 | 639 | JX008258 | Arctiidae | Arctiinae | Grammia quense | Paykull | | | Canada | Quebec | Schefferville, Sunny Mt | | | | | |
| LEP04130 | 587 | JX008321 | Arctiidae | Arctiinae | Grammia quense | Paykull | | | United States | New Hampshire | Mt Washington | | | | | |
| MM10592 | 658 | HQ570391 | Arctiidae | Arctiinae | Grammia quense | Paykull | Tomi Mutanen | 29-Jun-2003 | Finland | | Lapponia enontekiensi | | Enontekioe | 68,99700165 | 20,74399948 | |
| MM1059 | 658 | HQ570392 | Arctiidae | Arctiinae | Grammia quense | Paykull | Marko Mutanen | | Finland | | Lapponia enontekiensi | | Enontekioe | 68,99700165 | 20,74399948 | |

| | | | | | | | | | | | | | | | |
|---|---|---|---|---|---|---|---|---|---|---|---|---|---|---|---|
| MM10594 | 658 | HQ570393 | Arctiidae | Arctiinae | Grammia quense | Paykull | Marko Mutaner | | Finland | | Lapponia enontekiensi | | Enontekioe | 68,99700165 | 20,7439994ε |
| TLMF Lep 02032 | 658 | HQ968441 | Arctiidae | Arctiinae | Grammia quense | Paykull | Cerny K. | 18-Jul-1999 | Switzerlanc | Graubunder | | Val Muestair | Umbrai | 46,5416984є | 10,43360043 |
| TLMF Lep 02033 | 658 | HQ968442 | Arctiidae | Arctiinae | Grammia quense | Paykull | Cerny K. | 18-Sep-2008 | Austria | Tyrol | Osttiro | Pusterta | Thurntaler/ Sillian NW | 46,77280045 | 12,38360023 |
| TLMF Lep 02735 | 658 | JX034644 | Arctiidae | Arctiinae | Grammia quenseli | Paykull | Schmid J. | 25-Jul-2009 | Switzerland | Graubunden | | | Umg. Piz Minschuns, V. Costainas | 46,58100128 | 10,4829998 |
| 07PROBE-00524 | 653 | JX008377 | Tortricidae | Olethreutinae | Grapholita aureolana | Tengström | J.deWaard | 13-Jul-2007 | Canada | Manitoba | Churchill | Town of Churchil | field S of Churchill train statior | 58,76699829 | -94,1760025 |
| 09PROBE-09495 | 636 | HM375748 | Tortricidae | Olethreutinae | Grapholita aureolana | Tengström | P.D.N. Hebert | | Canada | Manitoba | Churchill | Town of Churchil | locality unconfirmec | 58,75799942 | -94,0680079 |
| MM00081 | 658 | HM396360 | Tortricidae | Olethreutinae | Grapholita aureolana | Tengström | Marko Mutaner | | Finland | | | | Enontekioe | 68,99700165 | 20,7439994ε |
| MM04128 | 658 | HM386916 | Tortricidae | Olethreutinae | Grapholita aureolana | Tengström | Marko Mutaner | | Finland | | | | Enontekioe | 68,99700165 | 20,7439994ε |
| MM04149 | 658 | HM386936 | Tortricidae | Olethreutinae | Grapholita aureolana | Tengström | Marko Mutaner | | Finland | | | | Enontekioe | 68,99700165 | 20,7439994ε |
| MM06375 | 658 | HQ570338 | Tortricidae | Olethreutinae | Grapholita aureolana | Tengström | Marko Mutanen, Anttoni Mutanen, Nestori Mutaner | 29-Jun-2007 | Finland | | Lapponia inarensis | | Utsjoki | 69,37599945 | 25,80500031 |
| TLMF Lep 01805 | 585 | JX034608 | Tortricidae | Olethreutinae | Grapholita aureolana | Tengström | Deutsch H. | 02-Jul-1992 | Austria | Tyrol | Osttiro | | Glo.Grp., Dorfertal, Moar Alr | 47,03329849 | 12,6281004 |
| TLMF Lep 01807 | 609 | HQ968275 | Tortricidae | Olethreutinae | Grapholita aureolana | Tengström | Deutsch H. | 02-Jul-1992 | Austria | Tyrol | Osttiro | | Glo.Grp., Dorfertal, Moar Alr | 47,03329849 | 12,6281004 |
| CNCNoctuoidea13623 | 587 | JX034582 | Arctiidae | Arctiinae | Holoarctia cervin | Fallou | P. Muck | 21-Jan-1982 | Austria | | | | Otzteler Alpen Kreuzpitz | | |
| CNCNoctuoidea13624 | 370 | JX034676 | Arctiidae | Arctiinae | Holoarctia cervin | Fallou | P. Muck | 21-Jan-1982 | Austria | | | | Otzteler Alpen Kreuzpitz | | |
| TLMF Lep 02893 | 658 | JF860346 | Arctiidae | Arctiinae | Holoarctia cervin | Fallou | May B. | 24-Jun-2008 | Switzerlanc | Valais | | | Zermatt / Gornergra | 45,98500061 | 7,76399939 |
| CNCNoctuoidea13625 | 591 | JX034597 | Arctiidae | Arctiinae | Holoarctia puengeler | Bang-Haas | G. Palmqvis | 10-Jul-1980 | Sweden | | | | Nissuntjarrc | | |
| MM19246 | 658 | JF854550 | Arctiidae | Arctiinae | Holoarctia puengeler | Bang-Haas | Timo Nupponen, Kari Nuppone | 07-Jul-2009 | Sweden | | To | | Nissuntjarrc | | |
| MM19247 | 658 | JF854551 | Arctiidae | Arctiinae | Holoarctia puengeler | Bang-Haas | Timo Nupponen, Kari Nuppone | 07-Jul-2009 | Sweden | | To | | Nissuntjarrc | | |
| MM17697 | 658 | JX034589 | Blastobasidae | | Hypatopa segnella | Zeller | Erkki Laasonen, Leena Laasone | | Finland | | Regio Aboensis | | Dragsfjaerd | 60,03409958 | 22,3871994 |
| TLMF Lep 02261 | 658 | JF859836 | Blastobasidae | | Hypatopa segnella | Zeller | Huemer P | 22-Jul-2010 | Italy | South Tyro | Etschta | | Montiggl/ Kleiner Pric | 46,4280014 | 11,30000019 |
| TLMF Lep 02262 | 658 | JF859837 | Blastobasidae | | Hypatopa segnella | Zeller | Huemer P | 22-Jul-2010 | Italy | South Tyro | Etschta | | Montiggl/ Kleiner Pric | 46,4280014 | 11,30000019 |
| TLMF Lep 02736 | 658 | JF860256 | Blastobasidae | | Hypatopa segnella | Zeller | Schmid J. | 18-Jul-2008 | Switzerlanc | Graubunder | | | Ida/ Castaneda | 46,25699997 | 9,144000053 |
| MM00079 | 658 | HM396358 | Prodoxidae | Incurvariinae | Incurvaria vetulella | Zetterstedt | Marko Mutaner | | Finland | | | | Enontekioe | 68,99700165 | 20,7439994ε |
| MM04160 | 658 | HM386947 | Prodoxidae | Incurvariinae | Incurvaria vetulella | Zetterstedt | Marko Mutaner | | Finland | | | | Enontekioe | 68,99700165 | 20,7439994ε |
| MM04161 | 645 | HM386948 | Prodoxidae | Incurvariinae | Incurvaria vetulella | Zetterstedt | Marko Mutaner | | Finland | | | | Enontekioe | 68,99700165 | 20,7439994ε |
| MM08280 | 658 | HM873845 | Prodoxidae | Incurvariinae | Incurvaria vetulella | Zetterstedt | Marko Mutanen | | Finland | | Lapponia kemensis pars orientalis | | Sodankylae | 68,16999817 | 27,09600067 |
| MM13850 | 658 | HM387061 | Prodoxidae | Incurvariinae | Incurvaria vetulella | Zetterstedt | M. Mutaner | | Finland | | | | Sodankylae | 60,7159996 | 27,07299995 |
| TLMF Lep 02737 | 658 | JF860257 | Prodoxidae | Incurvariinae | Incurvaria vetulella | Zetterstedt | Schmid J. | 07-Jul-2010 | Switzerlanc | Graubunder | | | Guralaetsch/ Vals | 46,55500031 | 9,126000404 |
| TLMF Lep 02775 | 512 | JX034629 | Prodoxidae | Incurvariinae | Incurvaria vetulella | Zetterstedt | Huemer P | 14-Jul-1993 | Austria | Tyrol | Nordtiro | | Kuehta | 47,2140007 | 11,02600002 |
| TLMF Lep 02794 | 658 | JF860292 | Prodoxidae | Incurvariinae | Incurvaria vetulella | Zetterstedt | Deutsch H. | 17-Jul-2004 | Austria | Tyrol | Osttirol, Lienzer Dolomiten | | Innsteinalm/ Lienz SE | 46,77500153 | 12,79199982 |
| MM00490 | 658 | HM871032 | Yponomeutidae | | Kessleria fasciapennella | Stainton | Marko Mutanen | 22-May-2006 | Finland | | Ostrobottnia borealis pars australis | | Kiiminki | 65,07099915 | 25,72500038 |
| MM00865 | 658 | HM871227 | Yponomeutidae | | Kessleria fasciapennella | Stainton | Marko Mutanen | | Finland | | Ostrobottnia borealis pars australis | | Kiiminki | 65,07099915 | 25,72500038 |
| MM06404 | 658 | HM873232 | Yponomeutidae | | Kessleria fasciapennella | Stainton | Marko Mutanen, Nestori Mutanen | 09-Jun-2007 | Finland | | Regio kuusamoensis | | Kuusamo | 66,37599945 | 29,41500092 |
| TLMF Lep 01871 | 658 | JF859608 | Yponomeutidae | | Kessleria fasciapennella | Stainton | Tarmann G. | 31-Aug-1991 | Austria | Tyrol | Osttirol, Teischnitztal | | Teischnitztal/ Talausgang S (linker Talhang) | 47,02999878 | 12,63809967 |
| TLMF Lep 01872 | 658 | JF859609 | Yponomeutidae | | Kessleria fasciapennella | Stainton | Tarmann G. | 31-Aug-1991 | Austria | Tyrol | Osttirol, Teischnitztal | | Teischnitztal/ Talausgang S (linker Talhang) | 47,02999878 | 12,63809967 |
| TLMF Lep 01873 | 658 | JF859610 | Yponomeutidae | | Kessleria fasciapennella | Stainton | Tarmann G. | 31-Aug-1991 | Austria | Tyrol | Osttirol, Teischnitztal | | Teischnitztal/ Talausgang S (linker Talhang) | 47,02999878 | 12,63809967 |
| TLMF Lep 01874 | 658 | JF859611 | Yponomeutidae | | Kessleria fasciapennella | Stainton | Tarmann G. | 31-Aug-1991 | Austria | Tyrol | Osttirol, Teischnitztal | | Teischnitztal/ Talausgang S (linker Talhang) | 47,02999878 | 12,63809967 |
| MM07022 | 658 | HM873637 | Prodoxidae | Incurvariinae | Lampronia standfussiella | Zeller | Tomi Mutanen | 18-Jun-2005 | Finland | | Ostrobottnia borealis pars australis | | Rovaniemi | 66,41999817 | 26,32799911 |
| MM15518 | 658 | HM876315 | Prodoxidae | Incurvariinae | Lampronia standfussiella | Zeller | Tomi Mutanen | 18-Jun-2005 | Finland | | Ostrobottnia borealis pars borealis | | Rovaniemen mlk | 66,50499725 | 25,65200043 |
| MM18068 | 658 | JF854100 | Prodoxidae | Incurvariinae | Lampronia standfussiella | Zeller | Marko Mutanen | 17-Jun-1999 | Finland | | Ostrobottnia borealis pars australis | | Oulu | 64,97899628 | 25,51729965 |
| TLMF Lep 02738 | 658 | JF860258 | Prodoxidae | Incurvariinae | Lampronia standfussiella | Zeller | Schmid J. | 08-Jul-2009 | Switzerland | Graubunden | | | Laegh da l`Albignia, Val Bregaglia | 46,3370018 | 9,642999649 |
| TLMF Lep 02786 | 658 | JF860288 | Prodoxidae | Incurvariinae | Lampronia standfussiell | Zeller | Huemer P | 27-Jun-1987 | Italy | Lombardy | Giudicarie | | Monte Tremalzo | 45,8409996 | 10,69999981 |
| BC ZSM Lep 24138 | 658 | GU687262 | Geometridae | Larentiinae | Lampropteryx otregiata | Metcalfe | A. Haslberger | 25-May-2003 | Germany | Bavaria | Oberbayern | | Ruhpolding, Roethelmoc | | |
| BC ZSM Lep 24972 | 658 | GU654947 | Geometridae | Larentiinae | Lampropteryx otregiata | Metcalfe | Dr. Theo Gruenewald | 23-May-2009 | Germany | Bavaria | Niederbayern | Landshu | Kroening, Oberschnittenkofer | 48,5530014 | 12,38399982 |
| BC ZSM Lep 37470 | 658 | HQ563600 | Geometridae | Larentiinae | Lampropteryx otregiata | Metcalfe | Dr. Andreas H. Segerer | 15-Aug-2009 | Germany | Bavaria | Niederbayern | Straubing-Boger | Wiesenfelden, Hoehenberg | 49,04449844 | 12,49730015 |
| MM01449 | 658 | HM386734 | Geometridae | Larentiinae | Lampropteryx otregiata | Metcalfe | Marko Mutanen, Panu Vaelimael | | Finland | | | | Imatra | 61,10800171 | 28,79899979 |
| MM01475 | 658 | HM386755 | Geometridae | Larentiinae | Lampropteryx otregiata | Metcalfe | Marko Mutanen, Panu Vaelimael | | Finland | | | | Imatra | 61,10800171 | 28,79899979 |
| MM01476 | 658 | HM386756 | Geometridae | Larentiinae | Lampropteryx otregiata | Metcalfe | Marko Mutanen, Panu Vaelimael | | Finland | | | | Imatra | 61,10800171 | 28,79899979 |
| MM02883 | 658 | HM871736 | Geometridae | Larentiinae | Lampropteryx otregiata | Metcalfe | Marko Mutanen | | Finland | | Ostrobottnia borealis pars australis | | Kiiminki | 65,07099915 | 25,72500038 |
| MM14769 | 658 | HM876251 | Elachistidae | Depressariinae | Levipalpus hepatariell | Lienig & Zelle | Petri Hirvoner | 01-Aug-2009 | Finland | | Savonia australis | | Imatra | 61,2060012ε | 28,86000061 |
| MM14770 | 658 | HM876252 | Elachistidae | Depressariinae | Levipalpus hepatariell | Lienig & Zelle | Petri Hirvoner | 01-Aug-2009 | Finland | | Savonia australis | | Imatra | 61,2060012ε | 28,86000061 |
| MM17543 | 658 | JX034617 | Elachistidae | Depressariinae | Levipalpus hepatariell | Lienig & Zelle | Juhani Itaemies | | Finland | | Kuusamo | | Kuusamo | 66,36799622 | 29,30200005 |
| TLMF Lep 02739 | 658 | JF860259 | Elachistidae | Depressariinae | Levipalpus hepatariell | Lienig & Zelle | Schmid J. | 22-Aug-2010 | Switzerlanc | Graubunder | | | Lagalb | 46,43600082 | 9,987000465 |
| TLMF Lep 03713 | 658 | JN266666 | Elachistidae | Depressariinae | Levipalpus hepatariell | Lienig & Zelle | Erlebach S. | 16-Aug-1993 | Austria | Tyrol | Nordtiro | Wattenta | Walchen/ Hintertux NW | 47,20830154 | 11,62510014 |
| TLMF Lep 04793 | 658 | JX034570 | Elachistidae | Depressariinae | Levipalpus hepatariell | Lienig & Zelle | Sonderegger P. | 09-Sep-2008 | Switzerlanc | Valais | | | Fully, Lousine | 46,17699814 | 7,127850056 | genitalia on card boa |
| BC ZSM Lep 24247 | 658 | GU687173 | Geometridae | Ennominae | Macaria fusca | Thunberg | A. Haslberger | 12-Aug-2000 | Austria | Karnten | | | Grossglockner, Obernassfel | 47,11809921 | 12,82759953 |
| MM03420 | 658 | HQ570295 | Geometridae | Ennominae | Macaria fusca | Thunberg | Marko Mutaner | | Finland | Lappi | Lapponia enontekiensi | | Enontekioe | 68,99700165 | 20,7439994ε |
| MM14587 | 658 | HM876177 | Geometridae | Ennominae | Macaria fusca | Thunberg | Marko Mutaner | | Finland | | Lapponia enontekiensi | | Enontekioe | 69,06300354 | 21,1019992ε |
| MM18508 | 658 | JF854375 | Geometridae | Ennominae | Macaria fusca | Thunberg | Marko Mutanen, Nestori Mutanen, Anttoni Mutaner | 11-Jul-2010 | Finland | | Lapponia inarensis | | Utsjoki | 69,37689972 | 25,98279953 |
| TLMF Lep 00449 | 658 | HM425925 | Geometridae | Ennominae | Macaria fusca | Thunberg | Huemer P. | 25-Jul-2009 | France | Provence-Alpes-Cote d'Azur | | | Col de la Moutiere W/ W St. Dalmas le Selvag | 44,32030106 | 6,795559883 |
| TLMF Lep 00450 | 658 | HM425926 | Geometridae | Ennominae | Macaria fusca | Thunberg | Huemer P. | 25-Jul-2009 | France | Provence-Alpes-Cote d'Azur | | | Col de la Moutiere W/ W St. Dalmas le Selvag | 44,32030106 | 6,795559883 |
| TLMF Lep 02740 | 658 | JF860260 | Geometridae | Ennominae | Macaria fusca | Thunberg | Schmid J. | 07-Aug-2009 | Switzerlanc | Graubunder | | | Vorab pign, Grat/ Laax | 46,8610000є | 9,16600036є |
| TLMF Lep 02909 | 658 | JF860360 | Geometridae | Ennominae | Macaria fusca | Thunberg | Mayr T. | 22-Jul-2010 | Switzerlanc | Graubunder | | | Alpe Plavna/ Taras | 46,7000007є | 10,25 |
| TLMF Lep 02910 | 658 | JX034584 | Geometridae | Ennominae | Macaria fusca | Thunberg | Huemer P. | 04-Aug-2010 | France | Provence-Alpes-Cote d'Azur | | | Col Agnel | 44,68899918 | 6,984000206 |
| TLMF Lep 02911 | 658 | JX034602 | Geometridae | Ennominae | Macaria fusca | Thunberg | Huemer P. | 04-Aug-2010 | France | Provence-Alpes-Cote d'Azur | | | Col Agnel | 44,68899918 | 6,984000206 |
| TLMF Lep 05524 | 658 | JX034625 | Geometridae | Ennominae | Macaria fusca | Thunberg | Huemer P. & Tarmann G. M. | 28-Jul-2011 | Macedonia | | | | Mavrovo NP, Korab, summit ridge | 41,78900146 | 20,54700089 |

| | | | | | | | | | | | | | | | | |
|---|---|---|---|---|---|---|---|---|---|---|---|---|---|---|---|---|
| TLMF Lep 05525 | 658 | JX034658 | Geometridae | Ennominae | Macaria fusca | Thunberg | Huemer P. & Tarmann G. M. | 28-Jul-2011 | Macedonia | | | | Mavrovo NP, Korab, summit ridge | 41,78900146 | 20,54700089 | |
| BC ZSM Lep 24200 | 658 | GU687203 | Geometridae | Larentiinae | Martania taeniata | Stephens | A. Haslberger | 13-Jul-2005 | Germany | Bavaria | Oberbayern | Berchtesgadener Land | Ramsau bei Berchtesgaden, Klausbachta | 47,58060074 | 12,81550026 | |
| MM01448 | 658 | HM386733 | Geometridae | Larentiinae | Martania taeniata | Stephens | Marko Mutanen, Panu Vaelimael | | Finland | | | | Imatra | 61,10800171 | 28,79899979 | |
| MM01764 | 647 | HM870896 | Geometridae | Larentiinae | Martania taeniata | Stephens | Marko Mutanen, Panu Vaelimael | | Finland | | | | Imatra | 61,10800171 | 28,79899979 | |
| MM08468 | 658 | HM873926 | Geometridae | Larentiinae | Martania taeniata | Stephens | Marko Mutanen, Panu Vaelimaeki | | Finland | | Lapponia kemensis pars occidentalis | | Kolari | 67,27600098 | 23,75300026 | |
| MM10552 | 658 | HM874804 | Crambidae | Odontiinae | Metaxmeste schrankiana | Hochenwarth | Marko Mutanen | 27-May-2002 | Finland | | Ostrobottnia borealis pars australis | | Utajaervi | 65,04199982 | 23,81399918 | |
| MM10553 | 658 | HM874805 | Crambidae | Odontiinae | Metaxmeste schrankiana | Hochenwarth | Marko Mutanen | 27-May-2002 | Finland | | Ostrobottnia borealis pars australis | | Utajaervi | 65,04199982 | 23,81399918 | |
| MM13925 | 658 | HM875799 | Crambidae | Odontiinae | Metaxmeste schrankiana | Hochenwarth | Marko Mutanen, Nestori Mutanen, Anttoni Mutanen | 30-May-2009 | Finland | | Ostrobottnia borealis pars australis | | Kiiminki | 65,07099915 | 25,72500038 | |
| MM18800 | 658 | JF854629 | Crambidae | Odontiinae | Metaxmeste schrankian | Hochenwarth | Reima Leinonen | 03-Jun-2007 | Finland | | Ostrobottnia kajanensi | | Suomussalm | 65,02200317 | 29,33399963 | |
| TLMF Lep 01775 | 658 | HQ968975 | Crambidae | Odontiinae | Metaxmeste schrankiana | Hochenwarth | Huemer P. | 01-Jul-2008 | France | Provence-Alpes-Cote d'Azur | | | Mont Chajol SE-Seite/ CastUrino E | 44,09590149 | 7,533609867 | |
| TLMF Lep 01776 | 641 | HQ968976 | Crambidae | Odontiinae | Metaxmeste schrankiana | Hochenwarth | Huemer P. | 07-May-2008 | Italy | | Cuneo, Massiccio Argentera | | Gias del Chiot/ PalanfrP SW | 44,19499969 | 7,489719868 | |
| TLMF Lep 01782 | 658 | HQ968982 | Crambidae | Odontiinae | Metaxmeste schrankian | Hochenwarth | Erlebach S. | 29-Jul-2004 | Austria | Tyrol | Nordtirol, Nordkette | | Langer Sattel/ Innsbruck NW | 47,31069946 | 11,35879993 | |
| TLMF Lep 01793 | 658 | HQ968992 | Crambidae | Odontiinae | Metaxmeste schrankiana | Hochenwarth | Huemer P. | 01-Jul-2008 | France | Provence-Alpes-Cote d'Azur | | | Mont Chajol SE-Seite/ CastUrino E | 44,09590149 | 7,533609867 | |
| TLMF Lep 02743 | 658 | JF860262 | Crambidae | Odontiinae | Metaxmeste schrankian | Hochenwarth | Schmid J. | 27-Jul-2007 | Switzerland | Graubunden | | | Val de la Prasgnola/ Aver | 46,36999893 | 9,50399971 | |
| 07PROBE-00535 | 653 | JX008403 | Gelechiidae | Gelechiinae | Neofaculta infernella | Herrich-Schäffer | J.deWaard | 15-Jul-2007 | Canada | Manitoba | Churchill | 16 km E Churchill, Bird Cove, Rock Bluff C | Tundra flat nr. rock bluff C | 58,76499939 | -93,86499786 | MIC5557 M |
| 07PROBE-10832 | 658 | JX008139 | Gelechiidae | Gelechiinae | Neofaculta infernella | Herrich-Schäffer | P.D.N. Hebert | 23-Jul-2007 | Canada | Manitoba | Churchill | 26 km SE Churchill, Twin Lake | | 58,63000107 | -93,81900024 | |
| 07PROBE-10833 | 658 | JX008290 | Gelechiidae | Gelechiinae | Neofaculta infernella | Herrich-Schäffer | P.D.N. Hebert | 23-Jul-2007 | Canada | Manitoba | Churchill | 26 km SE Churchill, Twin Lake | | 58,63000107 | -93,81900024 | MIC5558 M |
| MM02323 | 626 | HM871485 | Gelechiidae | Gelechiinae | Neofaculta infernella | Herrich-Schäffer | Marko Mutanen, Panu Vaelimael | | Finland | | Savonia australis | | Imatra | 61,10800171 | 28,79899979 | |
| MM03437 | 658 | HQ570300 | Gelechiidae | Gelechiinae | Neofaculta infernella | Herrich-Schäffer | Marko Mutanen | | Finland | Lappi | Lapponia enontekiensi | | Enontekioe | 68,99700165 | 20,74399948 | |
| MM04223 | 658 | HM872338 | Gelechiidae | Gelechiinae | Neofaculta infernella | Herrich-Schäffer | Marko Mutanen | 13-Jun-2006 | Finland | Etela-Suomen Laani | Karelia ladogensis | | Parikkala | 61,50799942 | 29,5739994 | |
| MM05472 | 658 | HM872844 | Gelechiidae | Gelechiinae | Neofaculta infernella | Herrich-Schäffer | Marko Mutanen, Nestori Mutanen, Anttoni Mutanen | 04-Jun-2007 | Finland | | Ostrobottnia borealis pars australis | | Kiiminki | 65,07099915 | 25,72500038 | |
| MM09029 | 658 | JX034600 | Gelechiidae | Gelechiinae | Neofaculta infernella | Herrich-Schäffer | Marko Mutanen, Panu Vaelimael | | Finland | | Larelia lagodensis | | Simpele | 61,42300034 | 29,32299995 | |
| MM13507 | 655 | HM875629 | Gelechiidae | Gelechiinae | Neofaculta infernella | Herrich-Schäffer | Marko Mutanen, Panu Vaelimael | | Finland | | Regio Aboensis | | Dragsfjaerd | 60,01100159 | 22,49799919 | |
| MM13508 | 658 | JX034649 | Gelechiidae | Gelechiinae | Neofaculta infernella | Herrich-Schäffer | Marko Mutanen, Panu Vaelimael | | Finland | | Regio Aboensis | | Dragsfjaerd | 60,01100159 | 22,49799919 | |
| MM17906 | 658 | JX034666 | Gelechiidae | Gelechiinae | Neofaculta infernella | Herrich-Schäffer | Ali Karhu | | Finland | | Karelia borealis | | Liperi | 62,54859924 | 29,09910011 | |
| MM17907 | 658 | JX034662 | Gelechiidae | Gelechiinae | Neofaculta infernella | Herrich-Schäffer | Ali Karhu | | Finland | | Karelia borealis | | Liperi | 62,54859924 | 29,09910011 | |
| MM17908 | 637 | JX034580 | Gelechiidae | Gelechiinae | Neofaculta infernella | Herrich-Schäffer | Ali Karhu | | Finland | | Karelia borealis | | Liperi | 62,54859924 | 29,09910011 | |
| MM18249 | 658 | JF854242 | Gelechiidae | Gelechiinae | Neofaculta infernella | Herrich-Schäffer | Marko Mutanen, Nestori Mutanen, Anttoni Mutanen | 18-Jun-2010 | Finland | | Savonia australis | | Lappeenranta | 61,05490112 | 28,72179985 | |
| MM18250 | 658 | JF854243 | Gelechiidae | Gelechiinae | Neofaculta infernella | Herrich-Schäffer | Tomi Mutanen | 19-Jun-2004 | Finland | | Ostrobottnia kajanensi | | Kajaan | 64,26750183 | 27,61899948 | |
| MM18251 | 658 | JF854244 | Gelechiidae | Gelechiinae | Neofaculta infernella | Herrich-Schäffer | Marko Mutanen, Nestori Mutanen, Anttoni Mutanen | 08-Jul-2010 | Finland | | Lapponia inarensis | | Utsjoki | 69,82800293 | 27 | |
| MM19600 | 658 | JN270928 | Gelechiidae | Gelechiinae | Neofaculta infernella | Herrich-Schäffer | H. Hendriksen | 05-Jul-1994 | Denmark | | Jylland | | Danner ho | | | |
| TLMF Lep 00545 | 657 | HM381388 | Gelechiidae | Gelechiinae | Neofaculta infernella | Herrich-Schäffer | Huemer P. | 13-Jul-2009 | Austria | Karnten | | | Petzen N, Umg. Gh. Siebenhuetten | 46,51819992 | 14,77350044 | |
| TLMF Lep 00592 | 658 | HM381434 | Gelechiidae | Gelechiinae | Neofaculta infernella | Herrich-Schäffer | Huemer P. | 20-Jul-2009 | Italy | | Belluno | | Passo di Valparola E - Passo Falzarego | 46,5223999 | 12,00699997 | |
| TLMF Lep 00830 | 657 | HQ968206 | Gelechiidae | Gelechiinae | Neofaculta infernella | Herrich-Schäffer | Huemer P | 01-Jul-2009 | Austria | Karnten | | | Umg. Zollnerseehuett | 46,60599899 | 13,0710001 | |
| TLMF Lep 00867 | 658 | HQ968241 | Gelechiidae | Gelechiinae | Neofaculta infernella | Herrich-Schäffer | Huemer P. | 20-Jun-2008 | Austria | Vorarlberg | | | Sonntag, Buchboden, NW Bad Rotenbrunnen | 47,24499893 | 9,970999718 | |
| TLMF Lep 00913 | 658 | HM381485 | Gelechiidae | Gelechiinae | Neofaculta infernella | Herrich-Schäffer | Huemer P. | 29-May-2009 | Austria | Vorarlberg | | | Sonntag, Buchboden, S Unter-Ueberlutalpe | 47,25099945 | 9,970999718 | |
| TLMF Lep 02061 | 658 | JF859652 | Gelechiidae | Gelechiinae | Neofaculta infernella | Herrich-Schäffer | Huemer P | 01-Jul-2010 | Italy | South Tyro | | | Ritten/ Obergruenwal | 46,59700012 | 11,43900013 | |
| TLMF Lep 02167 | 615 | JF859751 | Gelechiidae | Gelechiinae | Neofaculta infernella | Herrich-Schäffer | Huemer P | 04-Jun-2010 | Italy | South Tyro | Etschta | | Montiggl/ Kleiner Pric | 46,4280014 | 11,30000019 | |
| TLMF Lep 02218 | 658 | JF859800 | Gelechiidae | Gelechiinae | Neofaculta infernella | Herrich-Schäffer | Huemer P | 22-Jul-2010 | Italy | South Tyro | Etschta | | Montiggl/ Kleiner Pric | 46,4280014 | 11,30000019 | |
| TLMF Lep 02255 | 658 | JF859830 | Gelechiidae | Gelechiinae | Neofaculta infernella | Herrich-Schäffer | Huemer P | 30-Jun-2010 | Italy | South Tyro | Etschta | | Montiggl/ Kleiner Pric | 46,4280014 | 11,30000019 | |
| TLMF Lep 02745 | 627 | JF860264 | Gelechiidae | Gelechiinae | Neofaculta infernella | Herrich-Schäffer | Schmid J. | 22-Jun-2005 | Switzerland | Graubunden | | | Riefawald/ Vals | 46,61800003 | 9,194000244 | |
| TLMF Lep 00432 | 658 | HM425911 | Nymphalidae | Satyrinae | Oeneis glacialis | Mol | Huemer P | 23-Jul-2009 | Italy | | Cuneo | | Gias Valcavera/ Demonte NW | 44,37670135 | 7,136670113 | |
| TLMF Lep 01087 | 658 | HQ968455 | Nymphalidae | Satyrinae | Oeneis glacialis | Moll | Aistleitner U. | 13-Jun-2009 | Switzerland | Graubunden | | | Tiefencastel S/ Salouf, Got Grond | 46,65000153 | 9,597000122 | |
| MM00092 | 658 | HM396371 | Nymphalidae | Satyrinae | Oeneis norna | Thunberg | Marko Mutanen | | Finland | | | | Enontekioe | 68,99700165 | 20,74399948 | |
| MM03405 | 658 | HQ570284 | Nymphalidae | Satyrinae | Oeneis norna | Thunberg | Marko Mutanen | | Finland | Lappi | Lapponia enontekiensi | | Enontekioe | 68,99700165 | 20,74399948 | |
| MM04099 | 658 | HM386889 | Nymphalidae | Satyrinae | Oeneis norna | Thunberg | Marko Mutanen | | Finland | | | | Enontekioe | 68,99700165 | 20,74399948 | |
| MM06393 | 658 | HM873225 | Pterophoridae | Pterophorinae | Oidaematophorus rogenhoferi | Mann | Marko Mutanen, Panu Vaelimaeki, Jani Raitanen, Heikki Poeykkoe | | Finland | | Ostrobottnia borealis pars australis | | Tornio | 65,85099792 | 24,35000038 | |
| MM06582 | 658 | HM873344 | Pterophoridae | Pterophorinae | Oidaematophorus rogenhoferi | Mann | Marko Mutanen, Panu Vaelimaeki, Heikki Poeykkoe | | Finland | | Ostrobottnia borealis pars australis | | Tornio | 65,85099792 | 24,35000038 | |
| MM06583 | 658 | HM873345 | Pterophoridae | Pterophorinae | Oidaematophorus rogenhoferi | Mann | Marko Mutanen, Panu Vaelimaeki, Heikki Poeykkoe | | Finland | | Ostrobottnia borealis pars australis | | Tornio | 65,85099792 | 24,35000038 | |
| TLMF Lep 00578 | 658 | HM381420 | Pterophoridae | Pterophorinae | Oidaematophorus rogenhoferi | Mann | Huemer P. | 20-Jul-2009 | Italy | | Belluno | | Passo di Valparola E - Passo Falzarego | 46,5223999 | 12,00699997 | |
| TLMF Lep 01715 | 658 | HQ968919 | Pterophoridae | Pterophorinae | Oidaematophorus rogenhofe | Mann | Huemer P | 09-Aug-2010 | Slovenia | | | | Veliki Mangar | 46,43849945 | 13,63529968 | |
| TLMF Lep 01716 | 658 | HQ968920 | Pterophoridae | Pterophorinae | Oidaematophorus rogenhofe | Mann | Huemer P | 09-Aug-2010 | Slovenia | | | | Veliki Mangar | 46,43849945 | 13,63529968 | |
| TLMF Lep 02746 | 658 | JF860265 | Pterophoridae | Pterophorinae | Oidaematophorus rogenhofe | Mann | Schmid J. | 04-Aug-2009 | Switzerland | Graubunden | | | Ofenpass - Piz Dain | 46,63299942 | 10,2840004 | |
| 09PROBE-09314 | 658 | HM375570 | Tortricidae | Olethreutinae | Olethreutes schulziana | Fabricius | P.D.N. Hebert | 26-Jul-2009 | Canada | Manitoba | Churchill | 26 km SE Churchill, Twin Lakes burn site | | 58,61999893 | -93,83000183 | |
| 09PROBE-09469 | 658 | HM375723 | Tortricidae | Olethreutinae | Olethreutes schulzian | Fabricius | P.D.N. Hebert | 28-Jul-2009 | Canada | Manitoba | Churchill | 16 km E Churchill, tundra pond | | 58,75500107 | -93,91500092 | |
| 09PROBE-09473 | 658 | HM375727 | Tortricidae | Olethreutinae | Olethreutes schulzian | Fabricius | P.D.N. Hebert | 28-Jul-2009 | Canada | Manitoba | Churchill | 16 km E Churchill, tundra pond | | 58,75500107 | -93,91500092 | |
| MM03787 | 658 | HM872147 | Tortricidae | Olethreutinae | Olethreutes schulziana | Fabricius | Marko Mutanen | 29-Jun-2006 | Finland | Oulun laani | Ostrobottnia borealis pars australis | | Kiiminki | 64,98100281 | 25,72900009 | |
| MM04118 | 658 | HM386907 | Tortricidae | Olethreutinae | Olethreutes schulzian | Fabricius | Marko Mutanen | | Finland | | | | Enontekioe | 68,99700165 | 20,74399948 | |
| MM04119 | 658 | HM386908 | Tortricidae | Olethreutinae | Olethreutes schulzian | Fabricius | Marko Mutanen | | Finland | | | | Enontekioe | 68,99700165 | 20,74399948 | |
| MM04120 | 658 | HM386909 | Tortricidae | Olethreutinae | Olethreutes schulzian | Fabricius | Marko Mutanen | | Finland | | | | Enontekioe | 68,99700165 | 20,74399948 | |
| MM06203 | 658 | HM873115 | Tortricidae | Olethreutinae | Olethreutes schulzian | Fabricius | Marko Mutanen, Tomi Mutane | 16-Jun-2007 | Finland | | Karelia borealis | | Kesaelaht | 61,79299927 | 29,71100044 | |
| MM06286 | 658 | HM873172 | Tortricidae | Olethreutinae | Olethreutes schulziana | Fabricius | Marko Mutanen, Nestori Mutanen, Anttoni Mutanen | 27-Jun-2007 | Finland | | Lapponia kemensis pars orientalis | | Sodankylae | 68,22299957 | 27,21699905 | |

| | | | | | | | | | | | | | | | | |
|---|---|---|---|---|---|---|---|---|---|---|---|---|---|---|---|---|
| MM06311 | 658 | HM873193 | Tortricidae | Olethreutinae | Olethreutes schulziana | Fabricius | Marko Mutanen, Anttoni Mutanen, Nestori Mutanen | 28-Jun-2006 | Finland | | Lapponia inarensis | | Inari | 68,4469986 | 27,41399956 | |
| MM06351 | 658 | HM873219 | Tortricidae | Olethreutinae | Olethreutes schulziana | Fabricius | Marko Mutanen, Anttoni Mutanen, Nestori Mutanen | 29-Jun-2007 | Finland | | Lapponia inarensis | | Utsjoki | 69,89099884 | 27,07799911 | |
| MM08474 | 658 | JX034686 | Tortricidae | Olethreutinae | Olethreutes schulziana | Fabricius | Marko Mutanen, Panu Vaelimaeki | | Finland | | Lapponia kemensis pars occidentalis | | Kolari | 67,27600098 | 23,75300026 | |
| MM08475 | 658 | JX034661 | Tortricidae | Olethreutinae | Olethreutes schulziana | Fabricius | Marko Mutanen, Panu Vaelimaeki | | Finland | | Lapponia kemensis pars occidentalis | | Kolari | 67,27600098 | 23,75300026 | |
| MM14581 | 658 | JX034593 | Tortricidae | Olethreutinae | Olethreutes schulzian | Fabricius | Marko Mutanen | | Finland | | Lapponia enontekiensi | | Enontekioe | 69,06300354 | 21,10199928 | |
| MM14582 | 658 | JX034636 | Tortricidae | Olethreutinae | Olethreutes schulzian | Fabricius | Marko Mutanen | | Finland | | Lapponia enontekiensi | | Enontekioe | 69,06300354 | 21,10199928 | |
| MM18289 | 658 | JF854277 | Tortricidae | Olethreutinae | Olethreutes schulziana | Fabricius | Marko Mutanen, Nestori Mutanen, Anttoni Mutanen | 08-Jul-2010 | Finland | | Lapponia inarensis | | Utsjoki | 69,82800293 | 27 | |
| MM18290 | 658 | JF854278 | Tortricidae | Olethreutinae | Olethreutes schulzian | Fabricius | Marko Mutanen | 03-Jul-2003 | Finland | | Kuusamo | | Kuusamo | 66,31220245 | 29,4531002 | |
| MM18291 | 658 | JF854279 | Tortricidae | Olethreutinae | Olethreutes schulziana | Fabricius | Marko Mutanen | 21-Jun-2005 | Finland | | Ostrobottnia borealis pars australis | | Kiiminki | 65,07060242 | 25,72480011 | |
| MM19640 | 658 | JN274930 | Tortricidae | Olethreutinae | Olethreutes schulzian | Fabricius | H. Hendriksen | 04-Aug-2003 | Denmark | | Nwz | Sjaelland | Karupho | | | |
| NoA-08-325 | 609 | JX008477 | Tortricidae | Olethreutinae | Olethreutes schulziana | Fabricius | D.A. Macaulay, G.R. Pohl, A.D. Roe | 09-Jul-2001 | Canada | Alberta | | | La Butte Ck Wildlands Prov Pk | 59,36500168 | -111,1299973 | |
| NoA-08-326 | 658 | JX008173 | Tortricidae | Olethreutinae | Olethreutes schulzian | Fabricius | | 16-Jun-2001 | Canada | Alberta | | | Ft McMurray | 56,56000137 | -111,3099976 | |
| NoA-08-327 | 658 | JX008195 | Tortricidae | Olethreutinae | Olethreutes schulzian | Fabricius | | 02-Jul-2005 | Canada | Alberta | | | Ft McMurray | 56,93399811 | -111,5279999 | |
| NoA-08-328 | 658 | JX008250 | Tortricidae | Olethreutinae | Olethreutes schulzian | Fabricius | | 02-Jul-2005 | Canada | Alberta | | | Ft McMurray | 56,93399811 | -111,5279999 | |
| NoA-08-329 | 658 | JX008371 | Tortricidae | Olethreutinae | Olethreutes schulziana | Fabricius | D.A. Macaulay, G.R. Pohl, A.D. Roe | 06-Jul-2001 | Canada | Alberta | | | La Butte Ck Wildlands Prov Pk | 59,42699814 | -111,4440002 | |
| TLMF Lep 00826 | 658 | HQ968202 | Tortricidae | Olethreutinae | Olethreutes schulzian | Fabricius | Huemer P | 01-Jul-2009 | Austria | Karnten | | | Umg. Zollnerseehuett | 46,60599899 | 13,0710001 | |
| TLMF Lep 01795 | 658 | HQ968994 | Tortricidae | Olethreutinae | Olethreutes schulzian | Fabricius | Huemer P | 15-Jul-2009 | Austria | Steiermark | | | Woerschacher Moos NW | 47,55780029 | 14,17059994 | |
| TLMF Lep 04475 | 658 | JN275067 | Tortricidae | Olethreutinae | Olethreutes schulzian | Fabricius | Huemer P | 14-Aug-2010 | Austria | | Nordtiro | | Walchsee/ Schwemm SE | 47,65639877 | 12,3032999 | |
| 08BBLEP-02684 | 268 | JX008159 | Papilionidae | Parnassiinae | Parnassius phoebus | Fabricius | J.Straka,J.Cossey | 06-Aug-2008 | Canada | Alberta | Waterton | Waterton Lakes Nat. Park - Lineham | Subalpine meadow | 49,06600189 | -113,9980011 | |
| TLMF Lep 02761 | 658 | JF860278 | Papilionidae | Parnassiinae | Parnassius phoebus | Fabricius | Schmid J. | 10-Jul-2003 | Switzerland | Graubunden | | | Alp Rondadura/ Medel Lu | 46,56999969 | 8,774999619 | |
| TLMF Lep 02967 | 658 | JF860400 | Papilionidae | Parnassiinae | Parnassius phoebus | Fabricius | Aistleitner E | 03-Aug-2002 | Switzerland | Graubunden | | | Juf/ Avers | 46,4469986 | 9,579999924 | |
| TLMF Lep 02968 | 658 | JF860401 | Papilionidae | Parnassiinae | Parnassius phoebus | Fabricius | Aistleitner E | 03-Aug-2002 | Switzerland | Graubunden | | | Juf/ Avers | 46,4469986 | 9,579999924 | |
| BC ZSM Lep 24205 | 658 | HM376823 | Geometridae | Larentiinae | Perizoma minorata | Treitschke | A. Haslberger | 17-Jul-2006 | Germany | Bavaria | Oberbayern | Berchtesgadener Land | Lattengebirge, Baumgarten/Roetelbac | 47,68909836 | 12,86270046 | |
| MM00090 | 658 | HM396369 | Geometridae | Larentiinae | Perizoma minorata | Treitschke | Marko Mutanen | | Finland | | | | Enontekioe | 68,99700165 | 20,74399948 | |
| MM04107 | 658 | HM386897 | Geometridae | Larentiinae | Perizoma minorata | Treitschke | Marko Mutanen | | Finland | | | | Enontekioe | 68,99700165 | 20,74399948 | |
| MM06363 | 658 | HQ570329 | Geometridae | Larentiinae | Perizoma minorata | Treitschke | Marko Mutanen, Anttoni Mutanen, Nestori Mutanen | 29-Jun-2007 | Finland | | Lapponia inarensis | | Utsjoki | 69,37599945 | 25,80500031 | |
| TLMF Lep 00315 | 658 | HM381356 | Geometridae | Larentiinae | Perizoma minorata | Treitschke | Huemer P. | 16-Jul-2009 | Austria | Vorarlberg | | | Marul, Lagutzbach, Beim alten Stafel | 47,20220184 | 9,928059578 | |
| TLMF Lep 02747 | 658 | JF860266 | Geometridae | Larentiinae | Perizoma minorata | Treitschke | Schmid J. | 24-Jul-2005 | Switzerland | Graubunden | | | Riefawald/ Vals | 46,61800003 | 9,194000244 | |
| TLMF Lep 05552 | 658 | JX034681 | Geometridae | Larentiinae | Perizoma minorata | Treitschke | Huemer P. & Tarmann G. M. | 28-Jul-2011 | Macedonia | | | | Mavrovo NP, Korab, Korabska jezero, Kobilino pole | 41,77799988 | 20,58200073 | |
| TLMF Lep 05553 | 658 | JX034696 | Geometridae | Larentiinae | Perizoma minorata | Treitschke | Huemer P. & Tarmann G. M. | 28-Jul-2011 | Macedonia | | | | Mavrovo NP, Korab, Korabska jezero, Kobilino pole | 41,77799988 | 20,58200073 | |
| MM04194 | 550 | HQ570313 | Tortricidae | Olethreutinae | Phiaris septentrionana | Curtis | Marko Mutanen | | Finland | Lappi | Lapponia enontekiensi | | Enontekioe | 68,99700165 | 20,74399948 | MM932 |
| MM06395 | 658 | HM873227 | Tortricidae | Olethreutinae | Phiaris septentrionana | Curtis | Marko Mutanen, Anttoni Mutanen, Nestori Mutanen | 30-Jun-2007 | Finland | | Lapponia kemensis pars orientalis | | Pelkosenniemi | 67,15499878 | 27,7840004 | |
| MM15687 | 658 | HQ570412 | Tortricidae | Olethreutinae | Phiaris septentrionana | Curtis | Tomi Mutanen | 01-Jul-2003 | Finland | | Lapponia enontekiensi | | Enontekioe | 68,99700165 | 20,74399948 | |
| MM18292 | 658 | JF854280 | Tortricidae | Olethreutinae | Phiaris septentrionana | Curtis | Marko Mutanen | 27-Jun-2002 | Finland | | Lapponia inarensis | | Inari | 69,28559875 | 25,73390007 | MM931 |
| MM18293 | 658 | JF854281 | Tortricidae | Olethreutinae | Phiaris septentrionana | Curtis | Marko Mutanen, Nestori Mutanen, Anttoni Mutanen | 10-Jul-2010 | Finland | | Lapponia inarensis | | Utsjoki | 69,82800293 | 27 | |
| MM18294 | 658 | JF854282 | Tortricidae | Olethreutinae | Phiaris septentrionana | Curtis | Marko Mutanen, Nestori Mutanen, Anttoni Mutanen | 10-Jul-2010 | Finland | | Lapponia inarensis | | Utsjoki | 69,82800293 | 27 | MM930 |
| MM18295 | 658 | JF854283 | Tortricidae | Olethreutinae | Phiaris septentrionana | Curtis | Marko Mutanen | 03-Jul-2003 | Finland | | Kuusamo | | Kuusamo | 66,31220245 | 29,4531002 | |
| MM18297 | 658 | JF854284 | Tortricidae | Olethreutinae | Phiaris septentrionana | Curtis | Marko Mutanen, Nestori Mutanen, Anttoni Mutanen | 10-Jul-2010 | Finland | | Lapponia inarensis | | Utsjoki | 69,82800293 | 27 | MM933 |
| MM18299 | 656 | JF854286 | Tortricidae | Olethreutinae | Phiaris septentrionana | Curtis | Marko Mutanen | 30-Jun-2000 | Finland | | Ostrobottnia borealis pars australis | | Pudasjaervi | 65,70240021 | 27,65309906 | |
| MM18641 | 622 | JN274981 | Tortricidae | Olethreutinae | Phiaris septentrionana | Curtis | Panu Vaelimaek | 07-Jul-2003 | Finland | | Lapponia enontekiensi | | Enontekioe | 69,04560089 | 20,85540009 | |
| 07PROBE-10062 | 658 | JX008146 | Tortricidae | Olethreutinae | Phiaris turfosana | Herrich-Schäffer | P.D.N. Hebert | 16-Jul-2007 | Canada | Manitoba | Churchill | 13 km E Churchill, Eastern Creek | | 58,75500107 | -93,94400024 | |
| 09PROBE-09472 | 658 | HM375726 | Tortricidae | Olethreutinae | Phiaris turfosana | Herrich-Schäffer | P.D.N. Hebert | 28-Jul-2009 | Canada | Manitoba | Churchill | 16 km E Churchill, tundra pond | | 58,75500107 | -93,91500092 | |
| 09PROBE-09554 | 658 | HM375805 | Tortricidae | Olethreutinae | Phiaris turfosana | Herrich-Schäffer | S.Gillespie | 21-Jul-2009 | Canada | Manitoba | Churchill | 9 km S Churchill, Goose Creek Cabin Bog | | 58,69200134 | -94,13200378 | |
| MM03228 | 658 | HM871888 | Tortricidae | Olethreutinae | Phiaris turfosana | Herrich-Schäffer | Marko Mutanen | 29-Jun-2006 | Finland | Oulun laani | Ostrobottnia borealis pars australis | | Kiiminki | 65,07099915 | 25,72500038 | |
| MM13995 | 658 | HM875829 | Tortricidae | Olethreutinae | Phiaris turfosana | Herrich-Schäffer | Marko Mutanen | 11-Jun-2009 | Finland | | Ostrobottnia borealis pars australis | | Kempele | 64,8809967 | 25,58600044 | |
| MM18296 | 630 | JX034664 | Tortricidae | Olethreutinae | Phiaris turfosana | Herrich-Schäffer | Marko Mutanen | 12-Jul-2003 | Finland | | Kuusamo | | Kuusamo | 66,31559753 | 29,2303009 | |
| MM18298 | 658 | JF854285 | Tortricidae | Olethreutinae | Phiaris turfosana | Herrich-Schäffer | Marko Mutanen | 09-Jun-2005 | Finland | | Ostrobottnia borealis pars australis | | Kiiminki | 65,07060242 | 25,72480011 | |
| MM04162 | 658 | HM386949 | Tortricidae | Tortricinae | Phtheochroa vulneratan | Zetterstedt | Marko Mutanen | | Finland | | | | Enontekioe | 68,99700165 | 20,74399948 | |
| MM04163 | 658 | HM386950 | Tortricidae | Tortricinae | Phtheochroa vulneratan | Zetterstedt | Marko Mutanen | | Finland | | | | Enontekioe | 68,99700165 | 20,74399948 | |
| MM06092 | 658 | HM873032 | Tortricidae | Tortricinae | Phtheochroa vulneratan | Zetterstedt | Markus Rantala, Jari Kaitila | 01-Jul-2005 | Finland | | Regio kuusamoensis | | Kuusamo | 66,32099915 | 28,78499985 | |
| MM10310 | 658 | HM874648 | Tortricidae | Tortricinae | Phtheochroa vulneratan | Zetterstedt | Tomi Mutanen | 02-Jul-2008 | Finland | | Regio kuusamoensis | | Kuusamo | 66,31199646 | 29,45299911 | |
| TLMF Lep 02748 | 658 | JF860267 | Tortricidae | Tortricinae | Phtheochroa vulneratan | Zetterstedt | Schmid J. | 10-Jul-2008 | Switzerland | Graubunden | | | Val Zuort/ Tarasp-Fontan | 46,77199936 | 10,25800037 | |
| 04HBL003075 | 633 | JX008430 | Lycaenidae | Polyommatinae | Plebejus optilete | Knoch | P.D.N. Hebert | 04-Aug-2004 | Canada | Manitoba | Churchill | 22 km E Churchill, CNSC | Churchill Northern Studies Centre | 58,72999954 | -93,81999969 | |
| 07PROBE-00369 | 626 | JX008208 | Lycaenidae | Polyommatinae | Plebejus optilete | Knoch | S.VanRyswyk | 13-Jul-2007 | Canada | Manitoba | Churchill | 4 km SE Churchill, Dene Village | Railroad tracks nr | 58,73300171 | -94,12000275 | |
| MM03344 | 658 | HM871929 | Lycaenidae | Polyommatinae | Plebejus optilete | Knoch | Marko Mutanen, Nestori Mutanen, Anttoni Mutanen | 14-Jul-2006 | Finland | Oulun laani | Ostrobottnia borealis pars australis | | Kiiminki | 65,07099915 | 25,72500038 | |
| MM03345 | 658 | HM871930 | Lycaenidae | Polyommatinae | Plebejus optilete | Knoch | Marko Mutanen, Nestori Mutanen, Anttoni Mutanen | 14-Jul-2006 | Finland | Oulun laani | Ostrobottnia borealis pars australis | | Kiiminki | 65,07099915 | 25,72500038 | |
| TLMF Lep 01826 | 658 | HQ968289 | Lycaenidae | Polyommatinae | Plebejus optilete | Knoch | Mayr T. | 01-Aug-2009 | Switzerland | Graubunden | | | Val Bever, Alp Suvretta | 46,54999924 | 9,783329964 | |
| 09PROBE-09324 | 658 | HM375580 | Gelechiidae | Gelechiinae | Prolita sexpunctella | Fabricius | P.D.N. Hebert | 26-Jul-2009 | Canada | Manitoba | Churchill | 26 km SE Churchill, Twin Lakes burn site | | 58,61999893 | -93,83000183 | |
| 09PROBE-09328 | 658 | HM375584 | Gelechiidae | Gelechiinae | Prolita sexpunctella | Fabricius | P.D.N. Hebert | 26-Jul-2009 | Canada | Manitoba | Churchill | 26 km SE Churchill, Twin Lakes burn site | | 58,61999893 | -93,83000183 | |
| 09PROBE-09335 | 658 | HM375591 | Gelechiidae | Gelechiinae | Prolita sexpunctella | Fabricius | P.D.N. Hebert | 26-Jul-2009 | Canada | Manitoba | Churchill | 26 km SE Churchill, Twin Lakes burn site | | 58,61999893 | -93,83000183 | |

| | | | | | | | | | | | | | | | | |
|---|---|---|---|---|---|---|---|---|---|---|---|---|---|---|---|---|
| 09PROBE-09339 | 658 | HM375595 | Gelechiidae | Gelechiinae | Prolita sexpunctella | Fabricius | P.D.N. Hebert | 26-Jul-2009 | Canada | Manitoba | Churchill | 26 km SE Churchill, Twin Lakes burn site | | 58,61999893 | -93,83000183 | |
| 09PROBE-09360 | 658 | HM375616 | Gelechiidae | Gelechiinae | Prolita sexpunctella | Fabricius | P.D.N. Hebert | 26-Jul-2009 | Canada | Manitoba | Churchill | 26 km SE Churchill, Twin Lakes burn site | | 58,61999893 | -93,83000183 | |
| 09PROBE-0943( | 658 | HM37568! | Gelechiidae | Gelechiinae | Prolita sexpunctell. | Fabricius | P.D.N. Hebert | 30-Jul-2009 | Canada | Manitoba | Churchill | 11 km S Churchill, Goose Cree | Goose Creek Rd. at Rail Lin | 58,6599998! | -94,16999817 | |
| 09PROBE-0943: | 658 | HM37568! | Gelechiidae | Gelechiinae | Prolita sexpunctell. | Fabricius | P.D.N. Hebert | 30-Jul-2009 | Canada | Manitoba | Churchill | 11 km S Churchill, Goose Cree | Goose Creek Rd. at Rail Lin | 58,6599998! | -94,16999817 | |
| 09PROBE-09549 | 658 | HM375800 | Gelechiidae | Gelechiinae | Prolita sexpunctella | Fabricius | K.Mancuso | 21-Jul-2009 | Canada | Manitoba | Churchill | 9 km S Churchill, Goose Creek Cabin Bog | | 58,69200134 | -94,13200378 | |
| CNCLEP0002658 | 631 | JX008363 | Gelechiidae | Gelechiinae | Prolita sexpunctell. | Fabricius | JF Landry & G Poh | | Canada | Yukon Territory | | Whitehorse | Grey Mtn summi | 60,6749992 | -134,904007 | |
| CNCLEP0002658 | 634 | JX008393 | Gelechiidae | Gelechiinae | Prolita sexpunctell. | Fabricius | JF Landry & G Poh | | Canada | Yukon Territory | | Whitehorse | Grey Mtn summi | 60,6749992 | -134,904007 | |
| CNCLEP0002659 | 634 | JX008137 | Gelechiidae | Gelechiinae | Prolita sexpunctell. | Fabricius | JF Landry & G Poh | | Canada | Yukon Territory | | Whitehorse | Grey Mtn summi | 60,6749992 | -134,904007 | |
| CNCLEP0002659 | 634 | JX008301 | Gelechiidae | Gelechiinae | Prolita sexpunctell. | Fabricius | JF Landry & G Poh | | Canada | Yukon Territory | | Whitehorse | Grey Mtn summi | 60,6749992 | -134,904007 | |
| CNCLEP0002659 | 597 | JX008422 | Gelechiidae | Gelechiinae | Prolita sexpunctell. | Fabricius | JF Landry & G Poh | | Canada | Yukon Territory | | Whitehorse | Grey Mtn summi | 60,6749992 | -134,904007 | |
| CNCLEP0002873 | 631 | JX008480 | Gelechiidae | Gelechiinae | Prolita sexpunctell. | Fabricius | D Holder | 21-May-2006 | Canada | British Columbia | | Sheridan Lake | Cabin | | | |
| JD0184 | 658 | JX008455 | Gelechiidae | Gelechiinae | Prolita sexpunctell. | Fabricius | J. J. Dombroskie, C. Schmidt | 18-May-2006 | Canada | Alberta | | Jasper N. P., dunes | | 53,0970001 | -118,0029984 | MIC5911 M |
| JD0267 | 658 | JX008303 | Gelechiidae | Gelechiinae | Prolita sexpunctell. | Fabricius | J. J. Dombroskie, C. Schmidt | 18-May-2006 | Canada | Alberta | | Jasper N. P., dunes | | 53,0970001 | -118,0029984 | |
| KENWR 4389 | 658 | JX008221 | Gelechiidae | Gelechiinae | Prolita sexpunctella | Fabricius | D.Collet | | United States | Alaska | Kenai National Wildlife Refuge | | | | | |
| MM03220 | 658 | HM871884 | Gelechiidae | Gelechiinae | Prolita sexpunctella | Fabricius | Marko Mutanen | 29-Jun-2006 | Finland | Oulun laani | Ostrobottnia borealis pars australis | | Kiiminki | 65,07099915 | 25,72500038 | |
| MM06425 | 658 | HM873248 | Gelechiidae | Gelechiinae | Prolita sexpunctella | Fabricius | Marko Mutanen, Anttoni Mutanen, Nestori Mutanen | 08-Jun-2007 | Finland | | Regio kuusamoensis | | Kuusamo | 66,07499695 | 29,09900093 | |
| MM18233 | 658 | JF854228 | Gelechiidae | Gelechiinae | Prolita sexpunctella | Fabricius | Marko Mutanen | 10-Jun-2005 | Finland | | Ostrobottnia borealis pars australis | | Kiiminki | 65,07060242 | 25,72480011 | |
| NoA-08-219 | 658 | JX008475 | Gelechiidae | Gelechiinae | Prolita sexpunctell. | Fabricius | G.R. Poh | 14-Jun-2000 | Canada | Alberta | | | Ft Chipewyan Rc | 57,962001 | -105,068000 | |
| NoA-08-220 | 658 | JX008392 | Gelechiidae | Gelechiinae | Prolita sexpunctell. | Fabricius | G.R. Poh | 15-Jun-2000 | Canada | Alberta | | | Marguerite Crag & Tail Prov P | 57,7109985 | -110,3339996 | |
| NoA-08-221 | 658 | JX008259 | Gelechiidae | Gelechiinae | Prolita sexpunctell. | Fabricius | Pohl, G. R. | 15-Jun-2003 | Canada | Alberta | | | Caribou Mtns, Margaret I | 58,94900131 | -115,2649994 | |
| NoA-08-223 | 658 | JX008443 | Gelechiidae | Gelechiinae | Prolita sexpunctell. | Fabricius | | 16-Jun-2004 | Canada | Alberta | | | SE Hinton | 53,1850013 | -117,3529968 | |
| POHL-10-0008 | 658 | HM887742 | Gelechiidae | Gelechiinae | Prolita sexpunctell. | Fabricius | Pohl, G. R. | 15-Jul-2006 | Canada | Yukon Territory | | | Montana Mtn., vic Carcross | 60,0779991 | -134,7070007 | |
| TLMF Lep 0060 | 658 | HM381448 | Gelechiidae | Gelechiinae | Prolita sexpunctell. | Fabricius | Huemer P | 20-Jul-2009 | Italy | | Belluno | | Passo di Valparola E | 46,5255012 | 11,9997997 | |
| TLMF Lep 00757 | 658 | HM426111 | Gelechiidae | Gelechiinae | Prolita sexpunctella | Fabricius | Huemer P. | 29-Jul-2008 | Austria | Vorarlberg | | | Umg. Goeppinger Huette, Gamsboden | 47,21749878 | 10,03470039 | |
| TLMF Lep 00766 | 658 | HM426120 | Gelechiidae | Gelechiinae | Prolita sexpunctella | Fabricius | Huemer P. | 15-Jul-2009 | Austria | Steiermark | | | Tauplitzalm, Umg. Naturfreundehaus | 47,59579849 | 13,99499989 | |
| TLMF Lep 0179 | 658 | HQ968995 | Gelechiidae | Gelechiinae | Prolita sexpunctell. | Fabricius | Erlebach S. | 13-Jun-2009 | Austria | Tyrol | Nordtirol | | Sinnesbrunn, Kapelle E/ Tarren | 47,2983017 | 10,7982997 | |
| TLMF Lep 0179 | 658 | HQ968996 | Gelechiidae | Gelechiinae | Prolita sexpunctell. | Fabricius | Erlebach S. | 29-Jul-2004 | Austria | Tyrol | Nordtirol | Nordkette | Langer Sattel/ Innsbruck NW | 47,3106994 | 11,3587999 | |
| TLMF Lep 0179 | 658 | HQ968997 | Gelechiidae | Gelechiinae | Prolita sexpunctell. | Fabricius | Erlebach S. | 29-Jul-2004 | Austria | Tyrol | Nordtirol | Nordkette | Langer Sattel/ Innsbruck NW | 47,3106994 | 11,3587999 | |
| TLMF Lep 0179 | 658 | HQ968998 | Gelechiidae | Gelechiinae | Prolita sexpunctell. | Fabricius | Huemer P | 20-Jul-2009 | Italy | | Belluno | | Passo di Valparola E | 46,5255012 | 11,9997997 | |
| TLMF Lep 0275 | 606 | JF860270 | Gelechiidae | Gelechiinae | Prolita sexpunctell. | Fabricius | Schmid J. | 13-Jul-2009 | Switzerland | Graubunden | Val Poschiavo | | Curtinatsch/ Piz Alv S-Flanke | 46,4389991 | 9,9960002 | |
| TLMF Lep 05347 | 658 | JX034583 | Gelechiidae | Gelechiinae | Prolita sexpunctella | Fabricius | Huemer P. & Tarmann G. M. | 28-Jul-2011 | Macedonia | | | | Mavrovo NP, Korab, summit ridge | 41,78900146 | 20,54700089 | |
| TLMF Lep 05348 | 647 | JX034606 | Gelechiidae | Gelechiinae | Prolita sexpunctella | Fabricius | Huemer P. & Tarmann G. M. | 28-Jul-2011 | Macedonia | | | | Mavrovo NP, Korab, summit ridge | 41,78900146 | 20,54700089 | |
| MM1333 | 569 | HM87559 | Amphisbatidae | | Pseudatemelia elsa | Svensson | Marko Mutanen, Panu Vaelimael | | Finland | | Regio Aboensis | | Dragsfjaerd | 60,0110015 | 22,4979991 | |
| MM1560 | 658 | HM876401 | Amphisbatidae | | Pseudatemelia elsa | Svensson | Marko Mutanen | 09-Jul-2004 | Finland | | Regio Aboensis | | Karjalohja | 60,1920013 | 23,7549991 | |
| MM1561 | 658 | HM876402 | Amphisbatidae | | Pseudatemelia elsa | Svensson | Marko Mutanen | 10-Jul-2004 | Finland | | Regio Aboensis | | Karjalohja | 60,1920013 | 23,7549991 | |
| TLMF Lep 0278 | 454 | JX034598 | Amphisbatidae | | Pseudatemelia elsa | Svensson | Deutsch H. | 26-May-2000 | Italy | | Gorizia | | Monfalcone | 45,8120002 | 13,5240001 | |
| BC ZSM Lep 21996 | 658 | GU686972 | Hesperiidae | Pyrginae | Pyrgus andromedae | Wallengren | U. Buchsbaum | 17-Jul-1999 | Germany | Bavaria | Oberbayern | | Wettersteingebirge, Kreuzeck-Stuibensee | 47,45280075 | 11,0685997 | |
| MM0410 | 630 | HM38689 | Hesperiidae | Pyrginae | Pyrgus andromedae | Wallengren | Marko Mutanen | | Finland | | | | Enontekioe | 68,9970016 | 20,7439994 | |
| MM1057 | 631 | HQ57038 | Hesperiidae | Pyrginae | Pyrgus andromedae | Wallengren | Tomi Mutanen | 02-Jul-2003 | Finland | | Lapponia enontekiensis | | Enontekioe | 68,9970016 | 20,7439994 | |
| MM1710 | 658 | JN277845 | Hesperiidae | Pyrginae | Pyrgus andromedae | Wallengren | Tomi Mutanen | 25-Jun-2003 | Finland | | Lapponia enontekiensis | | Enontekioe | 69,0456008 | 20,8554000 | |
| MM1710 | 658 | JN277846 | Hesperiidae | Pyrginae | Pyrgus andromedae | Wallengren | Tomi Mutanen | 26-Jun-2003 | Finland | | Lapponia enontekiensis | | Enontekioe | 69,0456008 | 20,8554000 | |
| TLMF Lep 01108 | 658 | HQ968473 | Hesperiidae | Pyrginae | Pyrgus andromedae | Wallengren | Aistleitner U. | | Switzerland | Graubunden | | | Tiefencastel S/ Salouf, Got Grond | 46,65000153 | 9,597000122 | |
| TLMF Lep 0274 | 658 | JF860261 | Hesperiidae | Pyrginae | Pyrgus andromedae | Wallengren | Schmid J. | 11-Jun-2005 | Switzerland | Graubunden | | | Riefawald/ Vals | 46,6180000 | 9,194000244 | |
| 07PROBE-0037 | 658 | JX008344 | Geometridae | Larentiinae | Rheumaptera hastat | Linnaeus | S.VanRyswyk | 13-Jul-2007 | Canada | Manitoba | Churchill | 23 km E Churchill, Ramsay Cree | | 58,7229995 | -93,80699921 | |
| 08BBLEP-00165 | 658 | JX008391 | Geometridae | Larentiinae | Rheumaptera hastata | Linnaeus | J.Cossey,C.Carr,L.Wallace,J.Straka ,S.McCubbin,J.Sones,N.Jeffery | 29-Jun-2008 | Canada | Ontario | Pukaskwa | Pukaskwa National Park | Halfway Lake forest trail | 48,59400177 | -86,29000092 | |
| 08BBLEP-00167 | 658 | JX008492 | Geometridae | Larentiinae | Rheumaptera hastata | Linnaeus | J.Cossey,C.Carr,L.Wallace,J.Straka ,S.McCubbin,J.Sones,N.Jeffery | 29-Jun-2008 | Canada | Ontario | Pukaskwa | Pukaskwa National Park | Halfway Lake forest trail | 48,59400177 | -86,29000092 | |
| 08BBLEP-0018 | 658 | JX008351 | Geometridae | Larentiinae | Rheumaptera hastat | Linnaeus | J.Cossey | 29-Jun-2008 | Canada | Ontario | Pukaskwa | Pukaskwa National Par | camp site evergreen fores | 48,5900001 | -86,29000092 | |
| 08BBLEP-00521 | 658 | JX008291 | Geometridae | Larentiinae | Rheumaptera hastata | Linnaeus | J.Sones,J.Straka,J.Cossey,S.McCu bbin,L.Wallace | 29-Jun-2008 | Canada | Ontario | Pukaskwa | Pukaskwa National Park | Halfway Lake forest trail | 48,59000015 | -86,29000092 | |
| 08BBLEP-00594 | 658 | JX008296 | Geometridae | Larentiinae | Rheumaptera hastata | Linnaeus | J.Cossey,C.Carr,L.Wallace,J.Straka ,S.McCubbin,J.Sones,N.Jeffery | 02-Jul-2008 | Canada | Ontario | Pukaskwa | Pukaskwa National Park | Coastal Trail Boreal forest | 48,56000137 | -86,23000336 | |
| 08BBLEP-03441 | 658 | HM423866 | Geometridae | Larentiinae | Rheumaptera hastata | Linnaeus | J.Straka,J.Cossey | 26-Jul-2008 | Canada | Alberta | Waterton | Waterton Lakes Nat. Park - Blakiston Creek | Fast-flowing, rocky creekside | 49,07799911 | -113,8820038 | |
| 08BBLEP-03812 | 658 | JX008326 | Geometridae | Larentiinae | Rheumaptera hastata | Linnaeus | J.Straka,J.Cossey | 25-Jul-2008 | Canada | Alberta | Waterton | Waterton Lakes Nat. Park - Summit Lake Trai | Subalpine forest/meadow | 49,00799942 | -114,0250015 | |
| 08BBLEP-03813 | 658 | JX008358 | Geometridae | Larentiinae | Rheumaptera hastata | Linnaeus | J.Straka,J.Cossey | 25-Jul-2008 | Canada | Alberta | Waterton | Waterton Lakes Nat. Park - Summit Lake Trai | Subalpine forest/meadow | 49,00799942 | -114,0250015 | |
| 08BBLEP-03815 | 658 | JX008219 | Geometridae | Larentiinae | Rheumaptera hastata | Linnaeus | J.Straka,J.Cossey | 25-Jul-2008 | Canada | Alberta | Waterton | Waterton Lakes Nat. Park - Summit Lake Trai | Subalpine forest/meadow | 49,00799942 | -114,0250015 | |
| 09BBELE-1281 | 658 | HM415095 | Geometridae | Larentiinae | Rheumaptera hastata | Linnaeus | J. Cossey, R. Labbee, A. Smith, J. Smith, M. Zhang | 10-Jul-2009 | Canada | Newfoundland and Labrador | Terra Nova | Ochre`s Hill | Lookout | 48,50899887 | -53,95339966 | |
| 09BBELE-2209 | 658 | HM415808 | Geometridae | Larentiinae | Rheumaptera hastata | Linnaeus | J. Cossey, R. Labbee, A. Smith, J. Smith, M. Zhang | 08-Jul-2009 | Canada | Newfoundland and Labrador | Terra Nova | Sandy Pond | Hiking Trail | 48,49209976 | -54,02230072 | |
| 09BBELE-2210 | 658 | HM415809 | Geometridae | Larentiinae | Rheumaptera hastata | Linnaeus | J. Cossey, R. Labbee, A. Smith, J. Smith, M. Zhang | 08-Jul-2009 | Canada | Newfoundland and Labrador | Terra Nova | Sandy Pond | Hiking Trail | 48,49209976 | -54,02230072 | |
| 09BBELE-2436 | 658 | HM416031 | Geometridae | Larentiinae | Rheumaptera hastata | Linnaeus | J. Cossey, R. Labbee, A. Smith, J. Smith, M. Zhang | 05-Jul-2009 | Canada | Newfoundland and Labrador | Terra Nova | Highway 1 | Burn Site | 48,53519821 | -53,99140167 | |
| 09BBELE-2437 | 658 | HM416032 | Geometridae | Larentiinae | Rheumaptera hastata | Linnaeus | J. Cossey, R. Labbee, A. Smith, J. Smith, M. Zhang | 05-Jul-2009 | Canada | Newfoundland and Labrador | Terra Nova | Highway 1 | Burn Site | 48,53519821 | -53,99140167 | |
| 09BBELE-2442 | 658 | HM416037 | Geometridae | Larentiinae | Rheumaptera hastata | Linnaeus | J. Cossey, R. Labbee, A. Smith, J. Smith, M. Zhang | 04-Jul-2009 | Canada | Newfoundland and Labrador | Terra Nova | Malady Head | Green Head Meadow | 48,64870071 | -53,91699982 | |
| 09BBELE-2452 | 658 | HM416047 | Geometridae | Larentiinae | Rheumaptera hastata | Linnaeus | J. Cossey, R. Labbee, A. Smith, J. Smith, M. Zhang | 04-Jul-2009 | Canada | Newfoundland and Labrador | Terra Nova | Malady Head | Green Head Meadow | 48,64870071 | -53,91699982 | |

| | | | | | | | | | | | | | | | | |
|---|---|---|---|---|---|---|---|---|---|---|---|---|---|---|---|---|
| 09BBELE-2454 | 658 | HM416049 | Geometridae | Larentiinae | Rheumaptera hastata | Linnaeus | J. Cossey, R. Labbee, A. Smith, J. Smith, M. Zhang | 04-Jul-2009 | Canada | Newfoundland and Labrador | Terra Nova | Malady Head | Green Head Meadow | 48,64870071 | -53,91699982 | |
| 09PROBE-09636 | 639 | HM430280 | Geometridae | Larentiinae | Rheumaptera hastata | Linnaeus | P.D.N. Hebert | 26-Jul-2009 | Canada | Manitoba | Churchill | 26 km SE Churchill, Twin Lakes fen | | 58,63199997 | -93,78600311 | |
| 10BBCLP-0905 | 658 | JF841919 | Geometridae | Larentiinae | Rheumaptera hastat | Linnaeus | BIObus 2010 | 15-Jun-2010 | Canada | Saskatchewan | Prince Albert NP | Overflow Cmpgrd | Open field cmpgrd | 53,90499878 | -106,0739975 | |
| 10BBCLP-0911 | 658 | JF841925 | Geometridae | Larentiinae | Rheumaptera hastat | Linnaeus | BIObus 2010 | 11-Jun-2010 | Canada | Ontario | Pukaskwa NP | Pukaskwa NP Cmpgrd. Trls | Mossy boreal fores | 48,5929985 | -86,29389954 | |
| 10BBCLP-0912 | 658 | JF841926 | Geometridae | Larentiinae | Rheumaptera hastat | Linnaeus | BIObus 2010 | 12-Jun-2010 | Canada | Ontario | Pukaskwa NP | Pukaskwa NP Cmpgrd. Trls | Mossy boreal fores | 48,5929985 | -86,29389954 | |
| 10BBCLP-0913 | 658 | JF841927 | Geometridae | Larentiinae | Rheumaptera hastat | Linnaeus | BIObus 2010 | 10-Jun-2010 | Canada | Ontario | Pukaskwa NP | Pukaskwa NP Halfway Lak | Dried bank of lake | 48,59400177 | -86,29579926 | |
| 10BBCLP-0914 | 658 | JF841928 | Geometridae | Larentiinae | Rheumaptera hastat | Linnaeus | BIObus 2010 | 11-Jun-2010 | Canada | Ontario | Pukaskwa NP | Pukaskwa NP Cmpgrd. Trls | Mossy boreal fores | 48,5929985 | -86,29389954 | |
| 10BBCLP-0915 | 658 | JF841929 | Geometridae | Larentiinae | Rheumaptera hastat | Linnaeus | BIObus 2010 | 28-Jun-2010 | Canada | Alberta | Jasper NP | Pyramid Lake | Douglas fir forest | 52,91600037 | -118,1039963 | |
| 10BBCLP-0920 | 658 | JF841934 | Geometridae | Larentiinae | Rheumaptera hastat | Linnaeus | BIObus 2010 | 23-Jul-2010 | Canada | British Columbia | Yoho NP | Takakkaw Fall | Forested trail in river basin | 51,5 | -116,4729996 | |
| 10BBCLP-0921 | 658 | JF841935 | Geometridae | Larentiinae | Rheumaptera hastat | Linnaeus | BIObus 2010 | 23-Jul-2010 | Canada | British Columbia | Yoho NP | Takakkaw Fall | Forested trail in river basin | 51,5 | -116,4729996 | |
| 10BBCLP-0922 | 658 | JF841936 | Geometridae | Larentiinae | Rheumaptera hastat | Linnaeus | BIObus 2010 | 21-Jul-2010 | Canada | British Columbia | Yoho NP | Emerald Lake Trls | Lake perimeter | 51,44300079 | -116,5419998 | |
| 10BBCLP-0923 | 658 | JF841937 | Geometridae | Larentiinae | Rheumaptera hastat | Linnaeus | BIObus 2010 | 23-Jul-2010 | Canada | British Columbia | Yoho NP | Takakkaw Fall | Forested trail in river basin | 51,5 | -116,4729996 | |
| 10BBCLP-0924 | 658 | JF841938 | Geometridae | Larentiinae | Rheumaptera hastat | Linnaeus | BIObus 2010 | 28-Jul-2010 | Canada | British Columbia | Kootenay NP | Cobb Lake Trl | Mixed fores | 50,67200089 | -115,8949966 | |
| 10BBCLP-0925 | 658 | JF841939 | Geometridae | Larentiinae | Rheumaptera hastat | Linnaeus | BIObus 2010 | 28-Jul-2010 | Canada | British Columbia | Kootenay NP | Cobb Lake Trl | Mixed fores | 50,67200089 | -115,8949966 | |
| 10BBCLP-0926 | 658 | JF841940 | Geometridae | Larentiinae | Rheumaptera hastat | Linnaeus | BIObus 2010 | 28-Jul-2010 | Canada | British Columbia | Kootenay NP | Cobb Lake Trl | Mixed fores | 50,67200089 | -115,8949966 | |
| 10BBCLP-0927 | 658 | JF841941 | Geometridae | Larentiinae | Rheumaptera hastat | Linnaeus | BIObus 2010 | 28-Jul-2010 | Canada | British Columbia | Kootenay NP | Cobb Lake Trl | Mixed fores | 50,67200089 | -115,8949966 | |
| 10BBCLP-0929 | 658 | JF841943 | Geometridae | Larentiinae | Rheumaptera hastat | Linnaeus | BIObus 2010 | 15-Jul-2010 | Canada | British Columbia | Mt. Revelstoke NP | Summit Trls | Subalpine meadow | 51,03900146 | -118,1480026 | |
| 10BBCLP-0930 | 658 | JF841944 | Geometridae | Larentiinae | Rheumaptera hastat | Linnaeus | BIObus 2010 | 17-Jul-2010 | Canada | British Columbia | Mt. Revelstoke NP | Summit Trls | Subalpine meadow | 51,03900146 | -118,1480026 | |
| 10BBCLP-0931 | 658 | JF841945 | Geometridae | Larentiinae | Rheumaptera hastat | Linnaeus | BIObus 2010 | 17-Jul-2010 | Canada | British Columbia | Mt. Revelstoke NP | Summit Trls | Subalpine meadow | 51,03900146 | -118,1480026 | |
| 10BBCLP-0932 | 658 | JF841946 | Geometridae | Larentiinae | Rheumaptera hastat | Linnaeus | BIObus 2010 | 15-Jul-2010 | Canada | British Columbia | Mt. Revelstoke NP | Summit Trls | Subalpine meadow | 51,03900146 | -118,1480026 | |
| 10BBCLP-0933 | 658 | JF841947 | Geometridae | Larentiinae | Rheumaptera hastat | Linnaeus | BIObus 2010 | 16-Jul-2010 | Canada | British Columbia | Glacier NP | Loop Trl. | Riverside fores | 51,25400162 | -117,538002 | |
| 10BBCLP-0934 | 658 | JF841948 | Geometridae | Larentiinae | Rheumaptera hastat | Linnaeus | BIObus 2010 | 14-Jul-2010 | Canada | British Columbia | Glacier NP | Loop Trl. | Riverside fores | 51,25600052 | -117,538002 | |
| 10BBCLP-0935 | 658 | JF841949 | Geometridae | Larentiinae | Rheumaptera hastat | Linnaeus | BIObus 2010 | 16-Jul-2010 | Canada | British Columbia | Glacier NP | Illecillewaet Area Trls | Mountain valley | 51,26499939 | -117,4940033 | |
| 10BBCLP-0941 | 658 | JF841955 | Geometridae | Larentiinae | Rheumaptera hastat | Linnaeus | BIObus 2010 | 14-Jul-2010 | Canada | British Columbia | Glacier NP | Loop Trl. | Riverside fores | 51,25600052 | -117,538002 | |
| 10BBCLP-0942 | 658 | JF841956 | Geometridae | Larentiinae | Rheumaptera hastat | Linnaeus | BIObus 2010 | 16-Jul-2010 | Canada | British Columbia | Glacier NP | Illecillewaet Area Trls | Mountain valley | 51,26499939 | -117,4940033 | |
| 10BBCLP-0943 | 658 | JF841957 | Geometridae | Larentiinae | Rheumaptera hastat | Linnaeus | BIObus 2010 | 16-Jul-2010 | Canada | British Columbia | Glacier NP | Illecillewaet Area Trls | Mountain valley | 51,26499939 | -117,4940033 | |
| 10BBCLP-0944 | 658 | JF841958 | Geometridae | Larentiinae | Rheumaptera hastat | Linnaeus | BIObus 2010 | 14-Jul-2010 | Canada | British Columbia | Glacier NP | Bostock Trl | Montane fores | 51,22999954 | -117,6689987 | |
| 10BBCLP-0945 | 658 | JF841959 | Geometridae | Larentiinae | Rheumaptera hastat | Linnaeus | BIObus 2010 | 16-Jul-2010 | Canada | British Columbia | Glacier NP | Illecillewaet Area Trls | Mountain valley | 51,26499939 | -117,4940033 | |
| BC ZSM Lep 35517 | 232 | JX034628 | Geometridae | Larentiinae | Rheumaptera hastat | Linnaeus | P. Skou | 02-Jul-2000 | Norway | Vestfold | | | Prestseter | 60,68349838 | 10,64210033 | |
| KENWR LEP-05 | 630 | JX008389 | Geometridae | Larentiinae | Rheumaptera hastata | Linnaeus | D.Collet | 19-Jun-2004 | United States | Alaska | Kenai National Wildlife Refuge | Finger Lake Road | open mixed forest | 60,65000153 | -150,8329926 | |
| KENWR LEP-06 | 630 | JX008487 | Geometridae | Larentiinae | Rheumaptera hastata | Linnaeus | D.Collet | 19-Jun-2004 | United States | Alaska | Kenai National Wildlife Refuge | Finger Lake Road | open mixed forest | 60,65000153 | -150,8329926 | |
| MM00580 | 658 | HM871078 | Geometridae | Larentiinae | Rheumaptera hastata | Linnaeus | Marko Mutanen | 07-Jun-2006 | Finland | | Ostrobottnia borealis pars australis | | Kiiminki | 65,07099915 | 25,72500038 | |
| MM06321 | 609 | HM873199 | Geometridae | Larentiinae | Rheumaptera hastata | Linnaeus | Marko Mutanen, Anttoni Mutanen, Nestori Mutanen | 27-Jun-2006 | Finland | | Ostrobottnia borealis pars australis | | Tornio | 65,86100006 | 24,37100029 | |
| MM10108 | 658 | HM874579 | Geometridae | Larentiinae | Rheumaptera hastata | Linnaeus | Marko Mutanen, Nestori Mutanen, Anttoni Mutanen | 21-Jun-2008 | Finland | | Ostrobottnia borealis pars australis | | Kiiminki | 65,07099915 | 25,72500038 | |
| MM18450 | 658 | JF854331 | Geometridae | Larentiinae | Rheumaptera hastata | Linnaeus | Marko Mutanen, Nestori Mutanen, Anttoni Mutanen | 06-Jun-2010 | Finland | | Ostrobottnia borealis pars australis | | Kiiminki | 65,07060242 | 25,72480011 | |
| MM18451 | 658 | JF854332 | Geometridae | Larentiinae | Rheumaptera hastata | Linnaeus | Marko Mutanen, Nestori Mutanen, Anttoni Mutanen | 11-Jun-2010 | Finland | | Ostrobottnia borealis pars australis | | Kiiminki | 65,07060242 | 25,72480011 | |
| MM18452 | 658 | JF854333 | Geometridae | Larentiinae | Rheumaptera hastata | Linnaeus | Marko Mutanen | 21-Jun-1999 | Finland | | Ostrobottnia borealis pars australis | | Utajaervi | 65,042099 | 23,81450081 | |
| MNBTT-208 | 658 | JX008361 | Geometridae | Larentiinae | Rheumaptera hastat | Linnaeus | A. W. Thomas | 29-Jun-2005 | Canada | New Brunswick | York County | | Fredericton north, 45 | 45,84999847 | -67,69999695 | |
| MNBTT-2840 | 563 | JX008230 | Geometridae | Larentiinae | Rheumaptera hastat | Linnaeus | A. W. Thomas | 12-Jun-2006 | Canada | New Brunswick | Sunbury Co. | Acadia Research Fores | | 46 | -66,30000305 | |
| TLMF Lep 02812 | 658 | JF860306 | Geometridae | Larentiinae | Rheumaptera hastat | Linnaeus | Erlebach S. | 24-Mar-2010 | Austria | Tyrol | Nordtiro | | Riedaste/ Aschau | 47,26800156 | 11,86699963 | genitalia in glycerin |
| TLMF Lep 02813 | 658 | JF860307 | Geometridae | Larentiinae | Rheumaptera hastat | Linnaeus | Erlebach S. & Tarmann G. M | 16-Jul-2009 | Austria | Tyrol | Nordtiro | | Roetboden/ Lanersbach | 47,12799835 | 11,72599983 | |
| TLMF Lep 02815 | 428 | JX034635 | Geometridae | Larentiinae | Rheumaptera hastat | Linnaeus | Erlebach S. & Tarmann G. M | 16-Jul-2009 | Austria | Tyrol | Nordtiro | | Roetboden/ Lanersbach | 47,12799835 | 11,72599983 | genitalia in glycerin |
| TLMF Lep 02816 | 658 | JF860308 | Geometridae | Larentiinae | Rheumaptera hastat | Linnaeus | Erlebach S. | 25-Mar-2007 | Austria | Tyrol | Umg. Innsbruck | | Samertal, Brandstatt S | 47,33800125 | 11,3760004 | genitalia in glycerin |
| TLMF Lep 02818 | 658 | JF860309 | Geometridae | Larentiinae | Rheumaptera hastata | Linnaeus | Huemer P. & Erlebach S. | 11-Jul-2008 | Austria | Salzburg | | | Wildgerlostal, S Trisslalm/ Krimml | 47,18999863 | 12,11299992 | genitalia in glycerin |
| TLMF Lep 02824 | 658 | JF860310 | Geometridae | Larentiinae | Rheumaptera hastata | Linnaeus | Mayr T. | 01-Jul-2006 | Switzerland | Graubunden | | | Weg zwischen Gadastatt und Sulzalp, Splueger | 46,55799866 | 9,31099987 | |
| TLMF Lep 02825 | 658 | JF860311 | Geometridae | Larentiinae | Rheumaptera hastat | Linnaeus | Mayr T. | 19-Jun-2005 | Switzerland | Graubunden | | | Surselva, Sedrun, Stausee Nalp | 46,64199829 | 8,760000229 | |
| TLMF Lep 02829 | 658 | JF860312 | Geometridae | Larentiinae | Rheumaptera hastata | Linnaeus | Huemer P. & Erlebach S. | 11-Jul-2008 | Austria | Salzburg | | | Wildgerlostal, S Trisslalm/ Krimml | 47,18999863 | 12,11299992 | |
| 07PROBE-00363 | 655 | JX008307 | Geometridae | Larentiinae | Rheumaptera subhastat | Nolcken | E.Robinson | 13-Jul-2007 | Canada | Manitoba | Churchill | 23 km E Churchill, Ramsay Cree | | 58,72299957 | -93,80699921 | |
| 07PROBE-00602 | 656 | JX008407 | Geometridae | Larentiinae | Rheumaptera subhastat | Nolcken | P.D.N. Hebert | 15-Jul-2007 | Canada | Manitoba | Churchill | 26 km SE Churchill, Twin Lake | | 58,63199997 | -93,81900024 | |
| 07PROBE-03820 | 658 | JX008300 | Geometridae | Larentiinae | Rheumaptera subhastat | Nolcken | A.Renaud | 30-Jun-2007 | Canada | Manitoba | Churchill | 23 km E Churchill, Ramsay Cree | | 58,73099899 | -93,77999878 | |
| 08BBLEP-01669 | 644 | JX008379 | Geometridae | Larentiinae | Rheumaptera subhastat | Nolcken | J.Cossey, N.Jeffery, J.Straka | 14-Jul-2008 | Canada | Saskatchewan | Grasslands | Grasslands N.P. Visitor`s cente | grass cattle pasture | 49,24599838 | -107,7320023 | |
| 08BBLEP-03816 | 615 | JX008207 | Geometridae | Larentiinae | Rheumaptera subhastata | Nolcken | J.Straka,J.Cossey | 25-Jul-2008 | Canada | Alberta | Waterton | Waterton Lakes Nat. Park - Summit Lake Trai | Subalpine forest/meadow | 49,00799942 | -114,0250015 | |
| 09BBELE-2458 | 658 | HM416053 | Geometridae | Larentiinae | Rheumaptera subhastata | Nolcken | J. Cossey, R. Labbee, A. Smith, J. Smith, M. Zhang | 05-Jul-2009 | Canada | Newfoundland and Labrador | Terra Nova | Highway 1 | Burn Site | 48,53519821 | -53,99140167 | |
| 09PROBE-09522 | 658 | HM405868 | Geometridae | Larentiinae | Rheumaptera subhastata | Nolcken | P.D.N. Hebert | 19-Jul-2009 | Canada | Manitoba | Churchill | 26 km SE Churchill, Twin Lakes fen | | 58,63199997 | -93,78600311 | |
| 09PROBE-09525 | 658 | HM375776 | Geometridae | Larentiinae | Rheumaptera subhastata | Nolcken | P.D.N. Hebert | 19-Jul-2009 | Canada | Manitoba | Churchill | 26 km SE Churchill, Twin Lakes fen | | 58,63199997 | -93,78600311 | |
| 09PROBE-09562 | 658 | HM375813 | Geometridae | Larentiinae | Rheumaptera subhastata | Nolcken | P.D.N. Hebert | 24-Jul-2009 | Canada | Manitoba | Churchill | 26 km SE Churchill, Twin Lakes burn site | | 58,61999893 | -93,83000183 | |
| 09PROBE-09568 | 658 | HM375819 | Geometridae | Larentiinae | Rheumaptera subhastata | Nolcken | P.D.N. Hebert | 24-Jul-2009 | Canada | Manitoba | Churchill | 26 km SE Churchill, Twin Lakes burn site | | 58,61999893 | -93,83000183 | |
| 09PROBE-09571 | 658 | HM405869 | Geometridae | Larentiinae | Rheumaptera subhastata | Nolcken | P.D.N. Hebert | 24-Jul-2009 | Canada | Manitoba | Churchill | 26 km SE Churchill, Twin Lakes burn site | | 58,61999893 | -93,83000183 | |
| 09PROBE-09637 | 658 | HM430281 | Geometridae | Larentiinae | Rheumaptera subhastata | Nolcken | P.D.N. Hebert | 26-Jul-2009 | Canada | Manitoba | Churchill | 26 km SE Churchill, Twin Lakes fen | | 58,63199997 | -93,78600311 | |
| 09PROBE-09638 | 614 | HM430282 | Geometridae | Larentiinae | Rheumaptera subhastata | Nolcken | P.D.N. Hebert | 26-Jul-2009 | Canada | Manitoba | Churchill | 26 km SE Churchill, Twin Lakes fen | | 58,63199997 | -93,78600311 | |
| 10BBCLP-0906 | 658 | JF841920 | Geometridae | Larentiinae | Rheumaptera subhastat | Nolcken | BIObus 2010 | 18-Jun-2010 | Canada | Saskatchewan | Prince Albert NP | Overflow Cmpgrd | Open field cmpgrd | 53,90499878 | -106,0739975 | |
| BC NP 0376 | 658 | JX034645 | Geometridae | Larentiinae | Rheumaptera subhastat | Nolcken | N. Poel | 27-Jun-2004 | Austria | Oberoesterreich | | | Loeckenmoosberg | 47,56000137 | 13,55000019 | |
| BC ZSM Lep 39348 | 658 | JF851430 | Geometridae | Larentiinae | Rheumaptera subhastat | Nolcken | A. Haslberger | 17-Jul-1999 | Austria | Salzburg | | | Obertauern | 47,24829865 | 13,55770016 | |
| BC ZSM Lep 49214 | 658 | JN274121 | Geometridae | Larentiinae | Rheumaptera subhastat | Nolcken | L. Weigert | 07-Jul-2010 | Sweden | | Torne Lappmark | | 8 km NE Jukkasjaerv | 67,88890076 | 20,75639915 | |
| CHU06-COL-283 | 658 | JX008352 | Geometridae | Larentiinae | Rheumaptera subhastat | Nolcken | Arctic & Boreal Entomology | 14-Aug-2006 | Canada | Manitoba | Churchill | 23 km E Churchill, Ramsay Cree | Small Creek | 58,73099899 | -93,77999878 | |
| CHU06-COL-285 | 658 | JX008330 | Geometridae | Larentiinae | Rheumaptera subhastat | Nolcken | Arctic & Boreal Entomology | 14-Aug-2006 | Canada | Manitoba | Churchill | 23 km E Churchill, Ramsay Cree | Small Creek | 58,73099899 | -93,77999878 | |
| CHU06-COL-286 | 658 | JX008494 | Geometridae | Larentiinae | Rheumaptera subhastat | Nolcken | Arctic & Boreal Entomology | 14-Aug-2006 | Canada | Manitoba | Churchill | 23 km E Churchill, Ramsay Cree | Small Creek | 58,73099899 | -93,77999878 | |

| | | | | | | | | | | | | | | | |
|---|---|---|---|---|---|---|---|---|---|---|---|---|---|---|---|
| MM06292 | 658 | HM873178 | Geometridae | Larentiinae | Rheumaptera subhastata | Nolcken | Marko Mutanen, Nestori Mutanen, Anttoni Mutanen | 27-Jun-2007 | Finland | | Lapponia kemensis pars orientalis | Sodankylae | 68,22299957 | 27,21699905 | |
| MM06308 | 658 | HM873191 | Geometridae | Larentiinae | Rheumaptera subhastata | Nolcken | Marko Mutanen, Anttoni Mutanen, Nestori Mutanen | 28-Jun-2006 | Finland | | Lapponia inarensis | Inari | 68,4469986 | 27,41399956 | |
| MM10312 | 658 | HM874649 | Geometridae | Larentiinae | Rheumaptera subhastat | Nolcken | Tomi Mutanen | 02-Jul-2008 | Finland | | Regio kuusamoensis | Kuusamo | 66,31199646 | 29,45299911 | |
| MM17986 | 658 | JF854040 | Geometridae | Larentiinae | Rheumaptera subhastata | Nolcken | Marko Mutanen, Nestori Mutanen, Anttoni Mutanen | 10-Jul-2010 | Finland | | Lapponia inarensis | Utsjoki | 69,82800293 | 27 | |
| MM17987 | 658 | JF854041 | Geometridae | Larentiinae | Rheumaptera subhastata | Nolcken | Marko Mutanen, Nestori Mutanen, Anttoni Mutanen | 10-Jul-2010 | Finland | | Lapponia inarensis | Utsjoki | 69,82800293 | 27 | |
| MM18454 | 658 | JF854334 | Geometridae | Larentiinae | Rheumaptera subhastata | Nolcken | Marko Mutanen | 15-Jun-1999 | Finland | | Ostrobottnia borealis pars australis | Utajaervi | 65,042099 | 23,81450081 | |
| MM18455 | 658 | JF854335 | Geometridae | Larentiinae | Rheumaptera subhastata | Nolcken | Tomi Mutanen | 03-Jun-1999 | Finland | | Ostrobottnia borealis pars australis | Ii | 65,33529663 | 25,2826004 | |
| MM18456 | 658 | JN279414 | Geometridae | Larentiinae | Rheumaptera subhastat | Nolcken | Tomi Mutanen | 24-Jun-2003 | Finland | | Lapponia enontekiensis | Enontekioe | 69,04560089 | 20,85540009 | |
| MM18457 | 598 | JF854336 | Geometridae | Larentiinae | Rheumaptera subhastat | Nolcken | Marko Mutanen, Tomi Mutane | 01-Aug-2000 | Finland | | Lapponia inarensis | Inari | 68,39360046 | 27,2432003 | |
| MM18459 | 581 | JX034571 | Geometridae | Larentiinae | Rheumaptera subhastata | Nolcken | Marko Mutanen | 09-Jun-1999 | Finland | | Ostrobottnia borealis pars australis | Utajaervi | 65,042099 | 23,81450081 | |
| MM18460 | 658 | JF854337 | Geometridae | Larentiinae | Rheumaptera subhastata | Nolcken | Marko Mutanen | 09-Jun-1999 | Finland | | Ostrobottnia borealis pars australis | Utajaervi | 65,042099 | 23,81450081 | |
| MM18462 | 618 | JF854338 | Geometridae | Larentiinae | Rheumaptera subhastata | Nolcken | Marko Mutanen | 15-Jun-1999 | Finland | | Ostrobottnia borealis pars australis | Utajaervi | 65,042099 | 23,81450081 | |
| MM18463 | 658 | JF854339 | Geometridae | Larentiinae | Rheumaptera subhastata | Nolcken | Tomi Mutanen | 11-Jun-1999 | Finland | | Ostrobottnia borealis pars borealis | Simo | 65,69619751 | 25,47640038 | |
| MM18464 | 658 | JF854340 | Geometridae | Larentiinae | Rheumaptera subhastata | Nolcken | Tomi Mutanen | 11-Jun-1999 | Finland | | Ostrobottnia borealis pars borealis | Simo | 65,69619751 | 25,47640038 | |
| TLMF Lep 02831 | 658 | JF860313 | Geometridae | Larentiinae | Rheumaptera subhastat | Nolcken | Erlebach S. | 28-Mar-2006 | Austria | | Nordtirol, Zentralalpe | Zillergrund: Baerenbadalm | 47,12200165 | 12,04300022 | |
| MM15633 | 658 | HM876421 | Gelechiidae | Gelechiinae | Scrobipalpa murinella | Duponchel | Tomi Mutanen | 05-Jun-2004 | Finland | | Karelia borealis | Liperi | 62,63100052 | 29,5340004 | |
| MM15634 | 658 | HM876422 | Gelechiidae | Gelechiinae | Scrobipalpa murinella | Duponchel | Tomi Mutanen | 05-Jun-2004 | Finland | | Karelia borealis | Liperi | 62,63100052 | 29,5340004 | |
| MM17937 | 658 | JF853997 | Gelechiidae | Gelechiinae | Scrobipalpa murinella | Duponchel | Sami Haapala | 31-May-2009 | Finland | | Savonia australis | Imatra | 61,24129868 | 28,88100052 | |
| TLMF Lep 02847 | 658 | JX034595 | Gelechiidae | Gelechiinae | Scrobipalpa murinella | Duponchel | Huemer P. | 04-Aug-2010 | France | Provence-Alpes-Cote d'Azur | | Col Agnel | 44,68899918 | 6,984000206 | |
| TLMF Lep 02848 | 658 | JX034624 | Gelechiidae | Gelechiinae | Scrobipalpa murinella | Duponchel | Huemer P. | 04-Aug-2010 | France | Provence-Alpes-Cote d'Azur | | Col Agnel | 44,68899918 | 6,984000206 | |
| TLMF Lep 02849 | 658 | JX034587 | Gelechiidae | Gelechiinae | Scrobipalpa murinella | Duponchel | Huemer P. | 04-Aug-2010 | France | Provence-Alpes-Cote d'Azur | | Col Agnel | 44,68899918 | 6,984000206 | |
| MM10495 | 658 | HM874766 | Gelechiidae | Gelechiinae | Scrobipalpopsis petasitis | Pfaffenzeller | Marko Mutanen, Tomi Mutanen | | Finland | | Lapponia kemensis pars orientalis | Pelkosenniemi | 67,13700104 | 27,69199944 | |
| MM11172 | 658 | HM875036 | Gelechiidae | Gelechiinae | Scrobipalpopsis petasitis | Pfaffenzeller | Marko Mutanen, Panu Vaelimaeki | | Finland | | Lapponia kemensis pars orientalis | Pelkosenniemi | 67,15399933 | 27,85400009 | |
| MM18235 | 658 | JF854230 | Gelechiidae | Gelechiinae | Scrobipalpopsis petasitis | Pfaffenzeller | Marko Mutanen, Tomi Mutanen | 01-Aug-2000 | Finland | | Lapponia kemensis pars orientalis | Pelkosenniemi | 67,13710022 | 27,6916008 | |
| TLMF Lep 00865 | 658 | HQ968239 | Gelechiidae | Gelechiinae | Scrobipalpopsis petasitis | Pfaffenzeller | Huemer P. | 20-Jun-2008 | Austria | Vorarlberg | | Sonntag, Buchboden, NW Bad Rotenbrunnen | 47,24499893 | 9,970999718 | |
| TLMF Lep 00866 | 658 | HQ968240 | Gelechiidae | Gelechiinae | Scrobipalpopsis petasitis | Pfaffenzeller | Huemer P. | 20-Jun-2008 | Austria | Vorarlberg | | Sonntag, Buchboden, NW Bad Rotenbrunnen | 47,24499893 | 9,970999718 | |
| TLMF Lep 01930 | 658 | HQ968342 | Gelechiidae | Gelechiinae | Scrobipalpopsis petasitis | Pfaffenzeller | Huemer P | 08-Jun-2010 | Austria | Tyrol | Nordtirol, Hallta | Hallta | 47,32730103 | 11,52470016 | |
| TLMF Lep 01931 | 658 | HQ968343 | Gelechiidae | Gelechiinae | Scrobipalpopsis petasitis | Pfaffenzeller | Huemer P | 08-Jun-2010 | Austria | Tyrol | Nordtirol, Hallta | Hallta | 47,32730103 | 11,52470016 | |
| TLMF Lep 02753 | 658 | JF860271 | Gelechiidae | Gelechiinae | Scrobipalpopsis petasitis | Pfaffenzeller | Schmid J. | 26-May-2006 | Switzerland | Graubunder | | Versamer Tobel/ Bonadu | 46,79800034 | 9,345999718 | |
| TLMF Lep 02949 | 658 | JF860385 | Gelechiidae | Gelechiinae | Scrobipalpopsis petasitis | Pfaffenzeller | Huemer P. | 15-Jun-2010 | Austria | | Vorarlberg | Sonntag, Buchboden, E Hintergurga | 47,24900055 | 9,991000175 | |
| TLMF Lep 02950 | 658 | JF860386 | Gelechiidae | Gelechiinae | Scrobipalpopsis petasitis | Pfaffenzeller | Huemer P. | 15-Jun-2010 | Austria | | Vorarlberg | Sonntag, Buchboden, E Hintergurga | 47,24900055 | 9,991000175 | |
| MM15929 | 658 | JF853548 | Arctiidae | Arctiinae | Setema cereola | Hübner | Arto Tervonoen | 21-Jul-1995 | Finland | | Savonia australis | Mikkel | 61,75699997 | 27 | |
| MM17452 | 658 | JF853847 | Arctiidae | Arctiinae | Setema cereola | Hübner | Kari Vaalamo | 23-Jul-1993 | Finland | | Savonia australis | Mikkelin mlk | 61,75699997 | 27 | |
| TLMF Lep 00294 | 658 | HM381355 | Arctiidae | Arctiinae | Setema cereola | Hübner | Huemer P. | 16-Jul-2009 | Austria | Vorarlberg | | Marul, Lagutzbach, Beim alten Stafel | 47,20220184 | 9,928059578 | |
| TLMF Lep 02755 | 658 | JF860273 | Arctiidae | Arctiinae | Setema cereola | Hübner | Schmid J. | 23-Jun-2007 | Switzerland | Graubunder | | Stugl, Plazi/ Berguen | 46,65100098 | 9,729999542 | |
| TLMF Lep 02896 | 658 | JF860347 | Arctiidae | Arctiinae | Setema cereola | Hübner | Huemer P. | 16-Jul-2009 | Austria | | Vorarlberg | Marul, Lagutzbach, Beim alten Stafel | 47,20199966 | 9,92800045 | |
| TLMF Lep 02897 | 658 | JF860348 | Arctiidae | Arctiinae | Setema cereola | Hübner | Huemer P. | 16-Jul-2009 | Austria | | Vorarlberg | Marul, Lagutzbach, Beim alten Stafel | 47,20199966 | 9,92800045 | |
| MM04145 | 658 | HM386932 | Tortricidae | Tortricinae | Sparganothis praecana | Kennel | Marko Mutanen | | Finland | | | Enontekioe | 68,99700165 | 20,74399948 | |
| MM04146 | 658 | HM386933 | Tortricidae | Tortricinae | Sparganothis praecana | Kennel | Marko Mutanen | | Finland | | | Enontekioe | 68,99700165 | 20,74399948 | |
| MM14580 | 658 | HM876174 | Tortricidae | Tortricinae | Sparganothis praecana | Kennel | Marko Mutanen | | Finland | | Lapponia enontekiensis | Enontekioe | 69,06300354 | 21,10199928 | |
| TLMF Lep 02890 | 369 | JX034612 | Tortricidae | Tortricinae | Sparganothis praecana | Kennel | Jaros J. & Spitzer K. | 04-Jul-1997 | Austria | Steiermark | | Klosterneuburger Huett | 47,26499939 | 14,37800026 | |
| MM12509 | 658 | HM875304 | Noctuidae | | Standfussiana lucernea | Linnaeus | Marko Mutanen, Panu Vaelimaeki | | Finland | | Regio Aboensis | Dragsfjaerd | 60,01100159 | 22,49799919 | |
| MM17221 | 658 | JF853696 | Noctuidae | | Standfussiana lucernea | Linnaeus | Lauri Kaila | 30-Jun-2006 | Finland | | Nylandia | Tammisaari | 59,82899857 | 23,61199951 | |
| MM17499 | 658 | JX034667 | Noctuidae | | Standfussiana lucernea | Linnaeus | Henrik Bruun | | Finland | | Regio Aboensis | Houtskaer | 60,1969986 | 21,26199913 | |
| MM17500 | 658 | JX034653 | Noctuidae | | Standfussiana lucernea | Linnaeus | Henrik Bruun | | Finland | | Regio Aboensis | Houtskaer | 60,1969986 | 21,26199913 | |
| TLMF Lep 00351 | 658 | HM425843 | Noctuidae | | Standfussiana lucernea | Linnaeus | Huemer P. | 26-Jul-2009 | France | Provence-Alpes-Cote d'Azur | | N Col de la Boucharde/ Col de la Cayolle N | 44,28329849 | 6,743330002 | |
| TLMF Lep 00376 | 658 | HM425864 | Noctuidae | | Standfussiana lucernea | Linnaeus | Huemer P. | 31-Jul-2009 | Austria | Karnten | | Mallnitz, Doesental, Arthur-v.-Schmid-Haus W | 46,98939896 | 13,26690006 | |
| TLMF Lep 00415 | 658 | HM425898 | Noctuidae | | Standfussiana lucernea | Linnaeus | Huemer P. | 23-Jul-2009 | Italy | | Cuneo | Colle Valcavera NE/ Demonte NW | 44,38439941 | 7,106389999 | |
| TLMF Lep 01960 | 658 | HQ968372 | Noctuidae | | Standfussiana lucernea | Linnaeus | Huemer P. | 14-Jul-2010 | Italy | Abruzzi | L`Aquila | NP Gran Sasso, Campo Imperatore, ex Miniera di Lignite | 42,42670059 | 13,71329975 | |
| TLMF Lep 05543 | 658 | JX034615 | Noctuidae | | Standfussiana lucernea | Linnaeus | Huemer P. & Tarmann G. M. | 28-Jul-2011 | Macedonia | | | Mavrovo NP, Korab, Korabska jezero, Kobilino pole | 41,77799988 | 20,58200073 | |
| TLMF Lep 05544 | 658 | JX034610 | Noctuidae | | Standfussiana lucernea | Linnaeus | Huemer P. & Tarmann G. M. | 28-Jul-2011 | Macedonia | | | Mavrovo NP, Korab, Korabska jezero, Kobilino pole | 41,77799988 | 20,58200073 | |
| TLMF Lep 01292 | 307 | JX034586 | Pterophoridae | Pterophorinae | Stenoptilia alpinalis | Burmann | Ryrholm N | 04-Aug-1993 | Austria | | Osttiro | Sajathuette/ Praegraten NW | 47,03900146 | 12,35400009 | |
| TLMF Lep 01293 | 614 | HQ968552 | Pterophoridae | Pterophorinae | Stenoptilia alpinalis | Burmann | Ryrholm N | 04-Aug-1993 | Austria | | Osttiro | Sajathuette/ Praegraten NW | 47,03900146 | 12,35400009 | PTE139 PH |
| TLMF Lep 01296 | 658 | HQ968553 | Pterophoridae | Pterophorinae | Stenoptilia brigantiensis | Nel & Gibeaux | Nel J. | 06-Jul-2003 | France | Provence-Alpes-Cote d'Azur | | Col de Vars | 44,54199982 | 6,690999985 | |
| TLMF Lep 01297 | 658 | HQ968554 | Pterophoridae | Pterophorinae | Stenoptilia brigantiensis | Nel & Gibeaux | Nel J. | 19-Jul-2007 | France | Rhone-Alpes | | Tignes | 45,46099854 | 6,889999866 | 21819JN |
| TLMF Lep 01298 | 658 | HQ968555 | Pterophoridae | Pterophorinae | Stenoptilia brigantiensis | Nel & Gibeaux | Nel J. | 19-Jul-2007 | France | Rhone-Alpes | | Tignes | 45,46099854 | 6,889999866 | 21847JN |
| TLMF Lep 01267 | 648 | HQ968544 | Pterophoridae | Pterophorinae | Stenoptilia buvati | Nel & Gibeaux | Nel J. | 05-Jul-2004 | France | Provence-Alpes-Cote d'Azur | | Bois des Freaux | 45,04299927 | 6,281000137 | |

| | | | | | | | | | | | | | | | |
|---|---|---|---|---|---|---|---|---|---|---|---|---|---|---|---|
| MM00096 | 658 | HM396375 | Pterophoridae | Pterophorinae | Stenoptilia islandica | Staudinger | Marko Mutanen | | Finland | | | | Enontekioe | 68,99700165 | 20,74399948 |
| MM04137 | 658 | HM386924 | Pterophoridae | Pterophorinae | Stenoptilia islandica | Staudinger | Marko Mutanen | | Finland | | | | Enontekioe | 68,99700165 | 20,74399948 |
| MM04138 | 658 | HM386925 | Pterophoridae | Pterophorinae | Stenoptilia islandica | Staudinger | Marko Mutanen | | Finland | | | | Enontekioe | 68,99700165 | 20,74399948 |
| TLMF Lep 01768 | 658 | HQ968968 | Pterophoridae | Pterophorinae | Stenoptilia mercantourica | Nel & Gibeaux | Huemer P. | 04-Aug-2010 | France | Provence-Alpes-Cote d'Azur | | | Col Agnel | 44,68909836 | 6,98443985 |
| MM08501 | 658 | HM873931 | Psychidae | Oiketicinae | Sterrhopterix standfussi | Wocke | Marko Mutanen | 11-Jun-2005 | Finland | | Ostrobottnia borealis pars australis | | Kiiminki | 65,07099915 | 25,72500038 |
| MM08502 | 658 | HM873932 | Psychidae | Oiketicinae | Sterrhopterix standfussi | Wocke | Marko Mutanen | 13-Jun-2005 | Finland | | Ostrobottnia borealis pars australis | | Kiiminki | 65,07099915 | 25,72500038 |
| MM14127 | 658 | HM875907 | Psychidae | Oiketicinae | Sterrhopterix standfuss | Wocke | Marko Mutanen | | Finland | | Karelia borealis | | Tuupovaara | 62,375 | 30,90500069 |
| MM17518 | 658 | JF853884 | Psychidae | Oiketicinae | Sterrhopterix standfuss | Wocke | Ari Kallio | 01-Jan-1997 | Finland | | Satakunda | | Vammala | 61,24599838 | 22,90099907 |
| TLMF Lep 02756 | 658 | JF860274 | Psychidae | Oiketicinae | Sterrhopterix standfuss | Wocke | Schmid J. | 05-Jul-2004 | Switzerland | Graubunden | | | Platta, Pl. Pardatsch/ Medel Lu | 46,65800095 | 8,854000092 |
| MM09642 | 658 | HM874397 | Nepticulidae | Nepticulinae | Stigmella pretiosa | Heinemann | Marko Mutanen, Tomi Mutanen, Panu Vaelimaeki, Petri hirvone | 27-Sep-2008 | Finland | Aland | | | Eckeroe | 60,27500153 | 19,57999992 |
| MM09643 | 658 | HM874398 | Nepticulidae | Nepticulinae | Stigmella pretiosa | Heinemann | Marko Mutanen, Tomi Mutanen, Panu Vaelimaeki, Petri hirvone | 27-Sep-2008 | Finland | Aland | | | Eckeroe | 60,27500153 | 19,57999992 |
| TLMF Lep 02872 | 579 | JF860337 | Nepticulidae | Nepticulinae | Stigmella pretiosa | Heinemann | Huemer P | 04-Jul-1985 | Austria | Vorarlberg | | | Luenerkrinne | 47,05799866 | 9,770000458 |
| MM03414 | 658 | HQ570290 | Noctuidae | Oncocnemidinae | Sympistis nigrita | Boisduval | Marko Mutanen | | Finland | Lappi | Lapponia enontekiensis | | Enontekioe | 68,99700165 | 20,74399948 |
| MM03415 | 658 | HQ570291 | Noctuidae | Oncocnemidinae | Sympistis nigrita | Boisduval | Marko Mutanen | | Finland | Lappi | Lapponia enontekiensis | | Enontekioe | 68,99700165 | 20,74399948 |
| MM14552 | 658 | HM876163 | Noctuidae | Oncocnemidinae | Sympistis nigrita | Boisduval | Marko Mutanen | | Finland | | Lapponia enontekiensis | | Enontekioe | 69,06300354 | 21,10199928 |
| TLMF Lep 01840 | 658 | HQ968303 | Noctuidae | Oncocnemidinae | Sympistis nigrita | Boisduval | Deutsch H. | 20-Jun-1999 | Austria | | Salzburg | | Hochtor | 47,08330154 | 12,84500027 |
| TLMF Lep 01841 | 658 | HQ968304 | Noctuidae | Oncocnemidinae | Sympistis nigrita | Boisduval | Deutsch H. | 30-Jul-1989 | Austria | Tyrol | Osttirol, Lienzer Dolomiten | | Karlsbader Huette | 46,75999832 | 12,7986002 |
| TLMF Lep 02757 | 658 | JF860275 | Noctuidae | Oncocnemidinae | Sympistis nigrita | Boisduval | Schmid J. | 16-Jul-2009 | Switzerland | Graubunden | | | Avers-Cresta, Cucalner/Guggermue | 46,5019989 | 9,484999657 |
| TLMF Lep 03065 | 658 | JN275140 | Noctuidae | Oncocnemidinae | Sympistis nigrita | Boisduval | Huemer P. | 29-Jul-2008 | Austria | Vorarlberg | | | Umg. Goeppinger Huette, Gamsboden | 47,21749878 | 10,03470039 |
| 07PROBE-00082 | 658 | JX008349 | Noctuidae | Oncocnemidinae | Sympistis zetterstedtii | Staudinger | P.D.N. Hebert | 14-Jul-2007 | Canada | Manitoba | Churchill | 16 km E Churchill, Bird Cove, Rock Bluff A | Rockbluff A | 58,77099991 | -93,84600067 |
| 07PROBE-00083 | 658 | JX008226 | Noctuidae | Oncocnemidinae | Sympistis zetterstedtii | Staudinger | P.D.N. Hebert | 14-Jul-2007 | Canada | Manitoba | Churchill | 16 km E Churchill, Bird Cove, Rock Bluff A | Rockbluff A | 58,77099991 | -93,84600067 |
| 07PROBE-00084 | 658 | JX008434 | Noctuidae | Oncocnemidinae | Sympistis zetterstedtii | Staudinger | P.D.N. Hebert | 14-Jul-2007 | Canada | Manitoba | Churchill | 16 km E Churchill, Bird Cove, Rock Bluff A | Rockbluff A | 58,77099991 | -93,84600067 |
| 07PROBE-00085 | 656 | JX008165 | Noctuidae | Oncocnemidinae | Sympistis zetterstedtii | Staudinger | P.D.N. Hebert | 14-Jul-2007 | Canada | Manitoba | Churchill | 16 km E Churchill, Bird Cove, Rock Bluff A | Rockbluff A | 58,77099991 | -93,84600067 |
| 07PROBE-00130 | 657 | JX008373 | Noctuidae | Oncocnemidinae | Sympistis zetterstedt | Staudinger | S.VanRyswyk | 13-Jul-2007 | Canada | Manitoba | Churchill | 23 km E Churchill, Ramsay Cree | | 58,72299957 | -93,80699921 |
| 07PROBE-00142 | 658 | JX008113 | Noctuidae | Oncocnemidinae | Sympistis zetterstedtii | Staudinger | P.D.N. Hebert | 15-Jul-2007 | Canada | Manitoba | Churchill | 16 km E Churchill, Bird Cove, Rock Bluff C | | 58,76699829 | -93,86799622 |
| 07PROBE-00143 | 658 | JX008118 | Noctuidae | Oncocnemidinae | Sympistis zetterstedtii | Staudinger | P.D.N. Hebert | 15-Jul-2007 | Canada | Manitoba | Churchill | 16 km E Churchill, Bird Cove, Rock Bluff C | | 58,76699829 | -93,86799622 |
| 07PROBE-00144 | 658 | JX008141 | Noctuidae | Oncocnemidinae | Sympistis zetterstedtii | Staudinger | P.D.N. Hebert | 15-Jul-2007 | Canada | Manitoba | Churchill | 16 km E Churchill, Bird Cove, Rock Bluff C | | 58,76699829 | -93,86799622 |
| 07PROBE-00527 | 655 | JX008270 | Noctuidae | Oncocnemidinae | Sympistis zetterstedt | Staudinger | J.deWaard | 13-Jul-2007 | Canada | Manitoba | Churchill | Town of Churchil | field S of Churchill train statior | 58,76699829 | -94,1760025 |
| 07PROBE-00529 | 653 | JX008396 | Noctuidae | Oncocnemidinae | Sympistis zetterstedt | Staudinger | J.deWaard | 13-Jul-2007 | Canada | Manitoba | Churchill | Town of Churchil | field S of Churchill train statior | 58,76699829 | -94,1760025 |
| 07PROBE-03905 | 658 | JX008177 | Noctuidae | Oncocnemidinae | Sympistis zetterstedt | Staudinger | P.D.N. Hebert | 18-Jul-2007 | Canada | Manitoba | Churchill | 16 km E Churchill, Bird Cove | Tundra Pond Area | 58,76800156 | -93,85800171 |
| 07PROBE-03906 | 656 | JX008311 | Noctuidae | Oncocnemidinae | Sympistis zetterstedt | Staudinger | P.D.N. Hebert | 18-Jul-2007 | Canada | Manitoba | Churchill | 16 km E Churchill, Bird Cove | Tundra Pond Area | 58,76800156 | -93,85800171 |
| 07PROBE-03907 | 658 | JX008372 | Noctuidae | Oncocnemidinae | Sympistis zetterstedt | Staudinger | P.D.N. Hebert | 18-Jul-2007 | Canada | Manitoba | Churchill | 16 km E Churchill, Bird Cove | Tundra Pond Area | 58,76800156 | -93,85800171 |
| 07PROBE-10049 | 658 | JX008279 | Noctuidae | Oncocnemidinae | Sympistis zetterstedt | Staudinger | P.D.N. Hebert | 16-Jul-2007 | Canada | Manitoba | Churchill | 13 km E Churchill, Eastern Creek | | 58,75500107 | -93,94400024 |
| 07PROBE-10050 | 658 | JX008295 | Noctuidae | Oncocnemidinae | Sympistis zetterstedt | Staudinger | P.D.N. Hebert | 16-Jul-2007 | Canada | Manitoba | Churchill | 13 km E Churchill, Eastern Creek | | 58,75500107 | -93,94400024 |
| 07PROBE-10051 | 658 | JX008285 | Noctuidae | Oncocnemidinae | Sympistis zetterstedt | Staudinger | P.D.N. Hebert | 16-Jul-2007 | Canada | Manitoba | Churchill | 13 km E Churchill, Eastern Creek | | 58,75500107 | -93,94400024 |
| 07PROBE-10053 | 658 | JX008417 | Noctuidae | Oncocnemidinae | Sympistis zetterstedt | Staudinger | P.D.N. Hebert | 16-Jul-2007 | Canada | Manitoba | Churchill | 13 km E Churchill, Eastern Creek | | 58,75500107 | -93,94400024 |
| 07PROBE-10111 | 658 | JX008316 | Noctuidae | Oncocnemidinae | Sympistis zetterstedt | Staudinger | P.D.N. Hebert | 18-Jul-2007 | Canada | Manitoba | Churchill | 16 km E Churchill, tundra pond | | 58,75500107 | -93,91500092 |
| 07PROBE-10114 | 658 | JX008451 | Noctuidae | Oncocnemidinae | Sympistis zetterstedt | Staudinger | P.D.N. Hebert | 18-Jul-2007 | Canada | Manitoba | Churchill | 16 km E Churchill, tundra pond | | 58,75500107 | -93,91500092 |
| 07PROBE-10255 | 658 | JX008495 | Noctuidae | Oncocnemidinae | Sympistis zetterstedt | Staudinger | P.D.N. Hebert | 22-Jul-2007 | Canada | Manitoba | Churchill | 5 km E Churchill, Miss Piggy | | 58,75999832 | -94,08599854 |
| 07PROBE-10683 | 658 | JX008406 | Noctuidae | Oncocnemidinae | Sympistis zetterstedt | Staudinger | P.D.N. Hebert | 22-Jul-2007 | Canada | Manitoba | Churchill | 23 km E Churchill, Ramsay Cree | | 58,73099899 | -93,77999878 |
| 07PROBE-10684 | 658 | JX008292 | Noctuidae | Oncocnemidinae | Sympistis zetterstedt | Staudinger | P.D.N. Hebert | 22-Jul-2007 | Canada | Manitoba | Churchill | 23 km E Churchill, Ramsay Cree | | 58,73099899 | -93,77999878 |
| 07WNP-10798 | 658 | JX008323 | Noctuidae | Oncocnemidinae | Sympistis zetterstedt | Staudinger | J.McGowan | 27-Jul-2007 | Canada | Manitoba | Churchill | 22 km E Churchill, CNSC | | 58,72999954 | -93,81999969 |
| 07WNP-10806 | 658 | JX008463 | Noctuidae | Oncocnemidinae | Sympistis zetterstedtii | Staudinger | J.McGowan | 16-Jul-2007 | Canada | Manitoba | Churchill | 16 km E Churchill, Bird Cove, Rock Bluff B | | 58,77000046 | -93,84300232 |
| 07WNP-10816 | 658 | JX008398 | Noctuidae | Oncocnemidinae | Sympistis zetterstedtii | Staudinger | J.McGowan | 06-Jul-2007 | Canada | Manitoba | Churchill | Wapusk National Park, unspecified locality | sandy area | 58,64300156 | -93,22599792 |
| 07WNP-10817 | 658 | JX008440 | Noctuidae | Oncocnemidinae | Sympistis zetterstedtii | Staudinger | J.McGowan | 06-Jul-2007 | Canada | Manitoba | Churchill | Wapusk National Park, unspecified locality | sandy area | 58,64300156 | -93,22599792 |
| 09PROBE-09518 | 640 | HM375771 | Noctuidae | Oncocnemidinae | Sympistis zetterstedt | Staudinger | P.D.N. Hebert | 17-Jul-2009 | Canada | Manitoba | Churchill | 22 km E Churchill, CNSC | | 58,73799896 | -93,81900024 |
| 09PROBE-09573 | 658 | HM375822 | Noctuidae | Oncocnemidinae | Sympistis zetterstedt | Staudinger | P.D.N. Hebert | 21-Jul-2009 | Canada | Manitoba | Churchill | 14 km SW Churchill, Button Bay | | 58,80500031 | -94,21399689 |
| 09PROBE-09574 | 636 | HM375823 | Noctuidae | Oncocnemidinae | Sympistis zetterstedt | Staudinger | P.D.N. Hebert | 21-Jul-2009 | Canada | Manitoba | Churchill | 14 km SW Churchill, Button Bay | | 58,80500031 | -94,21399689 |
| CHU06-COL-104 | 620 | JX008243 | Noctuidae | Oncocnemidinae | Sympistis zetterstedtii | Staudinger | Arctic & Boreal Entomology | 09-Aug-2006 | Canada | Manitoba | Churchill | 16 km E Churchill, krummholz formation | Tundra | 58,75400162 | -93,91300201 |
| CHU06-COL-110 | 489 | JX008382 | Noctuidae | Oncocnemidinae | Sympistis zetterstedt | Staudinger | Arctic & Boreal Entomology | 06-Aug-2006 | Canada | Manitoba | Churchill | 11 km S Churchill, Goose Cree | Churchill-Goose creek Rd.NSC | 58,6629982 | -94,16699982 |
| CHU06-COL-158 | 658 | JX008465 | Noctuidae | Oncocnemidinae | Sympistis zetterstedtii | Staudinger | Arctic & Boreal Entomology | 18-Aug-2006 | Canada | Manitoba | Churchill | 16 km E Churchill, krummholz formation | Tundra | 58,75400162 | -93,91300201 |
| CHU06-COL-159 | 658 | JX008277 | Noctuidae | Oncocnemidinae | Sympistis zetterstedtii | Staudinger | Arctic & Boreal Entomology | 18-Aug-2006 | Canada | Manitoba | Churchill | 16 km E Churchill, krummholz formation | Tundra | 58,75400162 | -93,91300201 |
| CHU06-COL-161 | 654 | JX008431 | Noctuidae | Oncocnemidinae | Sympistis zetterstedtii | Staudinger | Arctic & Boreal Entomology | 18-Aug-2006 | Canada | Manitoba | Churchill | 16 km E Churchill, krummholz formation | Tundra | 58,75400162 | -93,91300201 |
| CHU06-COL-163 | 658 | JX008253 | Noctuidae | Oncocnemidinae | Sympistis zetterstedtii | Staudinger | Arctic & Boreal Entomology | 18-Aug-2006 | Canada | Manitoba | Churchill | 16 km E Churchill, krummholz formation | Tundra | 58,75400162 | -93,91300201 |
| CHU06-COL-166 | 645 | JX008183 | Noctuidae | Oncocnemidinae | Sympistis zetterstedtii | Staudinger | Arctic & Boreal Entomology | 18-Aug-2006 | Canada | Manitoba | Churchill | 16 km E Churchill, krummholz formation | Tundra | 58,75400162 | -93,91300201 |
| CHU06-LEP-027 | 658 | JX008248 | Noctuidae | Oncocnemidinae | Sympistis zetterstedt | Staudinger | Arctic & Boreal Entomology | 12-Aug-2006 | Canada | Manitoba | Churchill | 23 km E Churchill, Ramsay Cree | Stream | 58,73099899 | -93,78199768 |
| CNCNoctuoidea1069 | 573 | JX008218 | Noctuidae | Oncocnemidinae | Sympistis zetterstedt | Staudinger | H. Hense | 05-Jul-1995 | Canada | Manitoba | | | 24km E of Churchil | | |
| CNCNoctuoidea1069 | 562 | JX008154 | Noctuidae | Oncocnemidinae | Sympistis zetterstedt | Staudinger | H. Hense | 05-Jul-1995 | Canada | Manitoba | | | 24km E of Churchil | | |
| CNCNoctuoidea1069 | 517 | JX008216 | Noctuidae | Oncocnemidinae | Sympistis zetterstedt | Staudinger | H. Hense | 02-Jul-1995 | Canada | Manitoba | | | 24km E of Churchil | | |
| CNCNoctuoidea1069 | 586 | JX008112 | Noctuidae | Oncocnemidinae | Sympistis zetterstedt | Staudinger | D. Bowman | 16-Jul-1998 | United States | Colorado | | | Loveland Pass | | |
| CNCNoctuoidea1069 | 614 | JX008464 | Noctuidae | Oncocnemidinae | Sympistis zetterstedt | Staudinger | J. Troubridge | 28-Jun-1998 | Canada | British Columbia | | | Pink Mountain | 57,04999924 | -122,8499985 |
| CNCNoctuoidea1070 | 477 | JX008254 | Noctuidae | Oncocnemidinae | Sympistis zetterstedt | Staudinger | D. Bowman | 20-Jul-1998 | United States | Colorado | | | Loveland Pass | | |
| CNCNoctuoidea1070 | 551 | JX008194 | Noctuidae | Oncocnemidinae | Sympistis zetterstedt | Staudinger | J. Troubridge | 28-Jun-1998 | Canada | British Columbia | | | Pink Mountain | 57,04999924 | -122,8499985 |

| | | | | | | | | | | | | | | | | |
|---|---|---|---|---|---|---|---|---|---|---|---|---|---|---|---|---|
| LEP037938 | 658 | JX008449 | Noctuidae | Oncocnemidinae | Sympistis zetterstedtii | Staudinger | C. S. Guppy & G.E. Hutchings | | Canada | Yukon Territory | Swede Dome, Top of the World Hwy Km. 64, rock rubble | | | | | |
| LEP037946 | 658 | JX008156 | Noctuidae | Oncocnemidinae | Sympistis zetterstedtii | Staudinger | C. S. Guppy & G.E. Hutchings | | Canada | Yukon Territory | Dempster Hwy. Km. 152.8 ( E of Hwy.), limestone rock rubble & tundra | | | | | |
| LEP037947 | 658 | JX008347 | Noctuidae | Oncocnemidinae | Sympistis zetterstedtii | Staudinger | C. S. Guppy & G.E. Hutchings | | Canada | Yukon Territory | Mt. Klotz ( SW. of ), rock rubblee slope | | | | | |
| LEP037955 | 646 | JX008140 | Noctuidae | Oncocnemidinae | Sympistis zetterstedtii | Staudinger | C. Schmidt | | Canada | Yukon Territory | Montana Mtn., 10 km. S. Carcross, dry tundra | | | | | |
| LEP037956 | 658 | JX008381 | Noctuidae | Oncocnemidinae | Sympistis zetterstedtii | Staudinger | C. Schmidt | | Canada | Yukon Territory | Montana Mtn., 10 km. S. Carcross, dry tundra | | | | | |
| LEP037957 | 658 | JX008107 | Noctuidae | Oncocnemidinae | Sympistis zetterstedtii | Staudinger | C. Schmidt | | Canada | Yukon Territory | Montana Mtn., 10 km. S. Carcross, dry tundra | | | | | |
| LEP037958 | 658 | JX008446 | Noctuidae | Oncocnemidinae | Sympistis zetterstedtii | Staudinger | C. Schmidt | | Canada | Yukon Territory | Montana Mtn., 10 km. S. Carcross, dry tundra | | | | | |
| LEP037959 | 658 | JX008293 | Noctuidae | Oncocnemidinae | Sympistis zetterstedtii | Staudinger | C. Schmidt | | Canada | Yukon Territory | Montana Mtn., 10 km. S. Carcross, dry tundra | | | | | |
| MM15761 | 456 | JX034648 | Sesiidae | Sesiinae | Synanthedon polaris | Staudinger | Tomi Mutanen, Panu Vaelimae | | Finland | | Lapponia enontekiensis | | Enontekioe | 68,99700165 | 20,74399948 | |
| MM18778 | 658 | JX034613 | Sesiidae | Sesiinae | Synanthedon polaris | Staudinger | Reima Leinonen | | Finland | | Lapponia inarensis | | Utsjok | 69,95999908 | 27,96699905 | |
| MM18785 | 658 | JF854617 | Sesiidae | Sesiinae | Synanthedon polaris | Staudinger | Reima Leinonen | 18-Jul-2003 | Finland | | Ostrobottnia kajanensi | | Suomussalm | 65,02200317 | 29,33399963 | |
| TLMF Lep 02759 | 658 | JF860277 | Sesiidae | Sesiinae | Synanthedon polaris | Staudinger | Schmid J. | 03-Jun-1996 | Switzerland | Graubunder | | | Tigels, Berninapass | 46,44100189 | 9,984999657 | |
| MM02565 | 658 | HM871613 | Gelechiidae | Gelechiinae | Syncopacma karvoneni | Hackman | Marko Mutanen, Panu Vaelimael | | Finland | | Savonia australis | | Imatra | 61,10800171 | 28,79899979 | |
| MM02566 | 658 | JX034569 | Gelechiidae | Gelechiinae | Syncopacma karvoneni | Hackman | Marko Mutanen, Panu Vaelimael | | Finland | | Savonia australis | | Imatra | 61,10800171 | 28,79899979 | |
| MM02567 | 658 | JX034695 | Gelechiidae | Gelechiinae | Syncopacma karvoneni | Hackman | Marko Mutanen, Panu Vaelimael | | Finland | | Savonia australis | | Imatra | 61,10800171 | 28,79899979 | |
| MM06354 | 658 | HM873221 | Gelechiidae | Gelechiinae | Syncopacma karvoneni | Hackman | Marko Mutanen, Anttoni Mutanen, Nestori Mutanen | 29-Jun-2007 | Finland | | Lapponia inarensis | | Utsjoki | 69,88200378 | 27 | |
| MM09971 | 658 | HM874508 | Gelechiidae | Gelechiinae | Syncopacma karvoneni | Hackman | Marko Mutanen | 17-Jun-2008 | Finland | | Karelia borealis | | Kesaelaht | 61,89899826 | 29,79700089 | |
| MM10005 | 658 | JF853457 | Gelechiidae | Gelechiinae | Syncopacma karvoneni | Hackman | Marko Mutanen | 16-Jun-2008 | Finland | | Karelia borealis | | Kitee | 62,04199982 | 30,25 | |
| MM10034 | 658 | JF853460 | Gelechiidae | Gelechiinae | Syncopacma karvoneni | Hackman | Marko Mutanen | 18-Jun-2008 | Finland | | Savonia australis | | Imatra | 61,13499832 | 28,79999924 | |
| MM14076 | 658 | HM875876 | Gelechiidae | Gelechiinae | Syncopacma karvoneni | Hackman | Marko Mutanen | | Finland | | Karelia borealis | | Tuupovaara | 62,375 | 30,90500069 | |
| MM18247 | 658 | JF854240 | Gelechiidae | Gelechiinae | Syncopacma karvoneni | Hackman | Marko Mutanen | 27-Jun-2002 | Finland | | Lapponia inarensis | | Inari | 69,28559875 | 25,73390007 | |
| MM18248 | 658 | JF854241 | Gelechiidae | Gelechiinae | Syncopacma karvoneni | Hackman | Marko Mutanen | 29-Jun-2007 | Finland | | Lapponia inarensis | | Inari | 69,88179779 | 27 | |
| TLMF Lep 02758 | 658 | JF860276 | Gelechiidae | Gelechiinae | Syncopacma karvoneni | Hackman | Schmid J. | 14-Jun-2008 | Switzerland | Graubunder | | | Umg. Prasegras/ Berguen | 46,6269989 | 9,755999565 | genitalia on card boa |
| MM00072 | 658 | HM396351 | Noctuidae | Plusiinae | Syngrapha hochenwarthi | Hochenwarth | Marko Mutanen | | Finland | | | | Enontekioe | 68,99700165 | 20,74399948 | |
| MM04097 | 658 | HM386887 | Noctuidae | Plusiinae | Syngrapha hochenwarthi | Hochenwarth | Marko Mutanen | | Finland | | | | Enontekioe | 68,99700165 | 20,74399948 | |
| MM05909 | 647 | HQ570322 | Noctuidae | Plusiinae | Syngrapha hochenwarthi | Hochenwarth | Panu Vaelimaek | 03-Jul-2007 | Finland | | Lapponia inarensis | | Utsjok | 69,375 | 25,72900009 | |
| TLMF Lep 00172 | 657 | GU689159 | Noctuidae | Plusiinae | Syngrapha hochenwarthi | Hochenwarth | Huemer P | 25-Aug-2008 | Switzerland | Graubunder | | Umbrailpass | | 46,54199982 | 10,43400002 | |
| TLMF Lep 00173 | 657 | GU689153 | Noctuidae | Plusiinae | Syngrapha hochenwarthi | Hochenwarth | Huemer P. | 31-Jul-2006 | Austria | Salzburg | Hohe Tauern | Rauris: Grossglocknerstrasse, Hochtor . PG-0004 | | 47,08300018 | 12,84399986 | |
| TLMF Lep 02832 | 658 | JF860314 | Noctuidae | Plusiinae | Syngrapha hochenwarthi | Hochenwarth | Huemer P. & Erlebach S | 11-Apr-2009 | Switzerland | Graubunder | | | Umbrailpass | 46,54199982 | 10,43400002 | |
| TLMF Lep 02833 | 658 | JF860315 | Noctuidae | Plusiinae | Syngrapha hochenwarthi | Hochenwarth | Huemer P. & Erlebach S | 11-Apr-2009 | Switzerland | Graubunder | | | Umbrailpass | 46,54199982 | 10,43400002 | |
| CNCLEP00026601 | 634 | JX008119 | Douglasiidae | Douglasiinae | Tinagma dryadis | Staudinger | JF Landry & G Poh | | Canada | Yukon Territory | | Whitehorse | Grey Mtn summi | 60,67499924 | -134,904007 | |
| CNCLEP00026602 | 634 | JX008217 | Douglasiidae | Douglasiinae | Tinagma dryadis | Staudinger | JF Landry & G Poh | | Canada | Yukon Territory | | Whitehorse | Grey Mtn summi | 60,67499924 | -134,904007 | |
| CNCLEP00026603 | 634 | JX008412 | Douglasiidae | Douglasiinae | Tinagma dryadis | Staudinger | JF Landry & G Poh | | Canada | Yukon Territory | | Whitehorse | Grey Mtn summi | 60,67499924 | -134,904007 | |
| CNCLEP00026605 | 632 | JX008310 | Douglasiidae | Douglasiinae | Tinagma dryadis | Staudinger | JF Landry & G Poh | | Canada | Yukon Territory | | Whitehorse | Grey Mtn summi | 60,67499924 | -134,904007 | |
| CNCLEP00026606 | 632 | JX008229 | Douglasiidae | Douglasiinae | Tinagma dryadis | Staudinger | JF Landry & G Poh | | Canada | Yukon Territory | | Whitehorse | Grey Mtn summi | 60,67499924 | -134,904007 | |
| CNCLEP00026607 | 632 | JX008435 | Douglasiidae | Douglasiinae | Tinagma dryadis | Staudinger | JF Landry & G Poh | | Canada | Yukon Territory | | Whitehorse | Grey Mtn summi | 60,67499924 | -134,904007 | |
| CNCLEP00026608 | 618 | JX008325 | Douglasiidae | Douglasiinae | Tinagma dryadis | Staudinger | JF Landry & G Poh | | Canada | Yukon Territory | | Whitehorse | Grey Mtn summi | 60,67499924 | -134,904007 | |
| CNCLEP00026609 | 634 | JX008215 | Douglasiidae | Douglasiinae | Tinagma dryadis | Staudinger | JF Landry & G Poh | | Canada | Yukon Territory | | Whitehorse | Grey Mtn summi | 60,67499924 | -134,904007 | |
| CNCLEP00026610 | 634 | JX008304 | Douglasiidae | Douglasiinae | Tinagma dryadis | Staudinger | JF Landry & G Poh | | Canada | Yukon Territory | | Whitehorse | Grey Mtn summi | 60,67499924 | -134,904007 | |
| CNCLEP00026611 | 634 | JX008437 | Douglasiidae | Douglasiinae | Tinagma dryadis | Staudinger | JF Landry & G Poh | | Canada | Yukon Territory | | Whitehorse | Grey Mtn summi | 60,67499924 | -134,904007 | |
| CNCLEP00026612 | 634 | JX008197 | Douglasiidae | Douglasiinae | Tinagma dryadis | Staudinger | JF Landry & G Poh | | Canada | Yukon Territory | | Whitehorse | Grey Mtn summi | 60,67499924 | -134,904007 | |
| CNCLEP00026613 | 634 | JX008383 | Douglasiidae | Douglasiinae | Tinagma dryadis | Staudinger | JF Landry & G Poh | | Canada | Yukon Territory | | Whitehorse | Grey Mtn summi | 60,67499924 | -134,904007 | |
| CNCLEP00026614 | 634 | JX008322 | Douglasiidae | Douglasiinae | Tinagma dryadis | Staudinger | JF Landry & G Poh | | Canada | Yukon Territory | | Whitehorse | Grey Mtn summi | 60,67499924 | -134,904007 | |
| CNCLEP00026615 | 632 | JX008168 | Douglasiidae | Douglasiinae | Tinagma dryadis | Staudinger | JF Landry & G Poh | | Canada | Yukon Territory | | Whitehorse | Grey Mtn summi | 60,67499924 | -134,904007 | |
| CNCLEP00026616 | 634 | JX008263 | Douglasiidae | Douglasiinae | Tinagma dryadis | Staudinger | JF Landry & G Poh | | Canada | Yukon Territory | | Whitehorse | Grey Mtn summi | 60,67499924 | -134,904007 | |
| CNCLEP00026617 | 634 | JX008160 | Douglasiidae | Douglasiinae | Tinagma dryadis | Staudinger | JF Landry & G Poh | | Canada | Yukon Territory | | Whitehorse | Grey Mtn summi | 60,67499924 | -134,904007 | |
| CNCLEP00026618 | 634 | JX008294 | Douglasiidae | Douglasiinae | Tinagma dryadis | Staudinger | JF Landry & G Poh | | Canada | Yukon Territory | | Whitehorse | Grey Mtn summi | 60,67499924 | -134,904007 | |
| CNCLEP00026619 | 634 | JX008359 | Douglasiidae | Douglasiinae | Tinagma dryadis | Staudinger | JF Landry & G Poh | | Canada | Yukon Territory | | Whitehorse | Grey Mtn summi | 60,67499924 | -134,904007 | |
| CNCLEP00026620 | 610 | JX008201 | Douglasiidae | Douglasiinae | Tinagma dryadis | Staudinger | JF Landry & G Poh | | Canada | Yukon Territory | | Whitehorse | Grey Mtn summi | 60,67499924 | -134,904007 | |
| CNCLEP00026621 | 634 | JX008460 | Douglasiidae | Douglasiinae | Tinagma dryadis | Staudinger | JF Landry & G Poh | | Canada | Yukon Territory | | Whitehorse | Grey Mtn summi | 60,67499924 | -134,904007 | MIC5501 F |
| CNCLEP00026622 | 634 | JX008144 | Douglasiidae | Douglasiinae | Tinagma dryadis | Staudinger | JF Landry & G Poh | | Canada | Yukon Territory | | Whitehorse | Grey Mtn summi | 60,67499924 | -134,904007 | MIC5500 M |
| CNCLEP00026623 | 634 | JX008400 | Douglasiidae | Douglasiinae | Tinagma dryadis | Staudinger | JF Landry & G Poh | | Canada | Yukon Territory | | Whitehorse | Grey Mtn summi | 60,67499924 | -134,904007 | |
| MM00087 | 658 | HM396366 | Douglasiidae | Douglasiinae | Tinagma dryadis | Staudinger | Marko Mutanen | | Finland | | | | Enontekioe | 68,99700165 | 20,74399948 | |
| MM03964 | 658 | HQ570307 | Douglasiidae | Douglasiinae | Tinagma dryadis | Staudinger | Marko Mutanen | 05-Jul-2006 | Finland | Lappi | Lapponia enontekiensi | | Enontekioe | 68,99700165 | 20,74399948 | |
| MM03965 | 658 | HQ570308 | Douglasiidae | Douglasiinae | Tinagma dryadis | Staudinger | Marko Mutanen | 05-Jul-2006 | Finland | Lappi | Lapponia enontekiensi | | Enontekioe | 68,99700165 | 20,74399948 | |
| TLMF Lep 02763 | 658 | JF860280 | Douglasiidae | Douglasiinae | Tinagma dryadis | Staudinger | Schmid J. | 07-Jul-2007 | Switzerland | Graubunder | | | Alp Mora - Bargis/ Trin | 46,5929985 | 10,28800011 | |
| MM01862 | 658 | HM386852 | Crambidae | Spilomelinae | Udea decrepitalis | Herrich-Schäffer | Marko Mutanen, Panu Vaelimael | | Finland | | | | Imatra | 61,10800171 | 28,79899979 | |
| MM01863 | 658 | HM386853 | Crambidae | Spilomelinae | Udea decrepitalis | Herrich-Schäffer | Marko Mutanen, Panu Vaelimael | | Finland | | | | Imatra | 61,10800171 | 28,79899979 | |
| MM02941 | 658 | HM871750 | Crambidae | Spilomelinae | Udea decrepitalis | Herrich-Schäffer | Marko Mutanen | | Finland | | Ostrobottnia borealis pars australis | | Kiiminki | 65,07099915 | 25,72500038 | |
| TLMF Lep 00821 | 658 | HQ968197 | Crambidae | Spilomelinae | Udea decrepitalis | Herrich-Schäffer | Huemer P | 04-Jul-2009 | Austria | Steiermark | | | Turracher Hoehe | 46,9280014 | 13,86800003 | |
| TLMF Lep 00822 | 658 | HQ968198 | Crambidae | Spilomelinae | Udea decrepitalis | Herrich-Schäffer | Huemer P | 04-Jul-2009 | Austria | Steiermark | | | Turracher Hoehe | 46,9280014 | 13,86800003 | |
| TLMF Lep 00825 | 612 | HQ968201 | Crambidae | Spilomelinae | Udea decrepitalis | Herrich-Schäffer | Huemer P | 01-Jul-2009 | Austria | Karnten | | | Umg. Zollnerseehuett | 46,60599899 | 13,0710001 | |
| TLMF Lep 02764 | 658 | JF860281 | Crambidae | Spilomelinae | Udea decrepitalis | Herrich-Schäffer | Schmid J. | 22-Jun-2005 | Switzerland | Graubunder | | | Riefawald/ Vals | 46,61800003 | 9,194000244 | |
| MM19185 | 622 | JF854517 | Crambidae | Spilomelinae | Udea elutalis | Denis & Schiffermüller | Kari Nupponen | 14-Jul-2003 | Russia | | Cheliabinsk oblas | | Miass | | | |
| MM19186 | 658 | JF854518 | Crambidae | Spilomelinae | Udea elutalis | Denis & Schiffermüller | Kari Nupponen | 14-Jul-2003 | Russia | | Cheliabinsk oblas | | Miass | | | |
| MM19306 | 658 | JX034659 | Crambidae | Spilomelinae | Udea elutalis | Denis & Schiffermüller | Kari Nupponen | | Finland | | Kareliya | | Hamina, Melti | 60,5719986 | 27,20100021 | |
| TLMF Lep 02765 | 658 | JF860282 | Crambidae | Spilomelinae | Udea elutalis | Denis & Schiffermüller | Schmid J. | 30-Jun-2008 | Switzerland | Graubunden | Sesvenna | | Santa Maria in Muestair, Costeras | 46,60699844 | 10,42399979 | |
| 07PROBE-00073 | 658 | JX008454 | Crambidae | Spilomelinae | Udea inquinatalis | Lienig & Zelle | P.D.N. Hebert | 14-Jul-2007 | Canada | Manitoba | Churchill | 23 km E Churchill, Ramsay Cree | | 58,72299957 | -93,80699921 | |
| 07PROBE-00496 | 658 | JX008397 | Crambidae | Spilomelinae | Udea inquinatalis | Lienig & Zelle | J.deWaard | 13-Jul-2007 | Canada | Manitoba | Churchill | Town of Churchil | field S of Churchill train statior | 58,76699829 | -94,1760025 | |
| 07PROBE-00518 | 655 | JX008312 | Crambidae | Spilomelinae | Udea inquinatalis | Lienig & Zelle | J.deWaard | 13-Jul-2007 | Canada | Manitoba | Churchill | Town of Churchil | field S of Churchill train statior | 58,76699829 | -94,1760025 | |
| 07PROBE-00603 | 658 | JX008353 | Crambidae | Spilomelinae | Udea inquinatalis | Lienig & Zelle | P.D.N. Hebert | 15-Jul-2007 | Canada | Manitoba | Churchill | 26 km SE Churchill, Twin Lake | | 58,63199997 | -93,81900024 | |
| 07PROBE-10236 | 652 | JX008484 | Crambidae | Spilomelinae | Udea inquinatalis | Lienig & Zelle | P.D.N. Hebert | 21-Jul-2007 | Canada | Manitoba | Churchill | Town of Churchil | | 58,76900101 | -94,17199707 | |
| 07PROBE-10237 | 645 | JX008108 | Crambidae | Spilomelinae | Udea inquinatalis | Lienig & Zelle | P.D.N. Hebert | 21-Jul-2007 | Canada | Manitoba | Churchill | Town of Churchil | | 58,76900101 | -94,17199707 | |
| 07PROBE-10757 | 658 | JX008364 | Crambidae | Spilomelinae | Udea inquinatalis | Lienig & Zelle | P.D.N. Hebert | 23-Jul-2007 | Canada | Manitoba | Churchill | 26 km SE Churchill, Twin Lake | | 58,63000107 | -93,81900024 | |
| 07PROBE-10764 | 658 | JX008355 | Crambidae | Spilomelinae | Udea inquinatalis | Lienig & Zelle | P.D.N. Hebert | 23-Jul-2007 | Canada | Manitoba | Churchill | 26 km SE Churchill, Twin Lake | | 58,63000107 | -93,81900024 | |

| | | | | | | | | | | | | | | | |
|---|---|---|---|---|---|---|---|---|---|---|---|---|---|---|---|
| CHU06-COL-21 | 658 | JX008187 | Crambidae | Spilomelinae | Udea inquinatalis | Lienig & Zeller | Arctic & Boreal Entomology | 10-Aug-2006 | Canada | Manitoba | Churchill | 23 km E Churchill, Ramsay Cree | Small Creek | 58,73099899 | -93,77999878 |
| MM04133 | 658 | HM386920 | Crambidae | Spilomelinae | Udea inquinatalis | Lienig & Zeller | Marko Mutanen | | Finland | | | | Enontekioe | 68,99700165 | 20,74399948 |
| MM04139 | 658 | HM386926 | Crambidae | Spilomelinae | Udea inquinatalis | Lienig & Zeller | Marko Mutanen | | Finland | | | | Enontekioe | 68,99700165 | 20,74399948 |
| MM14586 | 658 | HQ570404 | Crambidae | Spilomelinae | Udea inquinatalis | Lienig & Zeller | Marko Mutanen | | Finland | | Lapponia enontekiensis | | Enontekioe | 68,99700165 | 20,74399948 |
| TLMF Lep 00854 | 658 | HQ968228 | Crambidae | Spilomelinae | Udea inquinatalis | Lienig & Zeller | Huemer P | 01-Jul-2009 | Austria | Karnten | | | Umg. Zollnerseehuett | 46,60599899 | 13,0710001 |
| TLMF Lep 00857 | 658 | HQ968231 | Crambidae | Spilomelinae | Udea inquinatalis | Lienig & Zeller | Huemer P | 02-Jul-2009 | Austria | Karnten | | | Umg. Zollnerseehuett | 46,60599899 | 13,0710001 |
| TLMF Lep 02834 | 658 | JF860316 | Crambidae | Spilomelinae | Udea inquinatalis | Lienig & Zeller | Huemer P | 03-Jul-2010 | Austria | Vorarlberg | | | Partenen, N Vermuntstause | 46,93899918 | 10,05700016 |
| MM06605 | 658 | HM873360 | Crambidae | Spilomelinae | Udea nebulalis | Hübner | Tomi Mutanen, Marko Mutanen, Nestori Mutanen, Anttoni Mutanen | 05-Jul-2007 | Finland | | Karelia borealis | | Ilomantsi | 62,50999832 | 30,88400078 |
| MM08327 | 658 | HM873870 | Crambidae | Spilomelinae | Udea nebulalis | Hübner | Marko Mutanen | | Finland | | Ostrobottnia borealis pars borealis | | Tornio | 65,89800262 | 24,45499992 |
| MM13868 | 642 | HM387073 | Crambidae | Spilomelinae | Udea nebulalis | Hübner | M. Mutanen | | Finland | | | | Tornio | 65,89800262 | 24,45499992 |
| TLMF Lep 00569 | 658 | HM381411 | Crambidae | Spilomelinae | Udea nebulalis | Hübner | Huemer P. | 20-Jul-2009 | Italy | | Belluno | | Passo di Valparola E - Passo Falzarego | 46,5223999 | 12,00699997 |
| TLMF Lep 00914 | 658 | HM381486 | Crambidae | Spilomelinae | Udea nebulalis | Hübner | Huemer P. | 29-May-2009 | Austria | Vorarlberg | | | Sonntag, Buchboden, S Unter-Ueberlutalpe | 47,25099945 | 9,970999718 |
| TLMF Lep 01705 | 658 | HQ968909 | Crambidae | Spilomelinae | Udea nebulalis | Hübner | Huemer P | 09-Aug-2010 | Slovenia | | | | Veliki Mangar | 46,43849945 | 13,63529968 |
| TLMF Lep 02074 | 658 | JF859663 | Crambidae | Spilomelinae | Udea nebulalis | Hübner | Huemer P | 01-Jul-2010 | Italy | South Tyrol | | | Ritten/ Obergruenwal | 46,59700012 | 11,43900013 |
| TLMF Lep 02766 | 658 | JF860283 | Crambidae | Spilomelinae | Udea nebulalis | Hübner | Schmid J. | 25-Jul-2006 | Switzerland | Graubunden | | | Nagens, Uaul Gonda/ Laa | 46,84799957 | 9,225999832 |
| 07PROBE-10693 | 658 | JX008448 | Geometridae | Larentiinae | Xanthorhoe alticolata | Barnes & McDunnough | P.D.N. Hebert | | Canada | Manitoba | Churchill | unspecified locality | (general coordinate) | 58,74000168 | -93,81999969 |
| 07PROBE-10920 | 658 | JX008135 | Geometridae | Larentiinae | Xanthorhoe alticolata | Barnes & McDunnough | P.D.N. Hebert | 23-Jul-2007 | Canada | Manitoba | Churchill | unspecified locality | (general coordinate) | 58,74000168 | -93,81999969 |
| 08BBLEP-04216 | 658 | JX008298 | Geometridae | Larentiinae | Xanthorhoe alticolata | Barnes & McDunnough | J.Straka,J.Cossey | 31-Jul-2008 | Canada | Alberta | Banff | Banff Nat. Park - Cave and Basin Area | Spring-fed marsh/meadow | 51,17100143 | -115,5879974 |
| 08BBLEP-04262 | 658 | JX008257 | Geometridae | Larentiinae | Xanthorhoe alticolata | Barnes & McDunnough | J.Straka,J.Cossey | 04-Aug-2008 | Canada | Alberta | Banff | Banff Nat. Park - Johnson Lake Area | Meadow, beside creek and lake | 51,19800186 | -115,4789963 |
| 08BBLEP-04446 | 636 | JX008476 | Geometridae | Larentiinae | Xanthorhoe alticolata | Barnes & McDunnough | J.Straka,J.Cossey | 31-Jul-2008 | Canada | Alberta | Banff | Banff Nat. Park - Cave and Basin Area | Spring-fed marsh/meadow | 51,17100143 | -115,5879974 |
| 08BBLEP-04499 | 611 | JX008272 | Geometridae | Larentiinae | Xanthorhoe alticolata | Barnes & McDunnough | J.Straka,J.Cossey | 31-Jul-2008 | Canada | Alberta | Banff | Banff Nat. Park - Cave and Basin Area | Spring-fed marsh/meadow | 51,17100143 | -115,5879974 |
| 08BBLEP-04507 | 658 | JX008247 | Geometridae | Larentiinae | Xanthorhoe alticolata | Barnes & McDunnough | J.Straka,J.Cossey | 31-Jul-2008 | Canada | Alberta | Banff | Banff Nat. Park - Cave and Basin Area | Spring-fed marsh/meadow | 51,17100143 | -115,5879974 |
| 08BBLEP-04510 | 637 | JX008129 | Geometridae | Larentiinae | Xanthorhoe alticolata | Barnes & McDunnough | J.Straka,J.Cossey | 31-Jul-2008 | Canada | Alberta | Banff | Banff Nat. Park - Cave and Basin Area | Spring-fed marsh/meadow | 51,17100143 | -115,5879974 |
| 08BBLEP-04513 | 658 | JX008281 | Geometridae | Larentiinae | Xanthorhoe alticolata | Barnes & McDunnough | J.Straka,J.Cossey | 31-Jul-2008 | Canada | Alberta | Banff | Banff Nat. Park - Cave and Basin Area | Spring-fed marsh/meadow | 51,17100143 | -115,5879974 |
| 08BBLEP-04518 | 658 | JX008202 | Geometridae | Larentiinae | Xanthorhoe alticolata | Barnes & McDunnough | J.Straka,J.Cossey | 31-Jul-2008 | Canada | Alberta | Banff | Banff Nat. Park - Cave and Basin Area | Spring-fed marsh/meadow | 51,17100143 | -115,5879974 |
| 08BBLEP-04532 | 658 | JX008115 | Geometridae | Larentiinae | Xanthorhoe alticolata | Barnes & McDunnough | J.Straka,J.Cossey | 31-Jul-2008 | Canada | Alberta | Banff | Banff Nat. Park - Cave and Basin Area | Spring-fed marsh/meadow | 51,17100143 | -115,5879974 |
| 08BBLEP-04896 | 658 | JX008386 | Geometridae | Larentiinae | Xanthorhoe alticolata | Barnes & McDunnough | | 06-Aug-2008 | Canada | Alberta | Waterton | Waterton Lakes Nat. Park - Blakiston Creek | Rocky creekside | 49,07799911 | -113,8820038 |
| 10BBCLP-0953 | 658 | JF841966 | Geometridae | Larentiinae | Xanthorhoe alticolata | Barnes & McDunnough | BIObus 2010 | 20-Jul-2010 | Canada | British Columbia | Yoho NP | Kicking Horse Cmpgrd | Forested cmpgrd | 51,42399979 | -116,4290009 |
| 10BBCLP-0954 | 658 | JF841967 | Geometridae | Larentiinae | Xanthorhoe alticolata | Barnes & McDunnough | BIObus 2010 | 23-Jul-2010 | Canada | British Columbia | Yoho NP | Lake O`Hara Parking area | Side of gravel road | 51,4469986 | -116,3239975 |
| BC ZSM Lep 49268 | 658 | JN274137 | Geometridae | Larentiinae | Xanthorhoe annotinata | Zettersted | L. Weigert | 01-Jul-2008 | Sweden | | Torne Lappmark | | 8 km NE Jukkasjaerv | 67,88890076 | 20,75639915 |
| MM00603 | 611 | HM871086 | Geometridae | Larentiinae | Xanthorhoe annotinata | Zetterstedt | Marko Mutanen | 08-Jun-2006 | Finland | | Ostrobottnia borealis pars australis | | Kiiminki | 65,07099915 | 25,72500038 |
| MM04112 | 658 | HM386902 | Geometridae | Larentiinae | Xanthorhoe annotinata | Zettersted | Marko Mutanen | | Finland | | | | Enontekioe | 68,99700165 | 20,74399948 |
| MM14036 | 658 | HM875852 | Geometridae | Larentiinae | Xanthorhoe annotinata | Zettersted | Marko Mutanen | | Finland | | Karelia borealis | | Tuupovaara | 62,375 | 30,90500069 |
| 10BBCLP-1060 | 658 | JF842073 | Geometridae | Larentiinae | Xanthorhoe baffinensis | McDunnough | BIObus 2010 | 25-Jun-2010 | Canada | Alberta | Jasper NP | Cottonwood Slough | Slough | 52,89199829 | -118,0910034 |
| 10BBCLP-1062 | 658 | JF842075 | Geometridae | Larentiinae | Xanthorhoe baffinensis | McDunnough | BIObus 2010 | 23-Jul-2010 | Canada | British Columbia | Yoho NP | Takakkaw Fall | Forested trail in river basin | 51,5 | -116,4729996 |
| CNCLEP00034159 | 658 | JX008211 | Geometridae | Larentiinae | Xanthorhoe baffinensis | McDunnough | H. Hense | 20-Jul-1996 | United States | Nevada | vic. Arviat | | | | |
| CNCLEP00034160 | 658 | JX008267 | Geometridae | Larentiinae | Xanthorhoe baffinensis | McDunnough | H. Hense | 18-Jul-1996 | United States | Nevada | vic. Arviat | | | | |
| 06-PROBE-2653 | 651 | JX008196 | Geometridae | Larentiinae | Xanthorhoe decoloraria | Esper | P.D.N. Hebert | 14-Aug-2006 | Canada | Manitoba | Churchill | 22 km ESE Churchill, vicinity of CNSC, Twin Lakes Roa | | 58,72499847 | -93,84100342 |
| 07PROBE-00551 | 658 | JX008123 | Geometridae | Larentiinae | Xanthorhoe decoloraria | Esper | J.deWaard | 13-Jul-2007 | Canada | Manitoba | Churchill | Town of Churchill | Aurora Inn | 58,77099991 | -94,1740036 |
| 07PROBE-03793 | 658 | JX008497 | Geometridae | Larentiinae | Xanthorhoe decoloraria | Esper | J.McGowan | 17-Jul-2007 | Canada | Manitoba | Churchill | Town of Churchill | Apartment in town | 58,77000046 | -94,16899872 |
| 07PROBE-03846 | 657 | JX008188 | Geometridae | Larentiinae | Xanthorhoe decoloraria | Esper | J.deWaard | 17-Jul-2007 | Canada | Manitoba | Churchill | 26 km SE Churchill, Twin Lake | Clearing near | 58,61800003 | -93,80999756 |
| 07PROBE-10020 | 658 | JX008175 | Geometridae | Larentiinae | Xanthorhoe decoloraria | Esper | P.D.N. Hebert | 18-Aug-2007 | Canada | Manitoba | Churchill | Town of Churchill | | 58,76900101 | -94,17199707 |
| 07PROBE-10168 | 658 | JX008453 | Geometridae | Larentiinae | Xanthorhoe decoloraria | Esper | P.D.N. Hebert | 19-Jul-2007 | Canada | Manitoba | Churchill | 26 km SE Churchill, Twin Lakes fen | | 58,63199997 | -93,78800201 |
| 07PROBE-10169 | 658 | JX008496 | Geometridae | Larentiinae | Xanthorhoe decoloraria | Esper | P.D.N. Hebert | 19-Jul-2007 | Canada | Manitoba | Churchill | 26 km SE Churchill, Twin Lakes fen | | 58,63199997 | -93,78800201 |
| 07PROBE-10239 | 645 | JX008235 | Geometridae | Larentiinae | Xanthorhoe decoloraria | Esper | P.D.N. Hebert | 21-Jul-2007 | Canada | Manitoba | Churchill | Town of Churchill | | 58,76900101 | -94,17199707 |
| 07PROBE-10320 | 658 | JX008425 | Geometridae | Larentiinae | Xanthorhoe decoloraria | Esper | P.D.N. Hebert | 22-Jul-2007 | Canada | Manitoba | Churchill | unspecified locality | (general coordinate) | 58,74000168 | -93,81999969 |
| 07PROBE-10321 | 658 | JX008413 | Geometridae | Larentiinae | Xanthorhoe decoloraria | Esper | P.D.N. Hebert | 22-Jul-2007 | Canada | Manitoba | Churchill | unspecified locality | (general coordinate) | 58,74000168 | -93,81999969 |
| 07PROBE-10322 | 658 | JX008439 | Geometridae | Larentiinae | Xanthorhoe decoloraria | Esper | P.D.N. Hebert | 22-Jul-2007 | Canada | Manitoba | Churchill | unspecified locality | (general coordinate) | 58,74000168 | -93,81999969 |
| 07PROBE-10323 | 658 | JX008132 | Geometridae | Larentiinae | Xanthorhoe decoloraria | Esper | P.D.N. Hebert | 22-Jul-2007 | Canada | Manitoba | Churchill | unspecified locality | (general coordinate) | 58,74000168 | -93,81999969 |
| 07PROBE-10402 | 658 | JX008245 | Geometridae | Larentiinae | Xanthorhoe decoloraria | Esper | P.D.N. Hebert | 22-Jul-2007 | Canada | Manitoba | Churchill | 10 km E Churchill, Launch Road | | 58,75999832 | -94 |
| 07PROBE-10404 | 658 | JX008204 | Geometridae | Larentiinae | Xanthorhoe decoloraria | Esper | P.D.N. Hebert | 22-Jul-2007 | Canada | Manitoba | Churchill | 10 km E Churchill, Launch Road | | 58,75999832 | -94 |
| 07PROBE-10405 | 657 | JX008121 | Geometridae | Larentiinae | Xanthorhoe decoloraria | Esper | P.D.N. Hebert | 22-Jul-2007 | Canada | Manitoba | Churchill | 10 km E Churchill, Launch Road | | 58,75999832 | -94 |
| 07PROBE-10443 | 650 | JX008411 | Geometridae | Larentiinae | Xanthorhoe decoloraria | Esper | P.D.N. Hebert | 25-Jul-2007 | Canada | Manitoba | Churchill | 2 km NW Churchill, Churchill Harbour | | 58,78099823 | -94,1969986 |
| 07PROBE-10634 | 658 | JX008367 | Geometridae | Larentiinae | Xanthorhoe decoloraria | Esper | P.D.N. Hebert | 20-Jul-2007 | Canada | Manitoba | Churchill | unspecified locality | (general coordinate) | 58,74000168 | -93,81999969 |
| 07PROBE-10636 | 658 | JX008414 | Geometridae | Larentiinae | Xanthorhoe decoloraria | Esper | P.D.N. Hebert | 20-Jul-2007 | Canada | Manitoba | Churchill | unspecified locality | (general coordinate) | 58,74000168 | -93,81999969 |
| 07PROBE-10694 | 658 | JX008424 | Geometridae | Larentiinae | Xanthorhoe decoloraria | Esper | P.D.N. Hebert | | Canada | Manitoba | Churchill | unspecified locality | (general coordinate) | 58,74000168 | -93,81999969 |
| 07PROBE-10917 | 658 | JX008302 | Geometridae | Larentiinae | Xanthorhoe decoloraria | Esper | P.D.N. Hebert | 23-Jul-2007 | Canada | Manitoba | Churchill | unspecified locality | (general coordinate) | 58,74000168 | -93,81999969 |
| 07PROBE-10918 | 658 | JX008350 | Geometridae | Larentiinae | Xanthorhoe decoloraria | Esper | P.D.N. Hebert | 23-Jul-2007 | Canada | Manitoba | Churchill | unspecified locality | (general coordinate) | 58,74000168 | -93,81999969 |
| 07PROBE-10919 | 658 | JX008206 | Geometridae | Larentiinae | Xanthorhoe decoloraria | Esper | P.D.N. Hebert | 23-Jul-2007 | Canada | Manitoba | Churchill | unspecified locality | (general coordinate) | 58,74000168 | -93,81999969 |
| 07WNP-10382 | 658 | JX008192 | Geometridae | Larentiinae | Xanthorhoe decoloraria | Esper | J.McGowan | 04-Aug-2007 | Canada | Manitoba | Churchill | Town of Churchill | 111 Hearne St., backyard | 58,76900101 | -94,15799713 |
| 07WNP-10391 | 658 | JX008178 | Geometridae | Larentiinae | Xanthorhoe decoloraria | Esper | J.McGowan | 04-Aug-2007 | Canada | Manitoba | Churchill | Town of Churchill | 111 Hearne St., backyard | 58,76900101 | -94,15799713 |
| 07WNP-10750 | 658 | JX008264 | Geometridae | Larentiinae | Xanthorhoe decoloraria | Esper | J.McGowan | 11-Aug-2007 | Canada | Manitoba | Churchill | Town of Churchill | 111 Hearne St., backyard | 58,76900101 | -94,15799713 |
| 07WNP-10751 | 658 | JX008374 | Geometridae | Larentiinae | Xanthorhoe decoloraria | Esper | J.McGowan | 11-Aug-2007 | Canada | Manitoba | Churchill | Town of Churchill | 111 Hearne St., backyard | 58,76900101 | -94,15799713 |
| 08BBLEP-04501 | 584 | JX008286 | Geometridae | Larentiinae | Xanthorhoe decoloraria | Esper | J.Straka,J.Cossey | 31-Jul-2008 | Canada | Alberta | Banff | Banff Nat. Park - Cave and Basin Area | Spring-fed marsh/meadow | 51,17100143 | -115,5879974 |
| 08BBLEP-04504 | 613 | JX008149 | Geometridae | Larentiinae | Xanthorhoe decoloraria | Esper | J.Straka,J.Cossey | 31-Jul-2008 | Canada | Alberta | Banff | Banff Nat. Park - Cave and Basin Area | Spring-fed marsh/meadow | 51,17100143 | -115,5879974 |
| 08BBLEP-04505 | 658 | JX008478 | Geometridae | Larentiinae | Xanthorhoe decoloraria | Esper | J.Straka,J.Cossey | 31-Jul-2008 | Canada | Alberta | Banff | Banff Nat. Park - Cave and Basin Area | Spring-fed marsh/meadow | 51,17100143 | -115,5879974 |

| | | | | | | | | | | | | | | | |
|---|---|---|---|---|---|---|---|---|---|---|---|---|---|---|---|
| 08BBLEP-04572 | 658 | JX008306 | Geometridae | Larentiinae | Xanthorhoe decoloraria | Esper | J.Straka,J.Cossey | 01-Aug-2008 | Canada | Alberta | Banff | Banff Nat. Park - Vermillion Lakes | Aspen/conifer stand, marsh border | 51,17599869 | -115,6350021 |
| 08BBLEP-04607 | 658 | JX008470 | Geometridae | Larentiinae | Xanthorhoe decoloraria | Esper | J.Straka,J.Cossey | 01-Aug-2008 | Canada | Alberta | Banff | Banff Nat. Park - Vermillion Lakes | Aspen/conifer stand, marsh border | 51,17599869 | -115,6350021 |
| 10BBCLP-0950 | 658 | JF841963 | Geometridae | Larentiinae | Xanthorhoe decoloraria | Esper | BIObus 2010 | 10-Aug-2010 | Canada | Alberta | Elk Island NP | Beaver Pond Trl | Aspen forest: sedge meadow | 53,68700027 | -112,8130035 |
| 10BBCLP-0960 | 658 | JF841973 | Geometridae | Larentiinae | Xanthorhoe decoloraria | Esper | BIObus 2010 | 02-Aug-2010 | Canada | Alberta | Jasper NP | Whisters Cmpgrd. | Pine forest | 52,8429985 | -118,0719986 |
| 10BBCLP-0966 | 658 | JF841979 | Geometridae | Larentiinae | Xanthorhoe decoloraria | Esper | BIObus 2010 | 03-Aug-2010 | Canada | Alberta | Jasper NP | Five Lakes Valley Tr | Mixed fores | 52,80799866 | -118,0230026 |
| 10BBCLP-1176 | 658 | JF842189 | Geometridae | Larentiinae | Xanthorhoe decoloraria | Esper | BIObus 2010 | 09-Aug-2010 | Canada | Alberta | Elk Island NP | Tawayik Lake Picnic Area | Meadow: aspen fores | 53,61800003 | -112,875 |
| 10-JDWBC-0180 | 658 | HM863507 | Geometridae | Larentiinae | Xanthorhoe decoloraria | Esper | deWaard, J.R. | 12-Jul-2009 | Canada | British Columbia | nr. Hazelton | Date Creek Silvicultural System | A4-T | 55,46500015 | -127,8059998 |
| 10-JDWBC-0886 | 658 | HM870322 | Geometridae | Larentiinae | Xanthorhoe decoloraria | Esper | deWaard, J.R. | 12-Jul-2009 | Canada | British Columbia | nr. Hazelton | Date Creek Silvicultural System | B2-T | 55,45899963 | -127,8119965 |
| 10-JDWBC-2400 | 658 | HM864164 | Geometridae | Larentiinae | Xanthorhoe decoloraria | Esper | deWaard, J.R. | 12-Jul-2009 | Canada | British Columbia | nr. Hazelton | Date Cr. Silvicultural Systen | A3-T | 55,4640007 | -127,8099976 |
| BC NP 0205 | 407 | JX034673 | Geometridae | Larentiinae | Xanthorhoe decoloraria | Esper | N. Poel | 09-Aug-2008 | Austria | Steiermark | Totes Gebirge | | Puehringerhuette | 47,68700027 | 13,96700001 |
| BC ZSM Lep 34365 | 658 | HQ957823 | Geometridae | Larentiinae | Xanthorhoe decoloraria | Esper | P. Skou | 25-Jul-2006 | Italy | Piedmont | | | Colle di Lombarda | 44,19919968 | 7,149000168 |
| BC ZSM Lep 34925 | 658 | HM914350 | Geometridae | Larentiinae | Xanthorhoe decoloraria | Esper | B. Schacht | 22-Jul-2009 | Russia | | Altai | | env. Aktasch | 50,31159973 | 87,61450195 |
| MM02857 | 658 | HM871730 | Geometridae | Larentiinae | Xanthorhoe decoloraria | Esper | Marko Mutanen | | Finland | | Ostrobottnia borealis pars australis | | Kiiminki | 65,07099915 | 25,72500038 |
| MM04106 | 658 | HM386896 | Geometridae | Larentiinae | Xanthorhoe decoloraria | Esper | Marko Mutanen | | Finland | | | | Enontekioe | 68,99700165 | 20,74399948 |
| MM06361 | 658 | HQ570327 | Geometridae | Larentiinae | Xanthorhoe decoloraria | Esper | Marko Mutanen, Anttoni Mutanen, Nestori Mutanen | 29-Jun-2007 | Finland | | Lapponia inarensis | | Utsjoki | 69,37599945 | 25,80500031 |
| TLMF Lep 00377 | 658 | HM425865 | Geometridae | Larentiinae | Xanthorhoe decoloraria | Esper | Huemer P. | 31-Jul-2009 | Austria | Karnten | | | Mallnitz, Doesental, Arthur-v.-Schmid-Haus W | 46,98939896 | 13,26690006 |
| TLMF Lep 00474 | 658 | HM425949 | Geometridae | Larentiinae | Xanthorhoe decoloraria | Esper | Huemer P. | 27-Jul-2009 | Italy | | Cuneo | | Colle Valcavera NE/ Demonte NW | 44,38439941 | 7,106389999 |
| TLMF Lep 00725 | 658 | HM426085 | Geometridae | Larentiinae | Xanthorhoe decoloraria | Esper | Tarmann G. M | 22-Jul-2009 | Italy | | Belluno | | Passo di Valparola W, Fort SW | 46,52669907 | 11,9921999 |
| CNCLEP00034063 | 307 | JX008481 | Geometridae | Larentiinae | Xanthorhoe incursata | Hübner | G.P. Holland | 02-Jul-1948 | Canada | British Columbia | Mt. Revelstoke | | | | |
| CNCLEP00034064 | 307 | JX008336 | Geometridae | Larentiinae | Xanthorhoe incursata | Hübner | G.P. Holland | 08-Jul-1948 | Canada | British Columbia | Eva Lake trai | | | | |
| TLMF Lep 01144 | 658 | HQ968504 | Geometridae | Larentiinae | Xanthorhoe incursata | Hübner | Aistleitner U. | 13-Jun-2009 | Switzerland | Graubunden | | | Tiefencastel S/ Salouf, Got Grond | 46,65000153 | 9,597000122 |
| TLMF Lep 01145 | 658 | HQ968505 | Geometridae | Larentiinae | Xanthorhoe incursata | Hübner | Aistleitner U. | 13-Jun-2009 | Switzerland | Graubunden | | | Tiefencastel S/ Salouf, Got Grond | 46,65000153 | 9,597000122 |
| TLMF Lep 02554 | 658 | JF860091 | Geometridae | Larentiinae | Xanthorhoe incursata | Hübner | Huemer P | 09-Jun-2010 | Italy | South Tyro | | | Ritten/ Obergruenwal | 46,59700012 | 11,43900013 |
| MM08067 | 658 | HM873764 | Noctuidae | Noctuinae | Xestia alpicola | Zetterstedt | Marko Mutanen, Panu Vaelimaeki | | Finland | | Lapponia kemensis pars occidentalis | | Kolari | 67,27600098 | 23,75300026 |
| MM08068 | 658 | HM873765 | Noctuidae | Noctuinae | Xestia alpicola | Zetterstedt | Marko Mutanen, Panu Vaelimaeki | | Finland | | Lapponia kemensis pars occidentalis | | Kolari | 67,27600098 | 23,75300026 |
| MM12102 | 658 | HM875192 | Noctuidae | Noctuinae | Xestia alpicola | Zetterstedt | Marko Mutanen, Panu Vaelimaek | | Finland | | Nylandia | | Hanko | 59,83599854 | 23,23600006 |
| MM18749 | 658 | JF854600 | Noctuidae | Noctuinae | Xestia alpicola | Zetterstedt | Reima Leinonen | 29-Jun-2010 | Finland | | Ostrobottnia kajanensi | | Puolanka | 64,66000366 | 28,04700089 |
| TLMF Lep 00366 | 658 | HM425856 | Noctuidae | Noctuinae | Xestia alpicola | Zetterstedt | Huemer P. | 01-Aug-2009 | Austria | Karnten | | | Mallnitz, Doesental, Arthur-v.-Schmid-Haus W | 46,98939896 | 13,26690006 |
| TLMF Lep 00727 | 658 | HM426087 | Noctuidae | Noctuinae | Xestia alpicola | Zetterstedt | Tarmann G. M | 22-Jul-2009 | Italy | | Belluno | | Passo di Valparola W, Fort SW | 46,52669907 | 11,9921999 |
| TLMF Lep 00728 | 658 | HM426088 | Noctuidae | Noctuinae | Xestia alpicola | Zetterstedt | Tarmann G. M | 22-Jul-2009 | Italy | | Belluno | | Passo di Valparola W, Fort SW | 46,52669907 | 11,9921999 |
| TLMF Lep 02768 | 658 | JF860284 | Noctuidae | Noctuinae | Xestia alpicola | Zetterstedt | Schmid J. | 19-Jul-2007 | Switzerland | Graubunden | | | NW Abhang Horn/ Vals | 46,61000061 | 9,194999695 |
| TLMF Lep 02898 | 658 | JF860349 | Noctuidae | Noctuinae | Xestia alpicola | Zetterstedt | Huemer P. | 31-Jul-2009 | Austria | Karnten | | | Mallnitz, Doesental, Arthur-v.-Schmid-Haus W | 46,98899841 | 13,2670002 |
| MM06114 | 560 | HM873049 | Noctuidae | Noctuinae | Xestia fennica | Brandt | Panu Vaelimaek | | Finland | | Regio kuusamoensis | | Kuusamo | 66,22299957 | 29,42200089 |
| MM18661 | 428 | JX034622 | Noctuidae | Noctuinae | Xestia fennica | Brandt | Panu Vaelimaeki, Petri Hirvonen | | Finland | | Lapponia kemensis pars occidentalis | | Muonio | 68,01000214 | 24,12899971 |
| CNC LEP00052808 | 657 | JX008472 | Noctuidae | Noctuinae | Xestia lorez | Staudinger | T. Kva | 18-Jul-1988 | United States | Alaska | | | Hazchor Pass, near Palme | | |
| CNC LEP00052809 | 642 | JX008404 | Noctuidae | Noctuinae | Xestia lorez | Staudinger | | 29-Jul-1979 | Canada | Yukon Territory | | | km 84 of Dempster Hwy | | |
| MM05675 | 658 | HQ570319 | Noctuidae | Noctuinae | Xestia lorez | Staudinger | Petri Hirvonen | 01-Jul-2007 | Finland | | Lapponia enontekiensis | | Enontekioe | 68,99700165 | 20,74399948 |
| MM15896 | 658 | HQ570424 | Noctuidae | Noctuinae | Xestia lorez | Staudinger | Marko Mutanen | | Finland | | Lapponia enontekiensis | | Enontekioe | 68,99700165 | 20,74399948 |
| MM15897 | 658 | HQ570425 | Noctuidae | Noctuinae | Xestia lorez | Staudinger | Marko Mutanen | | Finland | | Lapponia enontekiensis | | Enontekioe | 68,99700165 | 20,74399948 |
| NOC14148 | 658 | JX008346 | Noctuidae | Noctuinae | Xestia lorez | Staudinger | Wood & Lafontaine | 20-Jul-1981 | Canada | Yukon Territory | Km. 82 Dempster Hwy | | | 64,33000183 | -138,1399994 |
| TLMF Lep 00747 | 658 | HM426104 | Noctuidae | Noctuinae | Xestia lorez | Staudinger | Wieser C. | 15-Jul-2009 | Austria | Karnten | | | Kleiner Trieb E | 47 | 13,06669998 |
| TLMF Lep 00167 | 331 | JX034590 | Noctuidae | Noctuinae | Xestia rhaetica | Staudinger | Huemer P. & Erlebach S | 25-Aug-2005 | Austria | Salzburg | | Wildgerlostal, S Trisslalm- Krimm | | 47,18999863 | 12,11299992 |
| TLMF Lep 00168 | 331 | JX034680 | Noctuidae | Noctuinae | Xestia rhaetica | Staudinger | Huemer P. & Erlebach S | 25-Aug-2005 | Austria | Salzburg | | Wildgerlostal, S Trisslalm- Krimm | | 47,18999863 | 12,11299992 |
| TLMF Lep 00174 | 331 | JX034682 | Noctuidae | Noctuinae | Xestia rhaetica | Staudinger | Huemer P. | 23-Jul-2007 | Italy | South Tyrol | | Schlern, Touristensteig, Latschenfelder- Kastelrut | | 46,51200104 | 11,5909996 |
| TLMF Lep 00175 | 331 | JX034585 | Noctuidae | Noctuinae | Xestia rhaetica | Staudinger | Huemer P. | 23-Jul-2007 | Italy | South Tyrol | | Schlern, Touristensteig, Latschenfelder- Kastelrut | | 46,51200104 | 11,5909996 |
| 07PROBE-10458 | 658 | JX008199 | Noctuidae | Noctuinae | Xestia speciosa | Hübner | P.D.N. Hebert | 25-Jul-2007 | Canada | Manitoba | Churchill | 2 km NW Churchill, Churchill Harbour | | 58,78099823 | -94,1969986 |
| 07PROBE-10622 | 658 | JX008433 | Noctuidae | Noctuinae | Xestia speciosa | Hübner | P.D.N. Hebert | 20-Jul-2007 | Canada | Manitoba | Churchill | unspecified locality | (general coordinate) | 58,74000168 | -93,81999969 |
| 07WNP-10367 | 657 | JX008128 | Noctuidae | Noctuinae | Xestia speciosa | Hübner | J.McGowan | 04-Aug-2007 | Canada | Manitoba | Churchill | Town of Churchill | 111 Hearne St., backyard | 58,76900101 | -94,15799713 |
| 07WNP-10592 | 658 | JX008482 | Noctuidae | Noctuinae | Xestia speciosa | Hübner | J.McGowan | 10-Aug-2007 | Canada | Manitoba | Churchill | Town of Churchill | Cemetery | 58,76900101 | -94,15599823 |
| 07WNP-10593 | 658 | JX008468 | Noctuidae | Noctuinae | Xestia speciosa | Hübner | J.McGowan | 10-Aug-2007 | Canada | Manitoba | Churchill | Town of Churchill | Cemetery | 58,76900101 | -94,15599823 |
| 08BBLEP-04416 | 658 | JX008369 | Noctuidae | Noctuinae | Xestia speciosa | Hübner | J.Straka | 02-Aug-2008 | Canada | Alberta | Banff | Banff Nat. Park - Tunnel Mountain Campground | Pine forest near aspen stand | 51,19300079 | -115,5339966 |
| 08BBLEP-04498 | 658 | JX008212 | Noctuidae | Noctuinae | Xestia speciosa | Hübner | J.Straka,J.Cossey | 31-Jul-2008 | Canada | Alberta | Banff | Banff Nat. Park - Cave and Basin Area | Spring-fed marsh/meadow | 51,17100143 | -115,5879974 |
| 08BBLEP-04611 | 658 | JX008151 | Noctuidae | Noctuinae | Xestia speciosa | Hübner | J.Straka,J.Cossey | 01-Aug-2008 | Canada | Alberta | Banff | Banff Nat. Park - Vermillion Lakes | Aspen/conifer stand, marsh border | 51,17599869 | -115,6350021 |
| 08-JDWBC-0733 | 658 | JX008228 | Noctuidae | Noctuinae | Xestia speciosa | Hübner | deWaard, J.R. | 07-Aug-2008 | Canada | British Columbia | nr. Houston | MOFR MPB site | 138T | 54,66600037 | -126,3570023 |
| 08-JDWBC-0736 | 658 | JX008287 | Noctuidae | Noctuinae | Xestia speciosa | Hübner | deWaard, J.R. | 07-Aug-2008 | Canada | British Columbia | nr. Houston | MOFR MPB site | 138T | 54,66600037 | -126,3570023 |
| 08-JDWBC-0761 | 658 | JX008203 | Noctuidae | Noctuinae | Xestia speciosa | Hübner | deWaard, J.R. | 07-Aug-2008 | Canada | British Columbia | nr. Houston | MOFR MPB site | 138T | 54,66600037 | -126,3570023 |
| 08-JDWBC-0953 | 658 | JX008125 | Noctuidae | Noctuinae | Xestia speciosa | Hübner | deWaard, J.R. | 07-Aug-2008 | Canada | British Columbia | nr. Houston | MOFR MPB site | 280T | 54,63299942 | -126,4240036 |
| 08-JDWBC-0954 | 658 | JX008375 | Noctuidae | Noctuinae | Xestia speciosa | Hübner | deWaard, J.R. | 07-Aug-2008 | Canada | British Columbia | nr. Houston | MOFR MPB site | 280T | 54,63299942 | -126,4240036 |
| 08-JDWBC-0960 | 658 | JX008328 | Noctuidae | Noctuinae | Xestia speciosa | Hübner | deWaard, J.R. | 07-Aug-2008 | Canada | British Columbia | nr. Houston | MOFR MPB site | 280T | 54,63299942 | -126,4240036 |
| 08-JDWBC-0965 | 658 | JX008142 | Noctuidae | Noctuinae | Xestia speciosa | Hübner | deWaard, J.R. | 07-Aug-2008 | Canada | British Columbia | nr. Houston | MOFR MPB site | 280T | 54,63299942 | -126,4240036 |
| 08-JDWBC-0966 | 658 | JX008220 | Noctuidae | Noctuinae | Xestia speciosa | Hübner | deWaard, J.R. | 07-Aug-2008 | Canada | British Columbia | nr. Houston | MOFR MPB site | 280T | 54,63299942 | -126,4240036 |
| 08-JDWBC-0968 | 658 | JX008490 | Noctuidae | Noctuinae | Xestia speciosa | Hübner | deWaard, J.R. | 07-Aug-2008 | Canada | British Columbia | nr. Houston | MOFR MPB site | 280T | 54,63299942 | -126,4240036 |
| 08-JDWBC-0969 | 658 | JX008447 | Noctuidae | Noctuinae | Xestia speciosa | Hübner | deWaard, J.R. | 07-Aug-2008 | Canada | British Columbia | nr. Houston | MOFR MPB site | 280T | 54,63299942 | -126,4240036 |
| 08-JDWBC-0996 | 658 | JX008282 | Noctuidae | Noctuinae | Xestia speciosa | Hübner | deWaard, J.R. | 07-Aug-2008 | Canada | British Columbia | nr. Houston | MOFR MPB site | 280T | 54,63299942 | -126,4240036 |
| 08-JDWBC-0997 | 658 | JX008450 | Noctuidae | Noctuinae | Xestia speciosa | Hübner | deWaard, J.R. | 07-Aug-2008 | Canada | British Columbia | nr. Houston | MOFR MPB site | 280T | 54,63299942 | -126,4240036 |
| 08-JDWBC-0999 | 658 | JX008415 | Noctuidae | Noctuinae | Xestia speciosa | Hübner | deWaard, J.R. | 07-Aug-2008 | Canada | British Columbia | nr. Houston | MOFR MPB site | 280T | 54,63299942 | -126,4240036 |
| 08-JDWBC-1001 | 658 | JX008111 | Noctuidae | Noctuinae | Xestia speciosa | Hübner | deWaard, J.R. | 07-Aug-2008 | Canada | British Columbia | nr. Houston | MOFR MPB site | 280T | 54,63299942 | -126,4240036 |
| 08-JDWBC-1002 | 658 | JX008315 | Noctuidae | Noctuinae | Xestia speciosa | Hübner | deWaard, J.R. | 07-Aug-2008 | Canada | British Columbia | nr. Houston | MOFR MPB site | 280T | 54,63299942 | -126,4240036 |
| 08-JDWBC-1015 | 658 | JX008385 | Noctuidae | Noctuinae | Xestia speciosa | Hübner | deWaard, J.R. | 07-Aug-2008 | Canada | British Columbia | nr. Houston | MOFR MPB site | 280T | 54,63299942 | -126,4240036 |
| 08-JDWBC-1019 | 658 | JX008493 | Noctuidae | Noctuinae | Xestia speciosa | Hübner | deWaard, J.R. | 07-Aug-2008 | Canada | British Columbia | nr. Houston | MOFR MPB site | 280T | 54,63299942 | -126,4240036 |

| | | | | | | | | | | | | | | |
|---|---|---|---|---|---|---|---|---|---|---|---|---|---|---|
| 08-JDWBC-1857 | 658 | JX008324 | Noctuidae | Noctuinae | Xestia speciosa | Hübner | deWaard, J.R. | 07-Aug-2008 | Canada | British Columbia | nr. Houston | MOFR MPB site | 93T | 54,61700058 | -126,4110031 |
| 08-JDWBC-1863 | 658 | JX008273 | Noctuidae | Noctuinae | Xestia speciosa | Hübner | deWaard, J.R. | 07-Aug-2008 | Canada | British Columbia | nr. Houston | MOFR MPB site | 93T | 54,61700058 | -126,4110031 |
| 08-JDWBC-1867 | 658 | JX008339 | Noctuidae | Noctuinae | Xestia speciosa | Hübner | deWaard, J.R. | 07-Aug-2008 | Canada | British Columbia | nr. Houston | MOFR MPB site | 93T | 54,61700058 | -126,4110031 |
| 08-JDWBC-1868 | 658 | JX008335 | Noctuidae | Noctuinae | Xestia speciosa | Hübner | deWaard, J.R. | 07-Aug-2008 | Canada | British Columbia | nr. Houston | MOFR MPB site | 93T | 54,61700058 | -126,4110031 |
| 08-JDWBC-1870 | 658 | JX008327 | Noctuidae | Noctuinae | Xestia speciosa | Hübner | deWaard, J.R. | 07-Aug-2008 | Canada | British Columbia | nr. Houston | MOFR MPB site | 93T | 54,61700058 | -126,4110031 |
| 08-JDWBC-1872 | 658 | JX008297 | Noctuidae | Noctuinae | Xestia speciosa | Hübner | deWaard, J.R. | 07-Aug-2008 | Canada | British Columbia | nr. Houston | MOFR MPB site | 93T | 54,61700058 | -126,4110031 |
| 08-JDWBC-1873 | 658 | JX008438 | Noctuidae | Noctuinae | Xestia speciosa | Hübner | deWaard, J.R. | 07-Aug-2008 | Canada | British Columbia | nr. Houston | MOFR MPB site | 93T | 54,61700058 | -126,4110031 |
| 08-JDWBC-1876 | 658 | JX008213 | Noctuidae | Noctuinae | Xestia speciosa | Hübner | deWaard, J.R. | 07-Aug-2008 | Canada | British Columbia | nr. Houston | MOFR MPB site | 93T | 54,61700058 | -126,4110031 |
| 08-JDWBC-1877 | 658 | JX008489 | Noctuidae | Noctuinae | Xestia speciosa | Hübner | deWaard, J.R. | 07-Aug-2008 | Canada | British Columbia | nr. Houston | MOFR MPB site | 93T | 54,61700058 | -126,4110031 |
| 08-JDWBC-2314 | 658 | JX008420 | Noctuidae | Noctuinae | Xestia speciosa | Hübner | deWaard, J.R. | 06-Aug-2008 | Canada | British Columbia | nr. Hazelton | Date Cr. Silvicultural System | A3-T2 | 55,4640007 | -127,8099976 |
| 10BBCLP-1650 | 658 | JF842644 | Noctuidae | Noctuinae | Xestia speciosa | Hübner | BIObus 2010 | 23-Jul-2010 | Canada | British Columbia | Yoho NP | Lake O`Hara Parking area | Side of gravel road | 51,4469986 | -116,3239975 |
| 10BBCLP-1651 | 658 | JF842645 | Noctuidae | Noctuinae | Xestia speciosa | Hübner | BIObus 2010 | 23-Jul-2010 | Canada | British Columbia | Yoho NP | Lake O`Hara Parking area | Side of gravel road | 51,4469986 | -116,3239975 |
| 10BBCLP-1652 | 658 | JF842646 | Noctuidae | Noctuinae | Xestia speciosa | Hübner | BIObus 2010 | 23-Jul-2010 | Canada | British Columbia | Yoho NP | Lake O`Hara Parking area | Side of gravel road | 51,4469986 | -116,3239975 |
| 10BBCLP-1653 | 658 | JF842647 | Noctuidae | Noctuinae | Xestia speciosa | Hübner | BIObus 2010 | 23-Jul-2010 | Canada | British Columbia | Yoho NP | Lake O`Hara Parking area | Side of gravel road | 51,4469986 | -116,3239975 |
| 10BBCLP-1654 | 658 | JF842648 | Noctuidae | Noctuinae | Xestia speciosa | Hübner | BIObus 2010 | 23-Jul-2010 | Canada | British Columbia | Yoho NP | Lake O`Hara Parking area | Side of gravel road | 51,4469986 | -116,3239975 |
| 10BBCLP-1655 | 658 | JF842649 | Noctuidae | Noctuinae | Xestia speciosa | Hübner | BIObus 2010 | 28-Jul-2010 | Canada | British Columbia | Kootenay NP | Olive Lake Trl. | Mixed forest around shallow lake | 50,67499924 | -115,9309998 |
| 10BBCLP-1656 | 658 | JF842650 | Noctuidae | Noctuinae | Xestia speciosa | Hübner | BIObus 2010 | 20-Jul-2010 | Canada | British Columbia | Yoho NP | Kicking Horse Cmpgrd | Forested cmpgrd | 51,42399979 | -116,4290009 |
| 10BBCLP-1657 | 658 | JF842651 | Noctuidae | Noctuinae | Xestia speciosa | Hübner | BIObus 2010 | 16-Jul-2010 | Canada | British Columbia | Glacier NP | Loop Trl. | Riverside fores | 51,25600052 | -117,538002 |
| 10BBCLP-1658 | 658 | JF842652 | Noctuidae | Noctuinae | Xestia speciosa | Hübner | BIObus 2010 | 16-Jul-2010 | Canada | British Columbia | Glacier NP | Loop Trl. | Riverside fores | 51,25600052 | -117,538002 |
| 10-JDWBC-0018 | 658 | HM862547 | Noctuidae | Noctuinae | Xestia speciosa | Hübner | deWaard, J.R. | 12-Jul-2009 | Canada | British Columbia | nr. Hazelton | Date Creek Silvicultural System | B1-T | 55,43700027 | -127,8010025 |
| 10-JDWBC-0109 | 658 | HM862728 | Noctuidae | Noctuinae | Xestia speciosa | Hübner | deWaard, J.R. | 12-Jul-2009 | Canada | British Columbia | nr. Hazelton | Date Creek Silvicultural System | A4-T | 55,46500015 | -127,8059998 |
| 10-JDWBC-0218 | 658 | HM863925 | Noctuidae | Noctuinae | Xestia speciosa | Hübner | deWaard, J.R. | 12-Jul-2009 | Canada | British Columbia | nr. Hazelton | Date Creek Silvicultural System | B5-T | 55,42699814 | -127,8030014 |
| 10-JDWBC-0347 | 625 | HM865336 | Noctuidae | Noctuinae | Xestia speciosa | Hübner | deWaard, J.R. | 12-Jul-2009 | Canada | British Columbia | nr. Hazelton | Date Creek Silvicultural System | B4-T | 55,43099976 | -127,8099976 |
| 10-JDWBC-0496 | 658 | HM866962 | Noctuidae | Noctuinae | Xestia speciosa | Hübner | deWaard, J.R. | 12-Jul-2009 | Canada | British Columbia | nr. Hazelton | Date Creek Silvicultural System | B3-T | 55,43899918 | -127,814003 |
| 10-JDWBC-0497 | 658 | HM866973 | Noctuidae | Noctuinae | Xestia speciosa | Hübner | deWaard, J.R. | 12-Jul-2009 | Canada | British Columbia | nr. Hazelton | Date Creek Silvicultural System | B3-T | 55,43899918 | -127,814003 |
| 10-JDWBC-0498 | 658 | HM866984 | Noctuidae | Noctuinae | Xestia speciosa | Hübner | deWaard, J.R. | 12-Jul-2009 | Canada | British Columbia | nr. Hazelton | Date Creek Silvicultural System | B3-T | 55,43899918 | -127,814003 |
| 10-JDWBC-0499 | 658 | HM866995 | Noctuidae | Noctuinae | Xestia speciosa | Hübner | deWaard, J.R. | 12-Jul-2009 | Canada | British Columbia | nr. Hazelton | Date Creek Silvicultural System | B3-T | 55,43899918 | -127,814003 |
| 10-JDWBC-0500 | 658 | HM867006 | Noctuidae | Noctuinae | Xestia speciosa | Hübner | deWaard, J.R. | 12-Jul-2009 | Canada | British Columbia | nr. Hazelton | Date Creek Silvicultural System | B3-T | 55,43899918 | -127,814003 |
| 10-JDWBC-0501 | 658 | HM867017 | Noctuidae | Noctuinae | Xestia speciosa | Hübner | deWaard, J.R. | 12-Jul-2009 | Canada | British Columbia | nr. Hazelton | Date Creek Silvicultural System | B3-T | 55,43899918 | -127,814003 |
| 10-JDWBC-0502 | 658 | HM867028 | Noctuidae | Noctuinae | Xestia speciosa | Hübner | deWaard, J.R. | 12-Jul-2009 | Canada | British Columbia | nr. Hazelton | Date Creek Silvicultural System | B3-T | 55,43899918 | -127,814003 |
| 10-JDWBC-0503 | 658 | HM867039 | Noctuidae | Noctuinae | Xestia speciosa | Hübner | deWaard, J.R. | 12-Jul-2009 | Canada | British Columbia | nr. Hazelton | Date Creek Silvicultural System | B3-T | 55,43899918 | -127,814003 |
| 10-JDWBC-0648 | 658 | HM868596 | Noctuidae | Noctuinae | Xestia speciosa | Hübner | deWaard, J.R. | 12-Jul-2009 | Canada | British Columbia | nr. Hazelton | Date Cr. Silvicultural System | A2-T | 55,4659996 | -127,8150024 |
| 10-JDWBC-0815 | 658 | HM870251 | Noctuidae | Noctuinae | Xestia speciosa | Hübner | deWaard, J.R. | 12-Jul-2009 | Canada | British Columbia | nr. Hazelton | Date Creek Silvicultural System | B2-T | 55,45899963 | -127,8119965 |
| 10-JDWBC-0915 | 658 | HM870351 | Noctuidae | Noctuinae | Xestia speciosa | Hübner | deWaard, J.R. | 30-Jul-2009 | Canada | British Columbia | nr. Hazelton | Date Creek Silvicultural System | B4-T | 55,43099976 | -127,8099976 |
| 10-JDWBC-0935 | 658 | HM870371 | Noctuidae | Noctuinae | Xestia speciosa | Hübner | deWaard, J.R. | 30-Jul-2009 | Canada | British Columbia | nr. Hazelton | Date Creek Silvicultural System | B4-T | 55,43099976 | -127,8099976 |
| 10-JDWBC-3087 | 658 | HM864916 | Noctuidae | Noctuinae | Xestia speciosa | Hübner | deWaard, J.R. | 30-Jul-2009 | Canada | British Columbia | nr. Hazelton | Date Cr. Silvicultural System | B5-T | 55,42699814 | -127,8030014 |
| 10-JDWBC-3089 | 658 | HM864918 | Noctuidae | Noctuinae | Xestia speciosa | Hübner | deWaard, J.R. | 30-Jul-2009 | Canada | British Columbia | nr. Hazelton | Date Cr. Silvicultural System | B5-T | 55,42699814 | -127,8030014 |
| 10-JDWBC-3466 | 658 | HM865331 | Noctuidae | Noctuinae | Xestia speciosa | Hübner | deWaard, J.R. | 30-Jul-2009 | Canada | British Columbia | nr. Hazelton | Date Creek Silvicultural System | B2-T | 55,45899963 | -127,8119965 |
| 10-JDWBC-3788 | 658 | HM865683 | Noctuidae | Noctuinae | Xestia speciosa | Hübner | deWaard, J.R. | 30-Jul-2009 | Canada | British Columbia | nr. Hazelton | Date Creek Silvicultural System | B3-T | 55,43899918 | -127,814003 |
| 10-JDWBC-3795 | 658 | HM865691 | Noctuidae | Noctuinae | Xestia speciosa | Hübner | deWaard, J.R. | 30-Jul-2009 | Canada | British Columbia | nr. Hazelton | Date Creek Silvicultural System | B3-T | 55,43899918 | -127,814003 |
| 10-JDWBC-4109 | 658 | HM866035 | Noctuidae | Noctuinae | Xestia speciosa | Hübner | deWaard, J.R. | 30-Jul-2009 | Canada | British Columbia | nr. Hazelton | Date Cr. Silvicultural System | A2-T | 55,4659996 | -127,8150024 |
| 10-JDWBC-4110 | 658 | HM866036 | Noctuidae | Noctuinae | Xestia speciosa | Hübner | deWaard, J.R. | 30-Jul-2009 | Canada | British Columbia | nr. Hazelton | Date Cr. Silvicultural System | A2-T | 55,4659996 | -127,8150024 |
| 10-JDWBC-4425 | 658 | HM866380 | Noctuidae | Noctuinae | Xestia speciosa | Hübner | deWaard, J.R. | 30-Jul-2009 | Canada | British Columbia | nr. Hazelton | Date Creek Silvicultural System | B1-T | 55,43700027 | -127,8010025 |
| 10-JDWBC-4426 | 658 | HM866381 | Noctuidae | Noctuinae | Xestia speciosa | Hübner | deWaard, J.R. | 30-Jul-2009 | Canada | British Columbia | nr. Hazelton | Date Creek Silvicultural System | B1-T | 55,43700027 | -127,8010025 |
| 10-JDWBC-4427 | 658 | HM866382 | Noctuidae | Noctuinae | Xestia speciosa | Hübner | deWaard, J.R. | 30-Jul-2009 | Canada | British Columbia | nr. Hazelton | Date Creek Silvicultural System | B1-T | 55,43700027 | -127,8010025 |
| 10-JDWBC-4681 | 658 | HM866658 | Noctuidae | Noctuinae | Xestia speciosa | Hübner | deWaard, J.R. | 30-Jul-2009 | Canada | British Columbia | nr. Hazelton | Date Cr. Silvicultural System | A4-T | 55,46500015 | -127,8059998 |
| MM00840 | 658 | HM871213 | Noctuidae | Noctuinae | Xestia speciosa | Hübner | Marko Mutanen | | Finland | | Ostrobottnia borealis pars australis | | Kiiminki | 65,07099915 | 25,72500038 |
| MM02739 | 658 | HM871686 | Noctuidae | Noctuinae | Xestia speciosa | Hübner | Marko Mutanen | | Finland | | Ostrobottnia borealis pars australis | | Kiiminki | 65,07099915 | 25,72500038 |
| MM06113 | 658 | HM873048 | Noctuidae | Noctuinae | Xestia speciosa | Hübner | Panu Vaelimaek | | Finland | | Regio kuusamoensis | | Kuusamo | 66,22299957 | 29,42200089 |
| MM08072 | 658 | HM873766 | Noctuidae | Noctuinae | Xestia speciosa | Hübner | Marko Mutanen, Panu Vaelimaeki | | Finland | | Lapponia kemensis pars occidentalis | | Kolari | 67,27600098 | 23,75300026 |
| MM08073 | 658 | HM873767 | Noctuidae | Noctuinae | Xestia speciosa | Hübner | Marko Mutanen, Panu Vaelimaeki | | Finland | | Lapponia kemensis pars occidentalis | | Kolari | 67,27600098 | 23,75300026 |
| MM15113 | 658 | HM876782 | Noctuidae | Noctuinae | Xestia speciosa | Hübner | Nils Ryrholm | | Sweden | | Haerjedalen | | Funaesdalen | 62,33039856 | 12,32279968 |
| MM15114 | 658 | HM876783 | Noctuidae | Noctuinae | Xestia speciosa | Hübner | Nils Ryrholm | | Sweden | | Haerjedalen | | Funaesdalen | 62,33039856 | 12,32279968 |
| MM15132 | 658 | HM876784 | Noctuidae | Noctuinae | Xestia speciosa | Hübner | Nils Ryrholm | | Sweden | | Haerjedalen | | Sveg | 62,6576004 | 14,14680004 |
| MM15133 | 658 | HM876785 | Noctuidae | Noctuinae | Xestia speciosa | Hübner | Nils Ryrholm | | Sweden | | Haerjedalen | | Sveg | 62,6576004 | 14,14680004 |
| MM15134 | 658 | HM876786 | Noctuidae | Noctuinae | Xestia speciosa | Hübner | Nils Ryrholm | | Sweden | | Haerjedalen | | Sveg | 62,6576004 | 14,14680004 |
| MM15135 | 658 | HM876787 | Noctuidae | Noctuinae | Xestia speciosa | Hübner | Nils Ryrholm | | Sweden | | Haerjedalen | | Sveg | 62,6576004 | 14,14680004 |
| MM15136 | 658 | HM876788 | Noctuidae | Noctuinae | Xestia speciosa | Hübner | Nils Ryrholm | | Sweden | | Haerjedalen | | Sveg | 62,6576004 | 14,14680004 |
| MM15137 | 658 | HM876789 | Noctuidae | Noctuinae | Xestia speciosa | Hübner | Nils Ryrholm | | Sweden | | Haerjedalen | | Sveg | 62,6576004 | 14,14680004 |
| MM15138 | 658 | HM876790 | Noctuidae | Noctuinae | Xestia speciosa | Hübner | Nils Ryrholm | | Sweden | | Haerjedalen | | Sveg | 62,6576004 | 14,14680004 |
| MM15139 | 658 | HM876791 | Noctuidae | Noctuinae | Xestia speciosa | Hübner | Nils Ryrholm | | Sweden | | Haerjedalen | | Sveg | 62,6576004 | 14,14680004 |
| MM18760 | 658 | JF854608 | Noctuidae | Noctuinae | Xestia speciosa | Hübner | Reima Leinonen | 16-Jul-1999 | Finland | | | | | | |
| MM18761 | 658 | JF854609 | Noctuidae | Noctuinae | Xestia speciosa | Hübner | Reima Leinonen | 28-Jul-2001 | Russia | | | | Venehjaerv | | |
| MM18762 | 658 | JF854610 | Noctuidae | Noctuinae | Xestia speciosa | Hübner | Reima Leinonen | 31-Jul-2003 | Finland | | Ostrobottnia kajanensis | | Kuhmo | 64,28500366 | 28,79599953 |
| TLMF Lep 00169 | 636 | GU689157 | Noctuidae | Noctuinae | Xestia speciosa | Hübner | Huemer P. & Erlebach S | 25-Aug-2005 | Austria | Salzburg | | Wildgerlostal, S Trisslalm- Krimml | | 47,18999863 | 12,11299992 |
| TLMF Lep 00170 | 657 | GU689158 | Noctuidae | Noctuinae | Xestia speciosa | Hübner | Huemer P. & Erlebach S | 25-Aug-2005 | Austria | Salzburg | | Wildgerlostal, S Trisslalm- Krimml | | 47,18999863 | 12,11299992 |
| TLMF Lep 00182 | 658 | GU689197 | Noctuidae | Noctuinae | Xestia speciosa | Hübner | Huemer P | 21-Jul-2006 | Austria | Vorarlberg | | Fohramoos SE | | 47,41899872 | 9,8070016 |
| TLMF Lep 00183 | 590 | GU689151 | Noctuidae | Noctuinae | Xestia speciosa | Hübner | Huemer P | 21-Jul-2006 | Austria | Vorarlberg | | Fohramoos SE | | 47,41899872 | 9,8070016 |
| TLMF Lep 00250 | 658 | HM425759 | Noctuidae | Noctuinae | Xestia speciosa | Hübner | Huemer P | 13-Jul-2009 | Austria | Karnten | | | Petzen N, Obere Krischa | 46,5060997 | 14,75699997 |
| TLMF Lep 00316 | 658 | HM425811 | Noctuidae | Noctuinae | Xestia speciosa | Hübner | Huemer P. | 20-Jul-2009 | Italy | | Belluno | | Passo di Valparola E - Passo Falzarego | 46,5223999 | 12,00699997 |
| TLMF Lep 02458 | 658 | JF859999 | Noctuidae | Noctuinae | Xestia speciosa | Hübner | Huemer P | 19-Jul-2010 | Italy | South Tyrol | | | Ritten/ Obergruenwal | 46,59700012 | 11,43900013 |
| TLMF Lep 02969 | 658 | JF860402 | Noctuidae | Noctuinae | Xestia speciosa | Hübner | Huemer P | 26-Jul-2001 | Italy | | Udine | | Pontebba/ Torbiera di Pramoll | 46,55599976 | 13,28899956 |
| TLMF Lep 02970 | 658 | JF860403 | Noctuidae | Noctuinae | Xestia speciosa | Hübner | Huemer P. | 17-Jul-2007 | Austria | Tyrol | Osttirol | | Trojeralmtal, Ht. Trojeralm/ St. Jakob in Deferegger | 46,95100021 | 12,31000042 |
| TLMF Lep 04431 | 658 | JN284164 | Noctuidae | Noctuinae | Xestia speciosa | Hübner | Huemer P | 26-Jul-2005 | Austria | Vorarlberg | | | Fohramoos ENE | 47,42129898 | 9,806329727 |
| TLMF Lep 04432 | 658 | JN284165 | Noctuidae | Noctuinae | Xestia speciosa | Hübner | Huemer P | 26-Jul-2005 | Austria | Vorarlberg | | | Fohramoos ENE | 47,42129898 | 9,806329727 |
| BC ZSM Lep 36633 | 420 | JX034579 | Zygaenidae | Zygaeninae | Zygaena exulans | Hohenwarth | R. Oswald | 08-Jul-2002 | Austria | Tyrol | North Tirol | | Stubai, Gleirschjoech | 47,14839935 | 11,06620026 |
| MM15793 | 658 | HQ570417 | Zygaenidae | Zygaeninae | Zygaena exulans | Hohenwarth | Marko Mutanen | | Finland | | Lapponia enontekiensis | | Enontekioe | 68,99700165 | 20,74399948 |
| MM15794 | 658 | HQ570418 | Zygaenidae | Zygaeninae | Zygaena exulans | Hohenwarth | Marko Mutanen | | Finland | | Lapponia enontekiensis | | Enontekioe | 68,99700165 | 20,74399948 |
| MM18406 | 658 | JX034642 | Zygaenidae | Zygaeninae | Zygaena exulans | Hohenwarth | Marko Mutanen | | Finland | | Lapponia enontekiensis | | Enontekioe | 68,99659729 | 20,74370003 |
| TLMF Lep 00153 | 658 | GU689169 | Zygaenidae | Zygaeninae | Zygaena exulans | Hohenwarth | Huemer P. | 05-Aug-2008 | Italy | Piedmont | Cuneo | Colle Valcavera Umgebung- Demonte NW | | 44,38100052 | 7,102000237 |
| TLMF Lep 00385 | 647 | HM425872 | Zygaenidae | Zygaeninae | Zygaena exulans | Hohenwarth | Huemer P | 01-Aug-2009 | Austria | Karnten | | | Mallnitz, Doesental, Seealm | 46,99309921 | 13,27110004 |

| | | | | | | | | | | | | | | |
|---|---|---|---|---|---|---|---|---|---|---|---|---|---|---|
| TLMF Lep 01827 | 658 | HQ968290 | Zygaenidae | Zygaeninae | Zygaena exulans | Hohenwarth | Mayr T. | 01-Aug-2009 | Switzerland | Graubunden | | Val Bever, Alp Suvretta | 46,54999924 | 9,783329964 |
| TLMF Lep 01828 | 658 | HQ968291 | Zygaenidae | Zygaeninae | Zygaena exulans | Hohenwarth | Mayr T. | 01-Aug-2009 | Switzerland | Graubunden | | Val Bever, Alp Suvretta | 46,54999924 | 9,783329964 |
| TLMF Lep 02014 | 658 | HQ968424 | Zygaenidae | Zygaeninae | Zygaena exulans | Hohenwarth | Huemer P. | 13-Jul-2010 | Italy | Abruzzi | L`Aquila | NP Gran Sasso, Campo<br>Imperatore, E Observatorio | 42,44829941 | 13,57330036 |
| TLMF Lep 02912 | 658 | JX034643 | Zygaenidae | Zygaeninae | Zygaena exulans | Hohenwarth | Huemer P. | 04-Aug-2010 | France | Provence-Alpes-<br>Cote d'Azur | | Col Agnel | 44,68899918 | 6,984000206 |
| TLMF Lep 02913 | 658 | JX034620 | Zygaenidae | Zygaeninae | Zygaena exulans | Hohenwarth | Huemer P. | 04-Aug-2010 | France | Provence-Alpes-<br>Cote d'Azur | | Col Agnel | 44,68899918 | 6,984000206 |
