# Supplementary material for: Allopatry as a Gordian Knot for Taxonomists: Patterns of DNA Barcode Divergence in Arctic-Alpine Lepidoptera
Source: PLoS One. 2012 Oct 11;7(10):e47214. doi: 10.1371/journal.pone.0047214 (PMC3469483; doi:10.1371/journal.pone.0047214)
Supplement: Table S2 — Provisional check-list of arctic-alpine and boreo-montane species shared between the Alps and Fennoscandia but not barcoded from both major distribution areas in this study; taxonomic status of several taxa needs revision. (PDF) [file pone.0047214.s002.pdf]

Table S2. Provisional check-list of arctic-alpine and boreo-montane species shared between the Alps and Fennoscandia but not barcoded from both major distribution areas in this study; taxonomic status of several taxa needs revision.

**Nepticulidae**
*Stigmella vimineticola*
*Stigmella dryadella*
*Enteucha acetosae*
**Adelidae**
*Nematopogon magna*
**Prodoxidae**
*Lampronia aeripennella*
**Tineidae**
*Niditinea truncicolella*
*Nemapogon fungivorella*
*Agnathosia mendicella*
**Psychidae**
*Siederia rupicolella*
*Dahlica charlottae*
**Bucculatricidae**
*Bucculatrix argentisignella*
**Gracillariidae**
*Phyllonorycter pyrifoliella*
*Callisto insperatella*
*Gracillaria loriolella*
**Yponomeutidae**
*Paraswammerdamia conspersella*
**Nepticulidae**
*Lyonetia pulverulentella*
**Elachistidae**
*Depressaria silesiaca*
*Elachista festucicolella*
*Elachista compsa*
*Elachista excelsicola*
*Elachista kilmunella*
*Elachista ornithopodella*
*Elachista tengstromi*
*Elachista tetragonella*
*Elachista zernyi*
**Scythrididae**
*Scythris disparella*
**Coleophoridae**
*Coleophora idaeella*
*Coleophora ledi*
*Coleophora graminicolella*
*Coleophora paradrymidis*
**Gelechiidae**
*Monochroa inflexella*
*Monochroa ferrea*
*Aristotelia heliacella*
*Athrips pruinosella*
*Filatima incomptella*
*Gelechia cuneatella*
*Caryocolum cassella*
*Scrobipalpula diffluella*
**Tortricidae**
*Argyroploce lediana*
*Argyroploce concretana*
*Phiaris dissolutana*
*Aterpia sieversiana*
*Pelochrista huebneriana*
*Epinotia gimmerthaliana*
*Aethes fennicana*
*Clepsis lindebergi*
**Epermenidae**
*Epermenia profugella*
**Crambidae**
*Loxostege manualis*
*Metaxmeste phrygialis*
*Catoptria maculalis*
*Agriphila biarmicus*
**Lycaenidae**
*Plebejus glandon*
*Plebeius orbitulus*
**Nymphalidae**
*Boloria titania*
**Lasiocampidae**
*Eriogaster arbusculae*
**Geometridae**
*Eupithecia conterminata*
*Eupithecia veratraria*
*Baptria tibiale*
*Macaria carbonaria*
**Erebidae**
*Gynaephora selenitica*
**Noctuidae**
*Agrotis fatidica*
*Xestia sincera*
*Spaelotis suecica*
*Sympistis funebris*
*Acronicta cinerea*
