# Supplementary material for: Allopatry as a Gordian Knot for Taxonomists: Patterns of DNA Barcode Divergence in Arctic-Alpine Lepidoptera
Source: PLoS One. 2012 Oct 11;7(10):e47214. doi: 10.1371/journal.pone.0047214 (PMC3469483; doi:10.1371/journal.pone.0047214)
Supplement: Table S3 — Minimum, mean and maximum intraspecific variation in DNA barcodes in study species within and between the study regions. NA refers to North America, AL to Alps and FE to Fennoscandia. Species showing over 2% intraspecific divergence within or between the regions in question are shown highlighted. (PDF) [file pone.0047214.s003.pdf]

Table S3. Minimum, mean and maximum intraspecific variation in DNA barcodes in study species within and between the study regions. NA refers to North America, AL to Alps and FE to Fennoscandia. Species showing over 2% intraspecific divergence within or between the regions in question are shown highlighted.

**Gazoryctra ganna**						
	AL-AL	FE-FE	NA-NA	AL-FE	AL-NA	FE-NA
mean	0.3067	0.3067	N/A	1.4622	N/A	N/A
min	0.15	0	N/A	1.24	N/A	N/A
max	0.46	0.46	N/A	1.71	N/A	N/A
**Stigmella pretiosa**						
	AL-AL	FE-FE	NA-NA	AL-FE	AL-NA	FE-NA
mean	N/A	0	N/A	0.17	N/A	N/A
min	N/A	0	N/A	0.17	N/A	N/A
max	N/A	0	N/A	0.17	N/A	N/A
**Alloclemensia mesospilella**						
	AL-AL	FE-FE	NA-NA	AL-FE	AL-NA	FE-NA
mean	N/A	0.3066	N/A	0.72	N/A	N/A
min	N/A	0.15	N/A	0.62	N/A	N/A
max	N/A	0.46	N/A	0.77	N/A	N/A
**Incurvaria vetulella**						
	AL-AL	FE-FE	NA-NA	AL-FE	AL-NA	FE-NA
mean	0	0.123	N/A	**2.8213**	N/A	N/A
min	0	0	N/A	**2.42**	N/A	N/A
max	0	0.31	N/A	**3.14**	N/A	N/A
**Lampronia standfussiella**						
	AL-AL	FE-FE	NA-NA	AL-FE	AL-NA	FE-NA
mean	1.08	0.2067	N/A	1.735	N/A	N/A
min	1.08	0	N/A	1.55	N/A	N/A
max	1.08	0.31	N/A	**2.03**	N/A	N/A
**Sterrhopterix standfussi**						
	AL-AL	FE-FE	NA-NA	AL-FE	AL-NA	FE-NA
mean	N/A	0.1517	N/A	0.075	N/A	N/A
min	N/A	0	N/A	0	N/A	N/A
max	N/A	0.31	N/A	0.15	N/A	N/A
**Callisto coffeella**						
	AL-AL	FE-FE	NA-NA	AL-FE	AL-NA	FE-NA
mean	0.9655	0.385	N/A	1.4019	N/A	N/A
min	0	0	N/A	0.31	N/A	N/A
max	1.89	0.77	N/A	**2.37**	N/A	N/A
**Euhyponomeutoides albithoracellus**						
	AL-AL	FE-FE	NA-NA	AL-FE	AL-NA	FE-NA
mean	0.7433	0.21	N/A	1.1367	N/A	N/A
min	0.61	0.15	N/A	0.64	N/A	N/A
max	0.97	0.32	N/A	1.44	N/A	N/A
**Kessleria fasciapennella**						
	AL-AL	FE-FE	NA-NA	AL-FE	AL-NA	FE-NA
mean	0	0.1	N/A	0.05	N/A	N/A
min	0	0	N/A	0	N/A	N/A
max	0	0.15	N/A	0.15	N/A	N/A
**Tinagma dryadis**						
	AL-AL	FE-FE	NA-NA	AL-FE	AL-NA	FE-NA
mean	N/A	0	0.0425	0.31	1.425	1.5855
min	N/A	0	0	0	1.28	1.44

max	N/A	0	0.49	0.31	1.81	1.97
**Hypatopa segnella**						
	AL-AL	FE-FE	NA-NA	AL-FE	AL-NA	FE-NA
mean	0	N/A	N/A	0.15	N/A	N/A
min	0	N/A	N/A	0.15	N/A	N/A
max	0	N/A	N/A	0.15	N/A	N/A
**Aplota nigricans**						
	AL-AL	FE-FE	NA-NA	AL-FE	AL-NA	FE-NA
mean	0	0	N/A	0	N/A	N/A
min	0	0	N/A	0	N/A	N/A
max	0	0	N/A	0	N/A	N/A
**Pseudatemelia elsae**						
	AL-AL	FE-FE	NA-NA	AL-FE	AL-NA	FE-NA
mean	N/A	0	N/A	0.24	N/A	N/A
min	N/A	0	N/A	0.22	N/A	N/A
max	N/A	0	N/A	0.28	N/A	N/A
**Elachista elsaella**						
	AL-AL	FE-FE	NA-NA	AL-FE	AL-NA	FE-NA
mean	N/A	0	N/A	0.77	N/A	N/A
min	N/A	0	N/A	0.77	N/A	N/A
max	N/A	0	N/A	0.77	N/A	N/A
**Elachista subalbidella**						
	AL-AL	FE-FE	NA-NA	AL-FE	AL-NA	FE-NA
mean	0	0.0655	N/A	0.4345	N/A	N/A
min	0	0	N/A	0.18	N/A	N/A
max	0	0.36	N/A	0.46	N/A	N/A
**Depressaria leucocephala**						
	AL-AL	FE-FE	NA-NA	AL-FE	AL-NA	FE-NA
mean	0	0	N/A	0	N/A	N/A
min	0	0	N/A	0	N/A	N/A
max	0	0	N/A	0	N/A	N/A
**Exaeretia ciniflonella**						
	AL-AL	FE-FE	NA-NA	AL-FE	AL-NA	FE-NA
mean	N/A	0.195	N/A	0.124	N/A	N/A
min	N/A	0	N/A	0	N/A	N/A
max	N/A	0.62	N/A	0.47	N/A	N/A
**Levipalpus hepatariella**						
	AL-AL	FE-FE	NA-NA	AL-FE	AL-NA	FE-NA
mean	0.51	0	N/A	0.77	N/A	N/A
min	0.15	0	N/A	0.62	N/A	N/A
max	0.77	0	N/A	0.92	N/A	N/A
**Coleophora pappiferella**						
	AL-AL	FE-FE	NA-NA	AL-FE	AL-NA	FE-NA
mean	N/A	0.155	N/A	0.0775	N/A	N/A
min	N/A	0	N/A	0	N/A	N/A
max	N/A	0	N/A	0.31	N/A	N/A
**Coleophora svenssoni**						
	AL-AL	FE-FE	NA-NA	AL-FE	AL-NA	FE-NA
mean	N/A	0.1	N/A	**4.67**	N/A	N/A
min	N/A	0	N/A	**4.61**	N/A	N/A
max	N/A	0.15	N/A	**4.79**	N/A	N/A
**Coleophora unigenella**						
	AL-AL	FE-FE	NA-NA	AL-FE	AL-NA	FE-NA
mean	N/A	0	N/A	0.76	N/A	N/A

min	N/A	0	N/A	0.75	N/A	N/A
max	N/A	0	N/A	0.77	N/A	N/A
**Coleophora uliginosella**						
	AL-AL	FE-FE	NA-NA	AL-FE	AL-NA	FE-NA
mean	N/A	N/A	N/A	0	N/A	N/A
min	N/A	N/A	N/A	0	N/A	N/A
max	N/A	N/A	N/A	0	N/A	N/A
**Bryotropha boreella**						
	AL-AL	FE-FE	NA-NA	AL-FE	AL-NA	FE-NA
mean	0.075	0	N/A	0.3475	N/A	N/A
min	0	0	N/A	0.31	N/A	N/A
max	0.15	0	N/A	0.46	N/A	N/A
**Caryocolum petrophila**						
	AL-AL	FE-FE	NA-NA	AL-FE	AL-NA	FE-NA
mean	1.325	0	N/A	1.2025	N/A	N/A
min	0	0	N/A	0.77	N/A	N/A
max	**2.03**	0	N/A	**2.19**	N/A	N/A
**Caryocolum petryi**						
	AL-AL	FE-FE	NA-NA	AL-FE	AL-NA	FE-NA
mean	0.244	0	N/A	0.276	N/A	N/A
min	0	0	N/A	0.15	N/A	N/A
max	0.61	0	N/A	0.62	N/A	N/A
**Caryocolum pullatella**						
	AL-AL	FE-FE	NA-NA	AL-FE	AL-NA	FE-NA
mean	0.54	0.3333	1.87325	**3.31**	**4.7539**	**5.3173**
min	0.15	0	0	**2.99**	**3.79**	**4.78**
max	0.93	0.46	**4.56**	**3.47**	**5.63**	**6.38**
**Chionodes holosericella**						
	AL-AL	FE-FE	NA-NA	AL-FE	AL-NA	FE-NA
mean	0.064	0.1	N/A	0.482667	N/A	N/A
min	0	0	N/A	0.32	N/A	N/A
max	0.16	0.15	N/A	0.61	N/A	N/A
**Chionodes luctuella**						
	AL-AL	FE-FE	NA-NA	AL-FE	AL-NA	FE-NA
mean	0	0.1	N/A	**2.285**	N/A	N/A
min	0	0	N/A	**2.19**	N/A	N/A
max	0	0.15	N/A	**2.44**	N/A	N/A
**Chionodes lugubrella**						
	AL-AL	FE-FE	NA-NA	AL-FE	AL-NA	FE-NA
mean	0.4133	0.21	1.9856	1.211111	**5.2256**	**5.4189**
min	0	0.16	0	0.99	**4.11**	**4.77**
max	0.62	0.31	**3.47**	1.4	**5.95**	**6.29**
**Chionodes viduella**						
	AL-AL	FE-FE	NA-NA	AL-FE	AL-NA	FE-NA
mean	1.025	0.937	N/A	1.2984	N/A	N/A
min	0	0	N/A	0.46	N/A	N/A
max	1.55	**2.35**	N/A	**2.35**	N/A	N/A
**Gnorimoschema valesiella**						
	AL-AL	FE-FE	NA-NA	AL-FE	AL-NA	FE-NA
mean	N/A	0	N/A	0.62	N/A	N/A
min	N/A	0	N/A	0.62	N/A	N/A
max	N/A	0	N/A	0.62	N/A	N/A
**Syncopacma karvoneni**						
	AL-AL	FE-FE	NA-NA	AL-FE	AL-NA	FE-NA

mean	N/A	1.0233	N/A	1.678	N/A	N/A
min	N/A	0	N/A	1.55	N/A	N/A
max	N/A	1.87	N/A	1.71	N/A	N/A
**Neofaculta infernella**						
	AL-AL	FE-FE	NA-NA	AL-FE	AL-NA	FE-NA
mean	0.5136	**4.810**	1.58	**3.8526**	**8.9183**	**5.8769**
min	0	0	0	0.31	**8.48**	0.31
max	**2.29**	**8.88**	**2.37**	**9.01**	**9.28**	**9.1**
**Prolita sexpunctella**						
	AL-AL	FE-FE	NA-NA	AL-FE	AL-NA	FE-NA
mean	0.0858	0.5133	0.2566	0.46	**2.3878**	**2.4048**
min	N/A	0	0	0	**2.02**	**2.02**
max	0.31	0.77	0.62	0.83	**2.74**	**2.74**
**Scrobipalpa murinella**						
	AL-AL	FE-FE	NA-NA	AL-FE	AL-NA	FE-NA
mean	0.1	0.9267	N/A	0.6178	N/A	N/A
min	0	0.15	N/A	0	N/A	N/A
max	0.15	1.39	N/A	1.55	N/A	N/A
**Scrobipalpopsis petasitis**						
	AL-AL	FE-FE	NA-NA	AL-FE	AL-NA	FE-NA
mean	0.0857	0	N/A	0.8614	N/A	N/A
min	0	0	N/A	0.77	N/A	N/A
max	0.15	0	N/A	0.93	N/A	N/A
**Oidaematophorus rogenhoferi**						
	AL-AL	FE-FE	NA-NA	AL-FE	AL-NA	FE-NA
mean	0.41	0.3067	N/A	0.4367	N/A	N/A
min	0	0	N/A	0.15	N/A	N/A
max	0.62	0.46	N/A	0.77	N/A	N/A
**Acleris maccana**						
	AL-AL	FE-FE	NA-NA	AL-FE	AL-NA	FE-NA
mean	0	0.0585	0.1374	0.03	0.81	0.7830
min	0	0	0	0	0.53	0.49
max	0	0.15	0.61	0.15	1.08	1.08
**Aethes deutschiana**						
	AL-AL	FE-FE	NA-NA	AL-FE	AL-NA	FE-NA
mean	0.16	0.10	0.3651	0.41	**4.54**	**4.64**
min	0	0	0	0.17	**4.05**	**4.42**
max	0.33	0.15	1.7	0.77	**4.93**	**4.92**
**Ancylis habeleri**						
	AL-AL	FE-FE	NA-NA	AL-FE	AL-NA	FE-NA
mean	N/A	0.0916	N/A	0.0775	N/A	N/A
min	N/A	0	N/A	0.15	N/A	N/A
max	N/A	0.32	N/A	0.31	N/A	N/A
**Argyroploce arbutella**						
	AL-AL	FE-FE	NA-NA	AL-FE	AL-NA	FE-NA
mean	0	0.2033	N/A	0.2567	N/A	N/A
min	0	0.15	N/A	0.15	N/A	N/A
max	0	0.31	N/A	0.31	N/A	N/A
**Argyroploce noricana**						
	AL-AL	FE-FE	NA-NA	AL-FE	AL-NA	FE-NA
mean	0	0	N/A	0	N/A	N/A
min	0	0	N/A	0	N/A	N/A
max	0	0	N/A	0	N/A	N/A
**Argyroploce roseomaculana**						

	AL-AL	FE-FE	NA-NA	AL-FE	AL-NA	FE-NA
mean	0	0	N/A	0.045	N/A	N/A
min	0	0	N/A	0	N/A	N/A
max	0	0	N/A	0	N/A	N/A
**Clepsis rogana**						
	AL-AL	FE-FE	NA-NA	AL-FE	AL-NA	FE-NA
mean	1.2268	N/A	N/A	1.3267	N/A	N/A
min	0	N/A	N/A	0.94	N/A	N/A
max	**2.03**	N/A	N/A	1.58	N/A	N/A
**Epinotia mercuriana**						
	AL-AL	FE-FE	NA-NA	AL-FE	AL-NA	FE-NA
mean	0.678	0.1	N/A	**2.7133**	N/A	N/A
min	0	0	N/A	**2.5**	N/A	N/A
max	1.24	0.15	N/A	**3.14**	N/A	N/A
**Epinotia nemorivaga**						
	AL-AL	FE-FE	NA-NA	AL-FE	AL-NA	FE-NA
mean	N/A	0.785	N/A	0.6975	N/A	N/A
min	N/A	0	N/A	0.31	N/A	N/A
max	N/A	1.55	N/A	1.23	N/A	N/A
**Grapholita aureolana**						
	AL-AL	FE-FE	NA-NA	AL-FE	AL-NA	FE-NA
mean	0.17	0	0	0.4213	0.085	0.47
min	0.17	0	0	0.34	0	0.46
max	0.17	0	0	0.5	0.17	0.48
**Phiaris schulziana**						
	AL-AL	FE-FE	NA-NA	AL-FE	AL-NA	FE-NA
mean	0	0.3484	0.06	0.6288	1.8275	**2.0953**
min	0	0	0	0.46	1.71	1.55
max	0	1.08	0.16	0.77	**2.74**	**2.52**
**Phteochroa vulneratana**						
	AL-AL	FE-FE	NA-NA	AL-FE	AL-NA	FE-NA
mean	N/A	0.3067	N/A	0.23	N/A	N/A
min	N/A	0	N/A	0	N/A	N/A
max	N/A	0.46	N/A	0.46	N/A	N/A
**Sparganothis praecana**						
	AL-AL	FE-FE	NA-NA	AL-FE	AL-NA	FE-NA
mean	N/A	0.1	N/A	0.3633	N/A	N/A
min	N/A	0	N/A	0.27	N/A	N/A
max	N/A	0.15	N/A	0.55	N/A	N/A
**Synanthedon polaris**						
	AL-AL	FE-FE	NA-NA	AL-FE	AL-NA	FE-NA
mean	N/A	0.1233	N/A	**9.7033**	N/A	N/A
min	N/A	0	N/A	**9.42**	N/A	N/A
max	N/A	0.22	N/A	**10.08**	N/A	N/A
**Zygaena exulans**						
	AL-AL	FE-FE	NA-NA	AL-FE	AL-NA	FE-NA
mean	0.81	0.3067	N/A	1.3962	N/A	N/A
min	0	0	N/A	1.1	N/A	N/A
max	1.89	0.46	N/A	1.86	N/A	N/A
**Parnassius phoebus**						
	AL-AL	FE-FE	NA-NA	AL-FE	AL-NA	FE-NA
mean	0	N/A	N/A	N/A	1.55	N/A
min	0	N/A	N/A	N/A	1.55	N/A
max	0	N/A	N/A	N/A	1.55	N/A

**Pyrgus andromedae**						
	AL-AL	FE-FE	NA-NA	AL-FE	AL-NA	FE-NA
mean	0	0.1333	N/A	0.7475	N/A	N/A
min	0	0	N/A	0.62	N/A	N/A
max	0	0.32	N/A	0.96	N/A	N/A
**Aricia nicias**						
	AL-AL	FE-FE	NA-NA	AL-FE	AL-NA	FE-NA
mean	0	0.2033	N/A	0.5667	N/A	N/A
min	0	0.15	N/A	0.46	N/A	N/A
max	0	0.31	N/A	0.62	N/A	N/A
**Plebeius optilete**						
	AL-AL	FE-FE	NA-NA	AL-FE	AL-NA	FE-NA
mean	N/A	0	0	0.46	1.29	1.785
min	N/A	0	0	0.46	1.28	1.77
max	N/A	0	0	0.46	1.3	1.8
**Boloria eunomia**						
	AL-AL	FE-FE	NA-NA	AL-FE	AL-NA	FE-NA
mean	0.336	0.1	0.0705	1.042	**2.2218**	1.8523
min	0	0	0	0.92	1.81	1.48
max	0.77	0.15	0.65	1.24	**2.5**	**2.18**
**Boloria napaea**						
	AL-AL	FE-FE	NA-NA	AL-FE	AL-NA	FE-NA
mean	0.6133	0	N/A	**2.0233**	N/A	N/A
min	0	0	N/A	1.71	N/A	N/A
max	1.16	0	N/A	**2.34**	N/A	N/A
**Boloria thore**						
	AL-AL	FE-FE	NA-NA	AL-FE	AL-NA	FE-NA
mean	0.5289	0.21	N/A	0.3985	N/A	N/A
min	0	0	N/A	0.15	N/A	N/A
max	2.38	0.32	N/A	**2.55**	N/A	N/A
**Erebia pandrose**						
	AL-AL	FE-FE	NA-NA	AL-FE	AL-NA	FE-NA
mean	0.4068	0	N/A	0.3463	N/A	N/A
min	0	0	N/A	0.15	N/A	N/A
max	0.77	0	N/A	0.62	N/A	N/A
**Catoptria furcatella**						
	AL-AL	FE-FE	NA-NA	AL-FE	AL-NA	FE-NA
mean	0.3067	0	N/A	0.2033	N/A	N/A
min	0.15	0	N/A	0	N/A	N/A
max	0.46	0	N/A	0.46	N/A	N/A
**Crambus alienellus**						
	AL-AL	FE-FE	NA-NA	AL-FE	AL-NA	FE-NA
mean	0	0	0.2121	0	**3.5388**	**3.5388**
min	0	0	0	0	**3.47**	**3.47**
max	0	0	0.49	0	**3.7**	**3.7**
**Eudonia sudetica**						
	AL-AL	FE-FE	NA-NA	AL-FE	AL-NA	FE-NA
mean	**2.9179**	0	N/A	**2.865**	N/A	N/A
min	0	0	N/A	1.4	N/A	N/A
max	**6.32**	0	N/A	**6.32**	N/A	N/A
**Gesneria centuriella**						
	AL-AL	FE-FE	NA-NA	AL-FE	AL-NA	FE-NA
mean	N/A	0	0.9758	0	0.6443	0.6277
min	N/A	0	0	0	0	0

max	N/A	0	**2.82**	0	**2.5**	**2.5**
**Metaxmeste schrankiana**						
	AL-AL	FE-FE	NA-NA	AL-FE	AL-NA	FE-NA
mean	0.621	0	N/A	0.6825	N/A	N/A
min	0	0	N/A	0.31	N/A	N/A
max	1.11	0	N/A	0.95	N/A	N/A
**Udea decrepitalis**						
	AL-AL	FE-FE	NA-NA	AL-FE	AL-NA	FE-NA
mean	0.265	0.1	N/A	0.2092	N/A	N/A
min	0.15	0	N/A	0	N/A	N/A
max	0.46	0.15	N/A	0.46	N/A	N/A
**Udea elutalis**						
	AL-AL	FE-FE	NA-NA	AL-FE	AL-NA	FE-NA
mean	N/A	0	N/A	1.58	N/A	N/A
min	N/A	0	N/A	1.55	N/A	N/A
max	N/A	0	N/A	1.64	N/A	N/A
**Udea inquinatalis**						
	AL-AL	FE-FE	NA-NA	AL-FE	AL-NA	FE-NA
mean	0.1	0.1	0.2173	0.1522	0.5641	0.6191
min	0	0	0	0	0.46	0.46
max	0.15	0.15	0.78	0.31	0.92	0.93
**Udea nebulalis**						
	AL-AL	FE-FE	NA-NA	AL-FE	AL-NA	FE-NA
mean	0.276	0.1033	N/A	0.4227	N/A	N/A
min	0	0	N/A	0.31	N/A	N/A
max	0.46	0.16	N/A	0.62	N/A	N/A
**Arichanna melanaria**						
	AL-AL	FE-FE	NA-NA	AL-FE	AL-NA	FE-NA
mean	N/A	0.2033	0	0.1	0	0.1033
min	N/A	0.15	0	0	0	0
max	N/A	0.31	0	0.15	0	0.16
**Carsia sororiata**						
	AL-AL	FE-FE	NA-NA	AL-FE	AL-NA	FE-NA
mean	0.15	0.5167	1.25	0.695	1.325	1.245
min	0.15	0.31	1.25	0.62	0.93	0.77
max	0.15	0.62	1.25	0.77	1.72	1.72
**Colostygia turbata**						
	AL-AL	FE-FE	NA-NA	AL-FE	AL-NA	FE-NA
mean	0.4618	0	N/A	**3.2447**	N/A	N/A
min	0	0	N/A	**2.99**	N/A	N/A
max	0.93	0	N/A	**3.53**	N/A	N/A
**Elophos vittaria**						
	AL-AL	FE-FE	NA-NA	AL-FE	AL-NA	FE-NA
mean	0.4133	0.1	N/A	**6.2167**	N/A	N/A
min	0	0	N/A	**5.87**	N/A	N/A
max	0.62	0.15	N/A	**6.47**	N/A	N/A
**Entephria nobiliaria**						
	AL-AL	FE-FE	NA-NA	AL-FE	AL-NA	FE-NA
mean	0.3559	0	N/A	0.1855	N/A	N/A
min	0	0	N/A	0	N/A	N/A
max	1.87	0	N/A	1.71	N/A	N/A
**Eupithecia cretaceata**						
	AL-AL	FE-FE	NA-NA	AL-FE	AL-NA	FE-NA
mean	0.4738	N/A	0.6242	N/A	**6.3881**	N/A

min	0	N/A	0	N/A	**5.78**	N/A
max	1.26	N/A	1.4	N/A	**6.83**	N/A
**Gnophos obfuscata**						
	AL-AL	FE-FE	NA-NA	AL-FE	AL-NA	FE-NA
mean	**2.1791**	0.1	N/A	1.664	N/A	N/A
min	0	0	N/A	0.31	N/A	N/A
max	**3.95**	0.15	N/A	**3.63**	N/A	N/A
**Lampropteryx otregiata**						
	AL-AL	FE-FE	NA-NA	AL-FE	AL-NA	FE-NA
mean	0	0.075	N/A	0.0375	N/A	N/A
min	0	0	N/A	0	N/A	N/A
max	0	0.15	N/A	0.15	N/A	N/A
**Macaria fusca**						
	AL-AL	FE-FE	NA-NA	AL-FE	AL-NA	FE-NA
mean	0.7206	0.1	N/A	1.0867	N/A	N/A
min	0	0	N/A	0.31	N/A	N/A
max	1.87	0.15	N/A	1.71	N/A	N/A
**Martania taeniata**						
	AL-AL	FE-FE	NA-NA	AL-FE	AL-NA	FE-NA
mean	N/A	0.5167	N/A	0.31	N/A	N/A
min	N/A	0	N/A	0.15	N/A	N/A
max	N/A	0.78	N/A	0.62	N/A	N/A
**Perizoma minorata**						
	AL-AL	FE-FE	NA-NA	AL-FE	AL-NA	FE-NA
mean	0	0	N/A	0	N/A	N/A
min	0	0	N/A	0	N/A	N/A
max	0	0	N/A	0	N/A	N/A
**Psodos coracina**						
	AL-AL	FE-FE	NA-NA	AL-FE	AL-NA	FE-NA
mean	0.7755	0.0682	N/A	0.9176	N/A	N/A
min	0.15	0	N/A	0.15	N/A	N/A
max	1.39	0.15	N/A	1.24	N/A	N/A
**Rheumaptera hastata**						
	AL-AL	FE-FE	NA-NA	AL-FE	AL-NA	FE-NA
mean	0.4221	0.318	N/A	0.541	N/A	N/A
min	0	0	N/A	0	N/A	N/A
max	0.95	0.5	N/A	0.95	N/A	N/A
**Rheumaptera subhastata**						
	AL-AL	FE-FE	NA-NA	AL-FE	AL-NA	FE-NA
mean	**3.1383**	1.8264	N/A	**2.4252**	N/A	N/A
min	0	0	N/A	0	N/A	N/A
max	**4.75**	**5.07**	N/A	**5.07**	N/A	N/A
**Xanthorhoe decoloraria**						
	AL-AL	FE-FE	NA-NA	AL-FE	AL-NA	FE-NA
mean	0.336	0.4067	0.4862	0.7987	1.8171	1.9893
min	0	0	0	0	1.55	1.55
max	0.77	0.61	**2.22**	1.08	**2.1**	**2.37**
**Xanthorhoe incursata**						
	AL-AL	FE-FE	NA-NA	AL-FE	AL-NA	FE-NA
mean	0	N/A	**2.42**	N/A	1.298	N/A
min	0	N/A	**2.42**	N/A	0.68	N/A
max	0	N/A	**2.42**	N/A	1.71	N/A
**Grammia quenseli**						
	AL-AL	FE-FE	NA-NA	AL-FE	AL-NA	FE-NA

mean	0.1533	0	1.4049	1.5767	**5.1497**	**5.71**
min	0	0	0	1.55	**4.12**	**4.62**
max	0.46	0	**4.59**	1.71	**6.12**	**5.95**
**Setema cereola**						
	AL-AL	FE-FE	NA-NA	AL-FE	AL-NA	FE-NA
mean	0.09	0	N/A	0.4225	N/A	N/A
min	0	0	N/A	0.31	N/A	N/A
max	0.15	0	N/A	0.46	N/A	N/A
**Anarta melanopa**						
	AL-AL	FE-FE	NA-NA	AL-FE	AL-NA	FE-NA
mean	0	0.075	N/A	0.19	N/A	N/A
min	0	0	N/A	0.15	N/A	N/A
max	0	0.15	N/A	0.31	N/A	N/A
**Coenophila subrosea**						
	AL-AL	FE-FE	NA-NA	AL-FE	AL-NA	FE-NA
mean	0.15	0	N/A	0.092	N/A	N/A
min	0.15	0	N/A	0	N/A	N/A
max	0.15	0	N/A	0.16	N/A	N/A
**Coranarta cordigera**						
	AL-AL	FE-FE	NA-NA	AL-FE	AL-NA	FE-NA
mean	0	0.5133	N/A	0.2567	N/A	N/A
min	0	0.46	N/A	0.15	N/A	N/A
max	0	0.62	N/A	0.31	N/A	N/A
**Standfussiana lucernea**						
	AL-AL	FE-FE	NA-NA	AL-FE	AL-NA	FE-NA
mean	0	0.075	N/A	0.19	N/A	N/A
min	0	0	N/A	0.15	N/A	N/A
max	0	0.15	N/A	0.31	N/A	N/A
**Sympistis nigrita**						
	AL-AL	FE-FE	NA-NA	AL-FE	AL-NA	FE-NA
mean	0.2817	0	N/A	**2.38**	N/A	N/A
min	0	0	N/A	**2.34**	N/A	N/A
max	0.46	0	N/A	**2.5**	N/A	N/A
**Syngrapha hochenwarthi**						
	AL-AL	FE-FE	NA-NA	AL-FE	AL-NA	FE-NA
mean	0.075	0	N/A	0.2708	N/A	N/A
min	0	0	N/A	0.15	N/A	N/A
max	0.15	0	N/A	0.31	N/A	N/A
**Xestia alpicola**						
	AL-AL	FE-FE	NA-NA	AL-FE	AL-NA	FE-NA
mean	0	0	N/A	0	N/A	N/A
min	0	0	N/A	0	N/A	N/A
max	0	0	N/A	0	N/A	N/A
**Xestia lorezi**						
	AL-AL	FE-FE	NA-NA	AL-FE	AL-NA	FE-NA
mean	N/A	0.2033	0.2067	**2.8733**	1.9333	**2.7311**
min	N/A	0.15	0.15	**2.82**	1.87	**2.5**
max	N/A	0.31	0.31	**2.98**	**2.03**	**3.04**
**Xestia speciosa**						
	AL-AL	FE-FE	NA-NA	AL-FE	AL-NA	FE-NA
mean	0.1196	0.1129	0.2343	**2.4771**	1.8861	1.7014
min	0	0	0	**2.18**	1.55	1.39
max	0.34	0.31	1.7	**2.96**	**2.25**	**2.66**
