# Supplementary material for: Allopatry as a Gordian Knot for Taxonomists: Patterns of DNA Barcode Divergence in Arctic-Alpine Lepidoptera
Source: PLoS One. 2012 Oct 11;7(10):e47214. doi: 10.1371/journal.pone.0047214 (PMC3469483; doi:10.1371/journal.pone.0047214)
Supplement: Table S4 — Minimum, mean and maximum variation in DNA barcodes between allopatric species, which morphologically are considered distinct taxa, but which genetically are closely similar. NA refers to North America, AL to Alps and FE to Fennoscandia. Superscript numbers after the species refer to the areas of occurrence as follows: 1 = Alps, 2 = Fennoscandia, 3 = North America. (PDF) [file pone.0047214.s004.pdf]

Table S4. Minimum, mean and maximum variation in DNA barcodes between allopatric species, which morphologically are considered distinct taxa, but which genetically are closely similar. NA refers to North America, AL to Alps and FE to Fennoscandia. Superscript numbers after the species refer to the areas of occurrence as follows: 1=Alps, 2=Fennoscandia, 3=North America

**Gnorimoschema valesiella[1,2], G. alaskense[3]**						
	NA-NA	FE-FE	AL-AL	AL-FE	AL-NA	FE-NA
mean	0.338	0	0.76	0.746	2.138	2.076
min	0	0	0.32	0.62	1.621	1.869
max	1.08	0	0.98	0.98	2.829	2.505
**Stenoptilia alpinalis[1], S. buvati[1], S. brigantiensis[1], S. mercantourica[1], S. islandica[2]**						
	NA-NA	FE-FE	AL-AL	AL-FE	AL-NA	FE-NA
mean	N/A	0	0.211	0.26	N/A	N/A
min	N/A	0	0	0.15	N/A	N/A
max	N/A	0	0.467	0.47	N/A	N/A
**Erebia medusa[1], E. polaris[2]**						
	NA-NA	FE-FE	AL-AL	AL-FE	AL-NA	FE-NA
mean	N/A	0	0.202	0.125	N/A	N/A
min	N/A	0	0	0	N/A	N/A
max	N/A	0	0.64	0.479	N/A	N/A
**Oeneis glacialis[1], O. norna[2]**						
	NA-NA	FE-FE	AL-AL	AL-FE	AL-NA	FE-NA
mean	N/A	0	0.51	0.273	N/A	N/A
min	N/A	0	0.153	0	N/A	N/A
max	N/A	0	1.015	0.77	N/A	N/A
**Xanthorhoe decoloraria[1,2,3]**						
	NA-NA	FE-FE	AL-AL	AL-FE	AL-NA	FE-NA
mean	0.4	0.407	0.338	0.799	1.817	1.989
min	0	0	0	0	1.55	1.55
max	1.004	0.61	0.77	1.08	2.1	2.37
**Xanthorhoe decoloraria[1,2], X. alticolata[3]**						
	NA-NA	FE-FE	AL-AL	AL-FE	AL-NA	FE-NA
mean	0.209	0.407	0.338	0.799	1.432	1.216
min	0	0	0	0	1.082	0.769
max	0.77	0.61	0.77	1.08	1.711	1.708
**Xanthorhoe incursata[1], X. annotinata[2], X. baffinensis[3]**						
	NA-NA	FE-FE	AL-AL	AL-FE	AL-NA	FE-NA
mean	0.153	0.236	0	0.274	0.077	0.333
min	0	0	0	0.153	0	0.153
max	0.307	0.495	0	0.329	0.377	0.661
**Holoarctia cervini[1], H. puengeleri[2]**						
	NA-NA	FE-FE	AL-AL	AL-FE	AL-NA	FE-NA
mean	N/A	0.115	0.69	0.353	N/A	N/A
min	N/A	0	0	0.741	N/A	N/A

max	N/A	0.172	1.035	0.926	N/A	N/A
**Apamea maillardi[1], A. schildei[2]**						
	NA-NA	FE-FE	AL-AL	AL-FE	AL-NA	FE-NA
mean	N/A	0	0.982	1.388	N/A	N/A
min	N/A	0.527	0	0.153	N/A	N/A
max	N/A	0.926	2.185	2.025	N/A	N/A
**Apamea zeta[1,3], A. schildei[2]**						
	NA-NA	FE-FE	AL-AL	AL-FE	AL-NA	FE-NA
mean	2.191	0	0.278	2.138	2.78	2.734
min	0	0.527	0	1.664	0	2.501
max	3.422	0.926	0.926	2.505	3.67	2.988
**Coenophila subrosea[1,2], C. opacifrons[3]**						
	NA-NA	FE-FE	AL-AL	AL-FE	AL-NA	FE-NA
mean	0	0	0.15	0.076	1.395	1.364
min	0	0	0.15	0	1.474	1.286
max	0	0	0.15	0.16	1.553	1.409
**Sympistis nigrita[1,2], S. zetterstedtii[3]**						
	NA-NA	FE-FE	AL-AL	AL-FE	AL-NA	FE-NA
mean	0.412	0.00	0.25	2.34	2.993	1.263
min	0	0	0	2.36	2.627	1.08
max	2.876	0	0.46	2.5	4.23	2.359
**Xestia rhaetica[1], X. fennica[2]**						
	NA-NA	FE-FE	AL-AL	AL-FE	AL-NA	FE-NA
mean	N/A	0.184	0.175	0.181	N/A	N/A
min	N/A	0	0	0	N/A	N/A
max	N/A	0.315	0.307	0.467	N/A	N/A
